# Supplementary material for: A new variant of the colistin resistance gene MCR-1 with co-resistance to β-lactam antibiotics reveals a potential novel antimicrobial peptide
Source: PLoS Biol. 2023 Dec 13;21(12):e3002433. doi: 10.1371/journal.pbio.3002433 (PMC10786390; doi:10.1371/journal.pbio.3002433)
Supplement: S1 Supplementary materials — (DOCX) [file pbio.3002433.s001.docx]

**Supplementary materials**

**Materials and Methods**

**Strain construction**

We generated three mutant strains (Δ*mrcB*, Δ*ycbB*, and Δ*ycfM*) as previously described[1]. Briefly, three 20-bp spacer fragments targeting the indicated gene (i.e. *mrcB*, *ycbB* or *ycfM*) were digested with *BsmB*I (Thermo Scientific) and inserted into pgRNA (Addgene #44251). To construct the donor DNA, two 500-bp homologous arms were amplified separately and fused together through fusion PCR. Electrocompetent cells containing pREDCas9 (Addgene #71541) were generated as previously described[2]. After electroporation, cultures were incubated for 3 h at 30 °C and then plated on LB agar plates containing 100 µg/ml spectinomycin, 100 µg/ml ampicillin, and 50 µg/ml bleomycin. The mutant strains were confirmed with Sanger (DNA) sequencing. All the plasmids used in this study were listed in S8 Table.

**Plasmid construction**

To express MCR-1 and related variants, DNA fragments encoding indicated proteins were cloned into the plasmid backbone of pACYCDuet-1. The introduction of mutations was carried out through overlapping PCR with appropriate primers (S9 Table). The process for constructing vectors expressing MCR-1 or related mutants under the regulation of the inducible arabinose promoter was as followed. Two target fragments were inserted into the multiple cloning site (MCS) region of pACYCDuet-1 through a one-step cloning strategy. The plasmid backbone pACYCDuet-1 was digested with *HindIII/PstI* (Fast digest, Thermo Fisher). The DNA fragment encoding the arabinose promoter was amplified with the primers araC-HindIII-F/ParaBAD-*mcr-1*-R, carrying 15-20 bp overlapping arms that were homologous to the restricted plasmid. The DNA fragment encoding WT MCR-1 or related mutants was amplified with the primers ParaBAD-*mcr-1*-F/Terminator-PstI-R, carrying 15-20 bp overlapping arms that were homologous to the restricted plasmid. The purified plasmid and target fragments were mixed in a molar ratio of 1:3, followed by adding 2× ClonExpress^®^ Mix (ClonExpress Ultra One Step Cloning Kit, Vazyme) into the reaction and incubating in a thermocycler at 50 °C for 15 minutes. Finally, the well-reacted products were transformed into *E. coli* DH5-α competent cells for enrichment. All target constructs were confirmed by Sanger sequencing. The well-constructed plasmids were transformed into *E. coli* BW25113 cells through electroporation. Similar strategy was also applied for the construction of pACYC-NP-*mcr-1*. During cultivation, 30 µg/ml chloramphenicol (CHL) and 0.2% arabinose were required to maintain the existence of target plasmids and induce the expression of target proteins, respectively. All the plasmids and primers used in this study were listed in S8 and S9 Tables. And the plasmid maps for pACYC-Para-MCR-1 and pACYC-NP-MCR-1 were represented in S19 Fig.

**Homology protein modelling**

To analyse the protein structure of MCR-1 and related variants, homology models for WT MCR-1 and M6 were generated by SWISS-MODEL based on the crystal structure of *Neisseria meningitides* lipid A phosphoethanolamine (PEA) transferase (PDB entry: 5FGN). The PDB files of target proteins were visualized by PyMOL version 2.6 software.

To predict the binding mode of Lipid A to MCR-1, a workflow was designed as shown in S20A Fig. Small molecules always bind with proteins in their lowest energy binding conformations. Hence, we extracted a conformation of Lipid A from RCSB (https://www.rcsb.org/) (PDB code: 5IJD) for our molecular docking simulation. In the experimental complex structure, lipid A is in complex with TLR4/MD-2. Interestingly, we found that despite interacting with different proteins, the lipid A analogues, like eritoran and palmitoyllpid A, represented similar molecular conformations as lipid A. Structural analysis of lipid A and its analogues revealed that the lipid tails bunched together in the centre (S20B Fig). This result further confirmed that the conformation of lipid A obtained for docking is reasonable. The structure of MCR-1 was downloaded from AlphaFold DB, which is a protein database that is computationally predicted and uses the deep learning-based protein structure prediction method Alphafold[3]. SiteMap of Maestro[4] was used to search on the surface of MCR-1 to identify the potential binding sites for any ligand. As a result, a large potential binding site located around the area of the hinge-like linker was discovered with a Site Score in 0.964 (S20C Fig). Then, the residues within this site, like I168, F183, R190, I196 and K204, were used to determine the centre of the binding pocket. An inner grid with a size of 30×30×30 Å and a larger outer box with a size of 35×35×35 Å were set. Then, the mode of SP in Glide of Maestro[5] was chosen to perform docking simulation. Both the ligand and protein were taken as rigid. The radius of van der Waals, initial pose, and cut-off of the pose energy filter were set as 0.5, 10000 and 500 kcal/mol, respectively. The top-ranked binding pose was further structurally optimized by using Prime. This structure was used as the initial structure for molecular dynamics simulation to obtain a more reliable binding pose of lipid A against MCR-1. Six independent simulations (noted as A-F) with a length of 200 ns were performed by using Desmond[6] of Schrödinger2021-2 under the OPLS4 force field. The POPC membrane was assembled to MCR-1 by referencing the positions of the membrane in the homologue protein EptA. The complexed structures were explicitly solvated with TIP3P water molecules under cubic periodic boundary conditions for a 15 Å buffer region. The overlapping water molecules were deleted, and 0.15 M NaCl were added, and the systems were neutralized by adding Na^+^ as counter ions. Brownian motion simulation was used to relax these systems into local energy minimum states separately. An ensemble (NPT) was then applied to maintain the constant temperature (310 K) and pressure (1. 01325 bar) of the systems, and the simulations start with two random initial velocities. The produced trajectories were clustered by all atoms of the MCR1 protein using the Desmond trajectory cluster analysis module and produced five clusters per trajectory. Subsequently, the clustered conformers from all six trajectories were further clustered by heavy atoms of lipid A by using Maestro13.2. Finally, the largest cluster was identified as the representative conformation of MCR1-lipid A complex. The root-mean-square deviations (RMSDs) of the heavy atoms in all simulated trajectories were calculated by Maestro13.2, and the RMSD plot was plotted by Origin version 2021 (S20D Fig).

To assess the impact of mutations of MCR-1 on their binding affinity towards lipid A, the initial binding complex of lipid A and M6 was constructed via the Residue Scanning tool of Maestro based on WT MCR-1, and the residue conformations were correspondingly optimized. The generated M6-lipid A was taken as the input structure of MD simulations for further structure optimization. Both the membrane setting and the parameter settings were also the same as the simulations for the WT complex.

**Growth curve measurements**

Fresh single colonies of *E. coli* BW25113 carrying MCR-1, M6 or empty vector were inoculated into fresh Luria Bertani (LB) broth containing 30 μg/ml CHL and cultivated overnight at 37 °C. The overnight cultures were then adjusted to an OD_600_ ranging from 0.5 to 0.6 with saline and diluted at a ratio of 1:10. The 20 µl diluted cultures were inoculated into 180 µl LB broth containing 0.2% arabinose and 30 µg/ml CHL in a 96-well plate. Three replicates were carried out for each strain. A nanophotometer (NP80, IMPLEN) was utilized to measure the optical density at 600 nm (OD_600_) in each well per hour. Growth curves were visualized with Prism 9 software.

**Agar dilution MIC tests**

To evaluate the antibiotic susceptibility of *E. coli* BW25113 carrying MCR-1, M6 or empty plasmid, MIC assay was performed by the agar dilution method, which was conducted as previously established[7]. To prepare agar plates for MICs assay, antibiotics were added to Mueller–Hinton agar (MHA) containing 30 µg/ml chloramphenicol (CHL) and 0.2% arabinose. Fresh single colonies of the above target strains were inoculated into fresh Mueller–Hinton broth (MHB) containing 30 µg/ml CHL and cultivated overnight at 37 °C. The overnight cultures were then adjusted with saline to an OD_600_ that ranged from 0.5 to 0.6. The well-adjusted cultures were diluted in six gradients, ranging from 10^-1^ to 10^-6^, with saline. For each dilution, 3 µl of culture was spotted onto MHA plates containing antibiotics and incubated at 37 °C overnight. Growth was determined by counting colony-forming units (CFUs). Three replicates were carried out for each strain. The MIC values were defined as the concentration at which bacterial growth was absolutely inhibited. The following antibiotics in different concentrations (highest to lowest) were utilized for the MICs assay: ampicillin (AMP, 2 to 32 μg/ml), cefoxitin (FOX, 2 to 10 μg/ml), imipenem (IMP, 0.5 to 2.5 μg/ml), ceftazidime (CAZ, 0.2 to 0.6 μg/ml), cefotaxime (CTX, 0.1 to 0.5 μg/ml) and meropenem (MEM, 0.01 to 0.1 μg/ml). The results were visualized with Prism 9 software.

**Determination of the outer membrane integrity**

A 1-*N*-phenylnaphthylamine (NPN) uptake assay was performed to determine the outer membrane integrity. NPN is a kind of probe that emits strong fluorescent signals in phospholipid environments, thus, it can verify the permeability of the outer membrane for Gram-negative bacteria[8]. The procedures were performed as previously described[9]. Briefly, fresh single colonies of target strains were inoculated into fresh LB broth containing 30 µg/ml CHL and cultivated overnight at 37 °C. The overnight cultures were then adjusted to an OD_600_ ranging from 0.5 to 0.6 with saline and subcultured into 1 ml of LB broth containing 30 µg/ml CHL and 0.2% arabinose with shaking at 37 °C until the cultures reached an OD_600_ = 0.5. The cells were harvested by centrifugation (4,000 rpm for 3 min), washed twice with assay buffer (5 mM HEPES, 5 mM glucose, pH=7.2) and resuspended in assay buffer to a final OD_600_ = 1. Then, 100 μl of washed cultures and 100 μl of assay buffer containing 20 μM NPN were mixed. The mixture was added into a 96-well half area black opaque plate (Greiner Bio). The fluorescence for each well was immediately monitored with a microplate reader (BioTek) at an excitation wavelength of 350 nm and emission wavelength of 420 nm.

NPN uptake = *F_obs_ - F_ctrl_*

*F_obs_* represents the NPN uptake of different cultures, and *F_ctrl_* represents the background signal without the addition of bacterial culture. Three replicates were carried out for each strain. The results were visualized with Prism 9 software.

**Determination of the inner membrane integrity**

A PI (propidium iodide) staining assay was performed to determine the inner membrane integrity. Acting as a dye crossing compromised bacterial membranes and binding with DNA and RNA inside of damaged cells[10], PI was utilized to identify dead cells or those with irreversibly damaged membranes. Therefore, the uptake of PI can reflect the inner membrane permeability of Gram-negative bacteria. The process for PI staining was performed as follows: fresh single colonies of the above target strains were inoculated into fresh LB broth containing 30 µg/ml CHL and cultivated overnight at 37 °C. The overnight cultures were then adjusted to an OD_600_ ranging from 0.5 to 0.6 with saline and subcultured into 2 ml of LB broth containing 30 μg/ml CHL and 0.2% arabinose with shaking at 37 °C. Subsequently, samples were collected at 4 hr and 8 hr after subculture. The cells were harvested by centrifugation (4,000 rpm for 3 min), washed twice with saline and resuspended in 95 µl buffer A (PI staining kit, Sangon Biotech). Then, 5 µl of PI dye was added to each resuspended culture and incubated in the dark for 20 min. The percentage of the PI-positive population was verified by using a flow cytometer (Gallios10, Beckman). The sample without staining with PI was set as a control for gating. The data were analysed with FlowJo version 10 software and visualized with Prism 9 software.

**Screening of the MCR-1 mutant library against a variety of antibiotics**

The MCR-1 mutant library in the background of *E. coli* BW25113 constructed in our previous research was applied for antibiotics susceptibility testing to screen out MCR-1 mutant exhibited co-resistance upon other classes of antibiotic. The indicated mutant library was cloned into the plasmid backbone of pACYCDuet-1. The expression of target pool was regulated by arabinose promoter and required the induction with 0.2% arabinose. This library contains a total of 171,769 variants for the *mcr-1* gene, which covers 99.96% of the single-nucleotide mutations. The bacterial strain was kept in a final concentration of 30% glycerol at -80°C. Unless otherwise stated, the bacterial glycerol stocks were revived through cultivation in LB broth at 37°C with shaking. The *E. coli* strains carrying empty plasmid or WT MCR-1 were set as control. Total 13 types of antibiotics were selected for testing: ampicillin (AMP), ceftazidime (CAZ), imipenem (IMP), streptomycin (SM), tetracycline (TET), nalidixic acid (NAL), vancomycin (VAN), cefotaxime (CTX), cefepime (FEP), ceftriaxone (CRO), ertapenem (ETP), meropenem (MEM) and cefoxitin (FOX). LB agar plate containing above antibiotics in the concentrations of 0.8×, 1× or 2× MICs were prepared (S1 and S2 Tables), respectively. To ensure that all the mutation genotypes in the MCR-1 mutant library were present on each plate at least once, the coupon collector problem was applied as follows:

$$M=-N\frac{log(N)}{log(P)}$$

where *M* represents the number of cells on each plate, *N* is the number of total mutation genotypes and *P* is the probability of coverage for all the genotypes.

For P = 99.9%:

$M=-4858\times\frac{\log\left( 4858 \right)}{\log\left( 0.999 \right)}=$4.2×10^9^

Therefore, approximately 1×10^11^ cells were plated on each agar plate to achieve 99.9% coverage of all the single-point mutation genotypes. Same amounts of cells were also plated on the LB agar plates containing indicated antibiotics for the groups of empty plasmid control and WT MCR-1. After incubation at 37 °C overnight, CFUs were counted to evaluate viability.

To further verify the genotype of the MCR-1 mutant displaying co-resistance towards β-lactams antibiotics, 50 isolates of the MCR-1 library were selected from the plates containing CAZ or AMP in the concentration of 2x MICs. The plasmids harbouring *mcr-1* gene were extracted from the selected isolates. Next, to remove the interference caused by chromosomal mutation, the well-extracted plasmids were transformed into *E. coli* BW25113 competent cell for generating reconstructed strains. Meanwhile, the *mcr-1* genotypes of selected isolates were confirmed by Sanger-sequencing. Additionally, the reconstructed strains were applied for susceptibility testing among CAZ, AMP and FOX as mentioned above. The *E. coli* strains carrying empty plasmid or WT MCR-1 were set as control, CFUs were counted to evaluate viability after incubation at 37 °C overnight.

**Morphological analysis by scanning electron microscopy (SEM) to evaluate the bacterial cell wall surface**

Scanning electron microscopy (SEM) was performed to observe the change on bacterial surface. The main process was similar to that previously described[11]. For BW25113 expressing MCR-1 or M6, exponential phase cultures were collected, and BW25113 carrying the empty plasmid vector was set as a control. For ATCC 25922 treated with 24AA-2M or 19AA-2M-tag, cultures were grown to exponential phase and then treated with 92.5μM indicated peptides for 1 hr, and ATCC 25922 treated with DMSO was set as a control. All the samples were harvested by centrifugation (4,000 rpm for 3 min) and fixed with 2% glutaraldehyde (Servicebio) for at least 4 hr at room temperature, followed by dehydration with a graded ethanol series and air drying. The dried powder was loaded on a rotating stage, sputtered with gold by using a vacuum coater (Leica EM ACE200) and coated with a 0.1 mm gold layer. Microscopy was performed with a desktop field emission scanning electron microscope (Phenom Pharos G2, Thermo Fisher Scientific). Images were collected using a secondary electron detector, and the acceleration voltage was adjusted to 10 kV. SEM images were recorded at magnifications ranging from 20,000× to 100,000×.

**Morphological analysis by transmission electron microscopy (TEM) to evaluate cell wall morphology**

Transmission electron microscopy (TEM) was performed to explore the cell wall morphological change of *E. coli* BW25113 carrying MCR-1, M6 or empty plasmid. Exponential phase cultures were collected and harvested by centrifugation (4,000 rpm for 3 min), followed by fixation with 2% glutaraldehyde (Servicebio) for at least 4 hr at room temperature. The sample pellets were embedded in a 1 mm cube containing 4% low melting point agarose and postfixed with osmium tetroxide for 2 hr. The specimens were then dehydrated in a graded series of ethanol, transferred to propylene oxide and embedded in Epon according to standard procedures. Thin (80 nm) sections were cut and collected on copper grids. After staining with uranyl acetate and lead citrate, the specimens were subsequently examined with a field emission transmission electron microscope (JEM-2100, JEOL) operated at an accelerating voltage of 80 kV. TEM images were recorded at magnifications of 20,000× to 80,000×.

**Quantitative real-time PCR to assess transcription of envelope and transpeptidase genes**

To verify the change in the transcriptional level of genes related to envelope responses and LDTs (L, D-transpeptidases) pathway, mRNA was extracted from *E. coli* BW25113 carrying MCR-1, M6 or empty plasmid for quantitative real-time PCR (q-PCR). Overnight cultures of target strains were sub-cultured into 1 ml fresh LB broth containing 30 µg/ml CHL and 0.2% arabinose. After induction for 2 hr, exponential phase cultures were collected and cells were harvested by centrifugation (4,000 rpm for 3 min). For each sample, cell pellet was resuspended in RNA-easy Isolation Reagent (Vazyme). Total RNA was precipitated by adding isopropanol and was collected by centrifugation (37 °C, 12,000 rpm for 10 min). After discarding supernatant, the mRNA pellet was washed with 75% ethanol. The mRNA pellet was dehydrated through air drying and dissolved in RNase-free H_2_O. The contaminating genomic DNA was digested with gDNA wiper Mix (Vazyme). cDNA was prepared from purified mRNA with HiScriptII qRT SuperMix II (Vazyme) through reverse transcription. The cDNA levels of target genes were then quantified by quantitative real-time PCR (qRT‒PCR) on a CFX96 cycler (BIO RAD) by using AceQ Universal SYBR qPCR Master Mix (Vazyme) according to the manufacturers’ protocol. All primers were determined to be >95% efficient. Signals were normalized to those of the transcript of the housekeeping gene *rpoB* and quantified with ΔΔCT analysis.

**Expression level quantification of PbgA and LPS**

To verify the influence of MCR-1 or M6 expression on bacterial LPS homeostasis, immunoblot analysis was performed to evaluate the expression levels of PbgA (a periplasmic lipid A sensor, labelled with HA-tag at C-terminal) and LPS. Briefly, fresh single colonies of target strains were inoculated into 5 ml fresh Luria Bertani (LB) broth containing 30 µg/ml CHL and 0.2% arabinose. After induction for 2 hr, exponential phase cultures were collected and cells were harvested by centrifugation (4,000 rpm for 3 min). For each sample, 1 ml RIPA lysis buffer (Beyotime) was added to resuspend the cell pellet, and the bacillus was further broken down with an ultrasonic processor (SONICS, VCX 130). After centrifugation (10,000 rpm for 1 min), the supernatant of the lysate was stored, and the protein concentration of each sample was quantified by using the Bradford Protein Assay Kit (Beyotime) according to the manufacturer’s protocol. Next, the samples were electrophoresed on a 12% Bis-Tris SDS-polyacrylamide gel. After electrophoresis, proteins and LPS were transferred to a polyvinylidene difluoride (PVDF) membrane (Thermo Fisher) using the Trans-Blot Turbo Transfer System (BIO RAD). Primary antibodies against RpoB (BioLegend), LPS core (Hycult Biotech) and HA-tag (Cell Signalling) were used at dilutions of 1:100,000, 1:1,000 and 1:10,000, respectively. Goat anti-rabbit horseradish peroxidase (HRP) conjugate (Zen Bioscience) and rabbit anti-mouse HRP conjugate (Dingguo Biology) secondary antibodies were each used at a 1:10,000 dilution. After processing chemiluminescent detection with a ChemiDoc Touch Imaging System (BIO RAD), the protein levels were quantified with Fuji software, in which the expression level of RpoB was regarded as a reference. Three replicates were performed for each sample.

**Preparation of spheroplasts**

Spheroplasts of were generated as mentioned previously[12]. Briefly, overnight cultures of indicated strains were subcultured into fresh LB medium containing 30 μg/ml CHL and 0.2% arabinose with the ratio of 1:100. Cultures were then incubated at 37°C with shaking. Logarithmic phase cultures were collected and washed twice by centrifuging (3273× g, 20 min, 4°C), resuspending firstly in 10 ml Tris buffer (0.03 M, pH 8.0) and subsequently in Tris buffer (0.03 M, pH 8.0) containing 20% sucrose. EDTA (250 ml, 10 mg/ml) and lysozyme (1 ml, 10 mg/ml) were added to remove the OM and periplasm respectively, and the cell suspension was incubated for 1 hr in a water bath shaker at 30°C. The resulting spheroplasts produced were harvested by mild centrifugation (2000× g, 20 min, 4°C), and the morphology was observed by microscopy.

**Fluorescent imaging to assess membrane shrinkage**

Since membrane shrinkage of *E. coli* was accompanied by the formation of periplasmic foci[13], we labelled the periplasm with super-folded GFP (sfGFP) and the cytoplasm with mCherry to construct a two-fluorescent reporter system. For each strain, stationary phase culture was collected after induction with 0.2% arabinose, and 5 μl of bacterial culture was placed on a gel pad containing 1% agarose and covered with a coverslip. The GFP and mCherry signals were recorded with a fluorescence microscope (Olympus BX63) on phase contrast equipped with a 100× oil immersion objective and a xenon lamp. The images merged with the two fluorescent signals were processed with Fuji software.

**Fluorescent imaging to assess membrane voltage**

The construction of genetically-encoded membrane voltage sensor Vibac2 was as described in previous research[14]. ViBac2 is a double-channel fusion protein that emits green and red fluorescence. The fluorescence intensity of GFP (Ex=488 nm, Em=512 nm) responds to membrane voltage, and the mCherry (Ex=561 nm, Em=610) is used to normalize protein expression. Thus, the fluorescence ratio indicates the relative membrane voltage in *E. coli* cells. The plasmids encoding MCR-1 or M6 were transformed into *E. coli* BW25113 competent cell expressing Vibac2. Strain harbouring pACYCDuet-1 empty plasmid was set as control. For each strain, stationary phase culture was collected after induction with 0.2% arabinose, and 5 μl of bacterial culture was placed on a gel pad containing 1% agarose and covered with a coverslip. The GFP and mCherry signals were recorded with a fluorescence microscope (Olympus BX63) on phase contrast equipped with a 100× oil immersion objective and a xenon lamp. The images merged with the two fluorescent signals were processed with Fuji software.

**Label-free quantitative proteome analysis**

To profile the proteomic characteristics of the M6-positive strain, a label-free quantitative proteome analysis was performed to compare the differentially expressed proteins between *E. coli* BW25113 carrying MCR-1 and BW25113 carrying M6. The process for sample preparation was similar to that mentioned previously[15]. In brief, fresh single colonies of target strains were inoculated into fresh Luria Bertani (LB) broth containing 30 μg/ml CHL and 0.2% arabinose, and three replicates were carried out for each strain. After induction for 2 hr, samples were collected and harvested by centrifugation (4,000 rpm for 3 min). The cell pellets were sent to APTBIO Co. (Shanghai, China) for proteomic analysis. A volcano map was constructed with R studio (version 3.6.1) to show the comprehensive changes induced by the expression of M6 in *E. coli* compared with the *mcr-1*^+^ strain. The differentially expressed proteins were mapped to the KEGG database (<http://geneontology.org/>) and Gene Ontology (GO) terms (with Blast2GO software) for enrichment analysis, and the results were visualized by R studio (version 3.6.1). Additionally, the studied proteins were subjected to protein‒protein interaction (PPI) analysis through STRING (<http://string-db.org/>), and the results were visualized by Cytoscape software.

**SDS sensitivity assay to evaluate cell wall permeability**

The sodium dodecyl sulfate (SDS)–ethylenediaminetetraacetic acid (EDTA) sensitivity assay was performed with an agar dilution strategy to evaluate the cell wall permeability. To prepare agar plates containing SDS in different concentrations, SDS was added to Mueller–Hinton agar (MHA) containing 30 µg/ml CHL and 0.2% arabinose. EDTA was also added into the agar to a final concentration of 100 µM to increase the permeability of the bacterial cell wall. Fresh single colonies of indicated strains were inoculated into fresh Mueller–Hinton broth (MHB) containing 30 µg/ml CHL and cultivated at 37 °C overnight. The overnight cultures were then adjusted to an OD_600_ ranging from 0.5 to 0.6 with saline. The well-adjusted cultures were then diluted with saline in six gradients, ranging from 10^-1^ to 10^-6^. For each dilution, 3 µl of culture was spotted onto MHA plates containing SDS-EDTA and were incubated at 37 °C overnight. Growth was determined by counting CFUs. Three replicates were carried out for each strain. The inhibition values were defined as the concentration at which bacterial growth was absolutely inhibited.

**Biolayer interferometry**

An *in vitro* interaction assay was carried out to verify the affinity between lipid A and synthetic peptides derived from the linker domain of WT MCR-1 and M6, named peptide MCR-1 and peptide M6, respectively (S10 Table). Both peptides were labelled with biotin at the C-terminals. Kdo_2_-lipid A (Avanti Polar Lipids) and biotinylated peptide (GenScript) stock powders were dissolved in DMSO and diluted with PBST. The assay was performed at 25 °C in PBST buffer containing 10% DMSO. The biotin-blocked reference streptavidin (SA) biosensor biosensors (Sartorius) were soaked with PBST containing 10% DMSO buffer for 10 min, and biotinylated peptides were loaded onto SA biosensors to a response of approximately 0.5 nm. Target peptides were bound to Kdo_2_-lipid A in various concentrations (150, 100, 50, 25 and 10 μM) with 300 s association and dissociation steps. Assays were performed in triplicate on an Octet Red384 (Sartorius). Dissociation constants for peptide MCR-1 and peptide M6 interactions with Kdo_2_-lipidA were estimated by plotting response values at equilibrium as a function of concentration and fit to a global specific binding with the Hill slope model in Prism 9.

**Localization of synthetic peptide in *E. coli***

Fresh single colonies of *E. coli* ATCC 25922 was inoculated into fresh LB broth and cultivated at 37 °C overnight. The cultures were then subcultured into 1 ml LB broth with shaking at 37 °C. Logarithmic phase cultures were harvested by centrifugation (4,000 rpm for 3 min), and cell pellets washed twice with 1× PBS. After resuspension with 1 ml 1× PBS, 2 µl FM4-64 (for membrane staining) were added. The mixtures were then incubated at dark for 1 hr. After washing twice with 1× PBS, the resuspended cultures were then stained with 1 ml DAPI (for nucleic acids staining) for 15 min. Next, after washing twice with 1× PBS, 20 µl or 10 µl of 10 mg/ml FITC-labelled peptides were the added into the resuspended cultures. After incubation at dark for 2 hr, the mixtures were washed twice with 1× PBS, following by resuspension with 100 µl 1× PBS. The stained cells (3 µl) were spotted on a microscope slide and covered with 1% (w/v) agar slices and a glass coverslip. The localization of the FITC signal were determined by observation with fluorescent microscope (Olympus BX63).

**Extraction of lipid A from *E. coli***

To identify the distribution of modified and unmodified lipid A on the IM of indicated strains, LPS was extracted from the whole cells and spheroplasts of *E. coli* BW25113 bearing MCR-1 or M6. Briefly, overnight cultures of target strains were inoculated into 100ml LB broth containing 30 µg/ml CHL and 0.2% arabinose with the ratio of 1:100. After cultivation for 2 hr at 37 °C, spheroplasts were generated as mentioned previously[12]. All the samples were collected by centrifugation (8,000 rpm for 5 min). Next, for each sample, cellular LPS was extracted by using LPS extraction kit (BestBio) according to the manufacturers’ protocol. The well-extracted LPS was subsequently hydrolysed into lipid A based on previous research[16]. LPS was cleaved by hydrolysis in 20 mM Na acetic acid-sodium acetate buffer, pH 4.5, and 1% Na dodecyl sulfate at 100°C for 1 h at a concentration of 5 mg/ml. Next, lipid A was recovered from the lyophilized residue by two extractions with a volume of 0.5 ml of a chloroform-methanol-water (3:2:0.25, v/v) mixture. The air-dried lipid A was analyzed on a Bruker Microflex mass spectrometer (Bruker Daltonics, Billerica, MA) in the negative-ion mode with reflectron mode. The relative height of ion peak for lipid A or PEA-lipid A were counted to determine their relative abundance.

**Growth inhibition assay**

To verify the bacteriostatic activity of the synthetic peptides towards *E. coli* ATCC 25922, a growth inhibition assay was performed. The density of the overnight cultures was adjusted with saline to OD_600_=0.5 and further diluted at a ratio of 1:10. The diluted culture was aliquoted for treatment with synthetic peptides as follows: 24AA-WT, 24AA-2M and 19AA-2M-tag. All the peptides were in concentrations of 370, 185 and 92.5 μM. The samples without drug treatment were used as controls. For each treatment, 100 µl of culture was sampled 0, 3, 6 and 24 hr after the addition of target peptides. The collected sample was diluted with saline in six gradients, ranging from 10^-1^ to 10^-6^. Next, 3 μl of each dilution was spotted onto LB agar plates. Each treatment was repeated in triplicate. After incubation at 37 °C overnight, CFUs were counted to evaluate the antimicrobial effect. All the synthetic peptides used in this study were listed in S10 Table.

**Checkerboard assay**

Synergy measurement was utilized to determine the antimicrobial effect of colistin (CT) and 19AA-2M-tag upon *E. coli* ATCC 25922. The checkerboard assay was set up in a 96-well plate. Briefly, Columns 2 to 12 contained 2-fold serial dilutions of colistin (ranging from 10 to 0.0098 μg/ml), and Rows A to G contained 2-fold serial dilutions of 19AA-2M-tag (ranging from 92.5 to 1.45 μM). Column 1 contained a serial dilution of 19AA-2M-tag alone (ranging from 92.5 to 1.45 μM), while Row H contained a serial dilution of colistin alone (ranging from 10 to 0.0098 μg/ml). These two groups were used as controls to determine the MIC value of colistin or 19AA-2M-tag. *E. coli* ATCC 25922 was cultured to exponential phase and adjusted to a density of OD_600_=0.5 by diluting with LB broth. After dilution at a ratio of 1:10, 20 µl of diluted culture was added to 180 µl of LB broth containing colistin and/or 19AA-2M-tag in a 96-well plate. A nanophotometer (NP80, IMPLEN) was utilized to measure the optical density at 600 nm (OD_600_) in each well before and after incubation at 37 °C for 16 hr. The fractional inhibitory concentration index (FICI) was calculated as follows:

$$\mathrm{FICI}=\frac{\mathrm{MIC}A combined}{\mathrm{MIC}A alone}+\frac{\mathrm{MIC}B combined}{\mathrm{MIC}B alone}$$

where MIC _A combined_ and MIC _B combined_ are the MICs of each drug in combination, while MIC _A alone_ and MIC _B alone_ are the MICs of each drug individually.

**Murine red blood cell lysis assay**

To verify the cytotoxicity of 24AA-2M and 19AA-2M-tag towards murine red blood cells, the lactate dehydrogenase (LDH) assay was utilized to measure cell permeation. Each peptide was diluted with saline to a final concentration of 185 µM. Mouse blood samples were mixed with 100 µl of diluted peptide in a 96-well plate (3000 cells/well). The LDH-based TOX-7 kit (Sigma‒Aldrich) was used for quantification of LDH release from the cells. The results represent the mean values from triplicate measurements and were visualized by Prism 9.

**Murine infection model**

The effectiveness of drug combination containing colistin and 19AA-2M-tag was evaluated against *in vivo* infection. Ten-week-old C57BL/6 mice were infected with 1×10^7^ CFUs of *E. coli* ATCC 25922 or CRE through intraperitoneal injection. 200 μg 19AA-2M-tag was supplemented to the infected mouse by intraperitoneal injection as treatment 2hr after infection. The individuals without treatment were set as controls, and each condition was carried out in triplicate. Mice were sacrificed 24 hr after infection, and bacterial burdens in the liver and spleen were determined by spotting serial dilutions of tissue homogenates on LB plates. After incubation at 37 °C overnight, CFUs were counted to evaluate the antimicrobial effect.

**Statistical analysis**

Statistical analysis was performed using Prism (version 9, GraphPad Software). Data were analysed using the paired Student’s t test, and in the comparisons of data from three or more conditions, analysis of variance (ANOVA) was used. A *P* value of 0.05 or less was considered statistically significant.

**References**

1. Feng S, Liang W, Li J, Chen Y, Zhou D, Liang L, et al. MCR-1-dependent lipid remodelling compromises the viability of Gram-negative bacteria. Emerg Microbes Infect. 2022;11(1):1236-49. Epub 2022/04/20. doi: 10.1080/22221751.2022.2065934. PubMed PMID: 35437117; PubMed Central PMCID: PMCPMC9067951.

2. Li Y, Lin Z, Huang C, Zhang Y, Wang Z, Tang YJ, et al. Metabolic engineering of Escherichia coli using CRISPR-Cas9 meditated genome editing. Metab Eng. 2015;31:13-21. Epub 2015/07/05. doi: 10.1016/j.ymben.2015.06.006. PubMed PMID: 26141150.

3. Jumper J, Evans R, Pritzel A, Green T, Figurnov M, Ronneberger O, et al. Highly accurate protein structure prediction with AlphaFold. Nature. 2021;596(7873):583-9. Epub 2021/07/16. doi: 10.1038/s41586-021-03819-2. PubMed PMID: 34265844; PubMed Central PMCID: PMCPMC8371605.

4. Halgren TA. Identifying and characterizing binding sites and assessing druggability. J Chem Inf Model. 2009;49(2):377-89. Epub 2009/05/13. doi: 10.1021/ci800324m. PubMed PMID: 19434839.

5. Halgren TA, Murphy RB, Friesner RA, Beard HS, Frye LL, Pollard WT, et al. Glide: a new approach for rapid, accurate docking and scoring. 2. Enrichment factors in database screening. J Med Chem. 2004;47(7):1750-9. Epub 2004/03/19. doi: 10.1021/jm030644s. PubMed PMID: 15027866.

6. Bowers KJ, Chow DE, Xu H, Dror RO, Eastwood MP, Gregersen BA, et al., editors. Scalable Algorithms for Molecular Dynamics Simulations on Commodity Clusters. SC '06: Proceedings of the 2006 ACM/IEEE Conference on Supercomputing; 2006 11-17 Nov. 2006.

7. Wiegand I, Hilpert K, Hancock RE. Agar and broth dilution methods to determine the minimal inhibitory concentration (MIC) of antimicrobial substances. Nat Protoc. 2008;3(2):163-75. Epub 2008/02/16. doi: 10.1038/nprot.2007.521. PubMed PMID: 18274517.

8. Helander IM, Mattila-Sandholm T. Fluorometric assessment of gram-negative bacterial permeabilization. J Appl Microbiol. 2000;88(2):213-9. Epub 2000/03/29. doi: 10.1046/j.1365-2672.2000.00971.x. PubMed PMID: 10735988.

9. Muheim C, Gotzke H, Eriksson AU, Lindberg S, Lauritsen I, Norholm MHH, et al. Increasing the permeability of Escherichia coli using MAC13243. Sci Rep. 2017;7(1):17629. Epub 2017/12/17. doi: 10.1038/s41598-017-17772-6. PubMed PMID: 29247166; PubMed Central PMCID: PMCPMC5732295.

10. Rosenberg M, Azevedo NF, Ivask A. Propidium iodide staining underestimates viability of adherent bacterial cells. Sci Rep. 2019;9(1):6483. Epub 2019/04/26. doi: 10.1038/s41598-019-42906-3. PubMed PMID: 31019274; PubMed Central PMCID: PMCPMC6482146.

11. Hartmann M, Berditsch M, Hawecker J, Ardakani MF, Gerthsen D, Ulrich AS. Damage of the bacterial cell envelope by antimicrobial peptides gramicidin S and PGLa as revealed by transmission and scanning electron microscopy. Antimicrob Agents Chemother. 2010;54(8):3132-42. Epub 2010/06/10. doi: 10.1128/AAC.00124-10. PubMed PMID: 20530225; PubMed Central PMCID: PMCPMC2916356.

12. Sabnis A, Hagart KL, Klockner A, Becce M, Evans LE, Furniss RCD, et al. Colistin kills bacteria by targeting lipopolysaccharide in the cytoplasmic membrane. Elife. 2021;10. Epub 2021/04/07. doi: 10.7554/eLife.65836. PubMed PMID: 33821795; PubMed Central PMCID: PMCPMC8096433.

13. Shi H, Westfall CS, Kao J, Odermatt PD, Anderson SE, Cesar S, et al. Starvation induces shrinkage of the bacterial cytoplasm. Proc Natl Acad Sci U S A. 2021;118(24). Epub 2021/06/13. doi: 10.1073/pnas.2104686118. PubMed PMID: 34117124; PubMed Central PMCID: PMCPMC8214708.

14. Jin X, Zhang X, Ding X, Tian T, Tseng CK, Luo X, et al. Sensitive bacterial V(m) sensors revealed the excitability of bacterial V(m) and its role in antibiotic tolerance. Proc Natl Acad Sci U S A. 2023;120(3):e2208348120. Epub 2023/01/10. doi: 10.1073/pnas.2208348120. PubMed PMID: 36623202; PubMed Central PMCID: PMCPMC9934018.

15. Li H, Wang Y, Meng Q, Wang Y, Xia G, Xia X, et al. Comprehensive proteomic and metabolomic profiling of mcr-1-mediated colistin resistance in Escherichia coli. Int J Antimicrob Agents. 2019;53(6):795-804. Epub 2019/02/28. doi: 10.1016/j.ijantimicag.2019.02.014. PubMed PMID: 30811973.

16. El Hamidi A, Tirsoaga A, Novikov A, Hussein A, Caroff M. Microextraction of bacterial lipid A: easy and rapid method for mass spectrometric characterization. J Lipid Res. 2005;46(8):1773-8. Epub 2005/06/03. doi: 10.1194/jlr.D500014-JLR200. PubMed PMID: 15930524.
