# Supplementary material for: A new variant of the colistin resistance gene MCR-1 with co-resistance to β-lactam antibiotics reveals a potential novel antimicrobial peptide
Source: PLoS Biol. 2023 Dec 13;21(12):e3002433. doi: 10.1371/journal.pbio.3002433 (PMC10786390; doi:10.1371/journal.pbio.3002433)
Supplement: S1 Fig — (A) The schematic illustration shows the process of screening MCR-1 mutants with reduced susceptibility towards β-lactams. The variant library containing 171969 genotypes was cloned into the medium-copy plasmid pACYDuet-1 to generate the E. coli BW25113 strain pool. E. coli strains carrying WT MCR-1 or empty plasmid were set as control. Logarithmic-phase cultures of the 3 strains were first induced with 0.2% arabinose for 2 h, followed by plating on LB agar plates containing AMP, CAZ, IMP, SM, TET, NAL, or VAN. All antibiotics were in the concentrations of 0.8×, 1×, 1.5×, and 2× MICs. After incubation at 37°C for 16 h, the number of surviving bacilli was counted to evaluate antibiotic susceptibility (B). It appears that certain MCR-1 variants exhibited reduced sensitivity to β-lactam antibiotics. To confirm the sensitivity of β-lactam antibiotics among the 3 strains, a similar process was performed, and well-induced cultures were plated on LB agar plates containing CTX, FEP, CRO, ETP, MEM, or FOX. CFUs were counted after incubation at 37°C for 16 h (C). Fifty colonies of the mcr-1 library in the background of E. coli BW25113 were selected from the plates containing CAZ or AMP. For each isolate, the pACYCDuet-1 plasmid carrying mcr-1 variants was extracted, followed by transformation into E. coli BW25113 electroporation competent cells to eliminate influence caused by chromosomal mutation. Next, reconstructed strains were subjected to agar dilution MICs test to verify susceptibility to CAZ, AMP, or FOX. In addition, the mcr-1 genotypes of the target isolates were verified through Sanger sequencing. Both panels (B) and (C) were visualized with Prism 9 software. All the above-described experiments were performed 3 times with similar results. Error bars indicate standard errors of the means (SEMs) for 3 biological replicates. The raw data underlying this figure can be found in S1 Data. (PDF) [file pbio.3002433.s002.pdf]

Supplementary figures

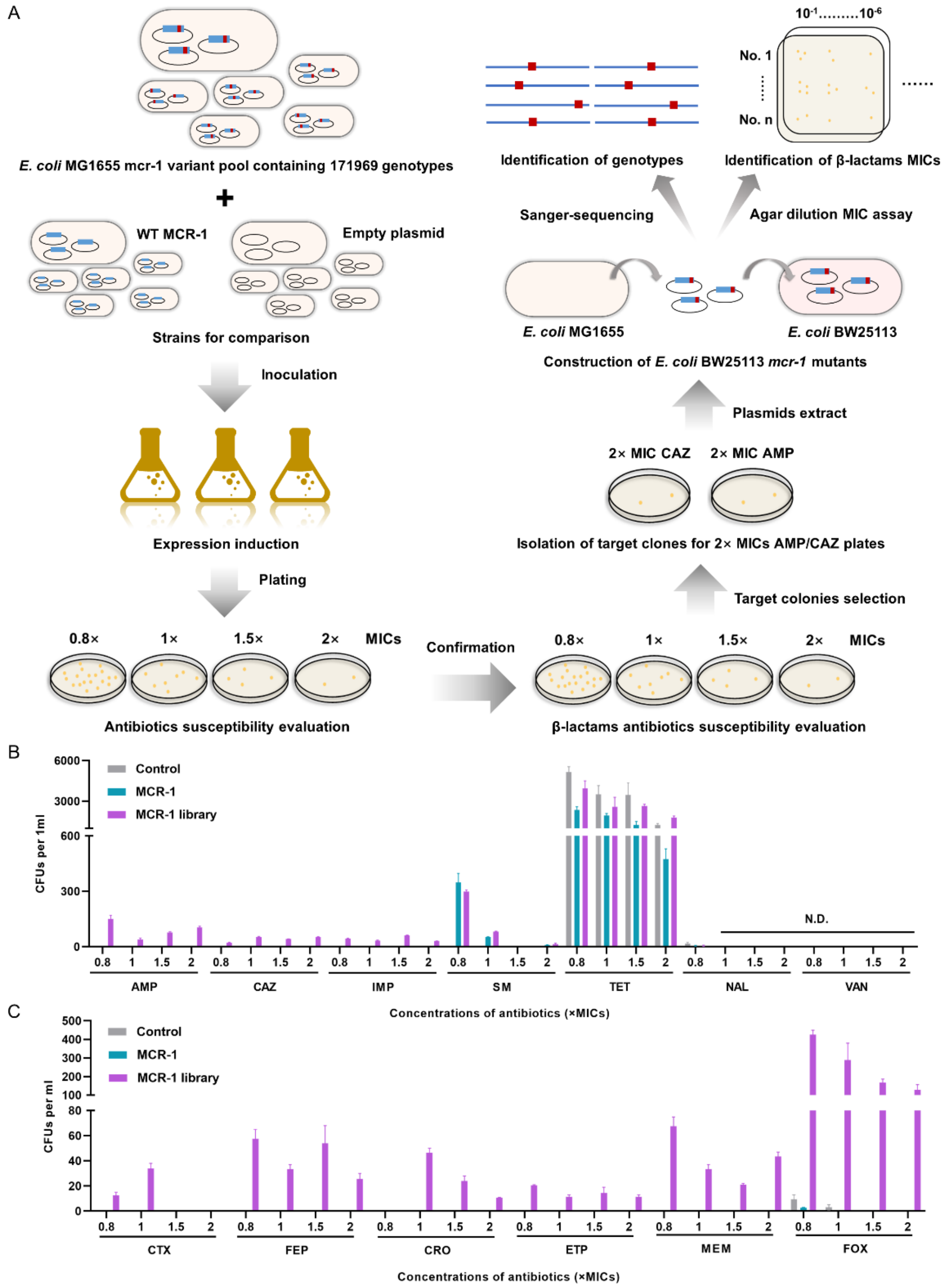

Figure S1. Identification of isolates with reduced sensitivity towards β-lactam antibiotics from the MCR-1 mutant library.

(A) The schematic illustration shows the process of screening MCR-1 mutants with reduced susceptibility towards β-lactams. The variant library containing 171969 genotypes was cloned into the medium-copy plasmid pACYDuet-1 to generate the *E. coli* BW25113 strain pool. *E. coli* strains carrying WT MCR-1 or empty plasmid were set as control. Logarithmic-phase cultures of the three strains were first induced with 0.2% arabinose for 2 hr, followed by plating on LB agar plates containing AMP, CAZ, IMP, SM, TET, NAL or VAN. All antibiotics were in the concentrations of 0.8×, 1×, 1.5× and 2× MICs. After incubation at 37 °C for 16 hr, the number of surviving bacilli was counted to evaluate antibiotic

susceptibility **(B)**. It appears that certain MCR-1 variants exhibited reduced sensitivity to β-lactam antibiotics. To confirm the sensitivity of β-lactam antibiotics among the three strains, a similar process was performed, and well-induced cultures were plated on LB agar plates containing CTX, FEP, CRO, ETP, MEM or FOX. CFUs were counted after incubation at 37 °C for 16 hr **(C)**. Fifty colonies of the *mcr-1* library in the background of *E. coli* BW25113 were selected from the plates containing CAZ or AMP. For each isolate, the pACYCDuet-1 plasmid carrying *mcr-1* variants was extracted, followed by transformation into *E. coli* BW25113 electroporation competent cells to eliminate influence caused by chromosomal mutation. Next, reconstructed strains were subjected to agar dilution MICs test to verify susceptibility to CAZ, AMP or FOX. In addition, the *mcr-1* genotypes of the target isolates were verified through Sanger sequencing. Both Panels **(B)** and **(C)** were visualized with Prism 9 software. All the above-described experiments were performed three times with similar results. Error bars indicate standard errors of the means (SEMs) for three biological replicates. The raw data underlying this Figure can be found in S1_data.
