# Supplementary material for: A new variant of the colistin resistance gene MCR-1 with co-resistance to β-lactam antibiotics reveals a potential novel antimicrobial peptide
Source: PLoS Biol. 2023 Dec 13;21(12):e3002433. doi: 10.1371/journal.pbio.3002433 (PMC10786390; doi:10.1371/journal.pbio.3002433)
Supplement: S2 Fig — A surface representation shows the conservation analysis calculated across 106 MCR family proteins, in which red represents low homologous positions and green represents high conservation. The linker region and the mutated residues are shown in cartoon and sticky representations, respectively. The protein structure was analyzed and plotted by PyMOL 2.6 software. (PDF) [file pbio.3002433.s003.pdf]

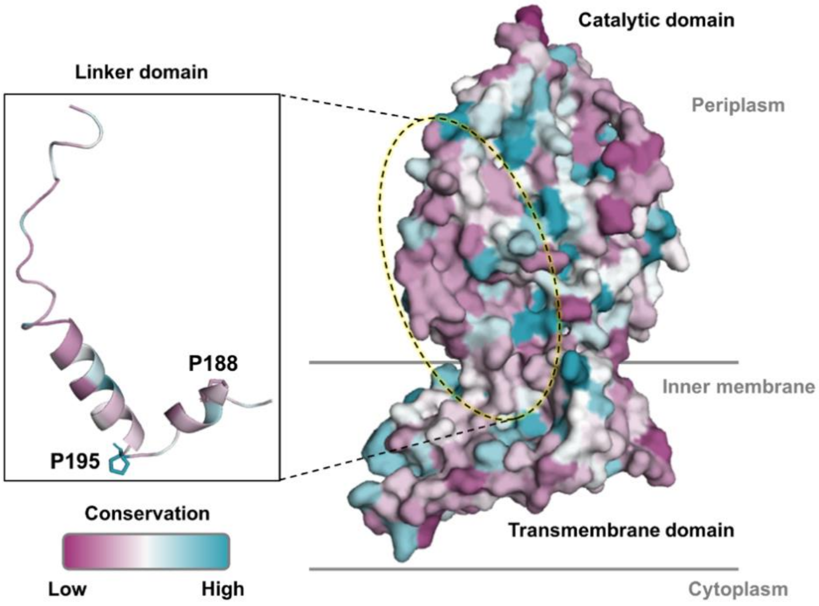

Figure S2. Conservation of the protein structure of the MCR family.

A surface representation shows the conservation analysis calculated across 106 MCR family proteins, in which red represents low homologous positions and green represents high conservation. The linker region and the mutated residues are shown in cartoon and sticky representations, respectively. The protein structure was analysed and plotted by PyMOL 2.6 software.
