# Supplementary material for: A new variant of the colistin resistance gene MCR-1 with co-resistance to β-lactam antibiotics reveals a potential novel antimicrobial peptide
Source: PLoS Biol. 2023 Dec 13;21(12):e3002433. doi: 10.1371/journal.pbio.3002433 (PMC10786390; doi:10.1371/journal.pbio.3002433)
Supplement: S3 Fig — (A) Zone of inhibition of E. coli BW25113 harboring empty plasmid, MCR-1 or M6 generated by disk diffusion method. (B) Different levels of antibiotic sensitivity in E. coli expressing either wild-type MCR-1, M6 or its single point mutants. The raw data underlying this figure can be found in S1 Data. (PDF) [file pbio.3002433.s004.pdf]

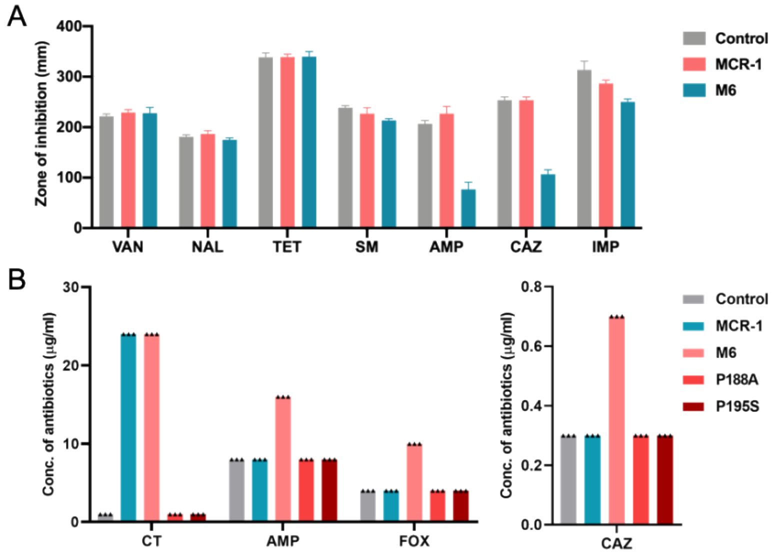

Figure S3. Verification of the β-lactam antibiotic co-resistance rendered by M6.

(A) Zone of inhibition of *E. coli* BW25113 harbouring empty plasmid, MCR-1 or M6 generated by disk diffusion method.

(B) Different levels of antibiotic sensitivity in *E. coli* expressing either wild-type MCR-1, M6 or its single point mutants.

The raw data underlying this Figure can be found in S1_data.
