# Supplementary material for: A new variant of the colistin resistance gene MCR-1 with co-resistance to β-lactam antibiotics reveals a potential novel antimicrobial peptide
Source: PLoS Biol. 2023 Dec 13;21(12):e3002433. doi: 10.1371/journal.pbio.3002433 (PMC10786390; doi:10.1371/journal.pbio.3002433)
Supplement: S4 Fig — pACYCDuet-1 carrying MCR-1 or M6 under the regulation of the MCR-1 native promoter (NP MCR-1 or NP M6) was generated. Overnight cultures were subcultured into fresh LB broth at a ratio of 1:100. For the gene under the regulation of arabinose promoter, addition of 0.2% arabinose was required for protein expression induction. Logarithmic phase cultures were collected for following assays. (A) The sensitivity of indicated strains towards CT and β-lactam antibiotics (AMP, FOX, and CAZ) were evaluated by agar dilution MIC tests. Each triangle represents an independent experiment. The experiments were performed 3 times with similar results. NP, native promoter. (B) Efficiency of plating assays on LB agar plates containing 0.1% SDS and 1 mM EDTA. Ten-fold serial dilution of indicated cultures was inoculated onto the agar plates. (C) The OM integrity of indicated strains was determined by measuring NPN uptake. And the fluorescent signal for each sample was monitored with a microplate reader at an excitation wavelength of 350 nm and emission wavelength of 420 nm after staining. (D, E) The IM permeability of indicated strains was evaluated by PI staining assay. Overnight cultures were subcultured into fresh LB broth at a ratio of 1:100. After cultivation for 8 h, cultures were collected, respectively, followed by staining with PI dye for 15 min. The PI-positive proportion was determined by flow cytometry and analyzed by FlowJo version 10 software. All the above-described experiments were performed thrice with similar results. Error bars indicate standard errors of the means (SEMs) for 3 biological replicates. A two-tailed unpaired t test was performed to determine the statistical significance of the data. ns, no significant difference; **, P < 0.01; ***, P < 0.001. The raw data underlying this figure can be found in S1 Data. (PDF) [file pbio.3002433.s005.pdf]

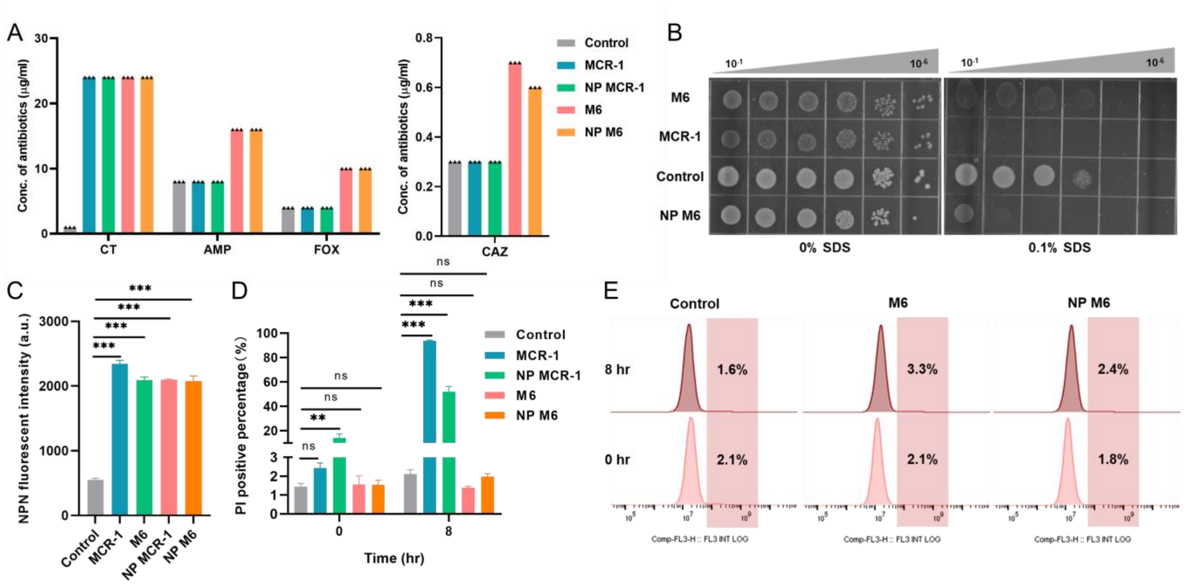

Figure S4. Co-resistance and membrane perturbation induced by native promoter-M6.

pACYCDuet-1 carrying MCR-1 or M6 under the regulation of the MCR-1 native promoter (NP MCR-1 or NP M6) was generated. Overnight cultures were sub-cultured into fresh LB broth at a ratio of 1:100. For the gene under the regulation of arabinose promoter, addition of 0.2% arabinose was required for protein expression induction. Logarithmic phase cultures were collected for following assays.

(**A**) The sensitivity of indicated strains towards CT and β-lactam antibiotics (AMP, FOX and CAZ) were evaluated by agar dilution MIC tests. Each triangle represents an independent experiment. The experiments were performed three times with similar results. NP, native promoter.

(B) Efficiency of plating assays on LB agar plates containing 0.1% SDS and 1 mM EDTA. Ten-fold serial-dilution of indicated cultures were inoculated onto the agar plates.

(C) The outer membrane integrity of indicated strains were determined by measuring NPN uptake. And the fluorescent signal for each sample was monitored with a microplate reader at an excitation wavelength of 350 nm and emission wavelength of 420 nm after staining.

(D-E) The inner membrane permeability of indicated strains were evaluated by PI staining assay. Overnight cultures were sub-cultured into fresh LB broth at a ratio of 1:100. After cultivation for 8 hr, cultures were collected, respectively, followed by staining with PI dye for 15 min. The PI-positive proportion was determined by flow cytometry and analysed by FlowJo version 10 software.

All the above-described experiments were performed thrice with similar results. Error bars indicate standard errors of the means (SEMs) for three biological replicates. A two-tailed unpaired *t* test was performed to determine the statistical significance of the data. ns, no significant difference; **, $P< 0.01$; ***, $P< 0.001$. The raw data underlying this Figure can be found in S1_data.
