# Supplementary material for: A new variant of the colistin resistance gene MCR-1 with co-resistance to β-lactam antibiotics reveals a potential novel antimicrobial peptide
Source: PLoS Biol. 2023 Dec 13;21(12):e3002433. doi: 10.1371/journal.pbio.3002433 (PMC10786390; doi:10.1371/journal.pbio.3002433)
Supplement: S5 Fig — (A) Representative periplasmic GFP and cytoplasmic mCherry images of E. coli BW25113 cells expressing MCR-1 or M6 during stationary stage. Shrinkage of the cytoplasm was evident by the bright periplasmic GFP signal. (B) The percentage of cells exhibiting shrinkage at pole(s) was calculated. The graph was visualized with Prism 9 software. All the above-described experiments were performed 3 times with similar results. Error bars indicate standard errors of the means (SEMs) for 3 biological replicates. A two-tailed unpaired t test was performed to determine the statistical significance of the data. **, P < 0.01; ***, P < 0.001. The raw data underlying this figure can be found in S1 Data. (PDF) [file pbio.3002433.s006.pdf]

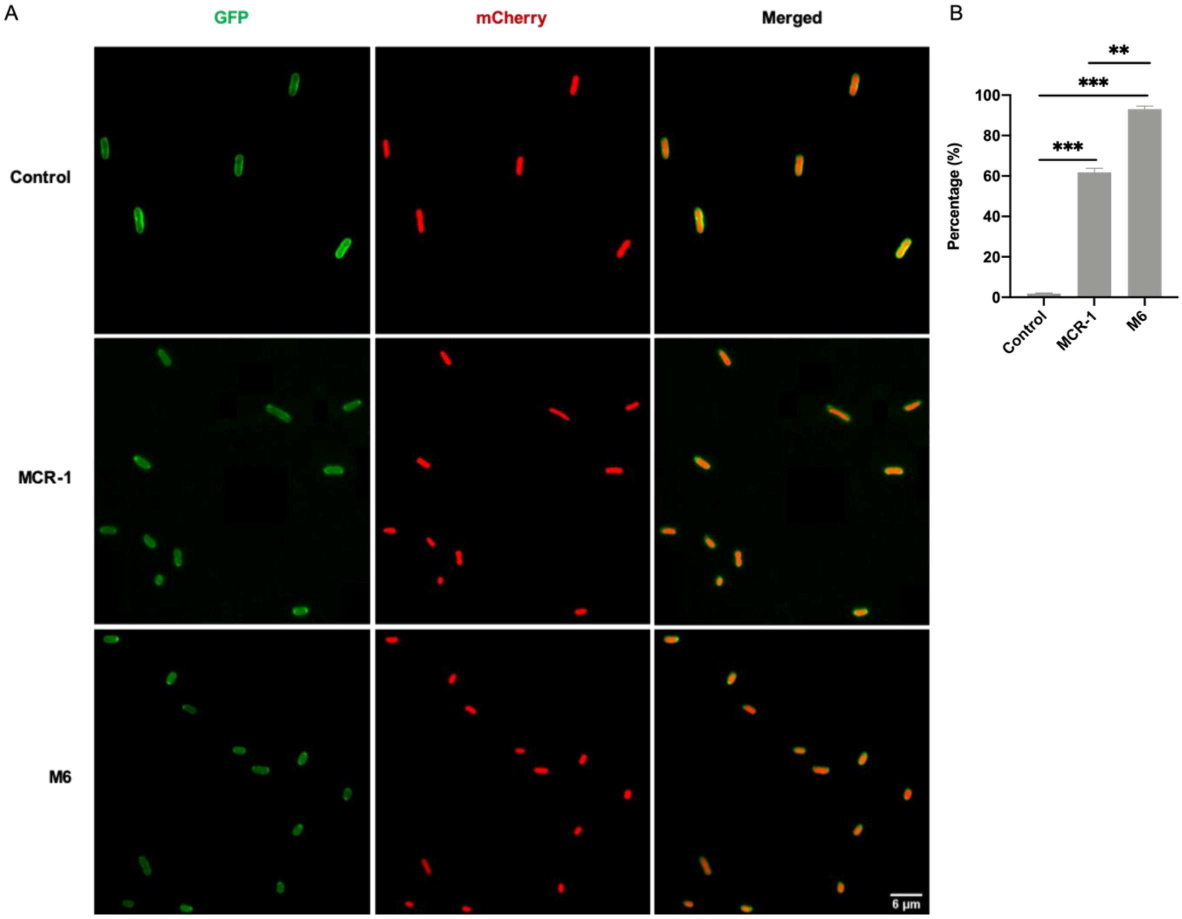

Figure S5. Expression of M6 inducing *E. coli* membrane shrinkage.

(A) Representative periplasmic GFP and cytoplasmic mCherry images of *E. coli* BW25113 cells expressing MCR-1 or M6 during stationary stage. Shrinkage of the cytoplasm was evident by the bright periplasmic GFP signal. **(B)** The percentage of cells exhibiting shrinkage at pole(s) were calculated. The graph was visualized with Prism 9 software.

All the above-described experiments were performed three times with similar results. Error bars indicate standard errors of the means (SEMs) for three biological replicates. A two-tailed unpaired *t* test was performed to determine the statistical significance of the data. **, $P< 0.01$; ***, $P< 0.001$. The raw data underlying this Figure can be found in S1_data.
