# Supplementary material for: A new variant of the colistin resistance gene MCR-1 with co-resistance to β-lactam antibiotics reveals a potential novel antimicrobial peptide
Source: PLoS Biol. 2023 Dec 13;21(12):e3002433. doi: 10.1371/journal.pbio.3002433 (PMC10786390; doi:10.1371/journal.pbio.3002433)
Supplement: S6 Fig — (A) The fluorescence ratio indicates the relative membrane voltage by using a genetically encoded voltage sensor Vibac2, which is a double-channel fusion protein that emits green and red fluorescence. The fluorescence intensity of GFP (Ig, Excitation = 488 nm, Emission = 512 nm) responds to membrane voltage, and the mCherry intensity (Ir, Excitation = 561 nm, Emission = 610) is used to normalize protein expression. Thus, the fluorescence ratio (Ig/Ir) indicates the relative membrane voltage in E. coli cells. To evaluate the impact of MCR-1 or M6 expression on the membrane voltage, a pBAD24 plasmid with the gene encoding Vibac2 was transformed into the indicated strains, and the fluorescence ratio (Ig/Ir) for each strain were measured and calculated during logarithmic phase. For each strain, 300 isolates were analyzed. And the representative GFP and mCherry images of BW25113 cells carrying empty plasmid, MCR-1 or M6 were represented as (B). The raw data underlying this figure can be found in S1 Data. (PDF) [file pbio.3002433.s007.pdf]

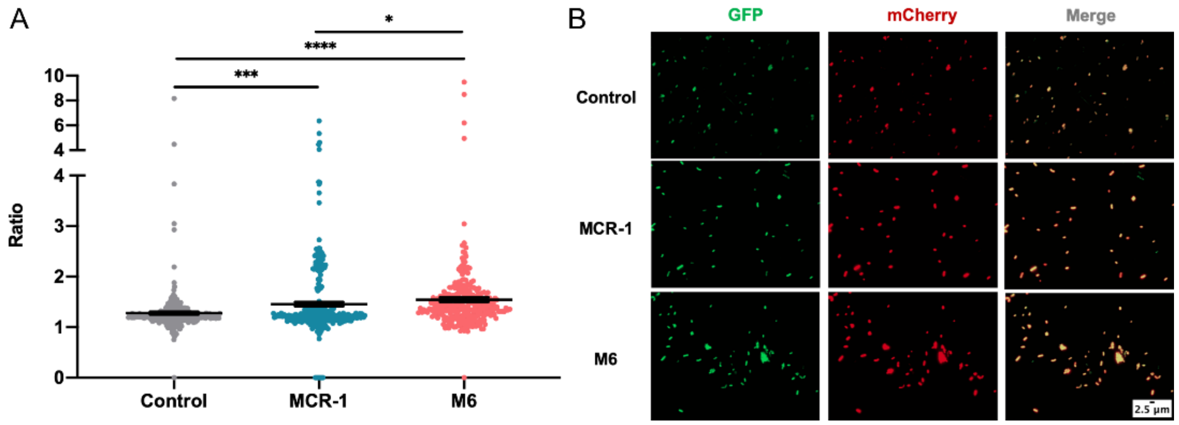

Figure S6. Membrane voltage measured by genetically-encoded sensor Vibac2.

(A) The fluorescence ratio indicates the relative membrane voltage by using a genetically-encoded voltage sensor Vibac2, which is a double-channel fusion protein that emits green and red fluorescence. The fluorescence intensity of GFP (Ig, Excitation = 488 nm, Emission = 512 nm) responds to membrane voltage, and the mCherry intensity (Ir, Excitation = 561 nm, Emission = 610) is used to normalize protein expression. Thus, the fluorescence ratio (Ig/Ir) indicates the relative membrane voltage in *E. coli* cells. To evaluation the impact of MCR-1 or M6 expression on the membrane voltage, a pBAD24 plasmid with the gene encoding Vibac2 was transformed into the indicated strains, and the fluorescence ratio (Ig/Ir) for each strain were measured and calculated during logarithmic phase. For each strain, 300 isolates were analysed. And the representative GFP and mCherry images of BW25113 cells carrying empty plasmid, MCR-1 or M6 were represented as **(B)**. The raw data underlying this Figure can be found in S1_data.
