# Supplementary material for: A new variant of the colistin resistance gene MCR-1 with co-resistance to β-lactam antibiotics reveals a potential novel antimicrobial peptide
Source: PLoS Biol. 2023 Dec 13;21(12):e3002433. doi: 10.1371/journal.pbio.3002433 (PMC10786390; doi:10.1371/journal.pbio.3002433)
Supplement: S7 Fig — (A) Venn diagram representing the differentially expressed proteins of E. coli BW25113 that harbored empty vector (control), MCR-1 and M6 during the exponential phase with the induction of 0.2% arabinose. (B) Top 20 pathways according to GO enrichment. The size and color of the points represent the number of target proteins and the q-values, respectively. The rich factor showed the enrichment degree in the GO pathway. (C) Visualization of the top KEGG enrichment pathway in M6-expressing cells compared with MCR-1-expressing cells. The up-regulated pathway is labeled in red, whereas the down-regulated pathway is labeled in blue. (D) Protein–protein interaction (PPI) analysis of the peptidoglycan layer remodeling pathway in M6 based on proteomics profiles, which were analyzed by STRING and visualized with Cytoscape software. Two biological replicates for each strain were used for the analysis. (PDF) [file pbio.3002433.s008.pdf]

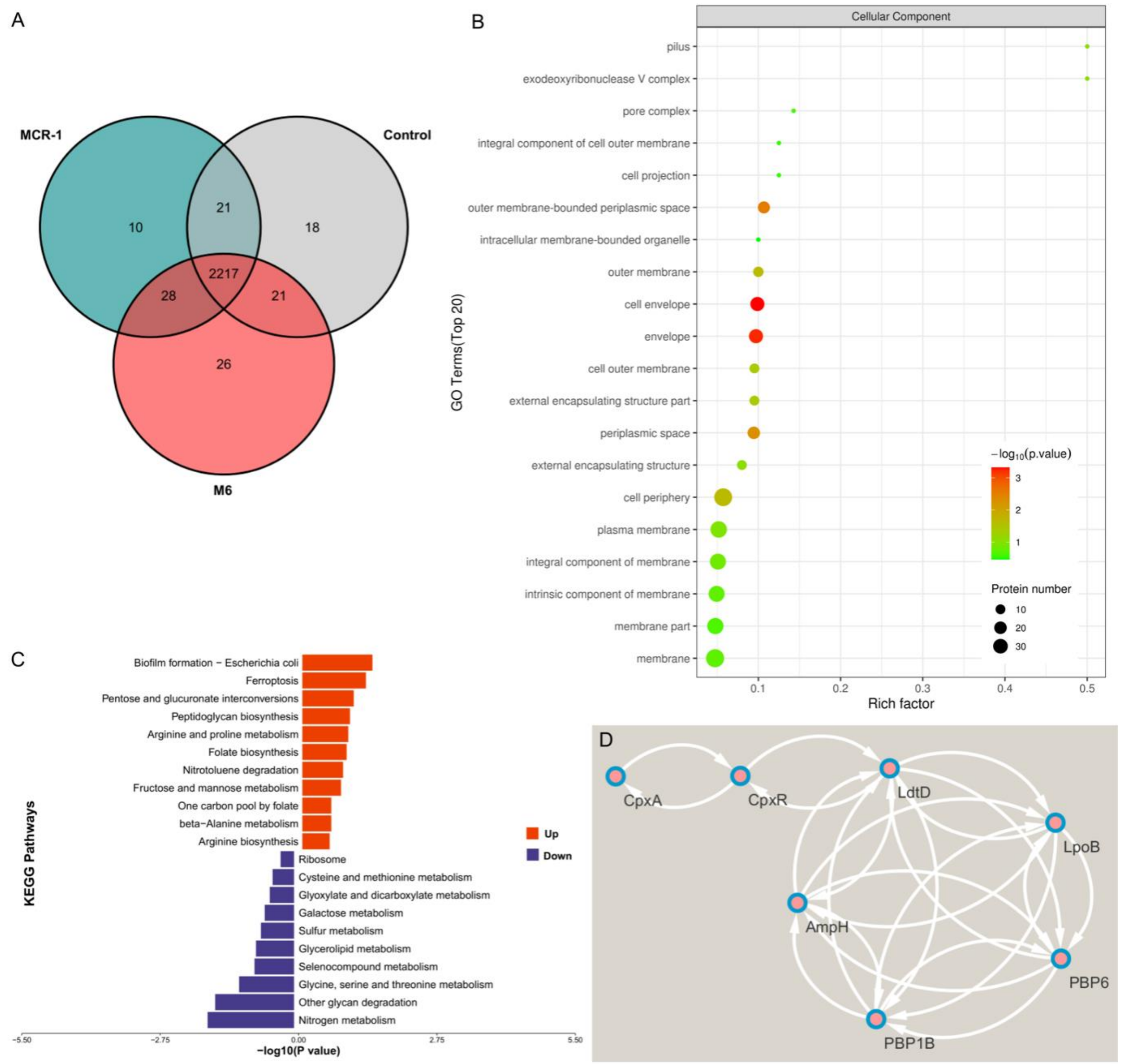

Figure S7. Comprehensive proteome profiles of M6 using label-free quantitative proteomics. **(A)** Venn diagram representing the differentially expressed proteins of *E. coli* BW25113 that harboured empty vector (control), MCR-1 and M6 during the exponential phase with the induction of 0.2% arabinose. **(B)** Top 20 pathways according to GO enrichment. The size and colour of the points represent the number of target proteins and the q-values, respectively. The rich factor showed the enrichment degree in the GO pathway. **(C)** Visualization of the top KEGG enrichment pathway in M6-expressing cells compared with MCR-1-expressing cells. The upregulated pathway is labelled in red, whereas the downregulated pathway is labelled in blue. **(D)** Protein–protein interaction (PPI) analysis of the peptidoglycan layer remodelling pathway in M6 based on proteomics profiles, which were analysed by STRING and visualized with Cytoscape software. Two biological replicates for each strain were used for the analysis.
