# Supplementary material for: A new variant of the colistin resistance gene MCR-1 with co-resistance to β-lactam antibiotics reveals a potential novel antimicrobial peptide
Source: PLoS Biol. 2023 Dec 13;21(12):e3002433. doi: 10.1371/journal.pbio.3002433 (PMC10786390; doi:10.1371/journal.pbio.3002433)
Supplement: S8 Fig — To test the role of LDTs in the M6-mediated phenotype, LdtD, PBP1B, or LpoB null strains carrying empty vector (control), WT MCR-1 or M6 were generated. (A) Antibiotic sensitivity of M6-expressing cells and MCR-1-expressing cells in the presence of copper. Overnight cultures of indicated strains were subcultured into fresh LB broth with or without 3.75 mM CuSO4 at a ratio of 1:100 and induced with 0.2% arabinose for 2 h. Next, the logarithmic-phase cultures were collected and adjusted to OD600 = 0.6, followed by spotting serial dilutions on LB agar plates containing CAZ or AMP, together with or without the addition of 3.75 mM CuSO4. MICs were determined after incubation at 37°C for 16 h. Each triangle represents an independent experiment. (B) Role of LDTs on β-lactam antibiotics susceptibility of M6. Overnight cultures of the indicated strains were subcultured into fresh LB broth at a ratio of 1:100 and induced with 0.2% arabinose for 2 h. The logarithmic-phase cultures were collected and adjusted to OD600 = 0.6, followed by spotting serial dilutions on LB agar plates with target antibiotics and incubation at 37°C for 16 h. Each triangle represents an independent experiment. The experiments were performed 3 times with the same results. (C) Efficiency of plating assays on LB agar plates containing 0.1% SDS and 1 mM EDTA or 0.001% SDS and 1 mM EDTA. Ten-fold serial dilution of indicated cultures was inoculated onto the agar plates. (D) mrcB deletion failed to reverse the OM permeability defect caused by M6. NPN uptake is represented by the background subtracted fluorescence at an excitation wavelength of 350 nm and emission wavelength of 420 nm. (E, F) mrcB deletion abolished M6-mediated IM integrity. The IM permeability was evaluated by PI staining assay. Overnight cultures were subcultured into fresh LB broth at a ratio of 1:100 and induced with 0.2% arabinose to express WT MCR-1 or M6. After induction for 4 h and 8 h, stationary and late-stationary phase cultures w [file pbio.3002433.s009.pdf]

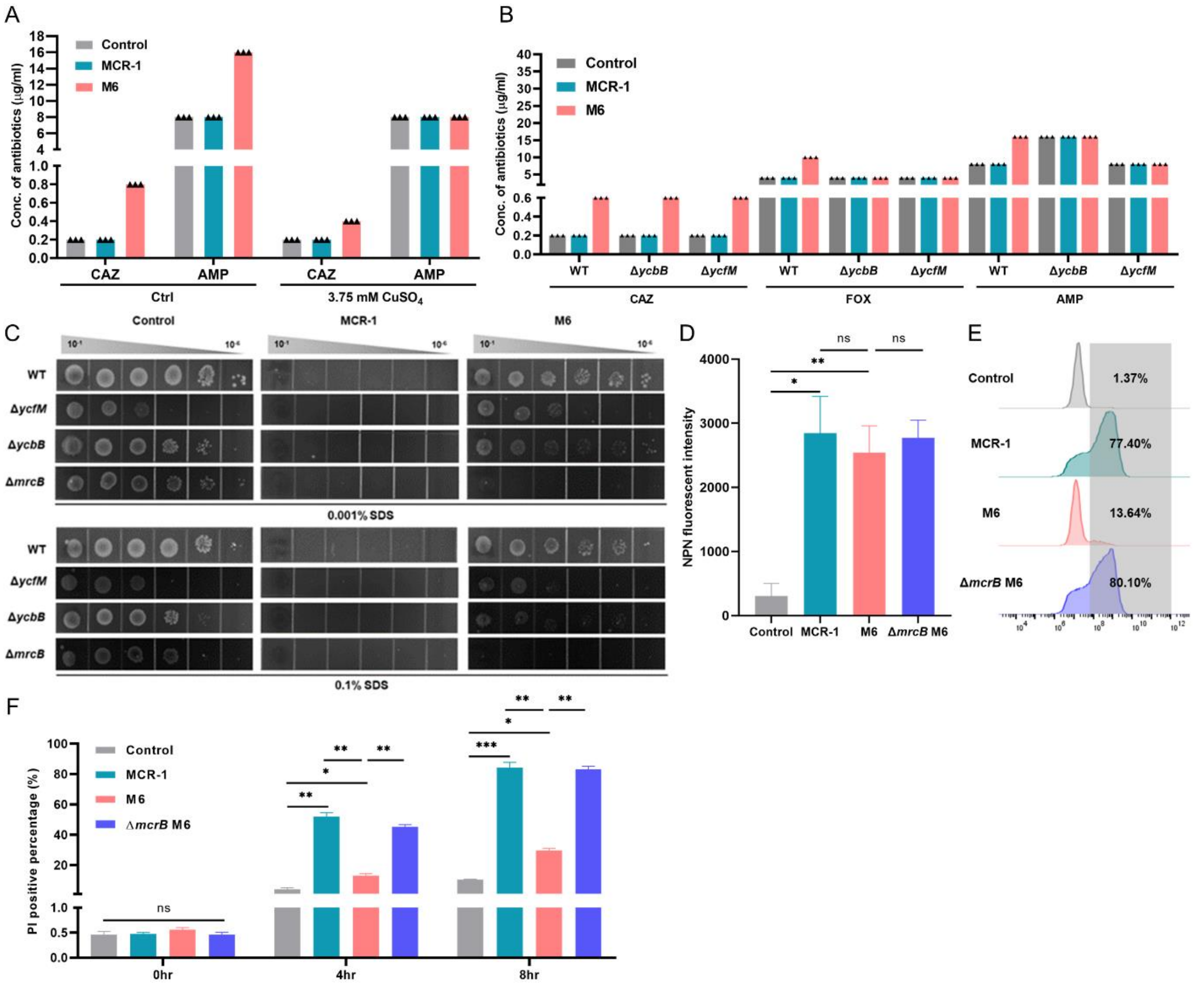

Figure S8. β-Lactams and SDS susceptibilities for LDTs-defective strains.

To test the role of LDTs in the M6-mediated phenotype, LdtD, PBP1B or LpoB null strains carrying empty vector (control), WT MCR-1 or M6 were generated.

(A)-Antibiotic sensitivity of M6-expressing cells and MCR-1-expressing cells in the presence of copper. Overnight cultures of indicated strains were sub-cultured into fresh LB broth with or without 3.75 mM $CuSO_4$ at a ratio of 1:100 and induced with 0.2% arabinose for 2 hr. Next, the logarithmic-phase cultures were collected and adjusted to OD_{600}=0.6, followed by spotting serial dilutions on LB agar plates containing CAZ or AMP, together with or without the addition of 3.75 mM $CuSO_4$. MICs were determined after incubation at 37 °C for 16 hr. Each triangle represents an independent experiment.

(B) Role of LDTs on β-lactam antibiotic susceptibility of M6. Overnight cultures of the indicated strains were sub-cultured into fresh LB broth at a ratio of 1:100 and induced with 0.2% arabinose for 2 hr. The logarithmic-phase cultures were collected and adjusted to OD_{600}=0.6, followed by spotting serial dilutions on LB agar plates with target antibiotics and incubation at 37 °C for 16 hr. Each triangle represents an independent experiment. The experiments were performed three times with the same results.

(C) Efficiency of plating assays on LB agar plates containing 0.1% SDS and 1 mM EDTA or 0.001% SDS and 1 mM EDTA. Ten-fold serial-dilution of indicated cultures were inoculated onto the agar plates.

(D) *mrcB* deletion failed to reverse the OM permeability defect caused by M6. NPN uptake is represented by the background subtracted fluorescence at an excitation wavelength of 350 nm and emission wavelength of 420 nm.

(E-F) *mrcB* deletion abolished M6-mediated inner membrane integrity. The inner membrane permeability was evaluated by PI staining assay. Overnight cultures were sub-cultured into fresh LB broth at a ratio of 1:100 and induced with 0.2% arabinose to express WT MCR-1 or M6. After induction for 4 hr and 8 hr, stationary and late-stationary phase cultures were collected, respectively, followed by staining with PI dye for 15 min. The PI-positive proportion was determined by flow cytometry and analysed by FlowJo version10 software. Representative results of three independent experiments are shown in **(E)**.

All the above-described experiments were performed three times with similar results. Error bars indicate standard errors of the means (SEMs) for three biological replicates. A two-tailed unpaired *t* test was performed to determine the statistical significance of the data. ns, no significant difference; *, $P< 0.1$; **, $P< 0.01$; ***, $P< 0.001$. The raw data underlying this Figure can be found in S1_data.
