# Supplementary material for: A new variant of the colistin resistance gene MCR-1 with co-resistance to β-lactam antibiotics reveals a potential novel antimicrobial peptide
Source: PLoS Biol. 2023 Dec 13;21(12):e3002433. doi: 10.1371/journal.pbio.3002433 (PMC10786390; doi:10.1371/journal.pbio.3002433)
Supplement: S9 Fig — (A) Morphology observation of spheroplasts for indicated strains. The shape of E. coli was changed from rob-shape into ball-shape, representing successful generation of spheroplasts. (B, C) The IM permeability of indicated spheroplasts was evaluated by PI staining assay. The well-prepared spheroplasts were incubated with PI dye for 15 min. The PI-positive proportion was determined by flow cytometry and analyzed by FlowJo version 10 software. All the above-described experiments were performed 3 times with similar results. Error bars indicate standard errors of the means (SEMs) for 3 biological replicates. A two-tailed unpaired t test was performed to determine the statistical significance of the data. ns, no significant difference; *, P < 0.1; ****, P < 0.0001. The raw data underlying this figure can be found in S1 Data. (PDF) [file pbio.3002433.s010.pdf]

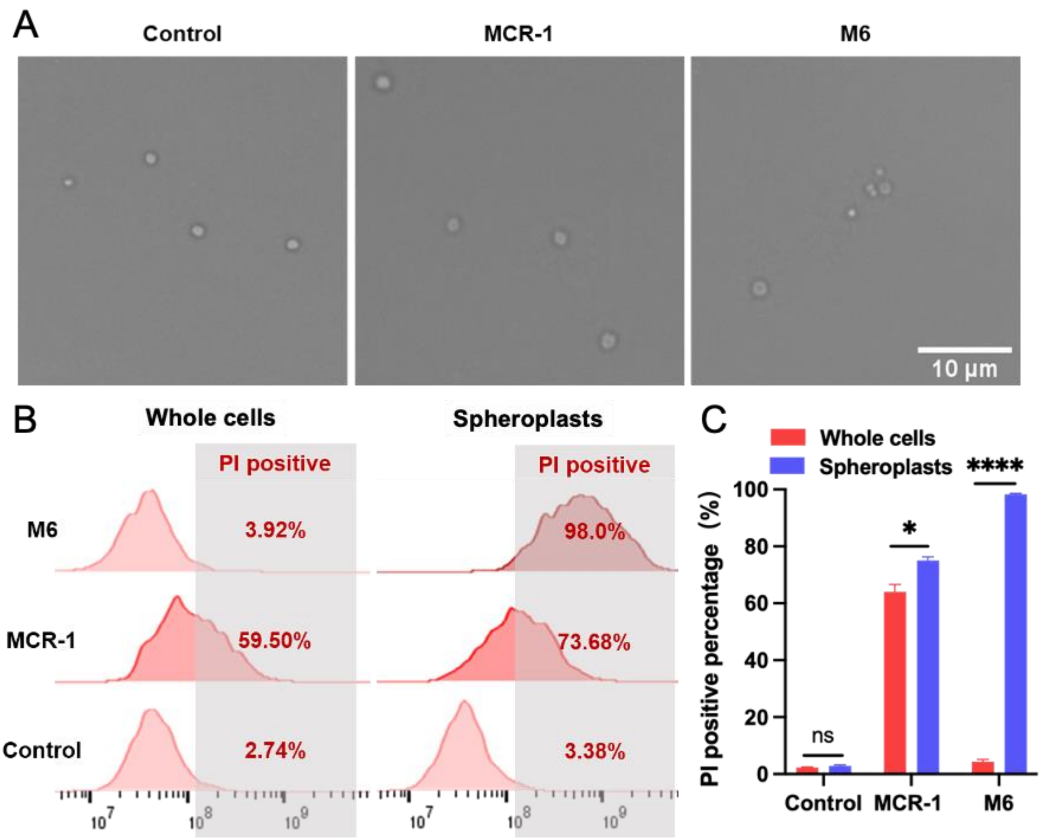

Figure S9. Spheroplasts LPS level perturbation of MCR-1 or M6 -bearing *E. coli*.

(A) Morphology observation of spheroplasts for indicated strains. The shape of *E. coli* was changed from rob-shape into ball-shape, representing successful generation of spheroplasts.

(B-C) The inner membrane permeability of indicated spheroplasts were evaluated by PI staining assay. The well-prepared spheroplasts were incubated with PI dye for 15 min. The PI-positive proportion was determined by flow cytometry and analysed by FlowJo version 10 software.

All the above-described experiments were performed three times with similar results. Error bars indicate standard errors of the means (SEMs) for three biological replicates. A two-tailed unpaired *t* test was performed to determine the statistical significance of the data. ns, no significant difference; *, $P< 0.1$; ****, $P< 0.0001$. The raw data underlying this Figure can be found in S1_data.
