# Supplementary material for: A new variant of the colistin resistance gene MCR-1 with co-resistance to β-lactam antibiotics reveals a potential novel antimicrobial peptide
Source: PLoS Biol. 2023 Dec 13;21(12):e3002433. doi: 10.1371/journal.pbio.3002433 (PMC10786390; doi:10.1371/journal.pbio.3002433)
Supplement: S10 Fig — The cellular LPS level in spheroplasts (A) or LpxC overexpressing strains (B) harboring empty plasmid, MCR-1 or M6 were determined by western blot. Error bars indicate standard errors of the means (SEM) for triple biological replicates. A two-tailed unpaired t test was performed to determine the statistical significance of the data. *, P < 0.1; **, P < 0.01; ***, P < 0.001. The bar graph was visualized with Prism 9 software. The raw data underlying this figure can be found in S1 Data. (PDF) [file pbio.3002433.s011.pdf]

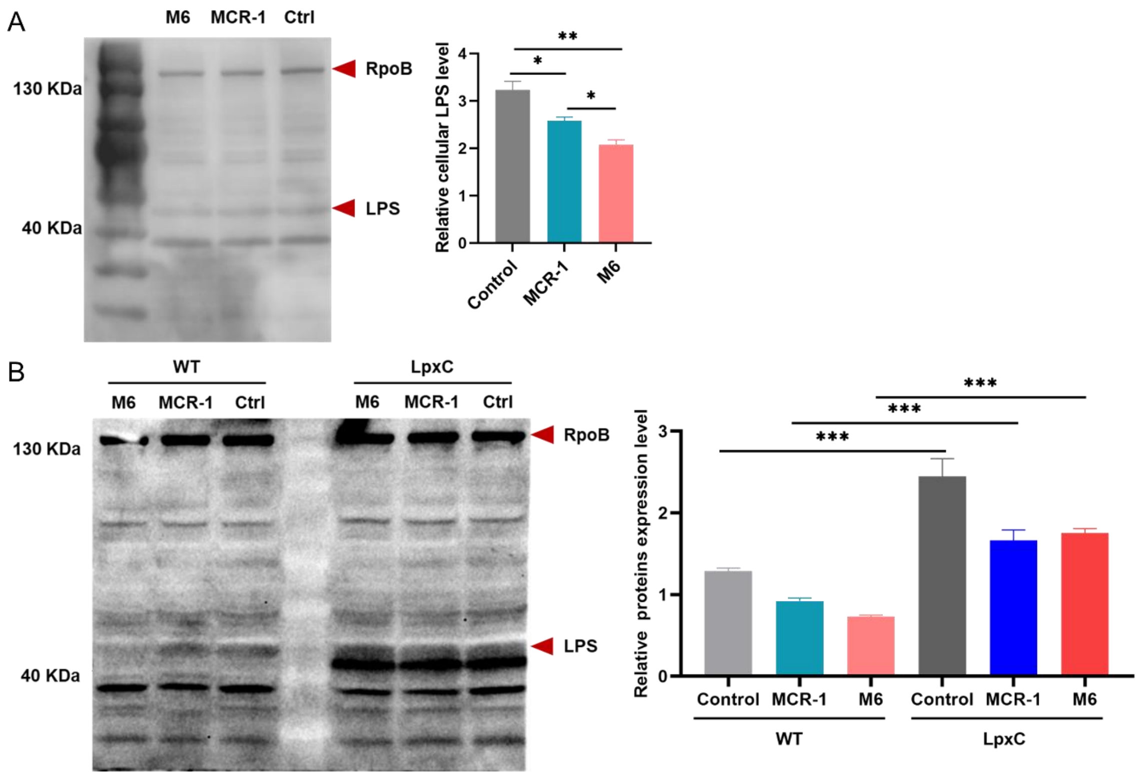

Figure S10. Cellular LPS level in spheroplasts and LpxC overexpressing strains.

The cellular LPS level in spheroplasts **(A)** or LpxC overexpressing strains **(B)** harbouring empty plasmid, MCR-1 or M6 were determined by western blot. Error bars indicate standard errors of the means (SEM) for triple biological replicates. A two-tailed unpaired *t* test was performed to determine the statistical significance of the data. *, $P< 0.1$; **, $P< 0.01$; ***, $P< 0.001$. The bar graph was visualized with Prism 9 software. The raw data underlying this Figure can be found in S1_data.
