# Supplementary material for: A new variant of the colistin resistance gene MCR-1 with co-resistance to β-lactam antibiotics reveals a potential novel antimicrobial peptide
Source: PLoS Biol. 2023 Dec 13;21(12):e3002433. doi: 10.1371/journal.pbio.3002433 (PMC10786390; doi:10.1371/journal.pbio.3002433)
Supplement: S11 Fig — (A) Deletion of the region that includes P188-P195. E. coli BW25113 carrying ΔP188-P195 was generated, and the susceptibility towards colistin (CT) and β-lactam antibiotics (AMP, FOX and CAZ) were evaluated by agar dilution MIC tests (B). The experiments were performed 3 times with the same results. (C) Efficiency of plating assays on LB agar plates containing 1% SDS and 1 mM EDTA or 0.001% SDS and 1 mM EDTA. Ten-fold serial dilution of indicated cultures was inoculated onto the agar plates. The raw data underlying this figure can be found in S1 Data. (PDF) [file pbio.3002433.s012.pdf]

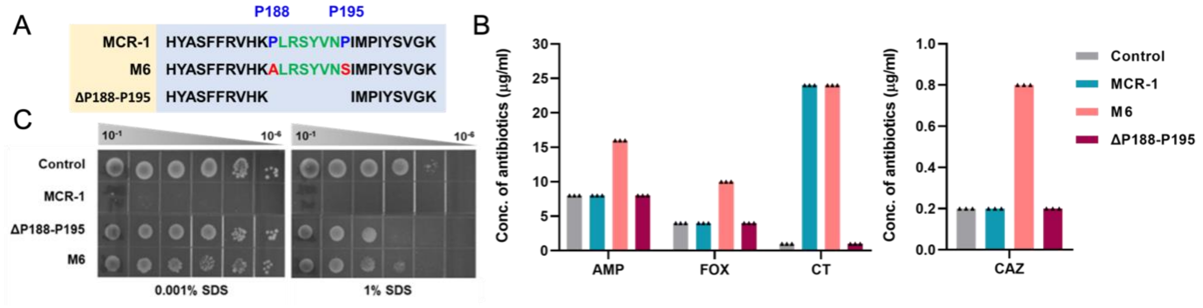

Figure S11. Necessity of P188-P195 segment for MCR-1 activity.

(A) Deletion of the region that includes P188-P195. *E. coli* BW25113 carrying ΔP188-P195 was generated, and the susceptibilities towards colistin (CT) and β-lactam antibiotics (AMP, FOX and CAZ) were evaluated by agar dilution MIC tests **(B)**. The experiments were performed three times with the same results.

(C) Efficiency of plating assays on LB agar plates containing 1% SDS and 1 mM EDTA or 0.001% SDS and 1 mM EDTA. Ten-fold serial-dilution of indicated cultures were inoculated onto the agar plates. The raw data underlying this Figure can be found in S1_data.
