# Supplementary material for: A new variant of the colistin resistance gene MCR-1 with co-resistance to β-lactam antibiotics reveals a potential novel antimicrobial peptide
Source: PLoS Biol. 2023 Dec 13;21(12):e3002433. doi: 10.1371/journal.pbio.3002433 (PMC10786390; doi:10.1371/journal.pbio.3002433)
Supplement: S12 Fig — The close-up view shows the MCR-1 lipid A binding cavity with LPS and the 4 regions responsible for anchoring LPS. The linker domain and transmembrane domain are in magenta and orange, respectively. LPS binding with MCR-1 is represented as a stick mode and colored in green. The salt bridges for the interaction with LPS are shown in red. The change in structure with mutated residues in each region is shown in detail. (PDF) [file pbio.3002433.s013.pdf]

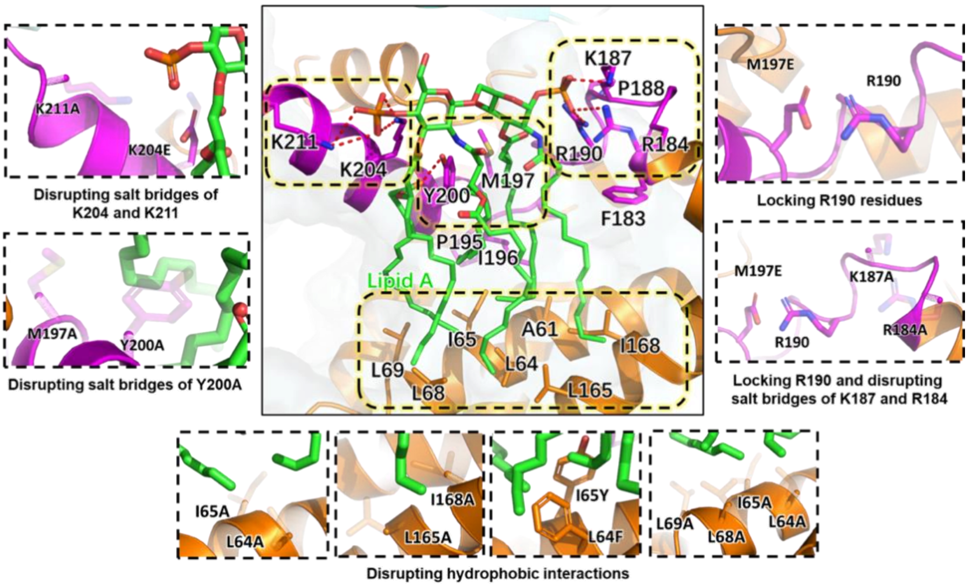

Figure S12. Mutations of essential regions for forming the lipid A binding cavity.

The close-up view shows the MCR-1 lipid A binding cavity with LPS and the four regions responsible for anchoring LPS. The linker domain and transmembrane domain are in magenta and orange, respectively. LPS binding with MCR-1 is represented as a stick mode and coloured in green. The salt bridges for the interaction with LPS are shown in red. The change in structure with mutated residues in each region is shown in detail.
