# Supplementary material for: A new variant of the colistin resistance gene MCR-1 with co-resistance to β-lactam antibiotics reveals a potential novel antimicrobial peptide
Source: PLoS Biol. 2023 Dec 13;21(12):e3002433. doi: 10.1371/journal.pbio.3002433 (PMC10786390; doi:10.1371/journal.pbio.3002433)
Supplement: S13 Fig — (A) Represents the RMSD fluctuations for WT and M6 of MCR1-lipid A. The variations in MCR-1 MM-GBSA binding energy with WT and M6 during the MD trajectories are shown in (B). (C, D) The statistics for the observed molecular interactions between lipid A and MCR-1 over the simulated trajectories of WT and the M6 mutant (the last 200-ns trajectories used for binding free energy calculations). (E) Quantification of lipid A with or without the modification of phosphoethanolamine (PEA). The bar graph represented the abundance of modified lipid A and unmodified lipid A extracted from the whole cells and spheroplasts of E. coli BW25113 expressing MCR-1 or M6, which was determined by MALDI-TOF. The experiments were performed 3 times with similar results. The raw data underlying this figure can be found in S1 Data. (PDF) [file pbio.3002433.s014.pdf]

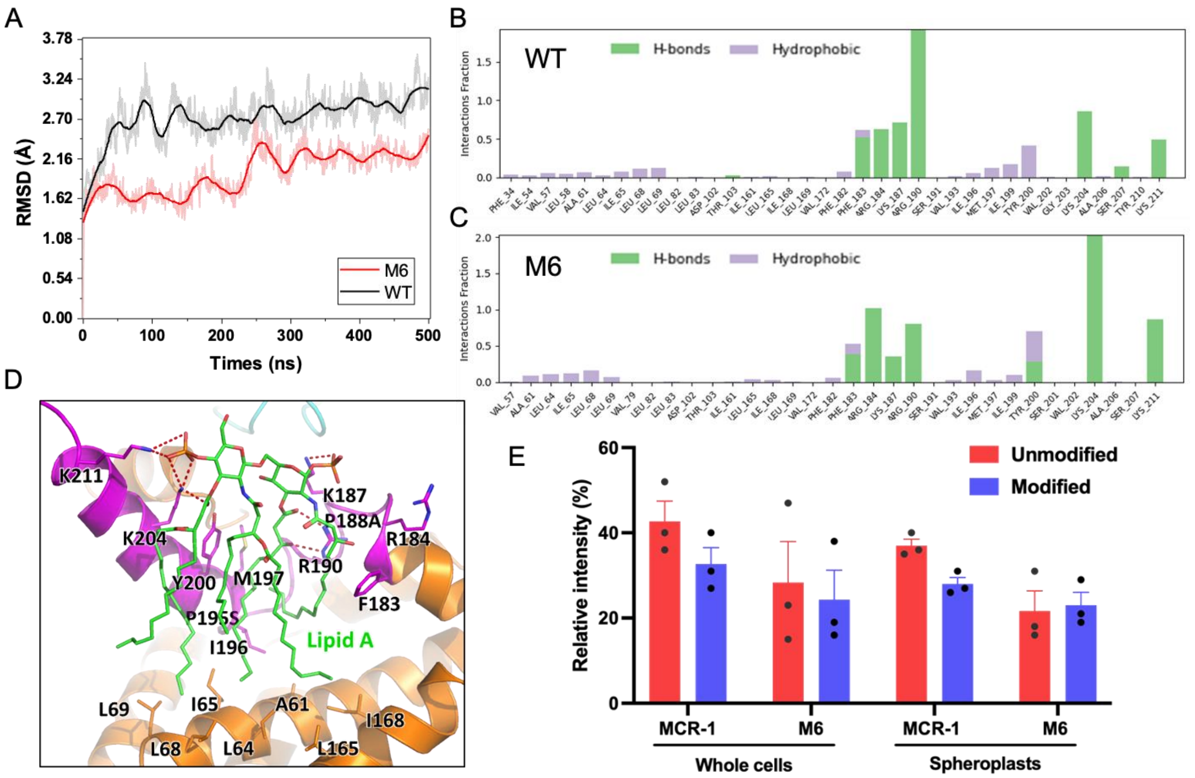

Figure S13. Impact of P188A+P195S mutations upon lipid A binding pocket.

(A) represents the RMSD fluctuations for WT and M6 of MCR1-lipid A. The variations in MCR-1 MM-GBSA binding energy with WT and M6 during the MD trajectories are shown in **(B).**

(C-D) The statistics for the observed molecular interactions between lipid A and MCR-1 over the simulated trajectories of WT and the M6 mutant (the last 200-ns trajectories used for binding free energy calculations).

(E) Quantification of lipid A with or without the modification of phosphoethanolamine (PEA). The bar graph represented the abundance of modified lipid A and unmodified lipid A extracted from the whole cells and spheroplasts of *E. coli* BW25113 expressing MCR-1 or M6, which was determined by MALDI-TOF. The experiments were performed three times with similar results. The raw data underlying this Figure can be found in S1_data.
