# Supplementary material for: A new variant of the colistin resistance gene MCR-1 with co-resistance to β-lactam antibiotics reveals a potential novel antimicrobial peptide
Source: PLoS Biol. 2023 Dec 13;21(12):e3002433. doi: 10.1371/journal.pbio.3002433 (PMC10786390; doi:10.1371/journal.pbio.3002433)
Supplement: S14 Fig — Logarithmic phase culture of E. coli ATCC 25922 was collected and stained by FM4-64 (red) and DAPI (cyan) to indicate the localization of bacterial membrane as well as cytoplasmic chromosome. The well-stained culture was subsequently treated with FITC-labeled 24AA-2M and 19AA-2M-tag in the concentrations of 0.05× and 0.1× MICs (18.5 μM and 37.0 μM), respectively. After washing with 1× PBS, the localization of the FITC signal were determined by observation with fluorescent microscope. (PDF) [file pbio.3002433.s015.pdf]

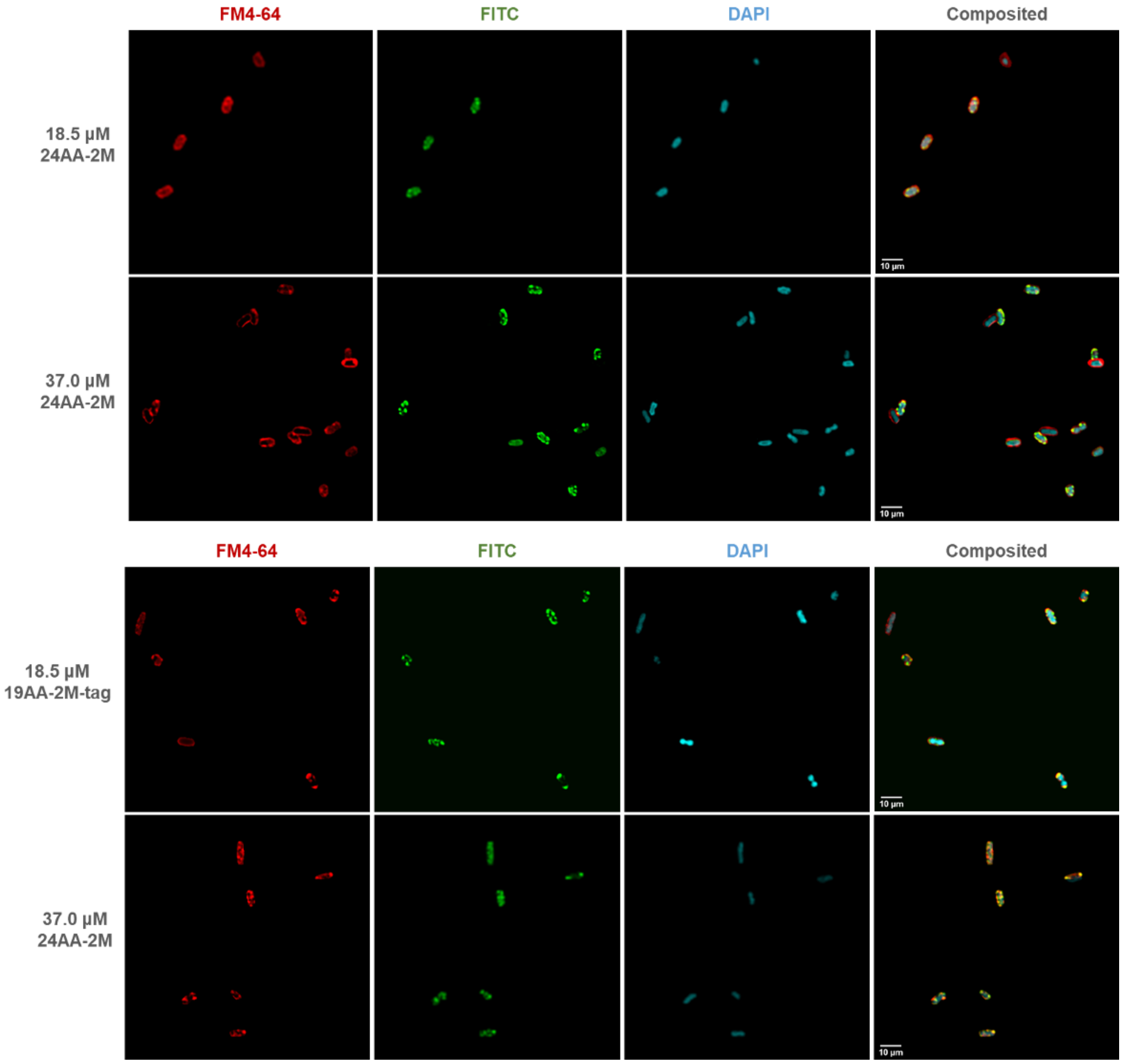

Figure S14. Localization of FITC-labelled peptides in *E. coli* ATCC 25922.

Logarithmic phase culture of *E. coli* ATCC 25922 was collected, and stained by FM4-64 (red) and DAPI (cyan) to indicate the localization of bacterial membrane as well as cytoplasmic chromosome. The well-stained culture was subsequently treated with FITC-labelled 24AA-2M and 19AA-2M-tag in the concentrations of 0.05× and 0.1× MICs (18.5 µM and 37.0 µM), respectively. After washing with 1× PBS, the localization of the FITC signal were determined by observation with fluorescent microscope.
