# Supplementary material for: A new variant of the colistin resistance gene MCR-1 with co-resistance to β-lactam antibiotics reveals a potential novel antimicrobial peptide
Source: PLoS Biol. 2023 Dec 13;21(12):e3002433. doi: 10.1371/journal.pbio.3002433 (PMC10786390; doi:10.1371/journal.pbio.3002433)
Supplement: S15 Fig — To identify the cytotoxicity of 24AA-2M and 19AA-2M-tag, the cell permeabilizing effects of the indicated peptides on mouse blood cells were determined by an LDH-based TOX-7 kit (Sigma). Fresh healthy mouse blood was treated with the above peptides in the concentrations of 46.25, 92.5, 185, or 370 μM. LDH activity was evaluated to determine LDH released from mousse cells. Error bars indicate standard errors of the means (SEMs) for 3 biological replicates. A two-tailed unpaired t test was performed to determine the statistical significance of the data. ns, no significant difference; *, P < 0.1; **, P < 0.01. The bar graph was visualized with Prism 9 software. N.D., not detected. The raw data underlying this figure can be found in S1 Data. (PDF) [file pbio.3002433.s016.pdf]

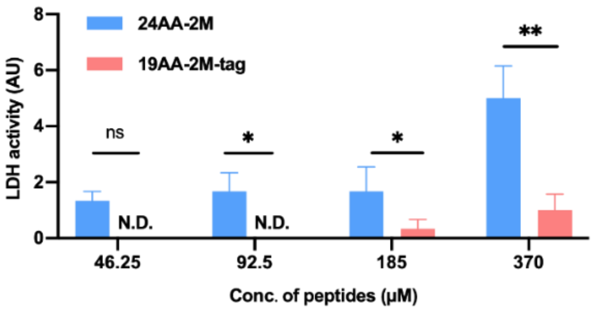

Figure S15. Permeabilization of synthetic peptides on mouse blood cells.

To identify the cytotoxicity of 24AA-2M and 19AA-2M-tag, the cell permeabilizing effects of the indicated peptides on mouse blood cells were determined by an LDH-based TOX-7 kit (Sigma). Fresh healthy mouse blood was treated with the above peptides in the concentrations of 46.25, 92.5, 185 or 370 μM. LDH activity was evaluated to determine LDH released from mousse cells. Error bars indicate standard errors of the means (SEMs) for three biological replicates. A two-tailed unpaired *t* test was performed to determine the statistical significance of the data. ns, no significant difference; *, $P< 0.1$; **, $P< 0.01$. The bar graph was visualized with Prism 9 software. N.D., not detected. The raw data underlying this Figure can be found in S1_data.
