# Supplementary material for: A new variant of the colistin resistance gene MCR-1 with co-resistance to β-lactam antibiotics reveals a potential novel antimicrobial peptide
Source: PLoS Biol. 2023 Dec 13;21(12):e3002433. doi: 10.1371/journal.pbio.3002433 (PMC10786390; doi:10.1371/journal.pbio.3002433)
Supplement: S16 Fig — By treating E. coli ATCC 25922, P. aeruginosa ATCC 27853, S. Typhimurium SL1344, K. pneumonic ATCC 13883, and A. baumanii ATCC 19606 with 19AA-2M-tag in a series of concentrations, dose-response curves were generated. The in vitro inhibitory rate and IC50 value of various strains treated with 19AA-2M-tag are presented. The raw data underlying this figure can be found in S1 Data. (PDF) [file pbio.3002433.s017.pdf]

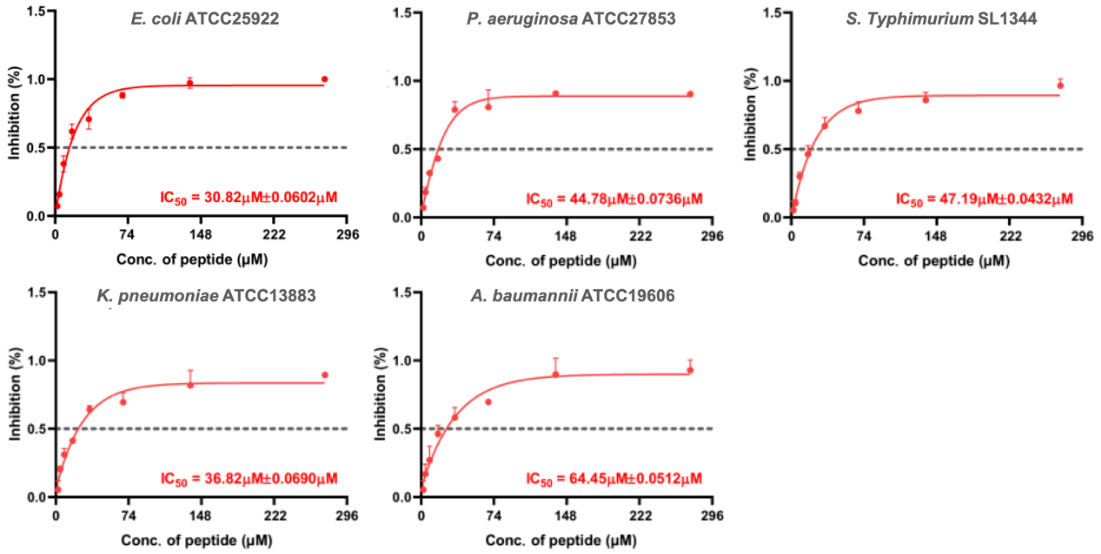

Figure S16. Inhibitory rate of 19AA-2M-tag among Gram-negative strains.

By treating *E. coli* ATCC 25922, *P. aeruginosa* ATCC 27853, *S. Typhimurium* SL1344, *K. pneumonic* ATCC 13883, and *A. baumanii* ATCC 19606 with 19AA-2M-tag in a series of concentrations, dose-response curves were generated. The *in vitro* inhibitory rate and IC_{50} value of various strains treated with 19AA-2M-tag are presented. The raw data underlying this Figure can be found in S1_data.
