# Supplementary material for: A new variant of the colistin resistance gene MCR-1 with co-resistance to β-lactam antibiotics reveals a potential novel antimicrobial peptide
Source: PLoS Biol. 2023 Dec 13;21(12):e3002433. doi: 10.1371/journal.pbio.3002433 (PMC10786390; doi:10.1371/journal.pbio.3002433)
Supplement: S17 Fig — (A) E. coli ATCC 25922 and a carbapenem-resistant E. coli isolated in the clinic were used to test the efficacy of a drug combination consisting of 19AA-2M-tag and colistin through a checkerboard assay. A total of 1 × 10^3^ CFUs of the indicated strains were inoculated at initiation (T0), followed by treatment with drug combinations in a series of concentrations at 37°C for 16 h (Tn). The OD600 was measured before and after the treatment. The graphs show the value of OD600 Tn minus the OD600 T0 of each well for both strains. A color gradient heat map, with hot (red: OD600 = 0) to cool (blue: OD600 = 0.5) colors, indicates low to high values. The synergetic effect was further determined by calculating the FICI. (B) Shows the inhibition curves of colistin with (red) or without (blue) the addition of 19AA-2M-tag. The dose-response curves of E. coli ATCC 25922 were generated by measuring the inhibitory rate after treatment with colistin in a series of concentrations, with or without co-treatment with 92.5 μM 19AA-2M-tag. The raw data underlying this figure can be found in S1 Data. (PDF) [file pbio.3002433.s018.pdf]

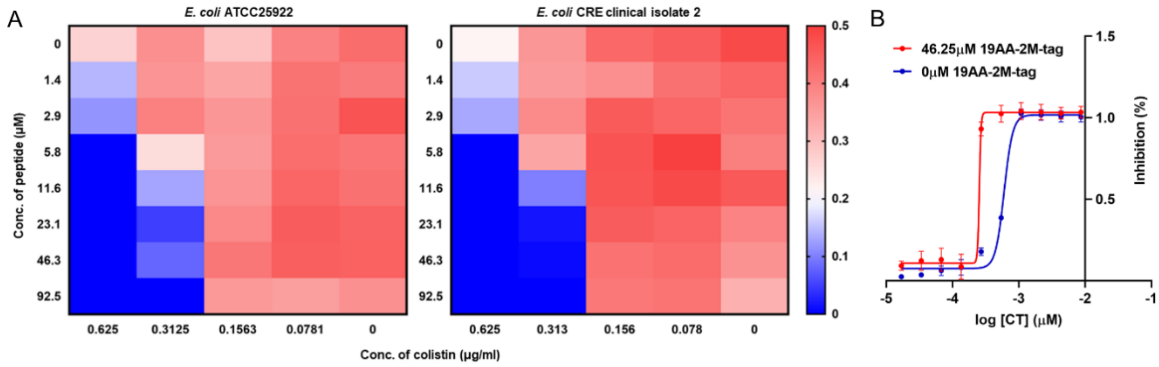

Figure S17. Synergistic inhibition of *E. coli* growth by 19AA-2M-tag and colistin. **(A)** *E. coli* ATCC 25922 and a carbapenem-resistant *E. coli* isolated in the clinic were used to test the efficacy of a drug combination consisting of 19AA-2M-tag and colistin through a checkerboard assay. A total of 1×10^3 CFUs of the indicated strains were inoculated at initiation (T0), followed by treatment with drug combinations in a series of concentrations at 37 °C for 16 hr (Tn). The OD_{600} was measured before and after the treatment. The graphs show the value of $OD_{600\ T0}$ minus the $OD_{600\ Tn}$ of each well for both strains. A colour gradient heat map, with hot (red: OD_{600} = 0) to cool (blue: OD_{600} = 0.5) colours, indicates low to high values. The synergetic effect was further determined by calculating the FICI. **(B)** shows the inhibition curves of colistin with (red) or without (blue) the addition of 19AA-2M-tag. The dose–response curves of *E. coli* ATCC 25922 were generated by measuring the inhibitory rate after treatment with colistin in a series of concentrations, with or without co-treatment with 92.5 µM 19AA-2M-tag. The raw data underlying this Figure can be found in S1_data.
