# Supplementary material for: A new variant of the colistin resistance gene MCR-1 with co-resistance to β-lactam antibiotics reveals a potential novel antimicrobial peptide
Source: PLoS Biol. 2023 Dec 13;21(12):e3002433. doi: 10.1371/journal.pbio.3002433 (PMC10786390; doi:10.1371/journal.pbio.3002433)
Supplement: S18 Fig — LPS modification mediated by MCR-1 might be guaranteed by a two-step process, which requires the LPS substrate to be loaded into binding pocket and transferred to the catalytic domain. During the first-half reaction, the Apo state MCR-1 interacts with the PE donor at the outer leaflet of the IM and accepts a PEA group in the catalytic domain, transforming into the active state to interact with LPS. Next, LPS binds with the MCR-1 loading cavity at the linker domain, which is inserted into the lipid bilayer, followed by transfer to the catalytic domain exposed at the interface between the IM and periplasm. During the second-half reaction, the PEA group at the catalytic domain was added to the 1′- or 4′-phosphate group of LPS to form PEA-LPS, which was then released back into the IM, and MCR-1 switched to the Apo state again. (PDF) [file pbio.3002433.s019.pdf]

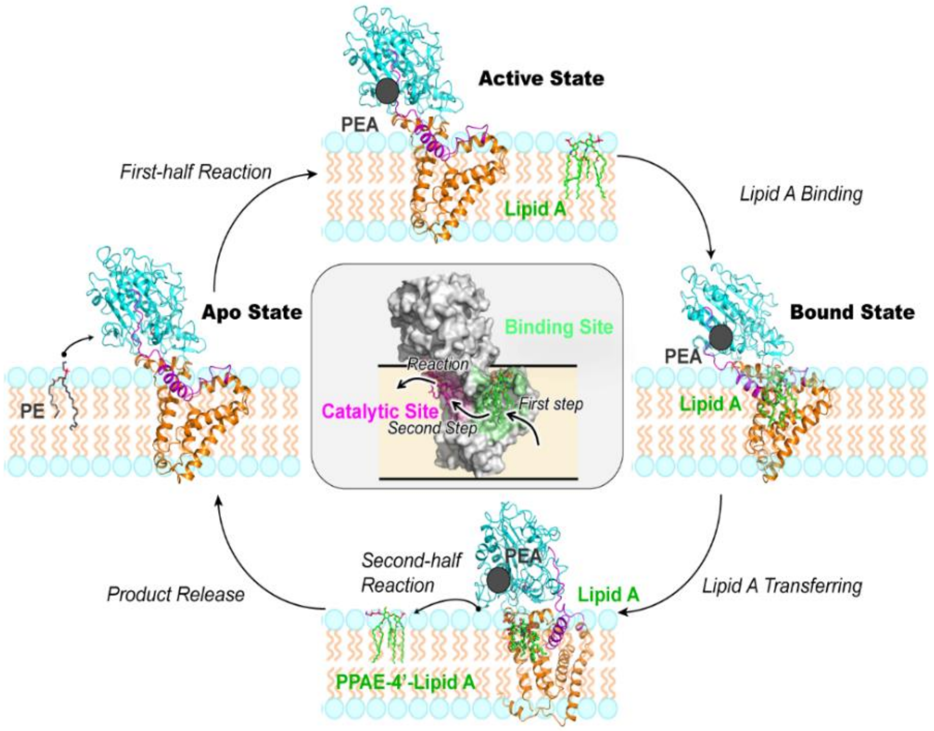

Figure S18. The "loading-transferring" mode of MCR-1.

LPS modification mediated by MCR-1 might be guaranteed by a two-step process, which requires the LPS substrate to be loaded into binding pocket and transferred to the catalytic domain. During the first-half reaction, the Apo state MCR-1 interacts with the PE donor at the outer leaflet of the inner membrane and accepts a PEA group in the catalytic domain, transforming into the active state to interact with LPS. Next, LPS binds with the MCR-1 loading cavity at the linker domain, which is inserted into the lipid bilayer, followed by transfer to the catalytic domain exposed at the interface between the inner membrane and periplasm. During the second-half reaction, the PEA group at the catalytic domain was added to the 1'- or 4'-phosphate group of LPS to form PEA-LPS, which was then released back into the inner membrane, and MCR-1 switched to the Apo state again.
