# Supplementary material for: A new variant of the colistin resistance gene MCR-1 with co-resistance to β-lactam antibiotics reveals a potential novel antimicrobial peptide
Source: PLoS Biol. 2023 Dec 13;21(12):e3002433. doi: 10.1371/journal.pbio.3002433 (PMC10786390; doi:10.1371/journal.pbio.3002433)
Supplement: S19 Fig — The plasmid for expressing MCR-1 or M6 under the regulation of arabinose promoter was shown as (A), and the one for expressing target proteins under the regulation of MCR-1 native promoter was shown as (B). The elements in each plasmid were as followed: CmR = chloramphenicol acetyltransferase; p15A ori = the medium-copy-number p15A origin of replication; T7 terminator = transcription terminator for bacteriophage T7 RNA polymerase; araC = gene encoding L-arabinose regulatory protein; araBAD promoter = promoter of the L-arabinose operon; MCR-1 native promoter = the native promoter of mcr-1 gene amplified from clinically collected mcr-1+ strain; WT MCR-1/M6 = the gene encoding WT MCR-1 or M6. (PDF) [file pbio.3002433.s020.pdf]

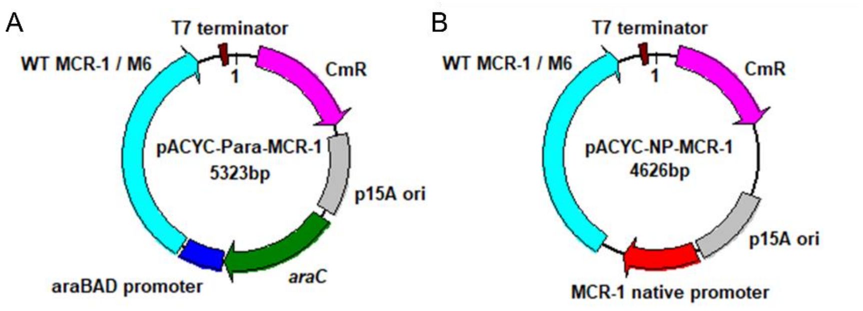

Figure S19. Plasmid maps of pACYC-Para-MCR-1 and pACYC-NP-MCR-1.

The plasmid for expressing MCR-1 or M6 under the regulation of arabinose promoter was shown as **(A)**, and the one for expressing target proteins under the regulation of MCR-1 native promoter was shown as **(B)**. The elements in each plasmid were as followed: CmR= chloramphenicol acetyltransferase; p15A *ori*= the medium-copy-number p15A origin of replication; T7 terminator= transcription terminator for bacteriophage T7 RNA polymerase; *araC*= gene encoding L-arabinose regulatory protein; *araBAD* promoter= promoter of the L-arabinose operon; MCR-1 native promoter= the native promoter of *mcr-1* gene amplified from clinically collected *mcr-1*$^{+}$ strain; WT MCR-1/M6= the gene encoding WT MCR-1 or M6.
