# Supplementary material for: A new variant of the colistin resistance gene MCR-1 with co-resistance to β-lactam antibiotics reveals a potential novel antimicrobial peptide
Source: PLoS Biol. 2023 Dec 13;21(12):e3002433. doi: 10.1371/journal.pbio.3002433 (PMC10786390; doi:10.1371/journal.pbio.3002433)
Supplement: S20 Fig — Molecular dynamic stimulation was utilized to discover the potential LPS binding cavity on the surface of MCR-1, and the workflow was designed as shown in (A). Structural comparison within lipid A (PDB code: 5IJD) and its analogues: Eritoran (PDB code: 2Z65) and Palmitoyllpid A (PDB code: 7BGL) are shown in (B). (C) The potential lipid A binding site on the surface of MCR-1 was discovered by SiteMap of Maestro. (D) Shows the RMSD fluctuations for multiple trajectories of MCR1-lipid A. (PDF) [file pbio.3002433.s021.pdf]

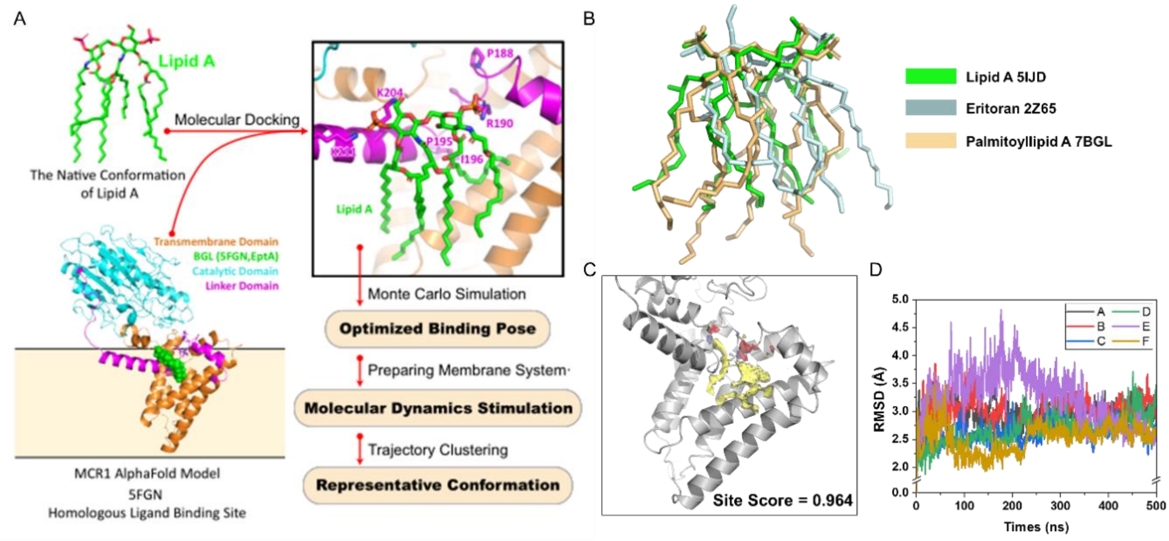

Figure S20. Analysis of the molecular dynamic simulation and binding energy. Molecular dynamic stimulation was utilized to discover the potential LPS binding cavity on the surface of MCR-1, and the workflow was designed as shown in **(A)**. Structural comparison within lipid A (PDB code: 5IJD) and its analogues: Eritoran (PDB code: 2Z65) and Palmitoyllpid A (PDB code: 7BGL) are shown in **(B)**. **(C)** The potential lipid A binding site on the surface of MCR-1 was discovered by SiteMap of Maestro. **(D)** shows the RMSD fluctuations for multiple trajectories of MCR1-lipid A.
