# Supplementary material for: A new variant of the colistin resistance gene MCR-1 with co-resistance to β-lactam antibiotics reveals a potential novel antimicrobial peptide
Source: PLoS Biol. 2023 Dec 13;21(12):e3002433. doi: 10.1371/journal.pbio.3002433 (PMC10786390; doi:10.1371/journal.pbio.3002433)
Supplement: S1 Table — (PDF) [file pbio.3002433.s022.pdf]

Supplementary Table 1. Concentrations of antibiotics used for screening the MCR-1 library.

Antibiotics	MIC			
	**0.8×**	**1×**	**1.5×**	**2×**
AMP	25.6	32	48	64
CAZ	0.4	0.5	0.75	1
IMP	0.2	0.25	0.375	0.5
SM	25.6	32	48	64
TET	6.4	8	12	16
NAL	6.4	8	12	16
VAN	204.8	256	384	512

Concentrations are in µg/ml.
