# Supplementary material for: A new variant of the colistin resistance gene MCR-1 with co-resistance to β-lactam antibiotics reveals a potential novel antimicrobial peptide
Source: PLoS Biol. 2023 Dec 13;21(12):e3002433. doi: 10.1371/journal.pbio.3002433 (PMC10786390; doi:10.1371/journal.pbio.3002433)
Supplement: S2 Table — (PDF) [file pbio.3002433.s023.pdf]

**Supplementary Table 2. Concentrations of β-lactam antibiotics used for screening the MCR-1 library.**

| Antibiotics | MIC | | | |
|---|---|---|---|---|
| | **0.8×** | **1×** | **1.5×** | **2×** |
| CTX | 0.4 | 0.5 | 0.75 | 1 |
| FEP | 1.6 | 2 | 3 | 4 |
| CRO | 0.8 | 1 | 1.5 | 2 |
| ETP | 0.1 | 0.125 | 0.1875 | 0.25 |
| MEM | 0.025 | 0.031 | 0.047 | 0.063 |
| FOX | 3.2 | 4 | 6 | 8 |

Concentrations are in µg/ml.
