# Supplementary material for: A new variant of the colistin resistance gene MCR-1 with co-resistance to β-lactam antibiotics reveals a potential novel antimicrobial peptide
Source: PLoS Biol. 2023 Dec 13;21(12):e3002433. doi: 10.1371/journal.pbio.3002433 (PMC10786390; doi:10.1371/journal.pbio.3002433)
Supplement: S3 Table — (PDF) [file pbio.3002433.s024.pdf]

Supplementary Table 3. Verification of MCR-1 isolates through Sanger sequencing.

No.	Mutations	MICs (μg/ml)		
		CAZ	AMP	FOX
1	V22D, A23D, A30T	0.2	8	4
2	S171A	0.2	8	4
3	P171L, A174V	0.4	16	4
4	L165P	0.4	16	8
5	WT	0.2	8	4
6	P188A, P195S	≥0.6	16	≥10
7	H186Q, P188T	0.4	16	4
8	P188R	0.4	8	4
9	A123E, R128L	0.2	8	4
10	WT	0.2	8	4
11	T53K	0.2	8	4
12	V512L, T514S, H515Y	0.2	8	4
13	P493L	0.2	8	4
14	D46V	0.2	8	4
15	WT	0.2	8	4
16	T558A, C562A	0.2	8	4
17	C563G	0.2	8	4
18	S177R	0.4	8	4
19	T29P	0.2	8	4
20	WT	0.2	8	4
21	A20S	0.2	8	4
22	V162G, V227E	0.2	8	4
23	V202G	0.2	8	4
24	F495C, F496F, D499G	0.2	8	4
25	WT	0.2	8	4
26	K212R, K500R, Q501R	0.2	8	4
27	Q265H	0.2	8	4
28	W8G	0.2	8	4
29	G87C, T96I, G98G	0.2	8	4
30	D366G, G368V	0.2	8	4
31	V19A, R157L, V533F	0.2	8	4
32	V19A, A342S, D346Y	0.2	8	4
33	WT	0.2	8	4
34	G368G (WT)	0.2	8	4
35	E418D, Q425R, A430D	0.2	8	4
36	WT	0.2	8	4
37	E418D, Q425R, A430D	0.2	8	4
