# Supplementary material for: A new variant of the colistin resistance gene MCR-1 with co-resistance to β-lactam antibiotics reveals a potential novel antimicrobial peptide
Source: PLoS Biol. 2023 Dec 13;21(12):e3002433. doi: 10.1371/journal.pbio.3002433 (PMC10786390; doi:10.1371/journal.pbio.3002433)
Supplement: S4 Table — (PDF) [file pbio.3002433.s025.pdf]

Table S4. The differentially expressed proteins between *E. coli* BW25113 carrying M6 and WT MCR-1

Protein_ID	Fc.value	P.value	gene
WP_000830741.1	8.87473	1.2E-48	*ampH*
WP_000471889.1	8.27719	2.2E-33	*mgtA*
WP_000190655.1	6.69928	4.7E-16	*yfhb*
WP_000732512.1	5.8143	5.8E-14	*rstB*
WP_001295285.1	4.90911	6.5E-08	*pmrD*
WP_000820410.1	4.89458	1.6E-19	*agn43*
WP_001092508.1	4.6609	2E-18	*rstA*
WP_001265471.1	4.043	9.1E-17	*phoP*
WP_001380339.1	3.81865	1.4E-15	*KXD89_14405*
WP_000705211.1	3.67125	1.4E-13	*ynfB*
WP_001273658.1	3.54056	1.8E-05	*ymdF*
WP_000433476.1	3.32089	7.1E-13	*maeA*
WP_000983871.1	3.30813	4.9E-05	*trpCF*
WP_000883034.1	3.22982	2.7E-11	*glsA*
WP_000597196.1	3.20858	3E-12	*slyB*
WP_000045315.1	3.19218	3.7E-12	*hemL*
WP_000066707.1	2.82914	9.2E-06	*fumB*
WP_000594599.1	2.80513	1.1E-05	*lpxT*
WP_000689228.1	2.79104	0.0005	*ytfK*
WP_000024560.1	2.76806	1.4E-05	*cbpM*
WP_000753946.1	2.74409	1.3E-09	*degP*
WP_001157540.1	2.62654	0.00107	*fetA*
WP_001138581.1	2.55387	1E-07	*speG*
WP_000953361.1	2.54374	0.00156	*nikA*
WP_000828648.1	2.51459	0.00179	*rmf*
WP_001300708.1	2.48639	3.8E-08	*dacC*
WP_000491520.1	2.46353	0.00226	*yedP*
WP_001292679.1	2.30525	2.2E-06	*mdoB*
WP_000436922.1	2.27911	0.0053	*ycbJ*
WP_001059922.1	2.2013	7.8E-06	*hypE*
WP_000190854.1	2.1995	0.00078	*ycgB*
WP_001253696.1	2.17048	0.00875	*envZ*
WP_000394140.1	2.12624	1.9E-05	*ansB*
WP_000624375.1	2.11382	2.3E-05	*folA*
WP_000989419.1	2.09378	0.01246	*astA*
WP_000925969.1	4.0901	1.7E-06	*ldtD*
WP_000459630.1	2.08903	0.01273	*tatD*
WP_001336277.1	2.05056	1.2E-05	*bglA*
WP_001297409.1	2.03771	0.01611	*tadA*
WP_000735412.1	2.01658	0.01775	*phoQ*
WP_000081588.1	2.01513	1.8E-05	*rpoS*
WP_001274963.1	2.00813	0.01845	*pspB*
WP_000426427.1	0.48755	5.6E-07	*dsdA*
WP_000845104.1	0.47602	2.3E-07	*asnA*

WP_000037608.1	0.47527	2E-05	*yhbO*
WP_001350521.1	0.46479	1.1E-05	*yedQ*
WP_000758655.1	0.46019	8.4E-06	*ygdR*
WP_000178044.1	0.4483	4.2E-06	*mliC*
WP_001300387.1	0.44345	9.6E-05	*garK*
WP_000890001.1	0.42062	6.7E-07	*truC*
WP_001308392.1	0.41722	9.2E-13	*ivy*
WP_001112301.1	0.41315	4E-07	*galP*
WP_001045689.1	0.40299	1.3E-05	*sbp*
WP_000646173.1	0.36176	1.1E-06	*ybdR*
WP_000498253.1	0.34248	9.8E-14	*osmB*
WP_000430057.1	0.34202	7.6E-10	*fiu*
WP_000761225.1	0.32266	8.8E-11	*malS*
WP_001309184.1	0.3172	1.6E-15	*nanM*
AIN31628.1	0.22799	2.4E-17	*BW25113_1171*
WP_000458809.1	0.2195	3.6E-18	*hcp*
WP_000281320.1	0.21767	2.3E-18	*ygaC*
WP_000868187.1	0.2058	9.9E-40	*rpmJ*
WP_001295684.1	0.19484	8.7E-30	*ydeN*
WP_000459379.1	0.14779	6.8E-20	*malY*
WP_000726974.1	0.08873	8.6E-44	*ymgG*
WP_000990333.1	0.0831	4.2E-46	*amiB*
WP_001295734.1	0.07192	2.2E-51	*nanC*
WP_000681354.1	0.57528	0.00779	*nhaA*
WP_001166395.1	0.72617	0.02402	*ispH*
WP_000597260.1	0.75275	0.03661	*carA*
WP_000736007.1	0.61402	0.01873	*mutT*
WP_000124438.1	0.69599	0.00576	*fhuA*
WP_001158931.1	0.61302	0.01834	*fhuC*
WP_000845394.1	1.8128	0.04469	*clcA*
WP_001291990.1	0.73816	0.02397	*gpt*
WP_001019920.1	0.61516	0.00524	*DXE50_03530*
WP_001102108.1	0.65099	0.01359	*KXD89_16980*
WP_000023927.1	0.63207	0.00837	*ECMP0215528_0369*
WP_000052206.1	1.4751	0.02913	*prpB*
WP_001275859.1	1.52746	0.00731	*HMPREF1599_03159*
WP_000413677.1	0.7623	0.04746	*ddl*
WP_001295332.1	1.64166	0.03482	*psiF*
WP_001295328.1	0.64444	0.00063	*DXE50_04210*
WP_001326929.1	0.6311	0.0264	*KXD89_16745*
WP_000844874.1	0.64375	0.03352	*priC*
WP_000738500.1	1.45019	0.01722	*bor*
WP_001201845.1	1.42015	0.02377	*ompT*
WP_001224604.1	1.62802	0.03801	*KXD89_15970*
WP_000381303.1	0.58105	0.00896	*A1UI_00656*
WP_000278509.1	1.75096	0.00052	*A1UI_00672*
WP_000503931.1	1.94949	0.00445	*tatE*
WP_000271153.1	1.86486	0.00043	*nagA*

WP_001113989.1	0.6236	0.02278	*nei*
WP_000254365.1	0.74511	0.03761	*sdhD*
WP_000568275.1	0.66499	0.00418	*DXE50_05885*
WP_000053415.1	0.65211	0.0009	*galK*
WP_000767389.1	1.55538	0.01297	*ECMP0215528_0917*
WP_000100800.1	1.93417	5.2E-05	*dps*
WP_001295296.1	1.5718	0.0044	*ompX*
WP_000815337.1	1.42197	0.04861	*poxB*
WP_001295343.1	0.67762	0.00625	*lolA*
WP_000850303.1	1.61106	0.04235	*dmsA*
WP_000165879.1	1.44322	0.01857	*A1UI_00969*
WP_000977920.1	0.73533	0.02199	*ompF*
WP_000048252.1	1.60609	0.04371	*yccX*
WP_001019197.1	0.60146	0.00349	*DXE50_07850*
WP_000420621.1	1.42312	0.04808	*cbpA*
WP_001151437.1	1.61626	0.00262	*wrbA*
AIN31510.1	1.80798	0.04566	-
WP_001144615.1	0.55664	0.00077	*trhO*
pdb|6H58|00	0.73734	0.02338	-
WP_000800153.1	0.55094	0.00415	*bhsA*
WP_000284280.1	1.89919	0.00628	*KXD89_12840*
WP_000702650.1	1.40496	0.02791	*narH*
WP_000718995.1	0.69111	0.00478	*galU*
WP_001360141.1	0.63673	0.0294	*tonB*
WP_001056490.1	1.64087	0.035	*KXD89_12400*
WP_001298828.1	0.50575	9.1E-05	*hslJ*
WP_001163872.1	1.35219	0.04805	*patD*
WP_000841554.1	1.5223	0.01819	*DXE50_16450*
WP_000246019.1	1.70277	0.02342	*gadC*
WP_000358930.1	1.77542	0.01448	*O3K_13030*
WP_000257409.1	0.64259	0.03281	*glsB*
WP_000214712.1	1.386	0.03402	*ydfZ*
WP_001300836.1	1.85322	0.03727	*A1UI_01776*
WP_001043339.1	1.75819	0.00047	*A1UI_01805*
WP_000483353.1	1.47956	0.01249	*hdhA*
WP_001296943.1	0.55992	5.1E-05	*slyA*
WP_000069375.1	0.69236	0.00502	*ppsA*
WP_001154168.1	1.47744	0.02847	*btuE*
WP_000267650.1	1.60056	0.00809	*DXE50_14915*
WP_000077872.1	1.40442	0.02807	*katE*
WP_000994973.1	1.86296	0.03567	*astB*
WP_000081983.1	1.56581	0.00471	*astC*
WP_001019882.1	1.48369	0.01193	*DXE50_14615*
WP_000394983.1	0.56835	0.00655	*DXE50_14440*
WP_000010115.1	0.50815	0.00115	*rlmA*
WP_000916763.1	0.70901	0.0478	*DXE50_14305*
WP_000936927.1	0.61509	0.01915	*ptrB*
WP_001350516.1	1.512	0.02018	*yebF*

WP_000891621.1	1.70638	0.00258	*A1UI_02070*
WP_001245695.1	1.59433	0.00864	*amyA*
WP_000740106.1	1.38884	0.03303	*msrP*
WP_001350529.1	1.60797	0.0432	*KXD89_08150*
WP_000490679.1	0.75665	0.04077	*DXE50_12405*
WP_000182899.1	0.76364	0.04917	*gatA*
WP_000153067.1	1.43572	0.04266	*O3K_08910*
WP_000422182.1	0.67197	0.02223	*yojI*
WP_000566471.1	1.78703	0.0134	*ECMP0215528_2665*
WP_000748271.1	1.40828	0.02695	*A1UI_02525*
WP_000991370.1	0.50596	9.2E-05	*evgA*
WP_000497400.1	0.6686	0.0019	*A1UI_02594*
WP_000021036.1	0.50089	0.0009	*cysA*
WP_001003709.1	1.65615	0.00163	*tal*
WP_000087280.1	1.49552	0.0238	*WG3_03045*
WP_001300814.1	0.71333	0.01732	*A1UI_02650*
WP_000133592.1	1.66697	0.00143	*pepB*
WP_000883122.1	0.71352	0.0174	*hmp*
WP_000986023.1	0.60834	0.01661	*acpS*
WP_000178456.1	1.47859	0.01263	*raiA*
WP_001203437.1	1.61073	0.04244	*bamE*
WP_000271909.1	1.38588	0.03406	*lhgO*
WP_000522415.1	1.59041	0.00354	*kbp*
WP_000777969.1	0.5006	0.00089	*nrdF*
WP_001199973.1	1.87674	0.00037	*queE*
WP_000626422.1	0.68335	0.00352	*sdaB*
WP_000016907.1	1.98765	0.00342	*DXE50_18860*
WP_001326497.1	0.69386	0.01014	*loiP*
WP_000994920.1	0.64399	0.03367	*yggU*
WP_000013149.1	1.40691	0.02734	*dkgA*
WP_000712658.1	1.74598	0.00055	*ygiW*
WP_000735278.1	1.70086	0.00095	*tolC*
WP_000046281.1	0.68739	0.0084	*A1UI_03332*
WP_001125331.1	0.72625	0.02406	*DXE50_20220*
WP_001066494.1	0.69351	0.03524	*A1UI_03348*
WP_000121433.1	1.42144	0.04885	*A1UI_03359*
WP_000031415.1	1.69309	0.00104	*ECMP0215528_3607*
WP_000785722.1	0.52762	8.2E-06	*DXE50_20455*
WP_000548347.1	0.56752	0.00642	*tdcB*
AIN33471.1	0.59149	0.00024	-
WP_001058209.1	0.58686	0.0022	*garL*
WP_001273753.1	0.64948	0.00247	*garD*
pdb|6H58|oo	0.74589	0.03012	-
WP_001300352.1	0.6827	0.00343	*gltB*
WP_000081674.1	0.67112	0.0051	*gltD*
WP_000979882.1	0.61511	0.00523	*ECMP0215528_3729*
WP_000209011.1	0.72079	0.02099	*nanK*
WP_000224714.1	0.6379	0.00045	*nanA*

WP_000695690.1	1.98787	0.02023	*O3K_02775*
WP_000354622.1	1.38054	0.03599	*accB*
WP_001219652.1	1.90568	0.02941	*dusB*
WP_001157751.1	1.44043	0.01914	*ompR*
WP_000856737.1	1.65146	0.03269	*greB*
WP_000444342.1	0.69685	0.01104	*malQ*
WP_000081909.1	0.56447	5.3E-06	*malP*
WP_000906961.1	0.64756	0.00231	*malT*
WP_001283723.1	1.49657	0.02356	*glgB*
WP_000108330.1	0.73424	0.02921	*gntK*
WP_001350553.1	1.88711	0.00033	*slp*
WP_000372240.1	1.60953	0.00283	*ECMP0215528_4059*
WP_001296794.1	1.9492	0.02414	*ECMP0215528_4067*
WP_001295233.1	0.66721	0.01996	*HMPREF1589_01249*
pdb|6H58|11	0.62209	0.0002	-
WP_001243431.1	0.65765	0.00327	*ibpB*
WP_001243437.1	0.56797	6.8E-06	*ibpA*
WP_001291268.1	1.6522	0.00466	*yieF*
WP_000763724.1	0.72424	0.02289	*EL76_4667*
WP_000281668.1	0.66034	0.04499	*A1UI_04118*
WP_000591375.1	0.7014	0.00704	-
WP_000940106.1	0.71048	0.04915	*DXE50_25690*
WP_001252058.1	0.60827	0.01659	*malG*
WP_001297290.1	0.55203	0.00064	*malF*
WP_000695387.1	0.55722	3.1E-06	*malE*
WP_000179165.1	0.6329	0.00035	*malK*
WP_000973663.1	0.58528	2.2E-05	*lamB*
WP_001326641.1	0.67306	0.0023	*malM*
WP_000002907.1	0.70491	0.04419	*DXE50_25955*
WP_001014565.1	1.89183	0.0066	*EKN05_018200*
WP_001131339.1	1.81878	0.04351	*HMPREF1602_03064*
WP_001298520.1	1.66727	0.00396	*HMPREF1599_00791*
WP_001295074.1	1.62661	0.00232	*lysS*
WP_001350567.1	1.95734	0.02326	*KXD89_19795*
WP_000853753.1	0.72311	0.01493	*fbp*
WP_001336303.1	0.67935	0.00299	*G711_01352*
WP_001407733.1	0.75656	0.04067	*treB*
WP_001181307.1	0.59136	0.01141	*G711_01354*
WP_000148581.1	0.71385	0.01095	*pyrI*
WP_000013046.1	0.65255	0.00092	*pyrB*
WP_000583469.1	0.75447	0.03841	*EKN05_017700*
WP_001309160.1	1.69988	0.02386	*G894_04371*
WP_000611568.1	0.68585	0.00802	*A13A_04926*
WP_000879155.1	0.56818	0.00117	*HMPREF1599_03219*
WP_000188283.1	0.51124	2.9E-06	*HMPREF1599_03218*
WP_000563040.1	0.60878	0.01677	*G925_04733*
WP_001338221.1	0.66377	0.0477	*O3K_21575*
WP_001264707.1	0.81635	0.1628	*A13A_05080*

WP_000241662.1	0.74519	0.09024	*thrB*
WP_000781074.1	0.95181	0.93496	*thrC*
WP_000906197.1	1.03389	0.88064	*yaaA*
WP_000130185.1	0.92334	0.73266	*tal*
WP_001295414.1	0.97072	0.79965	*EKN05_016655*
WP_000516135.1	0.84478	0.27015	*dnaK*
WP_001118476.1	0.95135	0.93171	*dnaJ*
pdb|6H58|tt	0.88207	0.46355	-
WP_000767329.1	0.91887	0.62089	*ribF*
WP_001286857.1	0.90312	0.59533	*ileS*
WP_000004655.1	0.84705	0.23238	*fkpB*
WP_001239142.1	1.08333	0.67653	*rihC*
WP_000543604.1	1.21412	0.28519	*dapB*
WP_001126348.1	0.77589	0.06712	*carB*
WP_000377098.1	1.17225	0.60238	*kefC*
WP_000257192.1	1.05322	0.79791	*apaH*
WP_000610901.1	1.31047	0.36755	*apaG*
WP_001065381.1	0.88888	0.38893	*rsmA*
WP_000241277.1	0.88659	0.48433	*pdxA*
WP_000800457.1	0.98514	0.88549	*surA*
WP_000746151.1	1.04462	0.62887	*lptD*
WP_001200579.1	1.13847	0.58471	*djlA*
WP_000525176.1	0.85565	0.44053	*rluA*
WP_001117011.1	0.92429	0.7393	*rapA*
WP_001300811.1	0.9002	0.57628	*araC*
WP_001301364.1	1.20395	0.4322	*HMPREF1589_03362*
WP_000818228.1	0.85399	0.25514	*leuD*
WP_001140652.1	0.90179	0.44603	*leuC*
WP_000042353.1	1.04881	0.81646	*leuB*
WP_000082850.1	0.93193	0.5925	*leuA*
WP_001115472.1	0.68064	0.0628	*A1UI_04836*
WP_001295534.1	0.8865	0.48395	*A1UI_04837*
WP_001300383.1	0.94017	0.74674	*A1UI_04838*
WP_000762401.1	0.92887	0.77145	*cra*
WP_001295533.1	1.13733	0.67605	*mraZ*
WP_000970479.1	0.96734	0.78113	*rsmH*
WP_000625658.1	0.87573	0.50867	*ftsL*
WP_000642196.1	1.09243	0.71129	*ftsI*
WP_000775093.1	0.95324	0.94522	*murE*
WP_000626685.1	0.98404	0.87311	*murF*
WP_000964131.1	1.12282	0.70836	*mraY*
WP_000796481.1	1.01371	0.97007	*murD*
WP_001295532.1	0.83355	0.37032	*ftsW*
WP_000016560.1	1.20449	0.43107	*murG*
WP_001096049.1	1.01332	0.97184	*murC*
WP_000130056.1	1.01358	0.97067	*ddl*
WP_000075748.1	0.75121	0.16423	*ftsQ*
WP_000588474.1	0.94555	0.66349	*ftsA*

WP_000462776.1	1.0943	0.45192	*ftsZ*
WP_000595482.1	1.48293	0.18594	*lpxC*
WP_000905789.1	0.8173	0.1658	*secA*
WP_000005042.1	1.12315	0.70761	*yacG*
WP_001194734.1	0.89325	0.51151	*zapD*
WP_001269520.1	0.85384	0.43456	*coaE*
WP_001217338.1	0.95184	0.93517	*guaC*
WP_001135174.1	0.93232	0.59449	*nadC*
WP_000923721.1	1.01528	0.97549	*ampD*
WP_000172005.1	0.99652	0.96341	*ampE*
WP_000331776.1	0.99487	0.9684	*pdhR*
WP_000003820.1	0.91737	0.69133	*aceE*
WP_000963518.1	0.97162	0.94895	*aceF*
WP_000102485.1	0.90815	0.62873	*lpdA*
WP_001307570.1	1.01333	0.75858	*acnB*
WP_000384306.1	1.26451	0.4358	*yacL*
WP_000734287.1	0.9611	0.74704	*speD*
WP_000818411.1	1.00619	0.99511	*speE*
WP_001295568.1	0.80198	0.28028	*DXE50_02840*
WP_001189647.1	1.33372	0.2214	*A1UI_04880*
WP_001306211.1	0.9638	0.82485	*HMPREF1595_04252*
WP_000683335.1	0.76557	0.05846	*DXE50_02855*
WP_000651599.1	0.94031	0.85274	*DXE50_02860*
WP_000150637.1	0.98231	0.86352	*DXE50_02865*
WP_000621515.1	1.00898	0.97557	*panD*
WP_000905383.1	1.03044	0.89572	*panC*
WP_000805497.1	1.10127	0.43012	*panB*
WP_000215139.1	0.84768	0.41458	*folK*
WP_010723084.1	0.76814	0.12839	*pcnB*
WP_001155227.1	1.14991	0.29874	*dksA*
WP_000396036.1	0.90082	0.59857	*sfsA*
WP_001350480.1	0.9749	0.87627	*hrpB*
WP_000918162.1	0.96317	0.98922	*mrcB*
WP_001310529.1	0.86023	0.45571	*HMPREF1602_04663*
WP_001295564.1	1.38027	0.07156	*erpA*
WP_000689844.1	0.93655	0.8259	*mtnN*
WP_000057073.1	1.27107	0.42547	*dgt*
WP_000272188.1	1.12433	0.52901	*yaeH*
WP_001186650.1	0.92808	0.76589	*dapD*
WP_001094586.1	0.88223	0.36101	*glnD*
WP_001018194.1	0.85153	0.30086	*map*
pdb|7OE0|B	0.94253	0.86861	-
WP_000818114.1	0.85373	0.31128	*tsf*
WP_000224573.1	0.87271	0.4098	*pyrH*
WP_000622418.1	0.98028	0.90812	*frr*
WP_000811923.1	0.78308	0.2328	*dxr*
WP_001295562.1	1.05958	0.85999	*ispU*
WP_000922446.1	1.46333	0.2015	*cdsA*

WP_001295561.1	1.07821	0.7534	*rseP*
WP_001240896.1	0.96483	0.9813	*bamA*
WP_000758956.1	1.17448	0.24545	*skp*
WP_001139279.1	0.91187	0.49311	*lpxD*
WP_000210739.1	0.9834	0.89354	*fabZ*
WP_000565966.1	1.09192	0.64385	*lpxA*
WP_000139654.1	1.46351	0.20135	*lpxB*
WP_000569430.1	0.94992	0.78421	*rnhB*
WP_001294757.1	1.13581	0.6794	*dnaE*
WP_000055741.1	0.96903	0.96124	*accA*
WP_001020973.1	1.50739	0.16806	*ldcC*
WP_000901099.1	1.01331	0.96099	*KXD89_17755*
WP_000176549.1	1.14245	0.6649	*tilS*
AIN30718.1	1.3214	0.23674	-
WP_000417058.1	1.33643	0.21813	*yaeP*
WP_001185290.1	0.95263	0.79466	*DXE50_03195*
WP_000239163.1	1.49961	0.08476	*nlpE*
WP_001260717.1	0.90438	0.60362	*proS*
WP_000094011.1	1.01606	0.97338	*tsaA*
WP_001202329.1	0.86006	0.27613	*rcsF*
WP_000874226.1	1.01418	0.75491	*WG3_00475*
WP_000594006.1	1.05222	0.83375	*metN*
WP_001140187.1	0.75849	0.11119	*gmhB*
WP_000997010.1	0.74602	0.09145	*dkgB*
WP_000648572.1	1.52717	0.15476	*KXD89_17630*
WP_001230983.1	1.47639	0.19101	*yafD*
WP_001052715.1	1.0794	0.74984	*gloB*
WP_000917883.1	0.85327	0.43271	*rnhA*
WP_001340895.1	0.92192	0.67724	*dnaQ*
WP_001118036.1	1.31126	0.25003	*yafV*
WP_000973083.1	1.04626	0.82732	*A1UI_00324*
WP_000284050.1	1.07233	0.52551	*lpcA*
WP_000333380.1	0.92693	0.69622	*ECMP0215528_0302*
WP_000729703.1	1.27459	0.30345	*dinJ*
WP_001292994.1	0.95562	0.96221	*pepD*
WP_000189532.1	0.97586	0.82797	*frsA*
WP_000174689.1	1.13901	0.48164	*crl*
WP_001285288.1	0.96482	0.76734	*proB*
WP_000893278.1	1.08697	0.47563	*proA*
WP_000081352.1	1.38877	0.27179	*A1UI_00392*
WP_001136613.1	1.0884	0.65713	*yagE*
WP_000151261.1	0.99478	0.95679	*yagF*
WP_000406871.1	0.97042	0.86329	*yagH*
WP_001121657.1	1.0879	0.78998	*kdgR_2*
WP_000803998.1	0.66951	0.0525	*rpmE2*
AIN30811.1	1.17912	0.58857	-
WP_001046307.1	1.27557	0.30191	*KXD89_16970*
WP_000370307.1	0.77926	0.07675	*KXD89_16990*

WP_000089110.1	1.0248	0.92265	*betB*
WP_000665120.1	0.83231	0.36654	*KZW89_19055*
WP_000692754.1	1.30743	0.07473	*A1UI_00416*
WP_000691956.1	1.34921	0.09457	*ECMP0215528_0390*
WP_001285927.1	1.4093	0.05475	*prpC*
WP_000076233.1	0.80794	0.29627	*codB*
WP_001301240.1	0.81402	0.1419	*A1UI_00427*
WP_000952503.1	0.83302	0.36868	*A1UI_00428*
WP_071843335.1	0.86787	0.41133	-
WP_001301325.1	0.91205	0.64016	*KXD89_17195*
WP_001013499.1	0.84016	0.39072	*mhpE*
WP_001096705.1	0.99066	0.94907	*DXE50_03945*
WP_000419081.1	0.8602	0.38301	*fghA*
WP_000842106.1	0.9761	0.82925	*frmA*
WP_001141271.1	0.94179	0.72368	*frmR*
WP_001295337.1	1.15036	0.29769	*hemB*
WP_000092067.1	0.86177	0.46089	*yaiW*
WP_000792970.1	1.19231	0.33449	*iraP*
WP_001295331.1	1.20797	0.29849	*proC*
WP_000158159.1	1.19008	0.46177	*yaiI*
WP_001276420.1	0.75786	0.17732	*DXE50_04100*
WP_000941942.1	1.12122	0.63031	*ppnP*
WP_001298537.1	0.95101	0.69261	*rdgC*
WP_001219309.1	1.17446	0.49682	*mak*
WP_000698951.1	1.15614	0.63566	*sbcC*
WP_000113933.1	0.88531	0.47918	*phoB*
WP_000893623.1	1.03253	0.92972	*phoR*
WP_000149639.1	0.97558	0.88318	*brnQ*
WP_001295329.1	1.26436	0.43603	*proY*
AIN30902.1	1.22995	0.49346	-
WP_001266503.1	1.11651	0.55543	*queA*
WP_000667319.1	0.81228	0.13794	*tgt*
WP_000007629.1	1.03277	0.67646	*yajC*
WP_000934822.1	0.90534	0.61001	*secD*
WP_000046637.1	0.93235	0.59466	*secF*
WP_000974813.1	0.91956	0.62391	*DXE50_04205*
WP_000543535.1	1.02972	0.90644	*nrdR*
WP_001150457.1	0.97823	0.89166	*ribD*
WP_001021161.1	0.77525	0.06607	*ribH*
WP_000801125.1	1.02127	0.93626	*nusB*
WP_000742109.1	0.91037	0.63389	*thiL*
WP_001199800.1	1.25965	0.20084	*A1UI_00501*
WP_000006797.1	0.91719	0.51878	*dxs*
WP_000347217.1	1.07218	0.77167	*A1UI_00503*
WP_001124935.1	0.8572	0.44564	*xseB*
WP_000668662.1	0.91794	0.52245	*thiI*
WP_001276305.1	0.90732	0.57071	*A1UI_00506*
WP_000705865.1	0.99262	0.95808	*panE*

WP_001138904.1	0.91527	0.67691	*yajQ*
WP_000467180.1	0.8902	0.51282	*DXE50_04315*
WP_001239436.1	0.80551	0.13099	*WG3_00715*
WP_001295326.1	0.82911	0.20657	*DXE50_04330*
WP_000973448.1	1.04105	0.84959	*bolA*
WP_001198386.1	0.88509	0.48161	*tig*
WP_000122253.1	1.02421	0.71203	*clpP*
WP_000130305.1	1.01767	0.73985	*clpX*
WP_001295325.1	1.05679	0.58207	*lon*
WP_001043542.1	0.96782	0.96701	*hupB*
WP_000969372.1	0.95293	0.94295	*ppiD*
WP_001194534.1	1.32241	0.23545	*DXE50_04375*
WP_000817220.1	0.8183	0.24674	*queC*
WP_000884589.1	0.66999	0.05291	*DXE50_04395*
WP_000075876.1	0.90277	0.45051	*tesB*
WP_000779842.1	0.92848	0.57496	*KXD89_16535*
WP_000136192.1	0.85119	0.24581	*DXE50_04450*
WP_000102564.1	0.88623	0.48285	*DXE50_04455*
WP_001291435.1	1.22386	0.39226	*hha*
WP_001132469.1	0.86583	0.37235	*DXE50_04470*
WP_001295324.1	0.9309	0.78585	*acrA*
WP_000101737.1	1.18917	0.56878	*acrR*
WP_000177732.1	1.04514	0.85632	*mscK*
WP_000051153.1	1.41717	0.13831	*rsmS*
WP_000127356.1	1.08228	0.49127	*apt*
WP_000122013.1	1.18608	0.3497	*dnaX*
WP_000467098.1	0.86183	0.35151	*ybaB*
WP_001195025.1	0.73702	0.1384	*recR*
WP_000678201.1	0.87125	0.40167	*htpG*
WP_001220233.1	0.9193	0.70463	*adk*
WP_001250103.1	1.31181	0.36571	*hemH*
WP_000671574.1	0.90065	0.44081	*gsk*
WP_000546237.1	0.79217	0.17891	*A1UI_00561*
WP_001251608.1	1.14813	0.65265	*A1UI_00562*
WP_000771748.1	1.31695	0.06813	*A13A_00461*
WP_000186631.1	1.0167	0.94963	*ybaK*
WP_000806442.1	1.19667	0.55433	*A13A_00465*
WP_000083955.1	1.39245	0.06401	*copA*
WP_001026747.1	0.94472	0.76423	*cueR*
WP_000904502.1	1.66928	0.08429	*DXE50_04660*
WP_001300573.1	0.8644	0.36483	*cnoX*
WP_000148959.1	1.26231	0.43929	*ybbO*
WP_001295836.1	1.08667	0.72819	*ECMP0215528_0556*
WP_001110573.1	1.10276	0.60391	*DXE50_04690*
WP_000141275.1	0.99616	0.94	*allR*
WP_001333621.1	1.26855	0.42941	*glxK*
WP_000815571.1	0.98379	0.91472	*purK*
WP_001295318.1	0.97671	0.88462	*purE*

WP_000255997.1	0.95387	0.94966	*ppiB*
WP_000912385.1	0.83895	0.24523	*cysS*
WP_000190288.1	0.95974	0.73966	*ybcJ*
WP_000729160.1	0.90849	0.47709	*folD*
WP_000662357.1	0.85891	0.4513	*nfrA*
WP_000770953.1	1.08868	0.78811	*cusR*
WP_000786319.1	1.04275	0.90307	*pheP*
WP_001153148.1	0.79327	0.25778	*DXE50_05095*
WP_000351487.1	0.98597	0.88163	*nfsB*
WP_001130654.1	1.65611	0.08926	*KXD89_15900*
WP_001034943.1	0.83456	0.19473	*fepA*
WP_000026812.1	0.89234	0.50773	*entE*
WP_001007138.1	0.95315	0.77574	*A1UI_00658*
WP_000347651.1	0.95155	0.76835	*A1UI_00659*
WP_001043156.1	1.28254	0.16694	*A1UI_00661*
WP_000913829.1	0.96879	0.84797	*dsbG*
WP_000052796.1	0.95778	0.97761	*ahpC*
WP_000887629.1	0.89024	0.5131	*ahpF*
WP_000089731.1	1.07847	0.69542	*rnk*
WP_000643397.1	0.85403	0.36092	*A1UI_00676*
WP_000062457.1	0.6866	0.0689	*citG*
WP_000034825.1	0.77316	0.06274	*cspE*
WP_000042632.1	0.8012	0.1145	*lipA*
WP_000284027.1	0.72996	0.06993	*lipB*
WP_000850550.1	1.02976	0.8987	*ybeD*
WP_001092082.1	1.0264	0.70283	*dacA*
WP_001231430.1	1.11063	0.57586	*rlpA*
WP_000131719.1	0.91228	0.64102	*mrdB*
WP_000776191.1	1.14993	0.55567	*mrdA*
WP_000776104.1	1.04672	0.85127	*rlmH*
WP_001161664.1	0.89358	0.40928	*rsfS*
WP_000620535.1	0.92552	0.69086	*EL76_3432*
WP_001269673.1	0.99825	0.98388	*lptE*
WP_001340834.1	0.85059	0.29644	*leuS*
WP_001044880.1	1.33978	0.05441	*DXE50_05445*
WP_001207520.1	0.95386	0.70792	*rihA*
WP_001177086.1	1.26484	0.19269	*gltI*
WP_000853021.1	0.8868	0.48517	*lnt*
WP_001278605.1	1.15629	0.42967	*corC*
WP_000084469.1	0.822	0.33589	*ybeY*
WP_001018040.1	0.89647	0.55225	*ybeZ*
WP_000162740.1	0.90816	0.62878	*miaB*
WP_000184541.1	0.76034	0.11435	*A1UI_00729*
WP_000337077.1	0.78125	0.0764	*WG3_00959*
WP_000153129.1	1.02039	0.94019	*nagD*
WP_000187594.1	1.119	0.54691	*nagC*
WP_001237072.1	0.78689	0.08864	*nagB*
WP_038432907.1	0.95675	0.72348	-

WP_001287154.1	0.91155	0.65155	*glnS*
WP_000131702.1	1.0407	0.64438	*fur*
WP_001018618.1	0.92645	0.75445	*fldA*
WP_001300829.1	0.99677	0.96438	*DXE50_05640*
WP_000773288.1	0.83029	0.18281	*ybfF*
WP_000848387.1	0.92375	0.55113	*seqA*
WP_001297249.1	1.06275	0.55995	*A1UI_00749*
WP_001310640.1	1.18424	0.57844	*A1UI_00756*
WP_000087939.1	0.9586	0.81767	*kdpB*
WP_000424924.1	1.13698	0.67682	*DXE50_05690*
WP_001053305.1	1.18345	0.57999	*ECMP0215528_0806*
WP_000798871.1	1.12976	0.34885	*DXE50_05710*
WP_001188343.1	0.87204	0.42712	*ECMP0215528_0810*
WP_000912724.1	1.08753	0.72565	*pxpC*
WP_000785834.1	0.99291	0.84971	*gltA*
AIN31197.1	0.84691	0.23193	-
WP_000775540.1	0.98519	0.88526	*sdhA*
WP_001235254.1	1.03229	0.67843	*A311_01246*
WP_001181473.1	0.90967	0.63888	*sucA*
WP_000099823.1	0.9641	0.98479	*WG3_01000*
WP_001048602.1	1.04163	0.6407	*sucC*
WP_000025458.1	1.04193	0.63949	*sucD*
WP_000509902.1	0.81552	0.31731	*mngR*
WP_000884361.1	1.0365	0.66128	*O3K_17975*
WP_000034602.1	1.01232	0.98342	*ybgE*
WP_001098384.1	0.86827	0.4829	*ybgC*
WP_000131314.1	0.93008	0.58309	*tolQ*
WP_000090097.1	0.97218	0.87006	*tolR*
WP_000030637.1	0.9279	0.57199	*tolA*
WP_001295307.1	1.07866	0.50354	*tolB*
WP_001295306.1	1.00588	0.79132	*pal*
WP_000097571.1	0.96876	0.78892	*cpoB*
WP_000784351.1	1.52436	0.15659	*DXE50_05990*
WP_001109196.1	0.97545	0.8257	*O3K_17900*
WP_001295305.1	0.99301	0.84925	*gpmA*
WP_000931389.1	0.96119	0.99871	*galM*
WP_000191497.1	0.7629	0.05532	*A1UI_00817*
WP_001265438.1	0.84068	0.25245	*galE*
WP_000096869.1	0.90315	0.55292	*G711_00399*
WP_001147439.1	0.90376	0.55549	*modE*
WP_000101984.1	1.0234	0.92679	*A1UI_00822*
WP_000891692.1	1.6354	0.09764	*modC*
WP_001300666.1	0.94489	0.66001	*A1UI_00825*
WP_000815435.1	1.10403	0.42169	*pgl*
WP_001091569.1	0.84252	0.21824	*ECMP0215528_0877*
WP_001295303.1	1.77385	0.05317	*bioA*
WP_000951213.1	0.72695	0.1218	*bioB*
WP_000044843.1	1.00058	0.97885	*bioD*

WP_000042533.1	1.05071	0.83855	*uvrB*
WP_001295302.1	1.05317	0.83075	*ECMP0215528_0925*
WP_000084639.1	1.25753	0.20425	*moaB*
WP_000080885.1	1.10881	0.66449	*moaC*
WP_000852296.1	0.99276	0.9587	*moaE*
WP_001393495.1	0.94452	0.65808	*hlyD*
WP_001296991.1	1.48208	0.1866	*cecR*
WP_000007101.1	0.95515	0.78492	*rhlE*
WP_000386551.1	1.01187	0.97839	*ECMP0215528_0948*
WP_000443530.1	1.13464	0.49541	*ECMP0215528_0949*
WP_001295299.1	1.17578	0.4938	*rlmF*
WP_000569083.1	0.86737	0.30271	*ECMP0215528_0957*
WP_001159065.1	0.92548	0.65015	*glnP*
WP_000843866.1	0.9164	0.68465	*glnH*
WP_000091016.1	0.84693	0.33635	*DXE50_06600*
WP_001056384.1	0.91727	0.51915	*ldtB*
WP_000961458.1	0.88929	0.5072	*ECMP0215528_0968*
WP_000168797.1	1.15477	0.54368	*ECMP0215528_0969*
WP_000114272.1	0.87368	0.32678	*ybiV*
WP_001336208.1	1.23195	0.48996	*fsa*
WP_000829217.1	1.03727	0.91732	*moeB*
WP_000397340.1	0.93717	0.83032	*moeA*
WP_000513781.1	1.02208	0.95734	*iaaA*
WP_001301279.1	1.16556	0.61604	*gsiA*
WP_000090140.1	1.17346	0.49913	*gsiB*
WP_000049367.1	0.80122	0.11972	*rimO*
WP_000555031.1	1.13945	0.67142	*A1UI_00905*
WP_001295292.1	1.03919	0.8576	*DXE50_06705*
WP_000450121.1	0.95999	0.80726	*deoR*
WP_000023565.1	1.17083	0.60526	*ybjI*
WP_001024876.1	1.04328	0.90171	*ybjL*
WP_001195240.1	1.10207	0.75621	*EKN05_012210*
WP_000189159.1	0.94833	0.67828	*nfsA*
WP_000203025.1	0.908	0.62508	*DXE50_06785*
WP_000126072.1	1.72773	0.06523	*O3K_17075*
WP_001295339.1	1.11397	0.72853	*DXE50_06835*
WP_000756569.1	1.08604	0.47872	*ECMP0215528_1029*
WP_000027205.1	1.00076	0.99533	*artP*
WP_001270734.1	1.05599	0.78634	*O3K_17020*
WP_001160737.1	1.27037	0.31016	*ybjQ*
WP_001252135.1	0.94104	0.75007	*amiD*
WP_001338420.1	0.95642	0.80928	*ECMP0215528_1034*
WP_000566376.1	0.91958	0.53048	*ltaE*
WP_000491142.1	0.80194	0.28019	*ECMP0215528_1040*
WP_000599802.1	0.68714	0.06947	*KXD89_14410*
WP_000746442.1	0.74895	0.09585	*macA*
WP_000410785.1	1.10462	0.59719	*cspD*
WP_000520781.1	0.82263	0.33773	*clpS*

WP_000934041.1	1.3153	0.06923	*clpA*
WP_001040187.1	0.89706	0.55604	*infA*
WP_001202177.1	1.06197	0.80312	*cydC*
WP_001043598.1	1.05219	0.83386	*cydD*
WP_000537418.1	0.96859	0.96335	*trxB*
WP_000228473.1	1.00353	0.80176	*GA0061070_10177*
WP_000076967.1	0.95066	0.76427	*ftsK*
WP_000067755.1	0.94732	0.77421	*DXE50_07020*
WP_000886683.1	0.91375	0.66651	*serS*
WP_000213098.1	1.00613	0.99987	*dmsB*
WP_000111043.1	0.88707	0.3812	*pflA*
WP_001292822.1	1.04751	0.61756	*A13A_00842*
WP_000642546.1	1.46191	0.10632	*focA*
WP_001295344.1	0.88681	0.49203	*DXE50_07080*
WP_000642849.1	1.44254	0.21926	*ECMP0215528_1073*
WP_000057138.1	0.93545	0.81808	*serC*
WP_000445231.1	0.94309	0.65049	*aroA*
WP_000125016.1	0.86821	0.30586	*cmk*
WP_000140327.1	0.87299	0.41132	*rpsA*
WP_000167336.1	1.33868	0.10374	*ihfB*
WP_000551270.1	1.0076	0.98025	*msbA*
WP_000570539.1	1.03624	0.92	*lpxK*
WP_000350058.1	0.82909	0.27869	*ycaR*
WP_000011603.1	1.09359	0.6376	*kdsB*
WP_001298300.1	0.89533	0.57853	*cmoM*
WP_001288850.1	0.90908	0.57823	*mukF*
WP_001295347.1	0.95492	0.78386	*mukE*
WP_000572698.1	0.97589	0.92874	*mukB*
WP_001109486.1	1.28895	0.15836	*A1UI_01038*
WP_000462687.1	0.94248	0.86824	*DXE50_07195*
WP_000117881.1	0.90393	0.60066	*asnS*
WP_001307697.1	0.90363	0.45446	*pncB*
WP_000193841.1	1.00841	0.78011	*A1UI_01043*
WP_001295352.1	0.8006	0.11817	*pyrD*
WP_000212426.1	1.02111	0.9349	*A1UI_01060*
WP_001086539.1	1.04951	0.81351	*rlmL*
WP_000053099.1	0.94479	0.65948	*uup*
WP_000445533.1	1.05773	0.81638	*O3K_16585*
WP_000759120.1	1.35644	0.19532	*pqiC*
WP_000227927.1	0.91701	0.68885	*fabA*
WP_000156518.1	0.97561	0.87955	*A1UI_01068*
WP_000877161.1	0.87743	0.5146	*matP*
WP_000750416.1	1.20989	0.1824	*ompA*
WP_001261235.1	0.83298	0.29073	*DXE50_07360*
WP_001295354.1	1.11555	0.38783	*A1UI_01075*
WP_000424181.1	0.83089	0.21327	*mgsA*
WP_001301418.1	1.16784	0.39724	*KXD89_13960*
WP_000116297.1	0.98925	0.90188	*rlmI*

WP_000904442.1	0.86553	0.4026	*tusE*
WP_000375136.1	0.92143	0.67537	*yccA*
WP_001300464.1	0.82791	0.3533	*G711_00113*
WP_001044279.1	1.15063	0.44623	*agp*
WP_001143120.1	1.41568	0.05157	*DXE50_07910*
WP_000191701.1	1.13272	0.68622	*rutR*
WP_001326840.1	0.97377	0.93876	*putA*
WP_001018479.1	0.84104	0.21374	*putP*
WP_000154398.1	0.72529	0.06444	*EKN05_011205*
WP_000533522.1	1.32347	0.34993	*phoH*
WP_000351317.1	1.08398	0.48554	*ghrA*
WP_000283667.1	1.17907	0.36745	*ycdX*
WP_001001921.1	1.05295	0.79902	*A13A_00953*
WP_001189321.1	1.44676	0.11631	*csgG*
WP_000857405.1	1.26374	0.32094	*ymdB*
WP_001343212.1	0.94304	0.87224	*mdoG*
WP_001295445.1	0.93603	0.6975	*mdoH*
WP_000180056.1	1.30993	0.13287	*ECMP0215528_1226*
WP_000183364.1	0.93462	0.69116	*lpxL*
WP_000749261.1	1.04732	0.84935	*yceI*
WP_000872833.1	1.03792	0.86313	*solA*
WP_001217754.1	0.97368	0.87584	*dinI_4*
WP_000126534.1	0.85564	0.32051	*pyrC*
WP_001295443.1	0.79767	0.19206	*DXE50_08675*
WP_000780912.1	1.1165	0.38512	*O3K_15240*
WP_000468186.1	1.13349	0.59765	*rimJ*
WP_000877107.1	1.16762	0.39784	*yceH*
WP_001050683.1	0.84721	0.41308	*murJ*
WP_000827360.1	0.9269	0.75762	*rne*
WP_000846343.1	1.18057	0.3636	*rluC*
WP_001125202.1	1.10862	0.58293	*EKN05_010785*
WP_001174481.1	0.92355	0.64154	*yceD*
WP_000197597.1	1.23249	0.48902	*plsX*
WP_000288132.1	1.03686	0.65979	*fabH*
WP_000191372.1	1.06602	0.54804	*fabD*
WP_001008535.1	1.05165	0.60155	*fabG*
WP_000103754.1	0.91807	0.69613	*acpP*
WP_000044679.1	0.93509	0.8155	*fabF*
WP_000478681.1	1.12964	0.69305	*pabC*
WP_000756827.1	0.91903	0.66631	*yceG*
WP_001257000.1	0.92772	0.66012	*tmk*
WP_001267956.1	1.17002	0.60691	*holB*
WP_000480245.1	1.07769	0.75497	*ECMP0215528_1276*
WP_000475719.1	0.99306	0.84903	*ptsG*
WP_000807125.1	1.01056	0.98428	*hinT*
WP_000164439.1	1.08518	0.66943	*lpoB*
WP_000529320.1	0.99875	0.98616	*nagZ*
WP_000587933.1	1.23034	0.25239	*ycfP*

WP_000211045.1	0.94078	0.85607	*DXE50_08910*
WP_001043459.1	0.8499	0.34653	*DXE50_08915*
WP_001336528.1	1.18642	0.57416	*comR*
WP_001115094.1	1.01209	0.76402	*mfd*
WP_000284714.1	1.06666	0.8422	*lolC*
WP_001033694.1	1.10629	0.67156	*lolD*
WP_001251348.1	0.96166	0.82949	*HMPREF1620_01675*
WP_000291270.1	1.17005	0.60685	*nagK*
AIN31583.1	1.02465	0.92317	-
WP_000759317.1	0.98933	0.90234	*potD*
WP_000580316.1	1.02238	0.93069	*potC*
AIN31588.1	1.00257	0.98638	-
WP_000531594.1	1.00948	0.98917	*potA*
WP_000359434.1	1.30417	0.13949	*pepT*
WP_000456506.1	1.02436	0.92411	*AB05_1317*
WP_000423742.1	0.96317	0.98921	*purB*
WP_001297479.1	1.06422	0.79613	*hflD*
WP_001297484.1	0.93794	0.62356	*trmU*
WP_000476093.1	1.32076	0.35355	*nudJ*
WP_000444484.1	0.94233	0.86717	*icd*
WP_000848748.1	0.82654	0.34922	*A311_03318*
WP_000888772.1	0.67962	0.0618	*ariR*
WP_001185665.1	0.91918	0.52851	*minE*
WP_000101055.1	1.10434	0.42077	*minD*
WP_001301105.1	1.08877	0.72199	*minC*
WP_001056840.1	0.73219	0.07267	*HMPREF1602_05104*
WP_000695215.1	1.04002	0.87281	*KXD89_12850*
WP_001295992.1	0.94612	0.66652	*ycgL*
WP_000943459.1	0.96616	0.84684	*dsbB*
WP_000406391.1	0.76015	0.11403	*nhaB*
WP_000234823.1	0.8679	0.41144	*fadR*
WP_001266908.1	0.93618	0.82324	*dadA*
WP_000197881.1	1.13371	0.49838	*dadX*
WP_000051560.1	1.04819	0.84658	*A1UI_01327*
WP_001301104.1	0.85682	0.37084	*emtA*
WP_000841714.1	1.34023	0.10234	*treA*
WP_001301101.1	0.97338	0.81432	*A1UI_01333*
WP_000059411.1	0.98689	0.88886	*EL76_2821*
WP_000733715.1	1.13044	0.34707	*A1UI_01335*
WP_000505866.1	0.93565	0.81949	*ychF*
WP_000152933.1	0.87289	0.32372	*pth*
WP_000823885.1	1.11267	0.65374	*ychH*
WP_001033352.1	0.94965	0.75962	*dauA*
WP_001298109.1	0.96093	1	*prs*
WP_001260332.1	0.93604	0.69755	*ispE*
WP_001130692.1	1.06157	0.76327	*lolB*
WP_001299679.1	1.10918	0.73959	*hemA*
WP_000804726.1	1.06009	0.76936	*prfA*

WP_000456467.1	0.95669	0.8103	*prmC*
WP_000811065.1	1.06646	0.54647	*kdsA*
WP_000063607.1	1.61491	0.10666	*chaA*
WP_001146444.1	1.06637	0.84293	*chaB*
WP_001169669.1	0.99515	0.93443	*ychN*
WP_000070491.1	0.98183	0.86089	*narL*
WP_000918073.1	1.20054	0.43932	*narX*
WP_000019827.1	1.30333	0.26087	*narK*
WP_000032939.1	1.21689	0.17169	*KXD89_12605*
WP_000571681.1	1.33354	0.22161	*narJ*
WP_001160108.1	1.41301	0.14168	*narI*
WP_000555857.1	0.92352	0.54998	*purU*
WP_001307143.1	0.97483	0.8803	*ychJ*
WP_000193447.1	1.126	0.7012	*rssB*
WP_001287378.1	1.08268	0.4899	*DXE50_00785*
WP_000068077.1	0.97464	0.87507	*tdk*
WP_000301651.1	1.0954	0.44841	*adhE*
WP_001393463.1	1.08807	0.47202	*oppA*
WP_000911112.1	1.03634	0.86997	*oppB*
WP_000979661.1	1.12952	0.60809	*O3K_14420*
WP_000110948.1	1.16084	0.41669	*oppD*
WP_000994906.1	1.08554	0.48035	*oppF*
WP_000214516.1	1.16786	0.61132	*cls*
WP_001309467.1	0.8516	0.42725	*DXE50_00840*
WP_001295624.1	1.31132	0.36638	*A1UI_01400*
WP_000967595.1	0.8329	0.29051	*DXE50_00850*
WP_000108160.1	0.88021	0.45887	*yciA*
WP_000028545.1	0.78077	0.22732	*yciC*
WP_000737226.1	1.09286	0.45651	*ompW*
WP_001079505.1	1.48223	0.09415	*A1UI_01407*
WP_000443067.1	1.15984	0.4195	*trpA*
WP_000209520.1	1.12136	0.53893	*trpB*
WP_000763511.1	0.91213	0.64046	*trpD*
WP_001194582.1	1.49261	0.08843	*A1UI_01414*
WP_001295575.1	1.37205	0.07711	*DXE50_17760*
WP_001291217.1	0.99475	0.93222	*WG3_01862*
WP_001278906.1	0.84014	0.31371	*HMPREF1591_00308*
WP_000559286.1	0.96076	0.74524	*KXD89_12335*
WP_000422045.1	0.99473	0.96776	*sohB*
WP_001031530.1	1.02748	0.90874	*DXE50_17730*
WP_001297122.1	1.04479	0.6282	*topA*
WP_000776253.1	0.92983	0.66959	*cysB*
WP_000099534.1	1.33864	0.05503	*acnA*
WP_001176295.1	0.87123	0.31731	*ribA*
WP_000876286.1	0.76527	0.19267	*lapA*
WP_000891353.1	0.93791	0.73809	*lapB*
WP_000176270.1	0.87706	0.43438	*pyrF*
WP_001295580.1	0.96668	0.8382	*ECMP0215528_1563*

WP_001088620.1	0.99243	0.95719	*KXD89_12255*
WP_000484984.1	0.94914	0.91586	*rnb*
WP_000506490.1	0.92278	0.72876	*fabI*
WP_001146163.1	0.97332	0.87445	*sapC*
WP_001250216.1	0.9719	0.86901	*O3K_13860*
WP_001296746.1	0.86463	0.47053	*puuA*
WP_001300506.1	1.30699	0.25583	*A1UI_01451*
WP_001009090.1	0.87511	0.50652	*A1UI_01453*
WP_000069229.1	0.92368	0.6839	*EL76_2650*
WP_001301108.1	0.94807	0.77708	*pspF*
WP_000511025.1	1.173	0.24843	*pspA*
WP_000907387.1	0.96914	0.85833	*pspC*
WP_000473109.1	0.93582	0.61253	*pspE*
WP_000075375.1	0.91183	0.63934	*A1UI_01474*
WP_000825775.1	0.89645	0.58261	*A1UI_01475*
WP_000138728.1	1.46718	0.19836	*ycjF*
WP_001300658.1	1.14548	0.4617	*tyrR*
WP_000084387.1	0.91692	0.68823	*tpx*
WP_001261211.1	1.10333	0.60182	*A1UI_01479*
WP_000219122.1	0.95439	0.80142	*mpaA*
WP_001300523.1	0.98617	0.92387	*A1UI_01482*
WP_000683020.1	1.01819	0.95002	*mppA*
WP_000559900.1	1.3276	0.34448	*ynaI*
WP_001262123.1	1.27742	0.09941	*uspE*
WP_000611911.1	0.77993	0.07775	*fnr*
WP_000945011.1	0.8847	0.54027	*DXE50_17365*
WP_000444929.1	0.71002	0.09704	*abgA*
WP_001046829.1	1.2152	0.51978	*smrA*
WP_000387388.1	1.07318	0.82597	*zntB*
WP_000123737.1	0.98579	0.92241	*dbpA*
WP_001157406.1	0.8635	0.28847	*ttcA*
WP_000948459.1	0.95835	0.81673	*C4A13_02150*
WP_001300461.1	1.20878	0.2967	*A1UI_01500*
WP_000628244.1	0.93072	0.58634	*nifJ*
WP_000762236.1	1.12099	0.37256	*HMPREF1591_00398*
WP_000698145.1	0.9474	0.77451	*EKN05_007005*
WP_000039887.1	1.07984	0.80959	*A1UI_01524*
WP_001123464.1	1.38635	0.27439	*EL76_2579*
WP_000097801.1	1.24619	0.35093	*O3K_13465*
WP_000048950.1	0.77532	0.14232	*azoR*
WP_000139543.1	1.02189	0.93352	*hrpA*
WP_001027942.1	1.0885	0.65673	*HGD79_04235*
WP_000115943.1	1.11616	0.38609	*aldA*
WP_000428998.1	1.27057	0.42625	*cybB*
WP_000414567.1	0.76413	0.19026	*KXD89_11535*
WP_000013789.1	1.29571	0.27164	*KXD89_11530*
WP_000375961.1	1.02853	0.91035	*opgD*
WP_001296778.1	1.04301	0.84119	*DXE50_16735*

WP_000140873.1	1.10872	0.74066	*rimL*
WP_000586732.1	1.12798	0.61217	*O3K_13350*
WP_001261013.1	1.19961	0.31731	*DXE50_16710*
WP_001303492.1	0.91172	0.58967	*CSW52_29105*
WP_000047424.1	1.29599	0.14938	*A1UI_01562*
WP_000531462.1	1.35975	0.08611	*CSW52_29020*
WP_000689363.1	0.7665	0.12535	*KXD89_11385*
WP_000550695.1	0.79397	0.10084	*KXD89_11380*
WP_001120143.1	0.801	0.27769	*pptA*
WP_010723100.1	1.23982	0.23466	*fdnG*
WP_001240582.1	1.42264	0.23755	*fdxH*
WP_000045648.1	1.55727	0.05944	*fdnI*
WP_000152305.1	1.34045	0.10214	*osmC*
WP_000830550.1	0.84815	0.34052	*EL76_2497*
WP_000350395.1	0.87273	0.49825	*EKN05_007410*
WP_001244968.1	0.99212	0.95575	*KXD89_11170*
WP_000832437.1	1.07902	0.81158	*KXD89_11165*
WP_001125439.1	0.92103	0.67387	*A1UI_01641*
WP_000154342.1	1.58929	0.11903	*lsrR*
WP_000172465.1	1.3343	0.2207	*lsrB*
WP_000774165.1	1.21046	0.29304	*lsrF*
WP_000558527.1	1.34641	0.32055	*lsrG*
WP_001286597.1	1.41424	0.14068	*tam*
WP_000156615.1	1.0954	0.772	*sad*
WP_000210799.1	0.84195	0.39635	*ydeA*
WP_000671737.1	1.29327	0.27517	*A311_02188*
WP_000210373.1	1.0102	0.77227	*dcp*
WP_000636571.1	0.97181	0.94803	*ydfG*
WP_000215549.1	1.11046	0.73662	*DXE50_16075*
WP_001019606.1	0.85056	0.24374	*WG3_02248*
WP_000534858.1	0.81405	0.31317	*relB*
WP_000448564.1	1.28009	0.41159	*WG3_02272*
WP_001295394.1	1.49196	0.17915	*rspA*
WP_001340362.1	1.0814	0.80576	*KXD89_10665*
WP_000148710.1	1.0834	0.73789	*dmsD*
WP_000919231.1	1.45354	0.20969	*bioD*
WP_000225262.1	0.99102	0.94244	*mlc*
WP_000014036.1	1.0156	0.96158	*pntB*
WP_001300486.1	1.02805	0.90621	*A1UI_01794*
WP_000769303.1	1.04693	0.6198	*KXD89_10570*
WP_000520804.1	1.05582	0.86954	*folM*
WP_000135181.1	1.34011	0.32841	*tus*
WP_001099085.1	1.04277	0.63616	*fumC*
WP_000066639.1	0.93972	0.84848	*DXE50_15495*
WP_001170664.1	0.87534	0.42458	*A1UI_01804*
WP_000945878.1	1.01378	0.95941	
WP_000969092.1	1.67658	0.08165	*DXE50_15465*
WP_000125583.1	0.83075	0.3618	*malX*

WP_000567490.1	0.85329	0.30918	*add*
WP_001282550.1	0.94237	0.72632	*KXD89_10470*
WP_000217950.1	0.83541	0.29843	*ydgT*
WP_000991805.1	0.82192	0.33565	*rsxB*
WP_000915778.1	1.09829	0.76515	*rsxC*
WP_000920784.1	1.18974	0.56768	*rsxG*
WP_000100932.1	0.78608	0.16504	*dtpA*
WP_000765749.1	1.21877	0.1689	*gstA*
WP_001307229.1	1.02102	0.93735	*pdxY*
WP_001295400.1	1.07831	0.50475	*tyrS*
WP_001282319.1	1.13502	0.49421	*pdxH*
WP_000835039.1	1.27406	0.30428	*anmK*
WP_001296937.1	1.25513	0.33538	*sodC*
WP_000250656.1	1.07163	0.72251	*DXE50_15315*
WP_000093589.1	0.94541	0.66275	*nemA*
WP_001237796.1	0.90862	0.47768	*gloA*
WP_001282283.1	0.98859	0.93953	*rnt*
WP_000108172.1	0.77429	0.06452	*DXE50_15280*
WP_000007283.1	1.05264	0.59779	*sodB*
WP_000190982.1	1.06712	0.74062	*purR*
WP_000098896.1	1.27481	0.17781	*DXE50_15240*
WP_000493947.1	1.05324	0.79781	*ribE*
WP_001174940.1	1.27293	0.42258	*mdtK*
WP_000534271.1	0.98002	0.85091	*KXD89_10260*
WP_000212657.1	1.13182	0.60202	*EKN05_007655*
WP_000716950.1	1.35804	0.30647	*KXD89_10240*
WP_000528342.1	1.15237	0.64361	*fumD*
WP_001295403.1	0.99749	0.82888	*DXE50_15165*
WP_000648420.1	0.83847	0.24326	*lpp*
WP_000817105.1	1.55418	0.06059	*ldtE*
WP_001196530.1	1.04496	0.89736	*sufE*
WP_000907979.1	1.35152	0.3143	*sufD*
WP_000948863.1	1.51312	0.16411	*sufC*
WP_000089364.1	1.01095	0.98711	*sufB*
WP_000367160.1	1.41625	0.13904	*sufA*
WP_001296104.1	1.13743	0.67583	*DXE50_15120*
WP_000637982.1	0.90944	0.63043	*ydiI*
WP_000613008.1	1.0556	0.82305	*KXD89_10145*
WP_000248636.1	0.97269	0.87203	*ydiK*
WP_000860201.1	0.98371	0.91698	*aroD*
WP_000368046.1	0.90964	0.4825	*ppsR*
WP_001082229.1	1.02791	0.90684	*aroH*
WP_001229265.1	1.01487	0.75194	*ihfA*
WP_000672380.1	0.99358	0.84666	*pheT*
WP_000018596.1	0.96551	0.97804	*pheS*
pdb|6H58|QQ	0.86159	0.35025	-
pdb|6H58|33	1.31814	0.06735	-
WP_001700733.1	1.12747	0.35494	*infC*

WP_001144202.1	0.94079	0.85613	*thrS*
WP_000251727.1	1.35172	0.09249	*EL76_2143*
WP_000146159.1	1.54989	0.06223	*ghoS*
WP_000106833.1	0.8861	0.37712	*A1UI_01927*
WP_001010696.1	1.02013	0.93817	*A1UI_01929*
WP_000412169.1	1.11454	0.72723	*chbB*
WP_001039044.1	1.15999	0.27588	*osmE*
WP_000175026.1	1.04662	0.62104	*nadE*
WP_001228984.1	0.89819	0.42969	*A1UI_01943*
WP_000368506.1	0.96843	0.85562	*astE*
WP_000177206.1	1.38023	0.17091	*astD*
WP_000673918.1	0.91575	0.51177	*xth*
WP_000939212.1	1.41773	0.13786	*A1UI_01951*
WP_001215339.1	1.44566	0.21651	*A1UI_01954*
WP_001350515.1	1.24275	0.35707	*ynjE*
WP_000373021.1	1.00708	1	*gdhA*
WP_001235800.1	1.05452	0.87283	*topB*
WP_001295485.1	1.02626	0.91412	*selD*
WP_000339283.1	1.11925	0.54607	*O3K_11135*
WP_001259810.1	1.05252	0.80085	*sppA*
WP_001170162.1	0.97494	0.87642	*DXE50_14700*
WP_001135066.1	1.07654	0.81768	*KXD89_09735*
WP_000719096.1	0.8888	0.49328	*HMPREF1591_01708*
WP_000645221.1	0.78431	0.23574	*KXD89_09695*
WP_001300415.1	1.03745	0.88117	*KXD89_09690*
WP_001284618.1	1.11407	0.56386	*msrB*
WP_000153502.1	0.87617	0.42929	*gapA*
WP_001335909.1	0.83791	0.20439	*A1UI_01981*
WP_000163771.1	0.97313	0.94177	*DXE50_14620*
WP_000219687.1	1.4224	0.23778	*yeaH*
WP_000766132.1	0.96344	0.83635	*O3K_11030*
WP_000138043.1	1.02932	0.90776	*A311_02408*
WP_001326526.1	1.49173	0.0889	*KXD89_09615*
WP_000691930.1	0.86522	0.4725	*ECMP0215528_2082*
WP_000939317.1	0.96462	0.8409	*yeaR*
WP_000758422.1	1.11204	0.5709	*fadD*
WP_000290576.1	1.13824	0.58532	*yeaY*
WP_001220966.1	0.79285	0.18051	*A1UI_02007*
WP_001295493.1	1.03046	0.89562	*DXE50_14475*
WP_001111995.1	1.22272	0.50624	*AC28_1962*
WP_000457334.1	1.34905	0.31731	*yoaH*
WP_000854958.1	0.9631	0.75795	*pabB*
WP_000456715.1	1.23719	0.48091	*nudL*
WP_000624298.1	1.13593	0.33287	*DXE50_14450*
WP_000150543.1	0.82754	0.2008	*manX*
WP_000406926.1	0.94042	0.71742	*DXE50_14430*
WP_000228655.1	0.76618	0.0592	*DXE50_14425*
WP_000156255.1	0.92785	0.69971	*yobD*

WP_001062678.1	1.0652	0.55102	*HMPREF0880_03203*
WP_001006866.1	0.98197	0.90776	*ECMP0215528_2119*
WP_001211011.1	1.18649	0.57402	*DXE50_14385*
WP_001262203.1	0.92063	0.53568	*kdgR*
WP_000984520.1	0.8267	0.17318	*htpX*
WP_001055791.1	1.06503	0.55165	*DXE50_14365*
WP_000431381.1	0.86862	0.3873	*proQ*
WP_001043877.1	0.92829	0.7674	*msrC*
WP_001326728.1	1.16706	0.51409	*yebT*
WP_001352260.1	1.14973	0.55614	*rsmF*
WP_001295499.1	1.60824	0.10976	*DXE50_14335*
WP_000976476.1	0.89048	0.50013	*EKN05_006800*
WP_000168747.1	1.04002	0.87281	*yobA*
WP_000944256.1	0.75501	0.17164	*exoX*
WP_000024757.1	1.49599	0.08664	*KXD89_09330*
WP_000257738.1	0.72033	0.11166	*yebG*
WP_000173484.1	1.06473	0.79457	*purT*
WP_000800512.1	0.90473	0.60594	*DXE50_14265*
WP_001069467.1	0.87316	0.32476	*edd*
WP_000301727.1	0.91147	0.65106	*zwf*
WP_001056706.1	0.93949	0.7132	*O3K_10665*
WP_000091148.1	1.14216	0.31731	*DXE50_14245*
WP_000448381.1	0.95637	0.79053	*msbB*
WP_001300644.1	0.7926	0.09839	*znuA*
WP_000202996.1	1.02201	0.95752	*znuC*
WP_000568519.1	1.19071	0.46038	*ruvB*
WP_000580323.1	1.01784	0.96863	*ruvA*
WP_000907248.1	1.02207	0.93269	*yebC*
WP_001258678.1	1.03993	0.64748	*aspS*
WP_000639274.1	1.40903	0.25083	*yecE*
WP_000019590.1	0.88012	0.35241	*cmoA*
WP_000564725.1	0.81439	0.14276	*cmoB*
WP_001185741.1	1.00617	1	*cutC*
WP_001326725.1	1.57063	0.12887	*GKR75_01495*
WP_001025322.1	0.91809	0.69628	*argS*
WP_000066951.1	0.78534	0.08612	*flhA*
WP_001295647.1	0.70971	0.09661	*flhD*
WP_000122416.1	0.8652	0.47245	*uspC*
WP_001295646.1	1.44509	0.11747	*otsA*
WP_001295645.1	1.05484	0.82547	*A1UI_02107*
WP_000100205.1	1.00875	0.99247	*EKN05_006250*
WP_001187819.1	0.97484	0.93371	*DXE50_13950*
WP_000548675.1	0.86835	0.38582	*DXE50_13945*
WP_000082127.1	0.91158	0.58906	*DXE50_13935*
WP_000917208.1	1.33276	0.05833	*ftnA*
WP_000797560.1	0.80035	0.276	*tyrP*
WP_000847902.1	1.09312	0.70928	*yecA*
WP_001160187.1	1.1594	0.62881	*pgsA*

WP_001283421.1	1.04552	0.85511	*uvrC*
WP_000611335.1	0.89422	0.51549	*uvrY*
WP_000106474.1	1.1275	0.61345	*EKN05_006160*
WP_001272991.1	1.06012	0.85863	*yecC*
WP_001158220.1	1.21293	0.52392	*tcyL*
WP_001128215.1	1.06113	0.76508	*dcyD*
WP_001296168.1	1.12844	0.35235	*HMPREF1604_01381*
WP_000079739.1	0.92585	0.6921	*fliC*
WP_001350519.1	0.98874	0.89906	*KXD89_08915*
WP_000118897.1	0.96698	0.83961	*HMPREF1595_04608*
WP_000790504.1	0.7604	0.11446	*yedF*
WP_000922686.1	1.34887	0.31753	*KXD89_08780*
WP_001157239.1	0.83217	0.2882	*G711_02468*
WP_000218212.1	1.19218	0.21201	*hchA*
WP_000920120.1	1.01511	0.97592	*HMPREF1611_00004*
WP_001007805.1	0.69713	0.0807	*HMPREF1589_00386*
WP_001302302.1	0.90087	0.54327	*mtfA*
WP_001060244.1	1.05235	0.80156	*amn*
WP_000532923.1	0.81574	0.14591	*yeeN*
WP_001011008.1	0.83732	0.38191	*HMPREF1602_03546*
AIN32411.1	1.55139	0.13979	-
WP_001166160.1	1.14662	0.65589	*cobT*
WP_001326708.1	0.92177	0.67667	*cobS*
WP_000450409.1	0.99509	0.83975	*yeeX*
WP_001105415.1	1.26595	0.31731	*sbmC*
WP_000980589.1	0.87081	0.42242	*sbcB*
WP_000234896.1	0.87337	0.43224	*FSF13_012590*
WP_000019197.1	0.83458	0.37345	*plaP*
WP_000754737.1	1.07517	0.70841	*yeeZ*
WP_000131782.1	0.91784	0.52196	*hisG*
WP_000009594.1	0.90289	0.45104	*hisD*
WP_000108941.1	0.94242	0.64697	*hisC*
WP_000080105.1	0.93205	0.59312	*hisB*
WP_001103560.1	1.01621	0.95128	*hisH*
WP_000586462.1	0.89689	0.5266	*hisA*
WP_000880182.1	1.07384	0.71371	*hisF*
WP_000954911.1	0.92288	0.6386	*hisI*
WP_001393575.1	0.87121	0.40144	*G736_02383*
WP_000043484.1	0.89891	0.56794	*G736_02385*
WP_000520320.1	0.99207	0.91746	*KXD89_08400*
WP_000601187.1	0.96935	0.85054	*G736_02388*
WP_001407542.1	0.90456	0.45873	*G736_02389*
WP_000272486.1	0.80093	0.11397	*G736_02391*
WP_001100981.1	0.79734	0.19127	*G736_02393*
WP_000783975.1	0.99324	0.9239	*G736_02394*
WP_001023610.1	0.90633	0.56643	*G736_02395*
WP_000699460.1	0.85513	0.31803	*rfbB*
WP_000183060.1	0.85945	0.33937	*galF*

WP_000454701.1	0.97529	0.88206	*EKN05_005265*
WP_001252331.1	1.09019	0.71784	*ECMP0215528_2416*
WP_001234768.1	1.14193	0.47256	*dcd*
WP_001295424.1	0.96962	0.79361	*udk*
WP_000288420.1	1.20201	0.43624	*alkA*
WP_000469708.1	0.92492	0.55701	*yegD*
WP_000678989.1	0.99669	0.96408	*mdtA*
WP_000137877.1	1.1626	0.52469	*baeR*
WP_000476011.1	0.94256	0.6477	*ECMP0215528_2437*
WP_000844219.1	0.76809	0.06154	*A1UI_02300*
WP_000823270.1	1.09506	0.44951	*A1UI_02302*
WP_000853883.1	0.86227	0.35376	*gatZ*
WP_001307281.1	0.98345	0.89334	*gatY*
WP_000129551.1	1.1765	0.24143	*fbaB*
WP_000846217.1	1.40244	0.15058	*ECMP0215528_2449*
WP_000434038.1	0.99119	0.94309	*DXE50_12360*
WP_001005448.1	0.93436	0.81031	*apbC*
WP_001350533.1	0.89745	0.55854	*metG*
WP_000950409.1	1.01306	0.96185	*DXE50_12250*
WP_000598641.1	1.11994	0.6338	*yehT*
WP_001295431.1	1.36197	0.30183	*btsS*
WP_000569315.1	1.05652	0.86775	*yehX*
WP_001130308.1	1.13327	0.49981	*osmF*
WP_000871504.1	1.03223	0.88789	*bglX*
WP_000097403.1	0.98824	0.87114	*dld*
WP_000079538.1	1.66843	0.0846	*DXE50_11940*
WP_001264861.1	1.22307	0.5056	*dusC*
WP_000553555.1	0.8985	0.59008	*cdd*
WP_001136389.1	1.22945	0.49433	*A1UI_02361*
WP_001275118.1	0.72315	0.06204	*mglC*
WP_000255039.1	0.93751	0.83277	*A1UI_02364*
WP_001036964.1	0.9365	0.82552	*mglB*
WP_000628648.1	0.89936	0.59322	*galS*
WP_001139613.1	0.96036	0.99599	*folE*
WP_000425438.1	1.14694	0.56315	*O3K_08675*
WP_000489247.1	0.76181	0.05408	*O3K_08670*
WP_000253273.1	1.14713	0.6548	*lysP*
WP_000548294.1	0.80366	0.20707	*DXE50_11840*
WP_000182053.1	1.00928	0.99159	*DXE50_11835*
WP_000873894.1	1.05498	0.79053	*nfo*
WP_001292460.1	1.21944	0.5121	*psuG*
WP_000854447.1	1.12092	0.54042	*fruA*
WP_000091263.1	0.9087	0.47807	*fruK*
WP_000487246.1	0.87733	0.34117	*HMPREF1589_01474*
WP_001136827.1	0.81447	0.15691	*yeiP*
WP_001091940.1	0.87168	0.49462	*KXD89_07695*
WP_000198828.1	1.06674	0.78835	*yeiR*
WP_000470560.1	1.10437	0.75081	*A1UI_02392*

WP_000637046.1	0.99503	0.95775	*KXD89_07670*
WP_000194927.1	1.14992	0.64883	*A1UI_02396*
WP_001234850.1	1.08174	0.6827	*rsuA*
WP_000494183.1	0.9618	0.99581	*rplY*
WP_000050793.1	0.96183	0.99564	*yejK*
WP_001135667.1	1.16975	0.25507	*yejL*
WP_000256203.1	1.6667	0.67526	*yejM*
WP_001113639.1	0.9767	0.88458	*narP*
WP_001026418.1	0.983	0.91169	*ccmE*
WP_000778061.1	1.33821	0.21601	*napA*
WP_000849209.1	0.87495	0.33174	*eco*
WP_000758077.1	0.75345	0.16857	*mqo*
WP_000865568.1	0.9691	0.96088	*ompC*
WP_001249081.1	1.02776	0.90751	*rcsD*
WP_001061917.1	1.08843	0.47086	*rcsB*
WP_000876011.1	1.31518	0.36109	*rcsC*
WP_000125282.1	1.6712	0.08359	*A1UI_02435*
WP_000012305.1	0.94103	0.75002	*ECMP0215528_2584*
WP_001281242.1	0.94827	0.90967	*gyrA*
WP_000990765.1	0.92177	0.54128	*ubiG*
WP_001075164.1	0.92497	0.74408	*ECMP0215528_2589*
WP_000332037.1	0.96996	0.9568	*DXE50_11490*
WP_000779105.1	0.91747	0.69199	*glpQ*
WP_000948731.1	0.82277	0.18394	*glpT*
WP_000857257.1	1.01912	0.94584	*glpA*
WP_001209927.1	0.95475	0.71268	*glpB*
WP_001000379.1	0.91998	0.62576	*A1UI_02458*
WP_000921621.1	1.07118	0.77473	*A1UI_02465*
WP_001300392.1	1.26006	0.32704	*A1UI_02466*
WP_000577625.1	1.51303	0.16417	*menE*
WP_001255628.1	1.0908	0.71603	*menC*
WP_000639996.1	1.1298	0.34876	*menB*
WP_001295284.1	0.96612	0.84668	*menD*
WP_001191419.1	0.74144	0.08488	*menF*
WP_000070621.1	1.03499	0.66738	*elaB*
WP_000574091.1	1.39616	0.1561	*EKN05_004155*
WP_001300687.1	1.11197	0.65568	*rbn*
WP_000156701.1	0.87505	0.43869	*nuoN*
WP_001056643.1	0.96229	0.8179	*nuoL*
WP_000612644.1	1.01975	0.96354	*nuoK*
WP_000172749.1	0.93321	0.59906	*nuoI*
WP_000118507.1	0.83169	0.2867	*nuoH*
WP_000190939.1	0.901	0.58148	*A1UI_02496*
WP_000789500.1	0.94706	0.90098	*WG3_01307*
WP_000545042.1	0.85706	0.26563	*nuoE*
AIN32696.1	0.95838	0.9819	-
WP_000386733.1	0.95503	0.71418	*nuoB*
WP_000062997.1	0.86386	0.39638	*nuoA*

WP_000622280.1	0.75267	0.16705	*lrhA*
WP_000074527.1	0.92248	0.54482	*DXE50_11220*
WP_000813859.1	1.05261	0.83252	*yfbR*
WP_001203392.1	1.18082	0.36296	*A1UI_02507*
WP_000426124.1	0.95567	0.96257	*yfbU*
WP_000095707.1	1.07318	0.52252	*ackA*
WP_000086722.1	1.04171	0.64038	*EKN05_004025*
WP_000437935.1	0.98594	0.92729	*yfcD*
WP_000772452.1	1.27448	0.30362	*DXE50_11170*
WP_000042442.1	1.23341	0.37414	*O3K_07990*
WP_000068457.1	0.79027	0.09432	*folX*
WP_001028341.1	1.31272	0.24809	*A1UI_02518*
WP_000737621.1	0.87205	0.32046	*hisJ*
WP_000334220.1	1.13007	0.60664	*purF*
WP_000146992.1	0.8793	0.52115	*dedD*
WP_000584546.1	0.89839	0.43061	*A1UI_02530*
WP_000118404.1	0.93667	0.82679	*accD*
WP_001283586.1	0.82385	0.34131	*truA*
WP_001289167.1	0.89327	0.4079	*A1UI_02534*
WP_000699148.1	1.01659	0.74451	*pdxB*
WP_000615834.1	1.28516	0.40396	*A1UI_02536*
WP_000817178.1	0.83789	0.24084	*fabB*
WP_000683799.1	1.03896	0.85861	*mnmC*
WP_000559764.1	0.87073	0.42213	*DXE50_11045*
WP_001043825.1	1.00321	0.9888	*mepA*
WP_001333535.1	1.0499	0.81187	*aroC*
WP_001300582.1	1.06643	0.74345	*prmB*
WP_000730806.1	1.12641	0.70027	*smrB*
WP_001195819.1	0.74198	0.14711	*sixA*
WP_000426176.1	1.09485	0.70426	*fadJ*
WP_000531952.1	1.13177	0.60217	*fadI*
WP_001296261.1	1.26369	0.19448	*WG3_01240*
WP_001295701.1	1.11543	0.38817	*AC28_2640*
WP_203277081.1	1.03011	0.89716	-
WP_000958671.1	0.93925	0.74321	*G925_02382*
WP_000703651.1	1.03725	0.8818	*KY274_06780*
WP_001030215.1	0.99925	0.98848	*KXD89_06785*
WP_000556048.1	0.84898	0.41876	
WP_000785931.1	1.03691	0.88291	*DXE50_10435*
WP_001295458.1	1.0018	0.98347	*DXE50_10425*
WP_000955906.1	0.74474	0.15209	*A1UI_02584*
WP_000170346.1	1.26782	0.10869	*glk*
WP_000376337.1	0.95579	0.7183	*nupC*
AIN32800.1	0.82082	0.33247	-
WP_000695655.1	0.94497	0.88604	*gltX*
WP_000443661.1	1.12008	0.54326	*ligA*
WP_001300494.1	0.9852	0.87953	*zipA*
WP_000254839.1	0.87679	0.51237	*cysZ*

WP_000034402.1	1.18953	0.21676	*cysK*
WP_000487600.1	0.93828	0.83824	*HMPREF0454_04299*
WP_000623140.1	0.88673	0.49155	*ptsI*
WP_000522247.1	1.092	0.45926	*DXE50_10250*
WP_000096674.1	0.78696	0.16702	*pdxK*
WP_001336044.1	1.05944	0.811	*cysM*
WP_000290230.1	0.92427	0.55375	*G711_02922*
WP_000517431.1	0.90302	0.45162	*ucpA*
WP_000966470.1	0.83993	0.39001	*murR*
WP_001159160.1	1.02985	0.90599	*murQ*
WP_001327042.1	1.14866	0.65151	*pbp4b*
WP_001350538.1	0.98689	0.88883	*yfeX*
WP_000838944.1	0.9407	0.71873	*EKN05_003410*
WP_000405996.1	1.12742	0.69801	*ypeA*
WP_000102886.1	1.42065	0.23945	*amiA*
WP_000801365.1	1.06614	0.84349	*hemF*
WP_001111023.1	1.16649	0.61414	*A1UI_02631*
WP_000372316.1	1.0459	0.89493	*eutC*
WP_000769961.1	1.05207	0.87908	*eutB*
WP_010723125.1	1.19146	0.45876	*yffS*
WP_000387713.1	0.79896	0.27235	*eutM*
WP_000342644.1	1.04063	0.64467	*maeB*
WP_000258236.1	1.55813	0.05912	*KXD89_06205*
WP_001277801.1	1.42778	0.13003	*dapE*
WP_000679799.1	1.17167	0.60356	*ypfH*
WP_001267498.1	0.98767	0.93526	*DXE50_09995*
WP_001295467.1	0.95145	0.9324	*purC*
WP_001297320.1	1.00867	0.77897	*bamC*
WP_001311023.1	0.9517	0.93415	*dapA*
WP_000176187.1	0.9853	0.88006	*EKN05_003185*
WP_001068682.1	0.81109	0.14676	*EKN05_003180*
WP_000489667.1	1.28526	0.28702	*bepA*
WP_000166446.1	1.00122	0.96785	*arsC*
WP_001307333.1	0.90707	0.62162	*hda*
WP_000198328.1	0.7067	0.09262	*uraA*
WP_001295473.1	0.77533	0.06619	*upp*
WP_001336050.1	1.00042	0.96347	*purM*
WP_001028612.1	0.85519	0.439	*purN*
WP_000529576.1	1.12344	0.53198	*ppk1*
WP_001121363.1	0.97549	0.82592	*O3K_06910*
WP_000076001.1	1.23994	0.47621	*O3K_06895*
WP_000138270.1	0.81645	0.16311	*guaA*
WP_001299507.1	0.88395	0.47478	*guaB*
WP_000937912.1	0.90682	0.56856	*xseA*
WP_001301158.1	1.02227	0.95683	*KXD89_06000*
WP_000249410.1	1.06411	0.75287	*engA*
WP_001177052.1	1.09114	0.46203	*bamB*
WP_000409205.1	1.0171	0.95489	*DXE50_09780*

WP_001107167.1	0.95132	0.93145	*hisS*
WP_000551807.1	0.88355	0.47235	*ispG*
WP_001090866.1	1.13607	0.49087	*rodZ*
WP_000003317.1	0.81154	0.13628	*trmG/rlmN*
WP_000963837.1	1.10469	0.41972	*ndk*
WP_000736312.1	0.98936	0.90248	*yfhM*
WP_000108626.1	1.23555	0.24251	*sseA*
WP_001295479.1	0.99071	0.9493	*sseB*
WP_000523616.1	1.13995	0.58089	*iscX*
WP_001124469.1	1.43	0.12835	*fdx*
WP_001196613.1	1.35691	0.08833	*hscA*
WP_000384413.1	0.78642	0.16581	*hscB*
WP_000028953.1	0.94454	0.7362	*iscA*
WP_000331707.1	0.79767	0.11094	*iscU*
WP_001295373.1	0.94137	0.86032	*iscS*
WP_001241357.1	1.22067	0.27156	*iscR*
WP_000940019.1	0.97095	0.80094	*trmJ*
WP_000553451.1	1.07898	0.50247	*suhB*
WP_000982994.1	0.83756	0.38265	*csiE*
WP_000919159.1	0.97189	0.94767	*glyA*
WP_000717694.1	0.83192	0.18729	*glnB*
WP_001295369.1	1.1088	0.66452	*A1UI_02746*
WP_001215861.1	1.43173	0.22904	*qseG*
WP_000970102.1	1.03612	0.87093	*purL*
WP_001013779.1	0.95278	0.79524	*DXE50_09565*
WP_001297412.1	1.0606	0.76724	*pdxJ*
WP_000020749.1	0.85215	0.24899	*era*
WP_001068343.1	0.98012	0.85145	*rnc*
WP_000002541.1	1.03195	0.89912	*lepB*
WP_000790168.1	1.00091	0.8135	*lepA*
WP_000812053.1	1.05425	0.82733	*rseB*
WP_001168459.1	0.93867	0.70947	*rseA*
WP_001295364.1	1.52798	0.15424	*rpoE*
WP_001094491.1	1.04256	0.86461	*nadB*
WP_001295363.1	0.77599	0.21627	*trmN*
WP_000219203.1	1.04573	0.82956	*srmB*
WP_000627807.1	1.12902	0.35081	*grcA*
WP_001262716.1	1.12128	0.63017	*ung*
WP_000997403.1	0.90984	0.64005	*DXE50_09455*
WP_001098726.1	0.9575	0.79576	*trxC*
WP_001300438.1	0.70805	0.0944	*A1UI_02775*
WP_000083005.1	1.14827	0.5598	*pat*
WP_000949265.1	1.08856	0.65651	*pssA*
WP_000841103.1	1.16769	0.5126	*kgtP*
WP_001235102.1	1.03119	0.68293	*clpB*
WP_000040169.1	1.19316	0.45507	*pgeF*
WP_000079100.1	1.1375	0.48635	*rluD*
WP_000197686.1	0.96121	0.99864	*bamD*

WP_000200120.1	0.96535	0.77023	*A1UI_02786*
WP_000225229.1	1.01243	0.96396	*tyrA*
WP_001168037.1	0.92162	0.6761	*aroF*
pdb|6H58|PP	0.82254	0.18316	-
WP_000264777.1	1.1373	0.58773	*trmD*
WP_000043335.1	0.88733	0.3823	*rimM*
WP_000256450.1	0.98102	0.90466	*rpsP*
WP_000460035.1	0.89165	0.52183	*ffh*
WP_001393454.1	1.04405	0.6311	*grpE*
WP_001059169.1	1.07177	0.82946	*nadK*
WP_001117838.1	0.89785	0.5877	*DXE50_09280*
WP_001300750.1	1.06657	0.84242	*ratA*
WP_000162574.1	1.06712	0.78717	*smpB*
WP_000101723.1	0.93909	0.7426	*KXD89_05435*
WP_000155570.1	0.97919	0.89708	*rnlA*
WP_000993126.1	1.16669	0.40038	*csiD*
WP_000772831.1	1.30591	0.07583	*gabD*
WP_001087611.1	1.30406	0.07719	*gabT*
WP_001229442.1	1.13745	0.67578	*A1UI_02878*
WP_000115383.1	1.03327	0.88333	*stpA*
WP_001295174.1	1.14049	0.57952	*DXE50_18090*
WP_000985494.1	0.97507	0.93261	*proV*
WP_000774988.1	0.99532	0.93538	*proW*
WP_001216525.1	0.90089	0.58076	*DXE50_18140*
WP_000378442.1	0.9925	0.91983	*emrR*
WP_001326681.1	0.94004	0.71571	*emrA*
WP_001130211.1	1.32891	0.06059	*luxS*
WP_000611804.1	1.03727	0.86594	*gshA*
WP_000273290.1	1.08776	0.79032	*A1UI_02899*
WP_000906486.1	1.13704	0.48782	*csrA*
WP_000047184.1	0.95644	0.96805	*alaS*
WP_000963143.1	1.2193	0.16814	*recA*
WP_000132231.1	0.99248	0.9197	*pncC*
WP_001295177.1	1.11995	0.63377	*mltB*
WP_000148878.1	0.75692	0.10856	*srlE*
WP_000216194.1	0.76981	0.06372	*A1UI_02912*
WP_001077358.1	0.79681	0.10605	*srlD*
WP_000804550.1	0.88323	0.47084	*srlR*
WP_001287420.1	1.09011	0.65065	*WG3_03337*
WP_001394680.1	1.34031	0.32815	*ascG*
WP_000337665.1	1.17646	0.59389	*hypB*
WP_001212985.1	1.44872	0.21384	*hypD*
WP_001272928.1	0.96503	0.76848	*mutS*
WP_001272592.1	1.39434	0.06291	*nlpD*
WP_000254708.1	1.2972	0.3863	*pcm*
WP_001295182.1	0.95063	0.76412	*surE*
WP_000568943.1	0.92631	0.65384	*truD*
WP_001219242.1	0.91311	0.49903	*ispF*

WP_000246138.1	0.92314	0.68185	*ispD*
WP_000517476.1	0.9756	0.88324	*ftsB*
WP_001173673.1	1.19632	0.555	*cysC*
WP_001090361.1	1.04254	0.8432	*cysN*
WP_000372108.1	0.92547	0.65009	*cysD*
WP_000039850.1	1.04551	0.85515	*cysH*
WP_001290679.1	1.0656	0.7468	*cysI*
WP_000211913.1	1.07105	0.7248	*cysJ*
WP_000108294.1	0.95664	0.7918	*queD*
WP_000036723.1	0.85492	0.31701	*eno*
WP_000210878.1	0.89381	0.53541	*pyrG*
WP_001071648.1	1.34612	0.32091	*mazG*
WP_000254738.1	0.76739	0.1972	*A1UI_02996*
WP_000226815.1	1.02108	0.93501	*DXE50_18695*
WP_000046812.1	1.05836	0.86307	*rumA*
WP_000186450.1	1.11055	0.73642	*barA*
WP_000098255.1	1.28276	0.29079	*gudD*
WP_000807753.1	1.07399	0.7131	*EKN05_002460*
WP_000206990.1	1.00779	0.9796	*ECMP0215528_3251*
WP_000342431.1	1.3304	0.34082	*syd*
WP_000100421.1	0.90839	0.5753	*queF*
WP_000627995.1	1.12159	0.37088	*ppnN*
WP_000450476.1	0.88077	0.35507	*DXE50_18755*
WP_000013588.1	1.18063	0.48275	*fucO*
WP_000724153.1	1.05581	0.82242	*fucI*
WP_000920840.1	1.24973	0.34471	*fucU*
WP_000642344.1	0.95771	0.81426	*fucR*
WP_001045520.1	0.98232	0.91059	*rlmM*
WP_000044401.1	0.99659	0.9637	*gcvA*
WP_000750398.1	0.83743	0.30489	*DXE50_18820*
WP_001300698.1	0.97257	0.87157	*O3K_05445*
WP_000184261.1	0.96752	0.84209	*A1UI_03026*
WP_000117728.1	0.89311	0.5109	*O3K_05435*
WP_000678646.1	0.95395	0.77938	*mltA*
WP_001138201.1	0.94143	0.72205	*ptrA*
WP_000946938.1	0.94018	0.7468	*recC*
WP_000816232.1	1.26197	0.32386	*thyA*
WP_000204658.1	0.96918	0.84976	*lgt*
WP_000957910.1	0.92602	0.56252	*EL76_1009*
WP_000564489.1	0.95422	0.8008	*rppH*
WP_001199295.1	1.15793	0.42496	*EKN05_001155*
WP_000899054.1	0.72085	0.05953	*aas*
WP_000201063.1	0.99256	0.94834	*A311_03510*
WP_001120711.1	0.71058	0.09779	*lysA*
WP_000848659.1	1.03134	0.89179	*ygeA*
WP_000256438.1	1.08056	0.49708	*DXE50_18990*
WP_000603502.1	1.12956	0.608	*HMPREF1620_04171*
WP_000383237.1	1.2067	0.53542	*kduI*

WP_000656030.1	0.79923	0.1959	*KXD89_04355*
WP_000065950.1	0.8269	0.272	*DXE50_19010*
WP_001301085.1	0.69628	0.0797	*ECMP0215528_3346*
WP_000417791.1	0.84713	0.41282	*A1UI_03071*
WP_000502395.1	1.15547	0.63706	*ygfK*
WP_001192820.1	1.30868	0.37003	*idi*
WP_000003071.1	0.96519	0.97954	*lysS*
WP_010723217.1	0.9896	0.86485	*prfB*
WP_000813200.1	0.96019	0.80819	*recJ*
WP_000715214.1	0.84558	0.22774	*dsbC*
WP_000806638.1	1.11667	0.72233	*xerD*
WP_001055874.1	0.83566	0.19787	*fldB*
WP_000354046.1	1.33009	0.22583	*sdhE*
WP_000886062.1	1.07418	0.51902	*ygfZ*
QRG61511.1	1.18797	0.34505	-
WP_000195062.1	0.79551	0.10584	*gcvP*
WP_000068701.1	0.77888	0.07218	*gcvT*
WP_001192229.1	1.114	0.65007	*ubiI*
WP_000111195.1	1.60437	0.11159	*ubiH*
WP_001290136.1	1.18123	0.3619	*pepP*
WP_001295378.1	0.86812	0.41225	*ygfB*
WP_001276008.1	1.02403	0.92523	*zapA*
WP_001151604.1	0.88417	0.47606	*DXE50_19325*
WP_000189743.1	0.94454	0.88297	*rpiA*
WP_000828351.1	0.99858	0.9854	*argP*
WP_000669834.1	1.07622	0.51194	*DXE50_19370*
WP_000389818.1	1.0984	0.43901	*mscS*
WP_000034372.1	0.92259	0.72742	*fbaA*
WP_000111269.1	0.91938	0.70521	*pgk*
WP_000218480.1	1.03837	0.86116	*epd*
WP_000098614.1	0.9174	0.69154	*ECMP0215528_3427*
WP_000105566.1	0.92297	0.73014	*speB*
WP_001300904.1	0.98091	0.90517	*speA*
WP_001062128.1	1.02225	0.72031	*metK*
WP_001222509.1	0.97499	0.82316	*rsmE*
WP_000593273.1	1.0052	0.79434	*gshB*
WP_001053178.1	0.98693	0.88908	*yqgE*
WP_000017106.1	1.02042	0.96174	*yqgF*
WP_000997795.1	0.85003	0.24199	*yggS*
WP_001094831.1	0.9601	0.74165	*yggT*
WP_001174777.1	0.91436	0.50507	*A1UI_03160*
WP_000239943.1	1.10151	0.75753	*A1UI_03161*
WP_000984796.1	1.06997	0.72915	*DXE50_19655*
WP_001107564.1	0.88735	0.49533	*DXE50_19660*
WP_000786911.1	1.07689	0.50962	*trmB*
WP_000091700.1	0.94561	0.89062	*yggX*
WP_000760323.1	1.29862	0.38426	*mltC*
WP_001049791.1	1.0832	0.80137	*nupG*

WP_001326492.1	0.85629	0.36895	*A1UI_03171*
WP_000084131.1	1.00433	0.79821	*glcB*
WP_000853256.1	1.01126	0.9811	*DXE50_19835*
WP_001297764.1	0.87408	0.50292	*ECMP0215528_3474*
WP_001297309.1	1.14967	0.4491	*EKN05_000500*
WP_001295515.1	1.40249	0.05834	*ECMP0215528_3491*
WP_001221939.1	1.54416	0.14411	*A1UI_03277*
WP_000083065.1	1.29023	0.27962	*hybC*
WP_000262172.1	1.14796	0.45421	*AB05_3609*
WP_000439331.1	1.14098	0.57826	*yqhA*
WP_000018760.1	1.19011	0.3398	*ECMP0215528_3505*
WP_001240712.1	0.75711	0.10887	*exbD*
WP_000527844.1	0.76883	0.06248	*DXE50_20010*
WP_001301079.1	1.54333	0.06482	*G894_03028*
WP_000268419.1	1.4819	0.18673	*DXE50_20020*
WP_001058802.1	0.95878	0.9847	*yqhD*
WP_000095187.1	0.92179	0.63376	*ygiQ*
WP_000059388.1	0.95324	0.77615	*ftsP*
WP_000965722.1	0.92072	0.62902	*A1UI_03298*
WP_001281881.1	0.96355	0.7604	*parC*
WP_001295629.1	1.23444	0.24459	*A1UI_03300*
WP_001221495.1	1.25505	0.33553	*qseB*
WP_000673402.1	1.11814	0.71898	*A1UI_03306*
WP_000065430.1	0.95276	0.70197	*mdaB*
WP_000958598.1	1.1047	0.59692	*DXE50_20115*
WP_000195296.1	0.94568	0.66419	*parE*
WP_000444747.1	1.00549	0.98738	*cpdA*
WP_000917117.1	0.93369	0.68693	*DXE50_20140*
WP_000831543.1	1.00905	0.97536	*ygiB*
WP_000442860.1	1.10978	0.57884	*DXE50_20155*
WP_001076997.1	1.25156	0.21411	*ribB*
WP_001295626.1	0.92386	0.55172	*ubiK*
WP_000350095.1	1.15819	0.63134	*glgS*
WP_000869178.1	1.03179	0.68047	*hldE*
WP_001301081.1	1.0432	0.84037	*glnE*
WP_000708487.1	1.0962	0.70034	*cca*
WP_001295541.1	0.93275	0.68268	*folB*
WP_001264352.1	0.88736	0.38245	*tsaD*
pdb|6H58|uu	0.79382	0.10198	-
WP_000918827.1	0.82238	0.33699	*dnaG*
WP_000437376.1	1.02152	0.72343	*rpoD*
WP_000228937.1	1.13654	0.58971	*mug*
WP_000018003.1	0.98247	0.91128	*EKN05_000130*
WP_000094721.1	1.60313	0.11219	*HMPREF1599_02928*
WP_001301395.1	1.18725	0.57253	*ygjG*
WP_000450594.1	0.81404	0.31316	*HMPREF1599_02930*
WP_000212475.1	1.17797	0.48879	*HMPREF1599_02931*
WP_000695487.1	0.96216	0.83142	*ygjK*

WP_000018695.1	0.97376	0.87098	*rlmG*
WP_000617698.1	0.98839	0.9386	*KXD89_03155*
WP_001098806.1	0.67892	0.06112	*ECMP0215528_3592*
WP_000211655.1	1.04169	0.90583	*sstT*
WP_001199390.1	1.60044	0.11349	*HMPREF1599_02950*
WP_000187442.1	1.07203	0.7209	*uxaC*
WP_000406488.1	0.91969	0.6245	*exuR*
WP_001295543.1	1.06115	0.85604	*DXE50_20445*
WP_000096091.1	1.54683	0.1425	*HMPREF1599_02960*
WP_000531213.1	1.37888	0.28255	*HMPREF1599_02962*
WP_001041009.1	1.14799	0.5605	*DXE50_20485*
WP_000622126.1	0.79908	0.27267	*A1UI_03392*
WP_000719990.1	0.96482	0.76734	*DXE50_20515*
WP_000861734.1	0.76558	0.12365	*O3K_03370*
WP_000107723.1	0.91052	0.63446	*tdcC*
WP_001307405.1	1.33437	0.3357	*O3K_03300*
WP_000072187.1	0.9107	0.63514	*DXE50_20600*
WP_001336162.1	1.31749	0.35795	*agaV*
WP_000809262.1	0.86695	0.30113	*rsmI*
WP_000249104.1	0.92075	0.53625	*lpoA*
WP_001158034.1	0.78199	0.15614	*diaA*
WP_000646033.1	1.08734	0.66115	*ECMP0215528_3660*
WP_000147574.1	1.36264	0.30105	*A1UI_03435*
WP_001300423.1	1.14433	0.66082	*A1UI_03436*
WP_000908554.1	1.0288	0.90293	*DXE50_20720*
WP_001295552.1	0.84861	0.41756	*ubiT*
WP_000130380.1	1.01555	0.95347	*O3K_03135*
WP_000224351.1	0.96026	0.82407	*DXE50_20745*
WP_001295553.1	0.9968	0.83199	*deaD*
WP_000802080.1	0.9786	0.89338	*nlpI*
WP_001295554.1	0.95436	0.95321	*pnp*
WP_000089698.1	0.86335	0.39453	*truB*
WP_001040205.1	1.00792	0.7823	*rbfA*
WP_000133044.1	1.03721	0.65839	*infB*
WP_001031057.1	0.95611	0.96571	*nusA*
WP_001300397.1	0.90407	0.4565	*rimP*
WP_000207680.1	1.33546	0.05679	*argG*
WP_001295556.1	0.94819	0.67753	*secG*
WP_000071134.1	0.92215	0.72436	*glmM*
WP_000764731.1	1.12615	0.52297	*folP*
WP_001107467.1	0.99334	0.84775	*ftsH*
WP_000145975.1	1.01285	0.96256	*rlmE*
WP_001054420.1	0.78554	0.16385	*yhbY*
WP_001148001.1	0.98007	0.90911	*greA*
WP_001212619.1	0.96331	0.8226	*WG3_03920*
WP_000673575.1	0.90028	0.43914	*cgtA*
pdb|6H58|WW	0.77099	0.05942	-
WP_000271401.1	0.9449	0.88549	*rplU*

WP_001047336.1	0.80322	0.11855	*ispB*
WP_000357259.1	0.9125	0.65801	*murA*
WP_000429656.1	0.73331	0.07408	*ibaG*
WP_000004488.1	1.21355	0.41257	*mlaB*
WP_000476487.1	1.16128	0.27304	*mlaC*
WP_001296448.1	1.23207	0.24908	*mlaD*
WP_000925795.1	1.20725	0.42536	*mlaE*
WP_000438245.1	1.53797	0.067	*mlaF*
WP_001295557.1	0.98132	0.85808	*kdsD*
WP_000030005.1	1.09739	0.62351	*kdsC*
WP_000030537.1	1.02787	0.91252	*lptC*
WP_000669785.1	0.99437	0.93014	*lptA*
WP_000224099.1	1.10111	0.60989	*lptB*
WP_000809057.1	1.13619	0.5906	*rpoN*
WP_001176599.1	1.39991	0.15278	*DXE50_20960*
WP_000183676.1	0.8531	0.25214	*ptsN*
WP_000243741.1	1.11752	0.55199	*rapZ*
WP_001300411.1	1.08893	0.46923	*elbB*
WP_000809774.1	0.96455	0.76589	*arcB*
WP_000054239.1	0.84213	0.21704	*nanE*
WP_000108454.1	0.73214	0.0726	*nanT*
WP_000523845.1	0.88501	0.47796	*nanR*
WP_000366129.1	1.04911	0.84363	*sspB*
WP_000257293.1	1.01306	0.75977	*sspA*
WP_000829818.1	0.87681	0.43293	*rpsI*
WP_000847559.1	0.89324	0.53181	*rplM*
WP_001192332.1	0.88128	0.4631	*zapE*
WP_001295270.1	0.94112	0.85852	*DXE50_21085*
WP_001295271.1	1.01302	0.9732	*degQ*
WP_000497723.1	1.18585	0.57526	*DXE50_21095*
WP_001295272.1	1.07112	0.52979	*mdh*
WP_001257846.1	0.89371	0.40985	*argR*
WP_000055909.1	0.97701	0.83431	*tldD*
WP_001253618.1	0.84257	0.39829	*yhdP*
WP_000123197.1	1.09228	0.64248	*DXE50_21150*
WP_000203105.1	1.13119	0.6037	*A1UI_03526*
WP_000802511.1	0.95799	0.79803	*WG3_03983*
WP_000913396.1	0.93526	0.8167	*mreB*
WP_001241469.1	1.20309	0.54217	*A1UI_03530*
WP_001148481.1	1.14902	0.30083	*EL76_0511*
WP_000884639.1	0.95213	0.93722	*accC*
WP_001175728.1	1.12438	0.70484	*panF*
WP_001145827.1	1.09616	0.62805	*prmA*
WP_000462905.1	1.05662	0.7837	*fis*
WP_001286216.1	1.18425	0.2265	*EKN05_022560*
WP_000451243.1	1.01544	0.95383	*aroE*
WP_001301412.1	0.84471	0.32886	*tsaC*
WP_001129722.1	0.9702	0.86245	*A1UI_03556*

WP_000114984.1	0.97423	0.81895	*def*
WP_000004473.1	1.01505	0.96406	*fmt*
WP_000744778.1	0.93813	0.62453	*rsmB*
WP_000691382.1	1.1033	0.68001	*trkA*
WP_000022442.1	1.16824	0.39613	*mscL*
WP_000285607.1	0.80975	0.30124	*zntR*
WP_001216368.1	1.02364	0.71443	*rplQ*
WP_001162094.1	0.99897	0.82219	*rpoA*
pdb|7OI0|D	0.97051	0.95421	-
pdb|6H58|kk	0.83955	0.24772	-
pdb|6H58|mm	0.92864	0.76983	-
WP_001118861.1	0.90465	0.45915	*secY*
pdb|6H58|LL	0.85285	0.30711	-
pdb|6H58|ZZ	0.78675	0.08691	-
pdb|7OE0|E	0.97401	0.9376	-
pdb|6H58|OO	0.92819	0.7667	-
pdb|6H58|GG	0.93849	0.83974	-
WP_000062611.1	0.84431	0.26806	*rpsH*
pdb|7OE0|N	0.88676	0.49171	-
WP_001096200.1	0.87431	0.41871	*rplE*
WP_000729185.1	1.00655	0.78835	*rplX*
WP_000613955.1	0.87788	0.43909	*rplN*
pdb|6H58|qq	0.89272	0.52854	-
WP_000644741.1	0.89748	0.55876	*rpmC*
WP_000941212.1	0.90928	0.63629	*rplP*
pdb|7OE0|C	1.00222	0.80759	-
WP_000447529.1	1.05715	0.58071	*rplV*
pdb|6H58|ss	0.92011	0.71023	-
pdb|6H58|CC	0.89626	0.55092	-
WP_000617544.1	0.80855	0.13941	*rplW*
WP_000424395.1	0.92512	0.74513	*rplD*
WP_000579833.1	0.92944	0.77552	*rplC*
WP_001181004.1	0.96326	0.98878	*rpsJ*
WP_000675504.1	1.22793	0.15588	*bfr*
WP_000031784.1	0.93475	0.81313	*tuf*
WP_000124700.1	0.90292	0.59404	*fusA*
pdb|7OE0|G	0.88097	0.45708	-
pdb|7OI0|L	0.87828	0.44138	-
WP_000903373.1	1.42639	0.234	*tusB*
WP_000820714.1	0.7291	0.12524	*tusC*
WP_000091466.1	1.02393	0.92445	*DXE50_21575*
WP_000838250.1	1.08962	0.46696	*A1UI_03620*
WP_001153615.1	0.87565	0.44104	*slyX*
WP_000861334.1	0.94707	0.90107	*DXE50_21590*
WP_001007730.1	1.38017	0.17097	*DXE50_21595*
WP_000634798.1	0.90114	0.44306	*DXE50_21610*
WP_000057356.1	0.84479	0.40534	*A1UI_03627*
WP_001274680.1	1.01082	0.98311	*DXE50_21625*

WP_001148908.1	1.08364	0.67535	*EKN05_022235*
WP_000242755.1	0.9654	0.97856	*GA0061070_104725*
WP_000963792.1	1.00139	0.99822	*argD*
WP_001280641.1	1.42287	0.23733	*DXE50_21655*
WP_000477225.1	1.24037	0.13957	*ppiA*
WP_000049208.1	1.55275	0.06114	*nirB*
WP_000084764.1	1.29082	0.39558	*nirD*
WP_000349855.1	1.12398	0.53018	*cysG*
WP_001276847.1	1.23564	0.48357	*phoB*
WP_001254790.1	1.02616	0.94652	*KXD89_01675*
WP_000165552.1	0.90396	0.60089	*trpS*
WP_001031729.1	1.04492	0.85704	*gph*
WP_000816280.1	1.02682	0.70108	*rpe*
WP_000742143.1	0.80532	0.28921	*O3K_02135*
WP_000343215.1	0.92126	0.5388	*damX*
WP_000439848.1	0.93359	0.60105	*aroB*
WP_000818618.1	0.79295	0.10003	*aroK*
WP_001336003.1	0.89181	0.40155	*mrcA*
WP_000045744.1	1.03131	0.9012	*nudE*
WP_000104533.1	0.94842	0.75398	*igaA*
WP_001295168.1	0.88688	0.48548	*DXE50_21820*
WP_000660483.1	0.75215	0.16604	*O3K_02070*
WP_001135574.1	0.95535	0.96026	*hslO*
WP_001265681.1	0.92218	0.72458	*pckA*
WP_000980727.1	1.20771	0.18585	*KXD89_01555*
WP_001200455.1	1.11135	0.73457	*feoA*
WP_000737039.1	1.00266	0.997	*feoB*
WP_001060070.1	0.91055	0.63455	*bioH*
WP_000619389.1	0.88969	0.50969	*nfuA*
WP_001131758.1	0.78856	0.17061	*gntT*
WP_000815099.1	1.05789	0.81588	*DXE50_21955*
WP_000371928.1	0.7822	0.23069	*glpE*
WP_000448136.1	0.78828	0.09002	*glpD*
WP_000993449.1	1.24295	0.22903	*DXE50_21995*
WP_001197646.1	1.34817	0.09544	*glgA*
WP_000253975.1	1.35167	0.09253	*glgC*
WP_000192523.1	1.33964	0.21433	*glgX*
WP_000799956.1	1.06041	0.56859	*asd*
WP_000210111.1	0.86368	0.46731	*gntU*
WP_000730252.1	1.10354	0.67933	*A13A_03767*
WP_000639811.1	1.30925	0.25275	*DXE50_22045*
WP_000236293.1	1.02518	0.70797	*DXE50_22050*
WP_001295206.1	1.35388	0.19812	*G711_04008*
WP_000595082.1	1.45722	0.20658	*ggt*
WP_000825996.1	1.58391	0.0503	*EKN05_021790*
WP_000073623.1	0.97313	0.86804	*ugpQ*
WP_000907792.1	1.66345	0.08645	*EL76_0318*
WP_000803190.1	1.37752	0.07338	*A1UI_03729*

WP_000778768.1	1.31378	0.12859	*panM*
WP_001042003.1	0.96195	0.81631	*ftsX*
WP_000617723.1	0.9832	0.89451	*ftsE*
WP_001040654.1	0.88946	0.39141	*ftsY*
WP_000743193.1	1.02243	0.93051	*ECMP0215528_3971*
WP_001311191.1	1.58098	0.12332	*KXD89_01265*
WP_000042895.1	0.83379	0.37104	*KXD89_01260*
WP_000106551.1	0.98919	0.90157	*zntA*
WP_000130621.1	0.97513	0.87731	*tusA*
WP_001245295.1	1.34542	0.05142	*dcrB*
WP_001190062.1	0.84818	0.3406	*nikR*
WP_000149156.1	0.95467	0.80251	*G711_04052*
WP_000361477.1	1.22999	0.25307	*KXD89_01155*
WP_000439170.1	1.07128	0.77441	*KXD89_01130*
WP_000902780.1	1.04204	0.84536	*DXE50_22455*
WP_000323571.1	1.1982	0.20152	*uspA*
WP_000686620.1	0.76146	0.11631	*rsmJ*
WP_001298719.1	0.91504	0.67531	*HMPREF1595_03282*
WP_001300574.1	0.88909	0.49444	*rlmJ*
WP_000160816.1	0.9435	0.87554	*gorA*
WP_001296814.1	1.0796	0.81017	*DXE50_22570*
WP_001298717.1	1.33107	0.11085	*hdeB*
WP_000756550.1	1.09764	0.62258	*hdeA*
WP_001081984.1	1.4869	0.18293	*mdtE*
WP_000784827.1	1.06903	0.83628	*ccp*
WP_000934216.1	1.29964	0.38279	*treF*
WP_000357790.1	0.90655	0.6197	*KXD89_00965*
WP_000191237.1	1.34949	0.31677	*A1UI_03828*
WP_000037562.1	0.81858	0.15268	*WG3_04286*
WP_001163141.1	0.99616	0.97431	*A1UI_03833*
WP_000858214.1	0.83714	0.20214	*dctA*
WP_001266293.1	0.98381	0.9148	*hmsP*
WP_000107012.1	1.27309	0.30583	*A1UI_03843*
WP_001196486.1	1.1982	0.32058	*dppD*
WP_000084677.1	1.34697	0.31986	*dppC*
WP_000938855.1	1.10226	0.68298	*ECMP0215528_4093*
WP_001222883.1	1.2582	0.11876	*dppA*
WP_001269197.1	0.7458	0.15404	*HMPREF1620_03849*
WP_000438957.1	1.28784	0.39997	*tag*
WP_000013950.1	1.05959	0.81056	*bisC*
WP_000747625.1	1.05379	0.79552	*DXE50_22830*
WP_000805038.1	0.91905	0.70289	*ghrB*
WP_000190516.1	1.16751	0.39813	*DXE50_22840*
WP_000014594.1	0.93057	0.78347	*cspA*
WP_001291772.1	0.93466	0.81249	*glyS*
WP_001168560.1	0.96849	0.96381	*glyQ*
WP_000275334.1	1.20383	0.54079	*xylB*
WP_001149591.1	0.79146	0.09675	*xylA*

WP_000694881.1	0.98429	0.87449	*DXE50_22900*
WP_000494484.1	0.76096	0.18365	*DXE50_22915*
WP_000144363.1	0.9578	0.72916	*avtA*
WP_000514240.1	1.14599	0.65724	*A1UI_03880*
WP_000164036.1	0.77289	0.13749	*O3K_00925*
WP_000183980.1	1.27853	0.17251	*aldB*
WP_000582468.1	1.07355	0.76751	*selB*
WP_000206275.1	0.93824	0.70753	*selA*
WP_000779792.1	1.07828	0.75319	*DXE50_23010*
WP_000072850.1	0.91789	0.66202	*O3K_00870*
WP_000093247.1	1.08089	0.68597	*O3K_00845*
WP_000645439.1	1.19184	0.21262	*mtlD*
WP_000517100.1	1.49101	0.08929	*DXE50_23040*
WP_000665680.1	0.96878	0.84792	*ECMP0215528_4158*
WP_000586962.1	0.88241	0.4656	*lldD*
WP_000932347.1	1.14459	0.56907	*trmL*
WP_001277561.1	1.04224	0.86566	*cysE*
WP_001076194.1	1.16025	0.41834	*gpsA*
WP_000003377.1	1.05716	0.58069	*secB*
WP_000024392.1	0.89496	0.41534	*grxC*
WP_001156181.1	1.08939	0.65339	*DXE50_23100*
WP_001350558.1	0.89747	0.5587	*gpmM*
AIN33940.1	0.96312	0.83513	-
WP_000646007.1	0.97811	0.91828	*tdh*
WP_001213834.1	1.07911	0.50202	*kbl*
WP_000842820.1	1.00178	1	*yibB*
WP_000587764.1	0.96109	0.99921	*rfaD*
WP_000699219.1	0.81571	0.31785	*O3K_00725*
WP_001264584.1	1.02882	0.93949	*rfaC*
WP_001395405.1	1.083	0.80187	*waaL*
WP_001236433.1	0.79039	0.25056	*KXD89_00410*
WP_000790279.1	0.93428	0.72421	*waaZ*
WP_000615254.1	0.85928	0.37966	*rfaY*
WP_000376841.1	0.95025	0.78548	*rfaJ*
WP_001188013.1	0.95948	0.82107	*waaO*
WP_000158225.1	0.68365	0.06584	*rfaS*
WP_000229840.1	0.93874	0.74126	*rfaP*
WP_000634283.1	1.15362	0.64098	*rfaG*
WP_000891564.1	0.96468	0.76658	*A13A_03970*
WP_001171866.1	0.94226	0.7258	*coaD*
WP_001114543.1	1.02474	0.92287	*mutM*
pdb|6H58|XX	1.03974	0.64823	-
WP_000050139.1	0.94117	0.64042	*coaBC*
AIN33967.1	1.14247	0.31657	-
WP_000818601.1	0.99555	0.93667	*slmA*
WP_000806177.1	0.87678	0.44543	*pyrE*
WP_000621340.1	0.99772	0.94863	*yicC*
WP_001295237.1	1.0699	0.72943	*gmk*

WP_000135058.1	0.85399	0.31253	*rpoZ*
WP_000280488.1	1.00951	0.97379	*spoT*
WP_001070177.1	1.03301	0.89565	*trmH*
WP_000678419.1	1.02854	0.94023	*recG*
WP_000468833.1	1.19349	0.56042	*gltS*
WP_001300954.1	1.15594	0.54082	*KXD89_00250*
WP_000779426.1	1.32521	0.34762	*O3K_00230*
WP_001065718.1	1.42127	0.23886	*adeD*
WP_000879194.1	0.92785	0.6997	*uhpT*
WP_000633668.1	1.089	0.72131	*uhpA*
WP_001181706.1	0.98572	0.92628	*ilvN*
WP_000168475.1	0.9053	0.46216	*A1UI_03990*
WP_000620888.1	0.68015	0.06232	*EKN05_019820*
WP_001336371.1	1.05761	0.86497	*KXD89_00070*
WP_000174305.1	1.11733	0.72083	*ECMP0215528_4259*
WP_000985549.1	0.86907	0.3091	*DXE50_23915*
WP_000522208.1	1.21669	0.4063	*yidB*
WP_000072067.1	0.95156	0.93319	*gyrB*
WP_000060112.1	0.96089	0.82651	*recF*
WP_000673464.1	0.93826	0.62523	*dnaN*
WP_000059111.1	1.06317	0.75669	*dnaA*
WP_000831330.1	0.97128	0.80272	*rpmH*
WP_000239730.1	1.21397	0.41173	*rnpA*
WP_000378250.1	0.84944	0.29114	*yidC*
WP_001282346.1	1.11341	0.56615	*mnmE*
WP_001295247.1	0.87304	0.41161	*tnaA*
WP_000131925.1	0.96836	0.85535	*A1UI_04026*
WP_000377786.1	0.97832	0.89208	*phoU*
WP_000063125.1	0.9348	0.69197	*pstB*
WP_000867146.1	0.94294	0.64974	*pstS*
WP_000334099.1	1.17091	0.25267	*glmS*
WP_000933736.1	0.89659	0.42254	*glmU*
WP_001251965.1	0.8857	0.48526	*atpC*
WP_000190506.1	0.96223	0.99373	*atpD*
WP_000896498.1	0.92101	0.71648	*atpG*
WP_001176745.1	0.91515	0.67611	*atpA*
WP_001288587.1	0.88103	0.45741	*atpH*
WP_001052219.1	0.96717	0.97011	*atpF*
WP_000429386.1	1.07013	0.53329	*atpE*
WP_000135625.1	0.97677	0.83296	*atpB*
WP_000932839.1	0.92209	0.63509	*rsmG*
WP_000499788.1	0.90139	0.4442	*mnmG*
WP_000432970.1	1.33741	0.21697	*asnC*
WP_001299914.1	1.19735	0.44609	*ravA*
WP_000102319.1	1.0914	0.71429	*kup*
WP_001301979.1	0.93772	0.62241	*rbsD*
WP_000387770.1	0.93046	0.58498	*rbsA*
WP_000211858.1	0.91696	0.61246	*rbsC*

WP_001056271.1	0.94961	0.9192	*rbsB*
WP_001300603.1	0.90868	0.47797	*rbsK*
WP_001131164.1	0.96594	0.83478	*DXE50_24215*
WP_000379245.1	0.88047	0.5253	*hdfR*
WP_000841001.1	0.85498	0.31731	*maoP*
WP_000208520.1	1.01053	0.98441	*ilvE*
WP_001127399.1	1.03617	0.88531	*ilvD*
WP_000785596.1	0.92619	0.69341	*ilvA*
WP_000024939.1	0.96088	0.74588	*ilvC*
WP_001140251.1	0.88481	0.37172	*DXE50_24310*
WP_001238899.1	0.9461	0.76952	*rep*
WP_001295254.1	1.03367	0.89347	*gppA*
WP_000047499.1	0.85214	0.30371	*rhlB*
WP_001280776.1	0.91794	0.69526	*trxA*
WP_001054527.1	0.90567	0.61216	*rho*
WP_001050960.1	1.20489	0.5388	*wecA*
WP_001295256.1	0.78556	0.08647	*wzzE*
WP_001340422.1	0.94198	0.72453	*wecB*
WP_000006621.1	1.00232	0.97391	*wecC*
WP_001226601.1	0.80121	0.20085	*rffG*
WP_000676056.1	0.98933	0.93598	*rfbA*
WP_000612043.1	0.94308	0.72954	*wecE*
WP_000217234.1	1.0585	0.86274	*rffT*
WP_001064040.1	0.82746	0.27372	*rffM*
WP_000774731.1	0.92299	0.68127	*DXE50_24410*
WP_000921791.1	1.04963	0.60932	*hemY*
WP_000138997.1	1.04073	0.85098	*hemX*
WP_000026046.1	1.23467	0.48525	*EL76_4618*
WP_001338644.1	0.91974	0.53129	*hemC*
WP_000999947.1	1.11311	0.5672	*cyaY*
WP_000799889.1	1.0844	0.73489	*DXE50_24485*
WP_001160654.1	0.72335	0.06225	*dapF*
WP_000812796.1	1.03929	0.8752	*DXE50_24495*
WP_000130691.1	0.76496	0.19201	*xerC*
WP_001213584.1	0.84488	0.32944	*yigB*
WP_000383406.1	0.98325	0.91486	*uvrD*
WP_000947159.1	0.976	0.88134	*corA*
WP_001277142.1	0.85459	0.43701	*DXE50_24560*
WP_001259700.1	0.97066	0.79933	*pldA*
WP_000487654.1	0.84225	0.39728	*pldB*
WP_000285362.1	0.97832	0.89208	*yigL*
WP_000153907.1	0.96952	0.95893	*metE*
WP_001336442.1	1.07591	0.70551	*EL76_4589*
WP_000045177.1	1.21171	0.17956	*O3K_24700*
WP_000347714.1	0.99057	0.94072	*rmuC*
WP_000227958.1	0.97855	0.84276	*ubiE*
WP_001295259.1	0.81431	0.31392	*ubiJ*
WP_000187530.1	1.74178	0.0613	*ubiB*

WP_001295260.1	1.14703	0.45699	*tatA*
WP_000459594.1	1.04407	0.85976	*tatB*
WP_001192396.1	1.17325	0.60036	*rfaH*
WP_000339804.1	1.06208	0.76118	*ubiD*
WP_000209826.1	0.83143	0.18594	*DXE50_24695*
WP_000438725.1	1.34502	0.09812	*fadA*
WP_000965936.1	1.33806	0.1043	*fadB*
WP_000444561.1	0.97388	0.93822	*pepQ*
WP_000853959.1	1.17761	0.4896	*hemG*
WP_001295263.1	1.26195	0.19719	*DXE50_24765*
WP_001065497.1	1.10559	0.67355	*srkA*
WP_000725337.1	0.94447	0.88245	*dsbA*
WP_000250006.1	0.92013	0.71038	*polA*
WP_000183349.1	1.04763	0.82146	*engB*
WP_001295266.1	0.93087	0.58707	*yihI*
WP_000116090.1	1.17975	0.36569	*hemN*
WP_001188777.1	0.84124	0.31731	*ntrC*
WP_000190577.1	0.85284	0.30705	*DXE50_24825*
WP_001271717.1	0.76894	0.0564	*DXE50_24830*
WP_000570668.1	0.97764	0.92051	*typA*
WP_000059678.1	0.82726	0.27309	*A13A_04267*
WP_001295269.1	1.25771	0.20396	*yihX*
WP_000560983.1	0.993	0.95981	*dtd*
WP_001297068.1	1.23809	0.3655	*WG3_04779*
WP_000027703.1	1.00344	0.98006	*fdhE*
WP_000331377.1	0.97244	0.86488	*fdxH*
WP_010723259.1	0.97146	0.94968	*fdnG*
WP_000753617.1	0.96541	0.84395	*fdhD*
WP_000122641.1	0.8421	0.25849	*sodA*
WP_001270260.1	0.94483	0.73754	*O3K_24310*
WP_000580417.1	1.04733	0.89125	*cpxA*
WP_001033722.1	1.2504	0.1275	*cpxR*
WP_001223800.1	1.68428	0.07895	*cpxP*
WP_000591795.1	0.97586	0.92886	*pfkA*
WP_001326656.1	0.84512	0.33023	*cdh*
WP_001216325.1	1.12072	0.37331	*tpiA*
WP_000802216.1	0.92057	0.67212	*KXD89_21165*
WP_000655989.1	1.21711	0.40547	*EL76_4498*
WP_000323556.1	1.14243	0.47103	*ECMP0215528_4537*
WP_000796332.1	1.08063	0.74616	*EL76_4496*
WP_001250644.1	1.09513	0.63186	*glpX*
WP_000136788.1	0.96104	0.99946	*glpK*
WP_000084268.1	1.06594	0.7908	*glpF*
WP_001296623.1	1.10785	0.41024	*zapB*
WP_000872908.1	1.18725	0.34681	*rraA*
WP_000139496.1	0.79352	0.1821	*menA*
WP_001293341.1	0.99644	0.83362	*hslU*
WP_000208242.1	0.94161	0.86197	*hslV*

WP_000068828.1	0.93849	0.62641	*ftsN*
WP_000644904.1	1.04259	0.86454	*cytR*
WP_000710769.1	0.85827	0.33349	*rpmE*
WP_000852812.1	0.84708	0.23247	*metJ*
WP_001295694.1	0.97075	0.85702	*A1UI_04255*
WP_000110772.1	0.91584	0.51223	*metL*
WP_001295695.1	1.25087	0.12696	*katG*
WP_000374004.1	1.13642	0.48978	*gldA*
WP_000424840.1	0.94486	0.73769	*fsa*
WP_001174077.1	0.9909	0.94198	*ptsP*
WP_000556306.1	1.00127	0.96817	*KXD89_20995*
WP_001005586.1	0.98823	0.87116	*ppc*
WP_001298964.1	0.96363	0.76084	*argE*
WP_000935370.1	1.07998	0.80925	*argC*
WP_001302318.1	1.26599	0.31724	*argB*
WP_001230087.1	1.21473	0.28391	*argH*
WP_001025939.1	1.01603	0.95967	*oxyR*
WP_001120810.1	0.90856	0.63145	*sthA*
WP_000187022.1	0.82445	0.16732	*trmA*
WP_000201820.1	0.9481	0.75251	*murI*
WP_001016699.1	1.04247	0.86491	*murB*
WP_000654630.1	0.89824	0.53221	*birA*
WP_000023081.1	0.98391	0.87239	*coaA*
WP_001275702.1	0.96292	0.82079	*secE*
WP_001287516.1	0.88433	0.47706	*nusG*
WP_001085926.1	0.87086	0.39951	*rplK*
WP_001096684.1	0.92014	0.71044	*rplA*
WP_001207201.1	0.89316	0.53131	*rplJ*
WP_000028878.1	0.87236	0.40784	*rplL*
WP_000263098.1	0.9396	0.84766	*rpoB*
WP_000653944.1	0.9235	0.73383	*rpoC*
WP_000934302.1	0.85382	0.3602	*rsd*
WP_000373940.1	1.19193	0.45774	*nudC*
WP_000137657.1	0.95953	0.73854	*hemE*
WP_000362388.1	1.27143	0.42491	*nfi*
WP_001044513.1	1.02907	0.69173	*hupA*
WP_000866800.1	0.97177	0.86178	*purD*
WP_001187559.1	0.93359	0.60104	*purH*
WP_000138905.1	1.23893	0.14138	*aceB*
WP_000857856.1	1.21685	0.17175	*aceA*
WP_001137220.1	1.75684	0.05735	*aceK*
WP_000632913.1	0.9388	0.74149	*espL4*
WP_000096011.1	0.86761	0.3036	*metH*
WP_000956830.1	1.53326	0.15087	*DXE50_25790*
WP_000421763.1	1.31647	0.12569	*pepE*
WP_000936377.1	1.12497	0.7035	*A1UI_04335*
WP_001207634.1	0.81575	0.31796	*A1UI_04336*
WP_001290310.1	0.78624	0.1654	*A1UI_04337*

WP_000789986.1	0.98235	0.89847	*pgi*
WP_001326644.1	0.94505	0.76547	*ubiC*
WP_000455227.1	0.96002	0.82318	*ubiA*
WP_000017354.1	0.9455	0.8898	*plsB*
WP_000646078.1	0.90332	0.45301	*lexA*
WP_001030593.1	1.18209	0.35971	*DXE50_25970*
WP_001295691.1	0.89907	0.59217	*zur*
WP_001298868.1	1.01929	0.94508	*dusA*
WP_000235508.1	1.24244	0.137	*KXD89_20510*
WP_000918363.1	0.90162	0.54644	*DXE50_26005*
WP_001147328.1	0.96129	0.81327	*alr*
WP_000486985.1	0.8222	0.16159	*tyrB*
WP_001226928.1	0.89197	0.40222	*DXE50_26025*
WP_000270375.1	1.53183	0.15177	*DXE50_26030*
WP_000155657.1	1.06859	0.73471	*DXE50_26035*
WP_000357740.1	1.08656	0.66415	*uvrA*
WP_000168305.1	0.93996	0.85024	*DXE50_26045*
WP_000106882.1	0.91278	0.64289	*DXE50_26070*
WP_000402210.1	0.78851	0.24592	*KY274_20395*
WP_000832573.1	1.30142	0.26353	*actP*
WP_000078239.1	1.31105	0.07216	*acs*
WP_000719886.1	0.87946	0.52174	*DXE50_26140*
WP_001046187.1	0.87273	0.42977	*alsB*
WP_000083216.1	0.88225	0.36111	*rpiR*
WP_000716794.1	0.99788	0.98217	*rpiB*
WP_000971886.1	0.97679	0.88784	*phnN*
WP_001300891.1	1.10621	0.59152	*DXE50_26305*
WP_000697926.1	1.10811	0.74209	*basR*
WP_000986601.1	1.19155	0.56417	*A1UI_04504*
WP_000611288.1	0.93337	0.72071	*dcuR*
WP_001295383.1	1.0355	0.8736	*DXE50_26450*
WP_001188520.1	1.26996	0.18492	*DXE50_26655*
WP_000068922.1	1.34184	0.32623	*dsbD*
WP_000883400.1	0.82981	0.359	*cutA*
WP_000961959.1	0.809	0.22104	*DXE50_26670*
WP_000069437.1	1.11226	0.39729	*aspA*
WP_001267448.1	1.12092	0.71265	*ECMP0215528_4825*
WP_001026276.1	0.88378	0.47372	*groES*
WP_000729117.1	0.89587	0.54847	*groL*
WP_000558209.1	1.17186	0.38638	*DXE50_26700*
WP_000940549.1	0.85358	0.35933	*epmB*
WP_000257278.1	0.77503	0.06571	*efp*
WP_000239596.1	1.16026	0.41832	*ecnB*
WP_001238378.1	1.34048	0.21335	*O3K_22885*
WP_001336292.1	1.03272	0.8966	*KXD89_19985*
WP_000208757.1	1.06028	0.85821	*frdC*
WP_000829498.1	1.13406	0.59616	*DXE50_26755*
WP_001192973.1	1.21488	0.28358	*frdA*

WP_000004771.1	0.91149	0.58866	*epmA*
WP_001236847.1	1.05718	0.81811	*ECMP0215528_4846*
WP_000934920.1	1.02532	0.91827	*psd*
WP_000041964.1	0.99428	0.92963	*rsgA*
WP_001295188.1	1.00127	0.96815	*orn*
WP_001295189.1	1.16889	0.50979	*nnrD*
WP_000981977.1	0.8986	0.53373	*tsaE*
WP_001280345.1	1.14091	0.57844	*miaA*
WP_001051883.1	1.08883	0.46955	*hfq*
WP_000460362.1	1.02138	0.93575	*hflX*
WP_000312488.1	0.9286	0.7696	*hflK*
WP_001232412.1	1.02873	0.69312	*hflC*
WP_000527955.1	1.03679	0.66011	*purA*
WP_001177639.1	0.87691	0.5128	*nsrR*
WP_000076332.1	1.16589	0.40259	*rnr*
WP_001293282.1	0.94848	0.75425	*rlmB*
WP_000569707.1	1.23755	0.4803	*yjfP*
WP_000133631.1	1.11498	0.72621	*ulaR*
WP_001216676.1	0.8051	0.12987	*rpsF*
WP_001315977.1	1.12181	0.71064	*priB*
pdb|7OI0|R	1.11373	0.39307	-
WP_001196062.1	0.99246	0.85177	*rplI*
WP_001119478.1	1.16893	0.50969	*ytfB*
WP_000211225.1	0.8875	0.49622	*DXE50_27040*
WP_000331456.1	0.72817	0.06779	*ytfE*
WP_000560561.1	1.00078	0.99546	*A13A_04864*
WP_000589431.1	1.0111	0.76833	*cpdB*
WP_000886919.1	1.05202	0.80295	*cysQ*
WP_000175279.1	1.01885	0.94706	*KXD89_19650*
WP_000935036.1	0.86156	0.46018	*WG3_00121*
WP_001295196.1	1.21032	0.29335	*msrA*
WP_001269327.1	1.17298	0.50024	*tamA*
WP_000060911.1	1.2019	0.31206	*tamB*
WP_001219160.1	1.03037	0.90429	*DXE50_27115*
WP_000055075.1	0.77357	0.06338	*ppa*
WP_000265913.1	1.40195	0.05863	*ytfQ*
WP_001219813.1	0.8735	0.32606	*mpl*
WP_000166270.1	1.32256	0.23526	*yjgA*
WP_001162171.1	0.99867	0.82356	*pmbA*
WP_000187778.1	1.08167	0.74304	*nrdD*
WP_000047539.1	0.813	0.15244	*DXE50_27225*
WP_000230281.1	0.83127	0.3634	*ECMP0215528_4971*
WP_000256658.1	0.79873	0.27177	*KXD89_19490*
WP_000036434.1	0.95965	0.82173	*KY274_19440*
WP_000002953.1	1.04861	0.81733	*rraB*
WP_001059402.1	0.91775	0.61596	*A311_00387*
WP_000416392.1	0.85985	0.34141	*valS*
WP_000786400.1	0.92355	0.68339	*holC*

WP_000397144.1	0.93057	0.7835	*pepA*
WP_000584114.1	0.94931	0.75809	*lptF*
WP_001295681.1	0.96392	0.82543	*lptG*
WP_001294573.1	1.31522	0.12702	*HMPREF1599_05895*
WP_000998695.1	1.07846	0.81296	*O3K_22310*
WP_000175457.1	0.90998	0.58215	*DXE50_19750*
WP_000373366.1	0.98522	0.92398	*A13A_04969*
WP_000695564.1	1.57182	0.12821	*HMPREF1589_00471*
WP_000438562.1	0.9742	0.81881	*uxuA*
WP_000208205.1	0.94874	0.68047	*HMPREF1589_00461*
WP_000833679.1	0.85451	0.36263	*A1UI_04679*
WP_000568432.1	0.97406	0.81802	*iadA*
WP_001300012.1	0.78565	0.23895	*HMPREF1589_00446*
WP_000443951.1	0.68574	0.068	*mcrB*
WP_001272447.1	0.78879	0.17112	*HMPREF1589_00433*
WP_001063204.1	0.7933	0.1008	*HMPREF1589_00432*
WP_000217931.1	0.93468	0.69142	*HMPREF1589_00430*
WP_001312515.1	0.83956	0.20929	*O3K_21780*
WP_001299714.1	1.10656	0.59025	*G925_04708*
WP_001308243.1	0.90539	0.61541	*DXE50_27710*
WP_000799911.1	1.19885	0.55019	*dnaC*
WP_000098818.1	0.92599	0.6524	*dnaT*
WP_001272330.1	1.06131	0.76434	*rsmC*
WP_000204012.1	1.08804	0.78965	*G925_04726*
WP_001092461.1	1.03978	0.91079	*rimI*
WP_000175940.1	0.9036	0.59852	*prfC*
WP_001295748.1	0.99616	0.8349	*osmY*
WP_001143230.1	1.39714	0.15523	-
WP_001298497.1	1.05928	0.77269	*deoC*
WP_000477807.1	1.08353	0.67577	*deoA*
WP_000816471.1	1.11983	0.37577	*deoB*
WP_000224877.1	1.05546	0.58708	*deoD*
WP_000105884.1	0.95279	0.77406	*lplA*
WP_001132955.1	0.94751	0.74982	*DXE50_28030*
WP_001029687.1	0.94496	0.66038	*radA*
WP_000093814.1	0.92832	0.6628	*nadR*
WP_000046749.1	1.00318	0.80331	*ettA*
WP_000409451.1	1.28192	0.29208	*slt*
WP_000068679.1	1.08961	0.71953	*trpR*
WP_000942344.1	0.81034	0.22464	*gpmB*
WP_000371666.1	1.26923	0.18601	*robA*
WP_000875487.1	1.02185	0.93369	*creA*
WP_001194358.1	1.17348	0.24746	*arcA*
WP_001223132.1	1.0654	0.79249	*trmJ*
QBM92672.2	0.97589	0.88437	-
WP_000737282.1	0.81067	0.30378	-
WP_000875061.1	1.11914	0.71669	-
WP_001306825.1	1.16561	0.61595	*ycgN*

WP_001350503.1	0.79271	0.09857	-
WP_001307844.1	1.02113	0.93485	*rnd*
QWY90515.1	0.71325	0.10146	-
QWY90557.1	0.96193	0.83051	-

The up-regulated proteins are highlighted in orange, while blue for down-regulated proteins. Target genes for analysis are in red.
