# Supplementary material for: A new variant of the colistin resistance gene MCR-1 with co-resistance to β-lactam antibiotics reveals a potential novel antimicrobial peptide
Source: PLoS Biol. 2023 Dec 13;21(12):e3002433. doi: 10.1371/journal.pbio.3002433 (PMC10786390; doi:10.1371/journal.pbio.3002433)
Supplement: S5 Table — (PDF) [file pbio.3002433.s026.pdf]

Table S5. The significantly enriched GO terms of differentially expressed proteins between *E. coli* BW25113 carrying WT MCR-1 and M6.

GO_ID	Term	Test	Ref	TestAll	RefAll	Test_per	Ref_per	P value	FDR	Rich Factor
GO:0030313	cell envelope	17	172	97	2350	0.175257732	0.073191489	0.0004768	0.16655752	0.098837209
GO:0031975	envelope	17	175	97	2350	0.175257732	0.074468085	0.000585571	0.16655752	0.097142857
GO:0030288	outer membrane-bounded periplasmic space	11	103	97	2350	0.113402062	0.043829787	0.00282408	0.344537731	0.106796117
GO:0042597	periplasmic space	12	127	97	2350	0.12371134	0.054042553	0.005088546	0.349201477	0.094488189
GO:0071944	cell periphery	32	559	97	2350	0.329896907	0.23787234	0.02272628	0.387364975	0.057245081
GO:0019867	outer membrane	7	70	97	2350	0.072164948	0.029787234	0.023382679	0.387364975	0.1
GO:0009279	cell outer membrane	6	63	97	2350	0.06185567	0.026808511	0.04304829	0.397201869	0.095238095
GO:0044462	external encapsulating structure part	6	63	97	2350	0.06185567	0.026808511	0.04304829	0.397201869	0.095238095
GO:0009338	exodeoxyribonuclease V complex	1	2	97	2350	0.010309278	0.000851064	0.080866281	0.446419352	0.5
GO:0009289	pilus	1	2	97	2350	0.010309278	0.000851064	0.080866281	0.446419352	0.5
GO:0030312	external encapsulating structure	6	75	97	2350	0.06185567	0.031914894	0.086215692	0.459912075	0.08
GO:0005886	plasma membrane	26	502	97	2350	0.268041237	0.213617021	0.114912024	0.499936259	0.051792829
GO:0016021	integral component of membrane	24	472	97	2350	0.24742268	0.200851064	0.149410422	0.53946267	0.050847458
GO:0031224	intrinsic component of membrane	24	487	97	2350	0.24742268	0.207234043	0.190702895	0.572108684	0.049281314
GO:0016020	membrane	33	696	97	2350	0.340206186	0.296170213	0.194793149	0.581203473	0.047413793
GO:0044425	membrane part	25	524	97	2350	0.257731959	0.222978723	0.234081901	0.630392568	0.047709924
GO:0046930	pore complex	1	7	97	2350	0.010309278	0.002978723	0.255806449	0.642735654	0.142857143
GO:0045203	integral component of cell outer membrane	1	8	97	2350	0.010309278	0.003404255	0.286615998	0.673885152	0.125
GO:0042995	cell projection	1	8	97	2350	0.010309278	0.003404255	0.286615998	0.673885152	0.125
GO:0043231	intracellular membrane-bounded organelle	1	10	97	2350	0.010309278	0.004255319	0.344497641	0.703082545	0.1
