# Supplementary material for: A new variant of the colistin resistance gene MCR-1 with co-resistance to β-lactam antibiotics reveals a potential novel antimicrobial peptide
Source: PLoS Biol. 2023 Dec 13;21(12):e3002433. doi: 10.1371/journal.pbio.3002433 (PMC10786390; doi:10.1371/journal.pbio.3002433)
Supplement: S6 Table — (PDF) [file pbio.3002433.s027.pdf]

Table S6. The KEGG pathways map of differentially expressed proteins between *E. coli* BW25113 carrying WT MCR-1 and M6.

Map_ID	Pathways	Test	TestAll	Ref	RefAll	Test_per	Ref_per	P value	FDR	Rich Factor
ko02026	Biofilm formation - Escherichia coli	3	97	28	2350	0.030928	0.011915	0.106128204	0.742897429	0.107142857
ko04216	Ferroptosis	1	97	2	2350	0.010309	0.000851	0.080866281	0.742897429	0.5
ko00040	Pentose and glucuronate interconversions	2	97	19	2350	0.020619	0.008085	0.183464562	0.791519469	0.105263158
ko00550	Peptidoglycan biosynthesis	2	97	21	2350	0.020619	0.008936	0.214007878	0.791519469	0.095238095
ko00330	Arginine and proline metabolism	2	97	22	2350	0.020619	0.009362	0.229463379	0.791519469	0.090909091
ko00790	Folate biosynthesis	2	97	23	2350	0.020619	0.009787	0.244994121	0.791519469	0.086956522
ko00633	Nitrotoluene degradation	1	97	6	2350	0.010309	0.002553	0.223680603	0.791519469	0.166666667
ko00051	Fructose and mannose metabolism	2	97	27	2350	0.020619	0.011489	0.307262512	0.807041971	0.074074074
ko00670	One carbon pool by folate	1	97	11	2350	0.010309	0.004681	0.371670174	0.807041971	0.090909091
ko00410	beta-Alanine metabolism	1	97	11	2350	0.010309	0.004681	0.371670174	0.807041971	0.090909091
ko00220	Arginine biosynthesis	1	97	12	2350	0.010309	0.005106	0.397727461	0.807041971	0.083333333
ko03010	Ribosome	1	97	56	2350	0.010309	0.02383	0.908299313	0.908299313	0.017857143
ko00270	Cysteine and methionine metabolism	1	97	34	2350	0.010309	0.014468	0.763916442	0.843534434	0.029411765
ko00630	Glyoxylate and dicarboxylate metabolism	1	97	29	2350	0.010309	0.01234	0.707686248	0.825633956	0.034482759
ko00052	Galactose metabolism	1	97	22	2350	0.010309	0.009362	0.606083889	0.807041971	0.045454545
ko00920	Sulfur metabolism	1	97	18	2350	0.010309	0.00766	0.533065071	0.807041971	0.055555556
ko00561	Glycerolipid metabolism	1	97	14	2350	0.010309	0.005957	0.446675733	0.807041971	0.071428571
ko00450	Selenocompound metabolism	1	97	13	2350	0.010309	0.005532	0.422714816	0.807041971	0.076923077
ko00260	Glycine, serine and threonine metabolism	2	97	35	2350	0.020619	0.014894	0.427731824	0.807041971	0.057142857
ko00511	Other glycan degradation	1	97	2	2350	0.010309	0.000851	0.080866281	0.742897429	0.5
ko00910	Nitrogen metabolism	2	97	16	2350	0.020619	0.006809	0.139178119	0.751493081	0.125

The up-regulated pathways are highlighted in red, while blue for down-regulated pathways.
