# Supplementary material for: A new variant of the colistin resistance gene MCR-1 with co-resistance to β-lactam antibiotics reveals a potential novel antimicrobial peptide
Source: PLoS Biol. 2023 Dec 13;21(12):e3002433. doi: 10.1371/journal.pbio.3002433 (PMC10786390; doi:10.1371/journal.pbio.3002433)
Supplement: S7 Table — (PDF) [file pbio.3002433.s028.pdf]

Table S7. Bacterial strains used in this study

Bacterial strains	Description	Reference/source
E.coli DH5α	Type strain (F-, φ80d lacZ ΔM15, Δ(lacZYA argF) U169, deoR , recA1 , endA1 , hsdR17 (rK-, mK+), phoA, supE44 , λ-, thi-1, gyrA96 , relA1)	Our lab
E.coli BW25113	Type strain (F- DE(araD-araB)567 lacZ4787(del)::rrnB-3 LAM-rph-1 DE(rhaD-rhaB)568 hsdR514)	Our lab
E.coli ATCC25922	Type strain	Our lab
P. aeruginosa ATCC27853	Type strain	Our lab
S. Typhimurium SL1344	Type strain	Our lab
K. pneumoniae ATCC13883	Type strain	Our lab
A. baumannii ATCC19606	Type strain	Our lab
S. aureus ATCC25913	Type strain	Our lab
A. baumannii clinical isolate 1	Clinical isolate	Our lab
A. baumannii clinical isolate 2	Clinical isolate	Our lab
E. coli CRE clinical isolate 1	Clinical isolate	Our lab
K. pneumoniae CRKP clinical isolate 1	Clinical isolate	Our lab
S. aureus MRSA clinical isolate 1	Clinical isolate	Our lab
S. aureus MRSA clinical isolate 2	Clinical isolate	Our lab
E. coli *mcr-1*$^{+}$ clinical isolate 1	Clinical isolate	Our lab
E. coli *mcr-1*$^{+}$ clinical isolate 2	Clinical isolate	Our lab
E.coli BW25113 Δ *mrcB*	*E. coli* BW25113 derivative with the deletion of *mrcB*	This study
E.coli BW25113 Δ *ycbB*	*E. coli* BW25113 derivative with the deletion of *ycbB*	This study
E.coli BW25113 Δ *ycfM*	*E. coli* BW25113 derivative with the deletion of *ycfM*	This study
