# Supplementary material for: A new variant of the colistin resistance gene MCR-1 with co-resistance to β-lactam antibiotics reveals a potential novel antimicrobial peptide
Source: PLoS Biol. 2023 Dec 13;21(12):e3002433. doi: 10.1371/journal.pbio.3002433 (PMC10786390; doi:10.1371/journal.pbio.3002433)
Supplement: S8 Table — (PDF) [file pbio.3002433.s029.pdf]

Table S8. Plasmids used in this study

Plasmids	Description	Reference/source
pACYCDuet-1	Vector carrying the P15A replicon, *lacI* gene and chloramphenicol resistance gene	Addgene #71147
pBAD24	A tightly controlled expression vectors regulated by the arabinose operon, carrying pBR322 replicon and ampicillin gene.	Our lab
pACYC-Para-*mcr-1*	pACYCDuet-1 derivative replacing T7 promoter with arabinose promoter and carrying wide type mcr-1 gene.	Our lab
pACYC-Para-M6	pACYCDuet-1 derivative replacing T7 promoter with arabinose promoter and carrying mcr-1 gene with mutations P188A+P195S.	This study
pACYC-Para-*mcr-1* P188A	pACYCDuet-1 derivative replacing T7 promoter with arabinose promoter and carrying mcr-1 gene with mutation P188A.	This study
pACYC-Para-*mcr-1* P195S	pACYCDuet-1 derivative replacing T7 promoter with arabinose promoter and carrying mcr-1 gene with mutation P195S.	This study
pACYC-NP-*mcr-1*	pACYCDuet-1 derivative replacing T7 promoter with native promoter of *mcr-1* and carrying wide type mcr-1 gene.	Our lab
pACYC-NP-M6	pACYCDuet-1 derivative replacing T7 promoter with native promoter of *mcr-1* and carrying mcr-1 gene with mutations P188A+P195S.	This study
pACYC-Para-*mcr-1* K187A	pACYCDuet-1 derivative replacing T7 promoter with arabinose promoter and carrying mcr-1 gene with mutation K187A.	This study
pACYC-Para-*mcr-1* L189A	pACYCDuet-1 derivative replacing T7 promoter with arabinose promoter and carrying mcr-1 gene with mutation L189A.	This study
pACYC-Para-*mcr-1* R190A	pACYCDuet-1 derivative replacing T7 promoter with arabinose promoter and carrying mcr-1 gene with mutation R190A.	This study
pACYC-Para-*mcr-1* S191A	pACYCDuet-1 derivative replacing T7 promoter with arabinose promoter and carrying mcr-1 gene with mutation S191A.	This study
pACYC-Para-*mcr-1* Y192A	pACYCDuet-1 derivative replacing T7 promoter with arabinose promoter and carrying mcr-1 gene with mutation Y192A.	This study
pACYC-Para-*mcr-1* V193A	pACYCDuet-1 derivative replacing T7 promoter with arabinose promoter and carrying mcr-1 gene with mutation V193A.	This study
pACYC-Para-*mcr-1* N194A	pACYCDuet-1 derivative replacing T7 promoter with arabinose promoter and carrying mcr-1 gene with mutation N194A.	This study
pACYC-Para-*mcr-1* P195A	pACYCDuet-1 derivative replacing T7 promoter with arabinose promoter and carrying mcr-1 gene with mutation P195A.	This study
pACYC-Para-*mcr-1* I196A	pACYCDuet-1 derivative replacing T7 promoter with arabinose promoter and carrying mcr-1 gene with mutation I196A.	This study
pACYC-Para-*mcr-1* M197A	pACYCDuet-1 derivative replacing T7 promoter with arabinose promoter and carrying mcr-1 gene with mutation M197A.	This study
pACYC-Para-*mcr-1* P198A	pACYCDuet-1 derivative replacing T7 promoter with arabinose promoter and carrying mcr-1 gene with mutation P198A.	This study
pACYC-Para-*mcr-1* I199A	pACYCDuet-1 derivative replacing T7 promoter with arabinose promoter and carrying mcr-1 gene with mutation I199A.	This study
pACYC-Para-*mcr-1* Y200A	pACYCDuet-1 derivative replacing T7 promoter with arabinose promoter and carrying mcr-1 gene with mutation Y200A.	This study
pACYC-Para-*mcr-1* S201A	pACYCDuet-1 derivative replacing T7 promoter with arabinose promoter and carrying mcr-1 gene with mutation S201A.	This study
pACYC-Para-*mcr-1* V202A	pACYCDuet-1 derivative replacing T7 promoter with arabinose promoter and carrying mcr-1 gene with mutation V202A.	This study
pACYC-Para-*mcr-1* G203A	pACYCDuet-1 derivative replacing T7 promoter with arabinose promoter and carrying mcr-1 gene with mutation G203A.	This study
pACYC-Para-*mcr-1* K204A	pACYCDuet-1 derivative replacing T7 promoter with arabinose promoter and carrying mcr-1 gene with mutation K204A.	This study
pACYC-Para-*mcr-1* L205A	pACYCDuet-1 derivative replacing T7 promoter with arabinose promoter and carrying mcr-1 gene with mutation L205A.	This study
pACYC-Para-*mcr-1* M197E	pACYCDuet-1 derivative replacing T7 promoter with arabinose promoter and carrying mcr-1 gene with mutation M197E.	This study
pACYC-Para-*mcr-1* L64F+I65Y	pACYCDuet-1 derivative replacing T7 promoter with arabinose promoter and carrying mcr-1 gene with mutation L64F+I65Y.	This study
pACYC-Para-*mcr-1* L165A+I168A	pACYCDuet-1 derivative replacing T7 promoter with arabinose promoter and carrying mcr-1 gene with mutation L165A+I168A.	This study
pACYC-Para-*mcr-1* M197A+Y200A	pACYCDuet-1 derivative replacing T7 promoter with arabinose promoter and carrying mcr-1 gene with mutation M197A+Y200A.	This study
pACYC-Para-*mcr-1* K204E+K211A	pACYCDuet-1 derivative replacing T7 promoter with arabinose promoter and carrying mcr-1 gene with mutation K204E+K211A.	This study
pACYC-Para-*mcr-1* M197E+R184A+K187A	pACYCDuet-1 derivative replacing T7 promoter with arabinose promoter and carrying mcr-1 gene with mutation M197E+R184A+K187A.	This study
pACYC-Para-*mcr-1* L64A+I65A+L68A+L69A	pACYCDuet-1 derivative replacing T7 promoter with arabinose promoter and carrying mcr-1 gene with mutation L64A+I65A+L68A+L69A.	This study
pACYC-Para-*mcr-1* ΔP188-P195	pACYCDuet-1 derivative replacing T7 promoter with arabinose promoter and carrying mcr-1 gene with the deletion of residues P188 to P195.	This study
