# Supplementary material for: A new variant of the colistin resistance gene MCR-1 with co-resistance to β-lactam antibiotics reveals a potential novel antimicrobial peptide
Source: PLoS Biol. 2023 Dec 13;21(12):e3002433. doi: 10.1371/journal.pbio.3002433 (PMC10786390; doi:10.1371/journal.pbio.3002433)
Supplement: S9 Table — (PDF) [file pbio.3002433.s030.pdf]

Table S9. Primers used in this study

Primers	Description	Reference/source
lpoB-sgRNA1-5F	tagcCGGGTGCCTAAACTGTCCTG	This study
lpoB-sgRNA1-3R	aaacCAGGACAGTTTAGGCACCCG	This study
lpoB-sgRNA2-5F	tagcGATGGTCAGTAAGATGCTTG	This study
lpoB-sgRNA2-3R	aaacCAAGCATCTTACTGACCATC	This study
ldtD-sgRNA1-5F	tagcTCGCTGGTCTACTATCAGAA	This study
ldtD-sgRNA1-3R	aaacTTCTGATAGTAGACCAGCGA	This study
ldtD-sgRNA2-5F	tagcATGTTGCTTAATATGATGTG	This study
ldtD-sgRNA2-3R	aaacCACATCATATTAAGCAACAT	This study
pbp1b-sgRNA1-5F	tagcGAGGTGTATCTCGGTCAGAG	This study
pbp1b-sgRNA1-3R	aaacCTCTGACCGAGATACACCTC	This study
pbp1b-sgRNA2-5F	tagcAAATCCGGGAAACCACTGCG	This study
pbp1b-sgRNA2-3R	aaacCGCAGTGGTTTCCCGGATTT	This study
lpoB-LR-5F	CTGATCATGAATAACCAACCGCC	This study
lpoB-LR-3R	TGCGAAACGGCACAAGATTCACCCCTTACAAAATATAG	This study
lpoB-RR-5F	TGCCGTTTCGCAGCAATAATCCCATCAC	This study
lpoB-RR-3R	CTGACGCGCTATGCACTAAA	This study
ldtD-LR-5F	AGCAGCGCGACTGGATGCTC	This study
ldtD-LR-3R	ATTTCCCCGAACTACTTCATCCCTTGCCCCCTGTTTTTAT	This study
ldtD-RR-5F	ATAAAAACAGGGGGCAAGGGATGAAGTAGTTCGGGGAAAT	This study
ldtD-RR-3R	AGTTGCACCGGTTTGCGCGT	This study
pbp1b-LR-5F	AAGCCGGATCGCCATTCGTTAT	This study
pbp1b-LR-3R	CGGTATTTCACGCTTAGATGGCTTTTTCTCCGCAATATTC	This study
pbp1b-RR-5F	TGCGGAGAAAAAGCCATCTAAGCGTGAAATACCG	This study
pbp1b-RR-3R	ATCTCTTCGGCGGTCACAACAGAA	This study
mcr-1 NP fuse-F	agataaaatatttctagaAGGAAAAAAGCGAAGGCT	This study
mcr-1 TAA fuse-R	attgagatctgccatatgTCAGCGGATGAATGCGGT	This study
pACYC-araC-F	GTCGACGGTATCGATAAGCTTTTATGACAACTTGACGGCTACATCA	This study
ParaBAD-mcr1-R	AAACGGGTATGGAGAAACAGTAGAGA	This study
ParaBAD-mcr1-F	CTGTTTCTCCATACCCGTTTTTTTGAGTAGTTTCTCATGATGCAG	This study
terminator-mcr1-R	CGTCGTTTTACTCAGCGGATGAATGCGGT	This study
mcr1-terminator-F	ATCCGCTGAGTAAAACGACGGCCAGTcaaa	This study
terminator-pACYC-R	CGCTCTAGAACTAGTGGATCCCAGctgcagTAACACTCGTcga	This study
MCR-1-linker delet-F	GTTTCTTTCGCGTGCATAAGATCATGCCAATCTACTCGGTGGG	This study
MCR-1-linker delet-R	ACCGAGTAGATTGGCATGATCTTATGCACGCGAAAGAAACTGG	This study
MCR-1 L189A-F	gccCGTAGCTATGTCAATCCGATCATGCCAAT	This study
MCR-1 L189A-R	TTGACATAGCTACGggcCGGCTTATGCACGCGAAA	This study
MCR-1 R190A-F	CTGgccAGCTATGTCAATCCGATCATGCCAAT	This study
MCR-1 R190A-R	TTGACATAGCTggcCAGCGGCTTATGCACGCG	This study
MCR-1 S191A-F	CTGCGTgccTATGTCAATCCGATCATGCCAAT	This study
MCR-1 S191A-R	TTGACATAggcACGCAGCGGCTTATGCACGCG	This study
MCR-1 Y192A-F	gccGTCAATCCGATCATGCCAATCTACTCGGT	This study
MCR-1 Y192A-R	ATGATCGGATTGACggcGCTACGCAGCGGCTTATGC	This study
MCR-1 V193A-F	TAGCTATgccAATCCGATCATGCCAATCTACTC	This study
MCR-1 V193A-R	TCGGATTggcATAGCTACGCAGCGGCTTATGC	This study
MCR-1 N194A-F	TATGTCgccCCGATCATGCCAATCTACTCGGT	This study
MCR-1 N194A-R	ATGATCGGggcGACATAGCTACGCAGCGGCTT	This study
MCR-1 M197A-F	AATCCGATCgccCCAATCTACTCGGTGGGTAAGC	This study
MCR-1 M197A-R	ATTGGggcGATCGGATTGACATAGCTACGCAG	This study
MCR-1 P198A-F	GATCATGgccATCTACTCGGTGGGTAAGCTTGC	This study
MCR-1 P198A-R	AGTAGATggcCATGATCGGATTGACATAGCTACG	This study
MCR-1 I199A-F	ATCATGCCAgccTACTCGGTGGGTAAGCTTGCC	This study
MCR-1 I199A-R	GAGTAggcTGGCATGATCGGATTGACATAGCT	This study
MCR-1 Y200A-F	ATCATGCCAATCgccTCGGTGGGTAAGCTTGCCA	This study
MCR-1 Y200A-R	GAggcGATTGGCATGATCGGATTGACATAGCT	This study
MCR-1 S201A-F	AATCTACgccGTGGGTAAGCTTGCCAGTATTGA	This study
MCR-1 S201A-R	TACCCACggcGTAGATTGGCATGATCGGATTGA	This study
MCR-1 V202A-F	ATCTACTCGgccGGTAAGCTTGCCAGTATTGAGTATAAA	This study
MCR-1 V202A-R	TTACCggcCGAGTAGATTGGCATGATCGGATT	This study
MCR-1 G203A-F	ATCTACTCGGTGgccAAGCTTGCCAGTATTGAGTATAAAAAA	This study
MCR-1 G203A-R	TTggcCACCGAGTAGATTGGCATGATCGGATT	This study
MCR-1 K204A-F	GTgccCTTGCCAGTATTGAGTATAAAAAAGCC	This study
MCR-1 K204A-R	AATACTGGCAAGggcACCCACCGAGTAGATTGGCA	This study
MCR-1 L205A-F	GTAAGgccGCCAGTATTGAGTATAAAAAAGCCAG	This study
MCR-1 L205A-R	AATACTGGCggcCTTACCCACCGAGTAGATTGGC	This study

MCR-1 K187A-F	TCTTTCGCGTGCATgccCCGCTGCGTAGCTATGTCAA	This study
MCR-1 K187A-R	ggcATGCACGCGAAAGAAACTGGCATAATGAC	This study
MCR-1 I196A-F	TATGTCAATCCGgccTGCCAATCTACTCGGTGGGT	This study
MCR-1 I196A-R	CAggcCGGATTGACATAGCTACGCAGCGGCTT	This study
Ec-LpxC-F	TTCAGGCGACGGGTGTCGGTTTACA	This study
Ec-LpxC-R	CCGGCGCGTTAACTTCGATAACAAT	This study
Ec-LdtD-F	TGTCGGCAATCAGTTTGTGCCTGGC	This study
Ec-LdtD-R	CGGCATACAACAGTTGAAGCTGGTT	This study
Ec-PBP1B-F	CTATGAGGATGAAGAACCGATGCCG	This study
Ec-PBP1B-R	GCTTCACCATCTCGTTCTTGCTGAT	This study
Ec-LpoB-F	TTGATTACCGCGCTGGCGATGTTTC	This study
Ec-LpoB-R	TCTTACTGACCATCGGCTGCATTGC	This study
Ec-16S-F	TCAACGATCAGTTCGTGATCGACAG	This study
Ec-AmpH-F	GTCTGCTTTTTTCTGCCGTGCT	This study
Ec-AmpH-R	TTCACGGTCCCCTGGTCGAGCAATT	This study
Ec-16S-R	CGGCCAGTTCACCAAAGTTAAAGGT	This study
rseP-ATG-5F	ATGCTGAGTTTTCTCTGG	This study
rseP-180bp-3R	aaCATATTCGGTGCCGAG	This study
cpxP-5F	ATGCGCATAGTTACCGCT	This study
cpxP-180bp-3R	TCGCATCTGCTGACGCTG	This study
M197A-Y200A-5F	ATCgcgccaatcgccTCGGTGGGTAAGCTTGCCA	This study
M197A-Y200A-3R	CGAggcgattggcgcGATCGGATTGACATAGCTACGCA	This study
M197E-5F	TCCGATCgagCCAATCTACTCGGTGGGTAAGC	This study
M197E-3R	AGATTGGctcGATCGGATTGACATAGCTACGCA	This study
R184A-K187A-M197E-5F	gctgcgtagctatgtcaatccgatcgagCCAATCTACTCGGTGGGTAAGC	This study
R184A-K187A-M197E-3R	ttgacatagctacgcagcggcgcatgcacggcAAAGAAACTGGCATAATGACTGCTG	This study
L165A-I168A-5F	AGTgctgcgctggctTTACTGCCTGTGGTGGCGTT	This study
L165A-I168A-3R	TAAagccagcgcagcACTTGCCACGATCAAGCCC	This study
L64F-I65Y-5F	atgctaTtCTAcaccacgctgttatcatcgtatcg	This study
L64F-I65Y-3R	gcgtggtgTAGaAtagcatcgcgccaaagagc	This study
L64A-I65A-L68A-L69A-5F	gcggccaccacggcggcaTCATCGTATCGCTATGTGCTAAAGC	This study
L64A-I65A-L68A-L69A-3R	tgccgccgtggtggccgcTAGCATCGCGCCAAAGAGC	This study
L64A-I65A-5F	ATGCTAgcggccACCACGCTGTTATCATCGTATCG	This study
L64A-I65A-3R	CGTGGTggccgcTAGCATCGCGCCAAAGAGC	This study
K204E-K211A-5F	gagcttgccagtattgagtatgcaaaagccagtgcgccaaaa	This study
K204E-K211A-3R	ctcaatactggcaagctCacccaccgagtagattggca	This study
