# Supplementary material for: A new variant of the colistin resistance gene MCR-1 with co-resistance to β-lactam antibiotics reveals a potential novel antimicrobial peptide
Source: PLoS Biol. 2023 Dec 13;21(12):e3002433. doi: 10.1371/journal.pbio.3002433 (PMC10786390; doi:10.1371/journal.pbio.3002433)
Supplement: S10 Table — (PDF) [file pbio.3002433.s031.pdf]

Table S10. Synthetic peptides used in this study

Peptides	Sequence	Description	Reference/source
24AA-WT	KPLRSYVNPIMPIYSVGKLASIEY	A 24AA synthetic peptide derived from linker domain of MCR-1	This study
24AA-2M	KALRSYVNSIMPIYSVGKLASIEY	A 24AA synthetic peptide derived from linker domain of M6	This study
19AA-2M-tag	KALRSYVNSIMPIYSVGKLWWWWW	A 19AA synthetic peptide derived from linker domain of M6 with five-trytophan modification at C-terminal	This study
Peptide MCR-1	KPLRSYVNPIMPIYSV	A 16AA synthetic peptide derived from linker domain of MCR-1 carrying biotin label at C-terminal	This study
Peptide M6	KALRSYVNSIMPIYSV	A 16AA synthetic peptide derived from linker domain of M6 carrying biotin label at C-terminal	This study
