# Supplementary material for: A new variant of the colistin resistance gene MCR-1 with co-resistance to β-lactam antibiotics reveals a potential novel antimicrobial peptide
Source: PLoS Biol. 2023 Dec 13;21(12):e3002433. doi: 10.1371/journal.pbio.3002433 (PMC10786390; doi:10.1371/journal.pbio.3002433)
Supplement: S1 Raw images — (DOCX) [file pbio.3002433.s033.docx]

**S1 Raw images.** Below all the raw images of western blot analysis performed in the research.


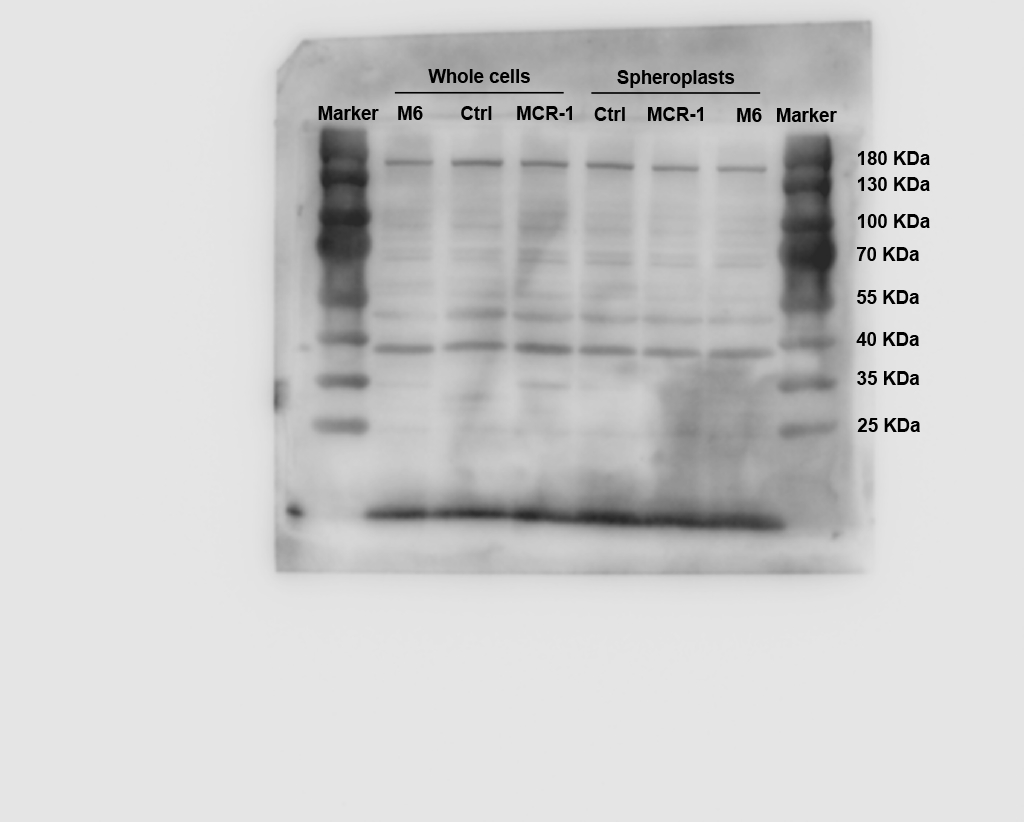


Raw images for Figure 2G LPS and Figure S10A


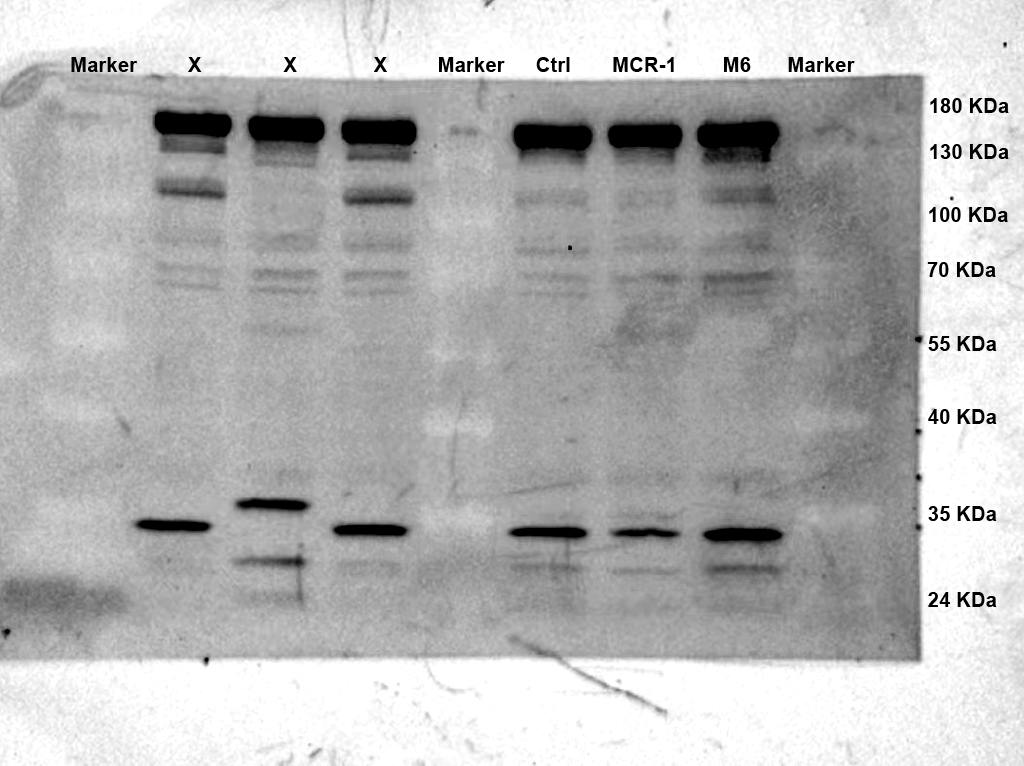


Raw images for Figure 2G PbgA


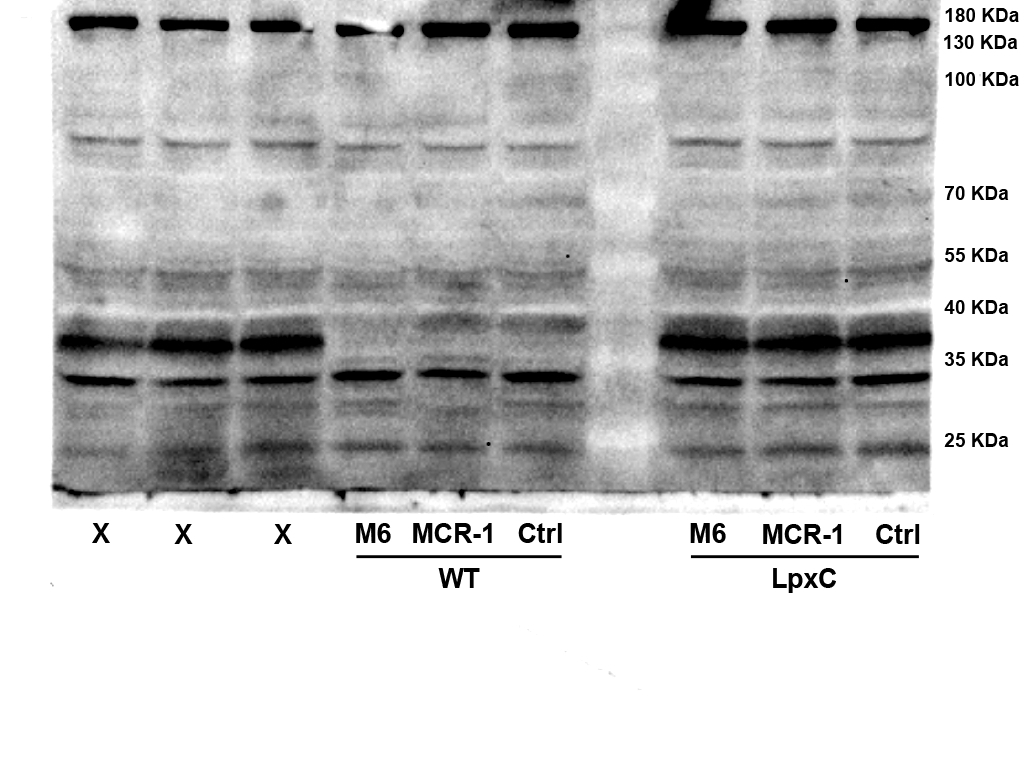


Raw images for Figure S10B
