# Supplementary material for: A new variant of the colistin resistance gene MCR-1 with co-resistance to β-lactam antibiotics reveals a potential novel antimicrobial peptide
Source: PLoS Biol. 2023 Dec 13;21(12):e3002433. doi: 10.1371/journal.pbio.3002433 (PMC10786390; doi:10.1371/journal.pbio.3002433)
Supplement: Attachment — Submitted filename: Response to Reviewers 20230918.docx [file pbio.3002433.s035.docx]

**Responding letter**

**Editor’s Comments**

Thank you for your patience while your manuscript "Identification of a novel MCR-1 variant displaying low level of co-resistance to β-lactam antibiotics that uncovers a potential novel antimicrobial peptide" was peer-reviewed at PLOS Biology. It has now been evaluated by the PLOS Biology editors, an Academic Editor with relevant expertise, and by several independent reviewers.

In light of the reviews, which you will find at the end of this email, we would like to invite you to revise the work to thoroughly address the reviewers' reports.

As you will see below, the reviewers find your work interesting, but raise some concerns that will need to be solved addressed publication. In particular, we think it is very important that you address reviewer #1's concern regarding that the claims presented in the model in Figure 5 are not sufficiently supported by the data and the reviewer asks for further experimental validation that modified lipid A accumulates in the inner membrane when the M6 MCR-1 variant is expressed. Reviewer #2 notes the reliance on the Mcr-1 overexpression system and asks that the authors provide evidence that the same phenotypes are observed in conditions of physiological MCR-1 expression.

Thank you for your kind effort handling our manuscript. Please see our point-to-point responses to the comments from each reviewer below.

Particularly, we have provided evidence to confirm the accumulation of modified lipid A on the inner membrane of M6 -bearing *E. coli*. Additionally, we have also provided compelling data to prove that M6 expression controlled by MCR-1 native promoter also resulted in phenotypic co-resistance and membrane permeabilization. Finally, we carefully edited the manuscript to improve clarity and readability. The sentences and paragraphs added to make corrections in the revised manuscript are highlighted in blue.

**#Reviewer 1**

Liang et al describe a point variant of the MCR-1 lipid A phosphoethanolamine transferase whose expression also reduces susceptibility to beta-lactam antibiotics. A screen of a previously described MCR mutant library identified a dual variant, M6, with reduced beta-lactam susceptibility. NPN and propidium iodide staining experiments identified that the outer membrane permeability of M6-expressing cells was similar to that of those expressing wild-type MCR-1, but that M6 reduced effects upon the inner membrane compared to those arising from wild-type MCR-1 expression. Proteomic comparison of M6- and wild-type MCR-1-expressing *E. coli* identified that the former induced upregulation of genes involved in peptidoglycan biosynthesis and remodeling. Increased beta-lactam MICs in the M6 strain were shown to be LdtD and PBP1b-dependent, consistent with a mechanism involving 3-3 rather than 4-3 cross-linking. The region of MCR-1 disrupted in the M6 mutant was identified as a putative lipidA binding site, as evidenced by docking experiments and molecular dynamics simulations; and by the effects of deletion of this region or mutation at positions predicted to be involved in lipidA binding. Peptides based upon the putative lipidA binding site of the M6 variant, but not wild-type MCR-1, showed antibacterial activity in in vitro assays against several Gram-negative species, and against E. coli in a mouse infection model. It is concluded differential effects upon lipidA binding in the M6 mutant, compared to wild-type MCR-1, ultimately affect LPS levels/distribution and peptidoglycan composition. Furthermore, the authors suggest that the lipidA binding properties of M6 are exploitable in new generations of antimicrobial peptides.

The manuscript contains very substantial new data providing new information on the relationship between MCR expression and the organization of the bacterial envelope, and is clearly of considerable interest in the area of Gram-negative bacterial physiology as well as antimicrobial (colistin) resistance. I would however suggest some revision and clarification before recommending acceptance.

Sincerely thanks for your appreciation of our research.

Q1: Most importantly, the connection between the data presented and the model proposed in Figure 5 is insufficiently clear. While I appreciate the uncertainties surrounding the connection between lipid A levels/modifications and the extent and type of peptidoglycan cross-linking, the reasoning underlying the model in Figure 5 is not explained either in lines 420 - 422 or in the legend. The Figure suggests that accumulation of modified lipid A in the inner membrane is occurring when the M6 variant is expressed, compared to WT MCR-1. Did the authors attempt to verify this experimentally, e.g. by mass spectrometry of spheroplasts from M6-expressing cells? It is then unclear how this relates to the reduction in PI accumulation in M6-expressing cells, compared to those expressing wild-type MCR-1, shown in Figure 1G.

Reply: We sincerely thanks the suggestion from reviewer. To further investigate the lipid A level in the inner membrane of M6 -bearing strains, we prepared the spheroplasts of indicated strains based on previous research[1]. The process in detail is as followed and has been added into the section of methods as “Preparation of spheroplasts” (line 248-257 of supplementary materials): “Spheroplasts of were generated as mentioned previously[1]. Briefly, overnight cultures of indicated strains were subcultured into fresh LB medium containing 30 μg/ml CHL and 0.2% arabinose with the ratio of 1:100. Cultures were then incubated at 37°C with shaking. Logarithmic phase cultures were collected and washed twice by centrifuging (3273× g, 20 min, 4°C), resuspending firstly in 10 ml Tris buffer (0.03 M, pH 8.0) and subsequently in Tris buffer (0.03 M, pH 8.0) containing 20% sucrose. EDTA (250 ml, 10 mg/ml) and lysozyme (1 ml, 10 mg/ml) were added to remove the OM and periplasm respectively, and the cell suspension was incubated for 1 hr in a water bath shaker at 30°C. The resulting spheroplasts produced were harvested by mild centrifugation (2000× g, 20 min, 4°C), and the morphology was observed by microscopy.”

We determined the cellular LPS levels of shperoplasts carrying empty plasmid, MCR-1 or M6 with western blot. We found that LPS level in M6 -bearing spheroplasts was lower compared to control or MCR-1 -expressing spheroplasts, demonstrating reduced LPS level on the inner membrane of M6 -bearing strain. The results have been added into the manuscript as Figure S10A followed:

**Figure S10. Cellular LPS level in spheroplasts and LpxC overexpressing strains.**

The cellular LPS level in spheroplasts **(A)** or LpxC overexpressing strains **(B)** harbouring empty plasmid, MCR-1 or M6 were determined by western blot. Error bars indicate standard errors of the means (SEM) for triple biological replicates. A two-tailed unpaired *t* test was performed to determine the statistical significance of the data. *, *P*< 0.1; **, *P*< 0.01; ***, *P*< 0.001. The bar graph was visualized with Prism 9 software.

And the demonstration of above result has been added into the manuscript as followed (line 247-251): “Consistently, the overall and IM level of LPS in M6 -expressing cells was significantly lower compared to control and MCR-1 -expressing cells (Figure 2F-G and Figure S10A). These results demonstrated that the expression of M6 led to significant reduction in cellular lipid A level, which might be responsible for the increased permeability of both OM and IM.”

Meanwhile, to further investigate the impact of M6 upon lipid A distribution, we undertook MALDI-TOF to analyse the abundance of modified or unmodified lipid A purified from MCR-1 or M6 -bearing cells. The process in detail is as followed and has been added into the section of methods as “Extraction of lipid A from *E. coli*” (line 337-350 of supplementary materials): “To identify the distribution of modified and unmodified lipid A on the IM of indicated strains, LPS was extracted from the whole cells and spheroplasts of *E. coli* BW25113 bearing MCR-1 or M6. Briefly, overnight cultures of target strains were inoculated into 100ml LB broth containing 30 µg/ml CHL and 0.2% arabinose with the ratio of 1:100. After cultivation for 2 hr at 37 °C, spheroplasts were generated as mentioned previously[1]. All the samples were collected by centrifugation (8,000 rpm for 5 min). Next, for each sample, cellular LPS was extracted by using LPS extraction kit (BestBio) according to the manufacturers’ protocol. The well-extracted LPS was subsequently hydrolysed into lipid A based on previous research[2]. LPS was cleaved by hydrolysis in 20 mM Na acetic acid-sodium acetate buffer, pH 4.5, and 1% Na dodecyl sulfate at 100°C for 1 h at a concentration of 5 mg/ml. Next, lipid A was recovered from the lyophilized residue by two extractions with a volume of 0.5 ml of a chloroform-methanol-water (3:2:0.25, v/v) mixture. The air-dried lipid A was analyzed on a Bruker Microflex mass spectrometer (Bruker Daltonics, Billerica, MA) in the negative-ion mode with reflectron mode. The relative height of ion peak for lipid A or PEA-lipid A were counted to determine their relative abundance.”

By determining the abundance of modified or unmodified lipid A extracted from whole cells or spheroplasts, we found that the abundance of modified lipid A was higher than that of the unmodified one in the M6 -bearing spheroplasts, indicating accumulation of modified lipid A on the IM of M6. The results have been added into the manuscript as Figure S13E as followed:

**Figure S13. Impact of P188A+P195S mutations upon lipid A binding pocket.**

**(A)** represents the RMSD fluctuations for WT and M6 of MCR1-lipid A. The variations in MCR1 MM-GBSA binding energy with WT and M6 during the MD trajectories are shown in **(B).**

**(C-D)** The statistics for the observed molecular interactions between lipid A and MCR-1 over the simulated trajectories of WT and the M6 mutant (the last 200-ns trajectories used for binding free energy calculations).

**(E)** Quantification of lipid A with or without the modification of phosphoethanolamine (PEA). The bar graph represented the abundance of modified lipid A and unmodified lipid A extracted from the whole cells and spheroplasts of *E. coli* BW25113 expressing MCR-1 or M6, which was determined by MALDI-TOF. The experiments were performed three times with similar results.

And the demonstration of above result has been added into the manuscript as followed (line 301-310): “Finally, to investigate the impact of P188A+P195S mutations upon lipid A distribution, we undertook MALDI-TOF to analyse the abundance of modified or unmodified lipid A purified from MCR-1 or M6 -bearing cells. we found that higher abundance of unmodified lipid A was detected than that of the modified lipid A in the spheroplasts expressing MCR-1, which was in line with distribution of lipid A in the whole cells of MCR-1 -bearing strains (Figure S13E). Inversely, the abundance of modified lipid A was higher than that of the unmodified lipidA in the M6 -bearing spheroplasts. Taken together, these results indicated that the P188A+P195S mutations enhanced the binding affinity towards lipid A of MCR-1, and potentially resulted in accumulation of modified lipid A on the bacterial IM.”

”

Additionally, the reduced LPS level on the inner membrane of M6 appeared as a conflict with the reduced PI signal of M6 -bearing *E. coli*. It can be explained by the protective effect from mediated by the L, D-transpeptidases (LDTs) pathway, which enables the PG layer remodeling during membrane damage[3]. Significant up-regulation in the transcriptional levels of genes belonging to the LDTs pathway was observed in M6 -bearing strain, indicating the enhanced expression of such pathway due to the expression of M6. Meanwhile, after the deletion of *mrcB*, a critical gene for the LDTs pathway[3], the PI absorption of M6 -bearing strain increased to the same as that of the *mcr-1*^+^ strain, but no significant change was observed for those of control and MCR -bearing cells. Meanwhile, previous research also demonstrated that the LDTs-mediated PG layer remodeling acts as a protection during OM LPS transferring defect[3]. And the activation of LDTs pathway enables the defense against detergent like SDS[4], which also been proved in our current research.

To further confirmed the IM integrity of M6 -bearing *E. coli*, we verify the PI-positive proportion of the M6 -expressing spheroplasts by using flow-cytometry. We found that after the removal of OM and PG layer, the permeability of M6 -bearing *E. coli* was significantly increased, demonstrating the reduced IM integrity of the target strain. The results have been added into the manuscript as Figure S9 as followed:

**Figure S9. Spheroplasts LPS level perturbation of MCR-1 or M6 -bearing *E. coli*.**

**(A)** Morphology observation of spheroplasts for indicated strains. The shape of *E. coli* was changed from rob-shape into ball-shape, representing successful preparation of spheroplasts.

**(B-C)** The inner membrane permeability of indicated spheroplasts were evaluated by PI staining assay. The well-prepared spheroplasts were incubated with PI dye for 15 min. The PI-positive proportion was determined by flow cytometry and analysed by FlowJo version 10 software.

All the above-described experiments were performed three times with similar results. Error bars indicate standard errors of the means (SEMs) for three biological replicates. A two-tailed unpaired *t* test was performed to determine the statistical significance of the data. ns, no significant difference; *, *P*< 0.1; ****, *P*< 0.0001.

And the demonstration of above result has been added into the manuscript as followed (line 237-240): “To further verify the IM integrity of M6 –bearing strain, the spheroplasts was generated by removing the OM and PG layer and was confirmed through the observation with microscopy (Figure S9A). Remarkably, the M6 –bearing spheroplasts exhibited increased PI-positive proportion than the whole cells (Figure S9B-C), demonstrating reduced IM integrity of M6 –expressing cells.”

Taken together, the expression of M6 resulted in accumulation of PEA modified lipid A on the bacterial IM and reduced cellular LPS level, especially those on IM. This impact reduced the integrity of bacterial IM, which further activated the LDTs-pathway and increased the transcriptional levels of genes related to this pathway. The up-regulation of the LDTs pathway maintained the IM barrier function of M6 -bearing strain.

Q2: Line 121: this section is unclear. Do the authors mean a defect in OM permeability (as written)? Ref. 30 suggests that what is meant is an increase.

Reply: We sincerely thanks the suggestion from reviewer and sorry for our mistake. Indeed, the expression of MCR-1 increases the permeability of bacterial membrane based on our previous research. And we have corrected the related sentence as followed (line 118-119): “Our recent research has suggested that MCR-1 induces membrane lipid remodelling, leading to a compromise in the OM integrity as well as bacterial viability[5].”

Q3: Figure legends throughout lack detail. It should be possible to interpret Figures based upon the legend and without extensive reference to the main text. Figures 1B, 1E, 2F, 2G and 3D, E, F, G and H are just some of multiple examples where the legend lacks sufficient detail to make clear exactly what is being shown.

Reply: We sincerely thanks the suggestion from reviewer. And the indicated legends have been corrected as followed:

Figures 1B: “The logarithmic phase cultures of *E. coli* BW25113 carrying empty vector, M6, or MCR-1 were collected to evaluate the sensitivity towards CT and β-lactam antibiotics (AMP, FOX, IMP, CAZ, CTX and MEM) by agar dilution MICs tests. Each triangle represents an independent experiment. And the experiments were performed three times with same results.”

Figure 1E: “The OM integrity of strains carrying M6 or MCR-1 was determined by measuring NPN uptake during logarithmic phase. And the fluorescent signal for each sample was immediately monitored with a microplate reader at an excitation wavelength of 350 nm and emission wavelength of 420 nm after staining.”

Figure 2F-G: “The logarithmic phase cultures of *E. coli* BW25113 carrying empty vector, M6, or MCR-1 were collected. And the cellular levels of PbgA and LPS of indicated strains were determined by western blot after cell lysis with ultrasonication.”

Figure 3D: “Estimate the free energy of binding of lipid A to WT MCR-1 and M6. The results show that the M6 mutant has a better binding free energy compared to WT. Where the binding free energy was calculated using the MM-GBSA for the last 200 ns trajectory after the protein-ligand complex reached equilibrium.”

Figure 3E-F: “The affinity between lipid A substrate and linker domain of MCR-1/M6 was determined by biolayer interferometry. The synthetic peptides derived from the linker domain of WT MCR-1 or M6 (named peptide MCR-1 and peptide M6, respectively) were coated on the surface of streptavidin (SA) biosensor biosensors. Target peptides were bound to Kdo_2_-lipid A in series of concentrations (150, 100, 50, 25 and 10 μM) with 300 s association and dissociation steps. Assays were performed in triplicate on an Octet Red384 (Sartorius).”

Figure 3G-H (have been moved to Figure S6A-B): “**(A)** The fluorescence ratio indicates the relative membrane voltage in indicated *E. coli* cells by using a genetically-encoded voltage sensor Vibac2, which is a double-channel fusion protein that emits green and red fluorescence. The fluorescence intensity of GFP (Ig, Excitation = 488 nm, Emission = 512 nm) responds to membrane voltage, and the mCherry intensity (Ir, Excitation = 561 nm, Emission = 610) is used to normalize protein expression. Thus, the fluorescence ratio (Ig/Ir) indicates the relative membrane voltage in *E. coli* cells. To evaluation the impact of MCR-1 or M6 expression on the membrane voltage, a pBAD24 plasmid with the gene encoding Vibac2 was transformed into the indicated strains, and the fluorescence ratio (Ig/Ir) for each strain were measured and calculated during logarithmic phase. For each strain, 300 isolates were analysed. And the representative GFP and mCherry images of BW25113 cells carrying empty plasmid, MCR-1 or M6 were represented as **(B)**.”

Q4: Line 200: clarify that while M6 expression clearly disrupts the OM this is also observed for wt MCR-1.

Reply: We thank the reviewer for pointing this out. Our previous study shown that MCR-1 causes lipid remodelling and further results in an OM permeability defect[5]. In comparison with WT MCR-1, M6 expression resulted in more severe OM damage. We have corrected the related sentence in the manuscript as followed (line 190-192): “Moreover, by employing transmission electron microscope (TEM), we found that M6 expression clearly induced noticeable interspace within the periplasm at one pole of the cell (Figure 2B), indicating cytoplasmic shrinkage.”

Q5: Lines 256 -7 and 275 - 6: While I agree that the observed increase in LdtD expression, and the effects of LdtD inhibition, are consistent with beta-lactam resistance in the M6-expressing strains resulting from LdtD-mediated PG remodeling, in the absence of a direct determination of PG composition this conclusion cannot be regarded as conclusive. I would suggest that the relevant sections be reworded to reflect this.

Reply: We sincerely thanks the suggestion from reviewer. We have corrected the related sentences as followed:

Line 240-242: “Overall, these results indicated that PBP1B was essential for phenotypic co-resistance and IM barrier function in M6 –bearing *E. coli*.”

Line 257-258: “Taken together, these results demonstrate that severe lipid A defect induced by M6 expression conferred reduced sensitivity to β-lactam antibiotics.”

Q6: Lines 262 - 264: were changes in PbgA expression evident from the proteomics experiments?

Reply: We sincerely thanks the suggestion from reviewer. Yes, based on the result of proteomics analysis, the PbgA (also named YejM) expression level in M6 –bearing *E. coli* is 1.6667-fold of the one in the strain expressing WT MCR-1. Such result is included in Table S4.

Q7: Figure 3C axis labels: surely NPN uptake measures OM, and PI IM, integrity, and not the reverse as shown?

Reply: We sincerely thanks the suggestion from reviewer and sorry for our mistake. We have corrected the related figure as followed:

Q8: Lines 317 - 319 and Figure 3: Which peptides are being used in these experiments? These should be detailed in the main text, as well as Methods.

Reply: We sincerely thanks the suggestion from reviewer and sorry for our mistake. The peptides mentioned in this part were peptide MCR-1 and peptide M6, which were included in Table S10. The demonstration of the related section has been corrected as followed (line 296-298): “To further confirm that this pocket facilitates the lipid A binding affinity, we employed synthetic peptides encompassing the pocket of MCR-1 and M6, named peptide MCR-1 (KPLRSYVNPIMPIYSV) and peptide M6 (KALRSYVNSIMPIYSV), to interact with LPS.”

Meanwhile, details about the indicated peptides have been added into the methods (line 310-312 of supplementary materials): “An *in vitro* interaction assay was carried out to verify the affinity between lipid A and synthetic peptides derived from the linker domain of WT MCR-1 and M6, named peptide MCR-1 and peptide M6, respectively (Table S10). Both peptides were labelled with biotin at the C-terminals.”

Q9: lines 320 - 22: This is unclear. Is membrane potential what is being measured here?

Reply: We sincerely thanks the suggestion from reviewer. The bacterial membrane potential is determined by the intracellular and extracellular concentration gradients of sodium (Na^+^) and potassium (K^+^)[6]. The sensor we applied here is a genetically encoded bacterial sensor which allows single-cell recording of bacterial membrane potential dynamics, which was advanced from a membrane voltage sensitive probe Arclight[7]. It consists of the voltage sensing domain of *Ciona intestinalis* voltage-sensitive phosphatase and super ecliptic pHluorin, which is an EGFP sensitive to acidic pH[8, 9]. Therefore, the fluorescence signal from ArcLight depended on both the membrane potential (Na^+^/K^+^ concentration gradients) as well as the intracellular pH. This sensor has been convincingly used to monitor bacterial V_m_ i*n vivo*. Since membrane permeability is a critical determinant for ion gradient concentration and membrane potential, we used a novel sensitive bacterial membrane voltage V_m_ sensor to investigate the impact of this mutation on MCR-1-mediated membrane potential change.

We have moved this part of results to the section of “Severe lipid A disorder caused by M6 expression” as Figure S6, to evidence the membrane perturbation caused by M6. The figure is as followed:

**Figure S6. Membrane voltage measured by genetically-encoded sensor Vibac2.**

**(A)** The fluorescence ratio indicates the relative membrane voltage in indicated *E. coli* cells by using a genetically-encoded voltage sensor Vibac2, which is a double-channel fusion protein that emits green and red fluorescence. The fluorescence intensity of GFP (Ig, Excitation = 488 nm, Emission = 512 nm) responds to membrane voltage, and the mCherry intensity (Ir, Excitation = 561 nm, Emission = 610) is used to normalize protein expression. Thus, the fluorescence ratio (Ig/Ir) indicates the relative membrane voltage in *E. coli* cells. To evaluation the impact of MCR-1 or M6 expression on the membrane voltage, a pBAD24 plasmid with the gene encoding Vibac2 was transformed into the indicated strains, and the fluorescence ratio (Ig/Ir) for each strain were measured and calculated during logarithmic phase. For each strain, 300 isolates were analysed. And the representative GFP and mCherry images of BW25113 cells carrying empty plasmid, MCR-1 or M6 were represented as **(B)**.

And the demonstration of above result has been added into the manuscript as followed (line 201-206): “Furthermore, to investigate the impact of P188A+P195S mutations upon membrane potential change, we used a novel sensitive bacterial membrane voltage V_m_ sensor, which allow single-cell recording of bacterial V_m_ dynamics in live cells, and found that this two-point mutation appeared to increase the membrane voltage of *mcr-1* bearing *E. coli* (Figure S6A-B). Since membrane permeability is a critical determinant for membrane potential, this result also suggested the disruption of membrane homeostasis caused by M6 expression.”

And the method to measure bacterial membrane potential has now been mentioned in the Materials and Methods section as “Fluorescent imaging to assess membrane voltage”, which is as followed (line 268-279 of supplementary materials): “The construction of genetically-encoded membrane voltage sensor Vibac2 was as described in previous research[7]. ViBac2 is a double-channel fusion protein that emits green and red fluorescence. The fluorescence intensity of GFP (Ex=488 nm, Em=512 nm) responds to membrane voltage, and the mCherry (Ex=561 nm, Em=610) is used to normalize protein expression. Thus, the fluorescence ratio indicates the relative membrane voltage in *E. coli* cells. The plasmids encoding MCR-1 or M6 were transformed into *E. coli* BW25113 competent cell expressing Vibac2. Strain harbouring pACYCDuet-1 empty plasmid was set as control. For each strain, stationary phase culture was collected after induction with 0.2% arabinose, and 5 μl of bacterial culture was placed on a gel pad containing 1% agarose and covered with a coverslip. The GFP and mCherry signals were recorded with a fluorescence microscope (Olympus BX63) on phase contrast equipped with a 100× oil immersion objective and a xenon lamp. The images merged with the two fluorescent signals were processed with Fuji software.”

Q10: Line 372: Can the authors argue that M6 induces OM permeabilization compared to WT MCR-1? This does not appear to be supported by the NPN uptake measurements in Figure 1E.

Reply: Thanks for pointing out this problem. NPN is a nonpolar dye that fluoresces strongly in hydrophobic environments, such as the lipid bilayer of the inner membrane or the inner leaflet of the outer membrane. The outer leaflet of the outer membrane is composed of lipopolysaccharide (LPS), which forms a tightly packed semicrystalline structure that effectively excludes both hydrophilic and hydrophobic compounds, including NPN[10]. It has been shown by several groups that the rate of NPN fluorescence increase was linearly related to the OM permeabilization[11], but the final amount of NPN uptake will be saturated with increased OM permeability[10]. Here, we observed a mark increase in NPN fluorescence in both MCR-1-expressing cells and M6-expressing cells. These results may reflect a limitation of the NPN assay to identify differences at extremely high levels of fluorescence absorption.

This part has now been added in the Discussion section as follows (line 427-429): “Finally, although the NPN uptake assay suggested that MCR-1 or M6 expression may lead to reduction in OM LPS and increasing OM permeability, development of novel methodology is required to distinguish the permeability of bacterial IM and OM precisely.”

**#Reviewer 2**

This manuscript by Tian and collaborators identifies a potential mechanism explaining membrane changes and fitness cost caused by the expression of the plasmid encoded Mcr1 ethanolamine transferase enzyme, which is responsible for inducing colistin resistance in Gram-negative bacteria. The authors describe a Mcr1 variant (M6) that causes outer membrane perturbation, increased resistance to beta-lactam antibiotics (co-resistance) and modifications in the cell wall peptidoglycan. The authors propose, based on modelling that the region modified in the M6 variant forms a cavity that accommodates lipid A and perturbs the affinity of the interaction, which in turn leads to alteration of lipid A levels and downstream effects related to cell permeability and PG defects. The authors also designed a peptide based on the linker sequence that has antimicrobial activities possibly by affecting lipid A. Overall, this manuscript has interesting results that advance the field.

Sincerely thanks for your appreciation of our research.

I have several major concerns that require attention:

Q1: The work is based on the overexpression of the MCR-1 protein and its derivatives. pACYCDuet is a plasmid of medium copy number (~20-50 copies per chromosome) and the cloned genes are expressed by a T7 promoter, which is highly efficient. These levels of expression are very high relative to the natural expression of MCR-1. Because MCR-1 is a membrane protein and membrane proteins are well known to induce pleiotropic effects when expressed, it would be important that the observed phenotypes are replicated under conditions of MCR-1 natural expression.

Reply: We sincerely thanks the suggestion from reviewer. Actually, we modified the plasmid backbone of pACYCDuet by changing the promoter into the one induced by arabinose. Therefore, the expression of M6 or WT MCR-1 required the addition of 0.2% arabinose as we mentioned in methods. Moreover, we also constructed a derivative plasmid that the expression of MCR-1 was regulated by its native promoter. The indicated plasmid map has been added into the manuscript as Figure S19, which are as followed:

**Figure S19. Plasmid maps of pACYC-Para-MCR-1 and pACYC-NP-MCR-1.**

The plasmid for expressing MCR-1 or M6 under the regulation of arabinose promoter was shown as **(A)**, and the one for expressing target proteins under the regulation of MCR-1 native promoter was shown as **(B)**. The elements in each plasmid were as followed: CmR= chloramphenicol acetyltransferase; p15A ori= the medium-copy-number p15A origin of replication; T7 terminator= transcription terminator for bacteriophage T7 RNA polymerase; *araC*= gene encoding L-arabinose regulatory protein; araBAD promoter= promoter of the L-arabinose operon; MCR-1 native promoter= the native promoter of mcr-1 gene amplified from clinically collected *mcr-1*^+^ strain; WT MCR-1/M6= the gene encoding WT MCR-1 or its two-points mutant M6.

Also, the process for the construction of pACYC-NP-*mcr-1* has been added into methods (line 31-32 of supplementary materials): “Similar strategy was also applied for the construction of pACYC-NP-*mcr-1*.”

According to the reviewer’s suggestions, we also evaluated the permeability of IM and OM as well as sensitivity towards β-lactams antibiotics of *E. coli* harboring pACYC-NP-*mcr-1*/M6. The results have been added into the section of “A novel MCR-1 variant, M6, induced co-resistance towards β-lactam antibiotics” as Figure S4 as followed:

**Figure S4. Co-resistance and membrane perturbation induced by native promoter-M6.**

pACYCDuet-1 carrying MCR-1 or M6 under the regulation of the MCR-1 native promoter (NP MCR-1 or NP M6) was generated. Overnight cultures were sub-cultured into fresh LB broth at a ratio of 1:100. For the gene under the regulation of arabinose promoter, addition of 0.2% arabinose was required for protein expression induction. Logarithmic phase cultures were collected for following assays.

**(A**) The sensitivity of indicated strains towards CT and β-lactam antibiotics (AMP, FOX and CAZ) were evaluated by agar dilution MIC tests. Each triangle represents an independent experiment. The experiments were performed three times with similar results. NP, native promoter.

**(B)** Efficiency of plating assays on LB agar plates containing 0.1% SDS and 1 mM EDTA. Ten-fold serial-dilution of indicated cultures were inoculated onto the agar plates.

**(C)** The outer membrane integrity of indicated strains were determined by measuring NPN uptake during logarithmic phase. And the fluorescent signal for each sample was immediately monitored with a microplate reader at an excitation wavelength of 350 nm and emission wavelength of 420 nm after staining.

**(D-E)** The inner membrane permeability of indicated strains were evaluated by PI staining assay. Overnight cultures were sub-cultured into fresh LB broth at a ratio of 1:100. After cultivation for 8 hr, cultures were collected, respectively, followed by staining with PI dye for 15 min. The PI-positive proportion was determined by flow cytometry and analysed by FlowJo version 10 software.

All the above-described experiments were performed thrice with similar results. Error bars indicate standard errors of the means (SEMs) for three biological replicates. A two-tailed unpaired *t* test was performed to determine the statistical significance of the data. ns, no significant difference; **, *P*< 0.01; ***, *P*< 0.001.

Briefly, we found that M6 expression controlled by MCR-1 native promoter also resulted in phenotypic co-resistance and membrane permeabilization. The results were mentioned in the manuscript as follows.

Line 159-161: “This phenotype was further confirmed by regulating M6 expression with MCR-1 native promoter (Figure S4A).”

Line 176-177: “Consistently, similar phenotypes were observed when M6 expression was controlled by MCR-1 native promoter (Figure S4B-E).”

Q2: Can the authors attribute the antimicrobial effects of the peptides to the identified mechanism of the linker region binding to lipid A. The peptides are provided to cells at micro-molar concentrations, which are likely not achievable clinically. Also, how do they enter bacterial cells? Is the entry of the peptides related to the membrane integrity defect induced by MCR-1 (or M6)? or they are inherently permeable to the OM? (Unlikely given their mass)

Reply: We sincerely thanks the suggestion from reviewer. Generally, antimicrobial peptides kill bacteria through two broad mode of actions (MOAs). In the first mechanism AMPs induce membrane disruption, leading to cell lysis and death. In the second MOA, AMPs enter cells without membrane disruption and inhibit essential intracellular functions by binding to nucleic acids or intracellular proteins[12]. Based on our result of biolayer interferometry, we found that the peptides derived from the linker region of WT MCR-1 or M6 (peptide MCR-1 and peptide M6) were both able to bind with lipid A, and peptide M6 displayed even higher affinity than the peptide MCR-1 did. Therefore, it is reasonable for us to believe that the antimicrobial peptide derived from the linker domain of M6 kills Gram-negative bacteria by disrupting the membrane integrity. To further solidify our conclusion, we labelled the C-terminal of target peptides, 24AA-2M and 19AA-2M-tag, with FITC fluorescent dye. Logarithmic phase culture of *E. coli* ATCC 25922 was treated with above peptides in the concentrations of 0.1× or 0.05× MICs, respectively, following by observation with fluorescent microscopy.

The result of fluorescent microscopy was as followed:

**Figure S14. Localization of FITC-labelled peptides in *E. coli* ATCC25922.**

Logarithmic phase culture of *E. coli* ATCC25922 was collected, and stained by FM4-64 (red) and DAPI (cyan) to indicate the localization of bacterial membrane as well as cytoplasmic chromosome. The well-stained culture was treated with FITC-labelled 24AA-2M and 19AA-2M-tag in the concentrations of 0.05× and 0.1× MICs (18.5 µM and 37.0 µM), respectively. After washing with 1× PBS, the localization of the FITC signal were determined by observation with fluorescent microscope.

The above result strongly indicated that the synthetic peptides derived from M6 combatted *E. coli* by interacting with bacterial membrane. And the demonstration of this result has been added into the manuscript as followed: line 326-329, “Moreover, by labelling the both indicated peptides with FITC fluorescent dye at the C-terminal, we found that the FITC signal was overlapped with the signal of membrane specific dye FM4-64 (Figure S14), which was in line with the previous observations of the antimicrobial peptides functioned by interacting with bacterial membrane[13].”

The process for microscopy observation in detail is as followed and has been added into the section of methods as “Localization of synthetic peptide in *E. coli*” (line 324-335 of supplementary materials): “Fresh single colonies of *E. coli* ATCC25922 was inoculated into fresh LB broth and cultivated overnight at 37 °C. The overnight cultures were then sub-cultured into 1 ml of LB broth with shaking at 37 °C. Logarithmic phase cultures were harvested by centrifugation (4,000 rpm for 3 min), and washed twice with 1× PBS. After resuspension with 1 ml 1× PBS, 2 µl FM4-64 (for membrane staining) were added. The mixtures were then incubated at dark for 1 hr. After washing twice with 1× PBS, the resuspended cultures were then stained with 1 ml DAPI (for nucleic acids staining) for 15 min. Next, after washing twice with 1× PBS, 20 µl or 10 µl FITC-labelled peptides (10 mg/ml) were the added into the resuspended cultures. After incubation at dark for 2 hr, the mixtures were washed twice with 1× PBS, following by resuspension with 100 µl 1× PBS. The stained cells (3 µl) were spotted on a microscope slide and covered with 1% (w/v) agar slices and a glass coverslip. The localization of the FITC signal were determined by observation with fluorescent microscope (Olympus BX63).”

Moreover, as for the concentration of the indicated peptides (24AA-2M and 19AA-2M-tag), these peptides damage the membrane integrity by binding with the lipid A component, which is the major component of the outer membrane of Gram-negative bacteria[14]. Due to its large amount on the bacterial surface, it is accepted that high concentration of indicated peptides are required to damage the barrier function of OM where the lipid A is localized. In our following research, we plan to modified the structure of the peptides and improve its antimicrobial efficiency, which allows the reduction in concentration for clinical application. We will further this work in another research.

Q3: L88-89, Is the topology of the ethanolamine transfer known? According to the sentence, the modification occurs after lipid A flipping across the inner membrane at the periplasmic side of the membrane. If this is true, the PEt should also be flipped across the membrane. Can the authors clarify?

Reply: We sincerely thanks the suggestion from reviewer. The inner membrane of *Escherichia coli* contains 70–80% phosphatidylethanolamine (PE), which is the major lipid component of bacterial inner membrane[15, 16]. PE is exclusively synthesized by phosphatidylserine decarboxylase (PSD) on the cytoplasmic side of the inner membrane[17]. It has been proved that MsbA lipid flippase of *E. coli* is involved in lipid export and presumably in flipping lipids from the inner layer to the outer layer of the cytoplasmic membrane, including both phospholipids (e.g. phosphatidylethanolamine) and LPS[18]. Previous research has shown that accumulation of newly synthesized PE as well as lipid A were detected in the inner face of the inner membrane following deletion or inactivation of MsbA[18, 19]. Meanwhile, the MsbA-mediated PE flippase activity was also observed in *E. coli* membrane vesicles and reconstituted proteoliposomes by *in vitro* assay[20]. Since MCR-1 anchored at the outer leaflet of inner membrane, the lipid flippase activity of MsbA allows the occurrence of MCR-1 -mediated lipid A modification in periplasm. And the relative demonstration has been added into the manuscript as followed (line 98-99): “Phosphatidylethanolamine (PE) is the major lipid component of IM, flipped by MsbA[16, 18].”

Q4: L110, the lipid A core is transported to the outer leaflet of the OM and the consequence of this is the formation of an asymmetrical OM lipid bilayer. But the transport is not set to "establish OM asymmetry". Asymmetry also depends on the movement of phospholipid from the outer leaflet of the OM to the cell membrane by the Mla system. Please clarify sentence.

Reply: We sincerely thanks the suggestion from reviewer. The maintenance of outer membrane lipid asymmetry (Mla) system mediates retrograde phospholipid transport from the outer membrane to the inner membrane in Gram-negative bacteria[21]. Lipid asymmetry is established by having phospholipids confined to the inner leaflet of the membrane and lipopolysaccharides (LPS) to the outer leaflet[22]. And this system maintains the lipid asymmetry in the outer membrane (OM) of *E. coli*, and such lipid arrangement is essential for the barrier function of the OM and for the viability of most Gram-negative bacteria[23].

We have corrected the related demonstration as followed (line 109-110): “Guaranteed by the maintenance of lipid asymmetry (Mla) system, LPS are transported from the IM to the OM and distribute asymmetrically on the outer leaflet of the OM[24].”

Q5: L114-117, MCR-1 is a membrane protein; many membrane proteins alter bacterial fitness when overexpressed. Is the fitness defect detectable when MCR-1 is expressed under normal conditions?

Reply: We sincerely thanks the suggestion from reviewer. As the results shown in Figure S4C-E, increased IM and OM permeability was also observed for *E. coli* carrying NP-*mcr-1* (NP, native promoter), which established that expression of MCR-1 was able to induce membrane damage even under normal condition. Moreover, our previous research also proved that, under the regulation of native promoter, the expression of MCR-1 disrupts lipid A homeostasis and further resulted in membrane damage as well as cell death[5]. And this phenotype was also observed in *E. coli* carrying IncX4 or IncHI2 plasmid, the major plasmid types driving the dissemination of *mcr-1*[5]. In addition, reduced fitness was also observed from clinically isolated *mcr-1*^+^ strains[25]. Therefore, the fitness defect is detectable when MCR-1 is expressed under normal conditions.

Q6: L127-128, according to the results in Fig 1, especially Fig1B, the M6 variant confers more resistance to beta-lactams and other antibiotics.

Reply: We sincerely thanks the suggestion from reviewer. We have corrected the related demonstration as followed (line 125-126): “Such variant possessed two mutations (P188A and P195S) within the linker domain, resulting in a co-resistance towards β-lactam antibiotics.”

Q7: L131-132, revise the sentence; it is confusing.

Reply: We sincerely thanks the suggestion from reviewer. The related demonstration has been corrected as followed: line 127-130, “Moreover, we identified a lipid A loading pocket located within the linker domain of MCR-1, and the two-points mutation in M6 might increase the binding affinity upon lipid A. Additionally, synthetic peptides derived from the linker domain of M6, which appeared as “a potential cure” to combat drug-resistant bacteria.”

Q8: L142-144, this needs to be explained better: the authors report a library of 171,169 mutant genotypes? I suppose they mean amino acid replacement mutants.

Reply: We are sorry for this confusing description. We would like to clarify that the *mcr-1* variants library we constructed contains a total of 171,769 variants, covering 99.96% of the single-nucleotide mutations. Please refer to the pre-printed article for details[26]. The related demonstration has been corrected as followed (line 140-142): “We tested this hypothesis using our previously established *mcr-1* mutant library, which encompassed 171,769 mutation genotypes[26], covering 99.96% (4858/4860) possible single-nucleotide mutations of *mcr-1*.”

Q9: L146-160, the screening needs to be better explained since the text is confusing. The authors appeared to have used a library of mutant plasmids and select for variants that confer resistance to beta-lactams. Further sequencing identified M6; is this correct?

Reply: Yes, we have revised this part as followed (line 146-153): “we observed that some colonies from MCR-1 library were able to grow on the plates containing penicillin (ampicillin, AMP), cephalosporin (ceftazidime, CAZ) or carbapenem (imipenem, IMP) antibiotics, but not for the control strains (Figure S1B). Moreover, we confirmed that the MCR-1 library indeed exhibited higher viability than the control strains after exposure to β-lactam antibiotics (Figure S1C and Table S2). Next, 50 isolates of the MCR-1 library growing on the plates containing 2 x MICs CAZ or AMP were selected, and the *mcr-1* genotypes of the selected isolates were identified by Sanger sequencing (Table S3).”

We also modified the “Screening of the MCR-1 mutant library against a variety of antibiotics” in the method section as follows (line 146-179 of supplementary materials): “The MCR-1 mutant library in the background of *E. coli* BW25113 constructed in our previous research was applied for antibiotics susceptibility testing to screen out MCR-1 mutant exhibited co-resistance upon other classes of antibiotic. The indicated mutant library was cloned into the plasmid backbone of pACYCDuet-1. The expression of target pool was regulated by arabinose promoter and required the induction with 0.2% arabinose. This library contains a total of 171,769 variants for the *mcr-1* gene, which covers 99.96% of the single-nucleotide mutations. The bacterial strain was kept in a final concentration of 30% glycerol at -80°C. Unless otherwise stated, the bacterial glycerol stocks were revived through cultivation in LB broth at 37°C with shaking. The *E. coli* strains carrying empty plasmid or WT MCR-1 were set as control. Total 13 types of antibiotics were selected for testing: ampicillin (AMP), ceftazidime (CAZ), imipenem (IMP), streptomycin (SM), tetracycline (TET), nalidixic acid (NAL), vancomycin (VAN), cefotaxime (CTX), cefepime (FEP), ceftriaxone (CRO), ertapenem (ETP), meropenem (MEM) and cefoxitin (FOX). LB agar plate containing above antibiotics in the concentrations of 0.8×, 1× or 2× MICs were prepared (Table S1 and Table S2), respectively. To ensure that all the mutation genotypes in the MCR-1 mutant library were present on each plate at least once, the coupon collector problem was applied as follows:

$$M=-N\frac{log(N)}{log(P)}$$

where *M* represents the number of cells on each plate, *N* is the number of total mutation genotypes and *P* is the probability of coverage for all the genotypes.

For P = 99.9%:

$M=-4858\times\frac{\log\left( 4858 \right)}{\log\left( 0.999 \right)}=$4.2×10^9^

Therefore, approximately 1×10^11^ cells were plated on each agar plate to achieve 99.9% coverage of all the single-point mutation genotypes. Same amounts of cells were also plated on the LB agar plates containing indicated antibiotics for the groups of empty plasmid control and WT MCR-1. After incubation at 37 °C overnight, CFUs were counted to evaluate viability.

To further verify the genotype of the MCR-1 mutant displaying co-resistance towards β-lactams antibiotics, 50 isolates of the MCR-1 library were selected from the plates containing CAZ or AMP in the concentration of 2x MICs. The plasmids harbouring *mcr-1* gene were extracted from the selected isolates. Next, to remove the interference caused by chromosomal mutation, the well-extracted plasmids were transformed into *E. coli* BW25113 competent cell for generating reconstructed strains. Meanwhile, the *mcr-1* genotypes of selected isolates were confirmed by Sanger-sequencing. Additionally, the reconstructed strains were applied for susceptibility testing among CAZ, AMP and FOX as mentioned above. The *E. coli* strains carrying empty plasmid or WT MCR-1 were set as control, CFUs were counted to evaluate viability after incubation at 37 °C overnight.”

Q10: L161-168, another confusing section: the authors show that M6 mutant is more resistant to the beta-lactams, also in Table S3, but in the text they say in L165 "lower sensitivity".

Reply: We sincerely thanks the suggestion from reviewer. We have corrected the related sentence as followed (line 157-159): “Compared to MCR-1-expressing cells, this mutant displayed increased resistance towards several β-lactam antibiotics and maintained resistance towards colistin (Figure 1B and Table 1), but not for other types of antibiotics (Figure S3A).”

Q11: L171-186, the permeability assays should be done with the genes expressed at its normal dosage. pACYC184 is a plasmid of moderate copy number and the authors are consequently overexpressing a membrane protein.

Reply: We sincerely thanks the suggestion from reviewer. We have followed your comments and conducted related experiments with results as followed:

**Figure S4. Co-resistance and membrane perturbation induced by native promoter-M6.**

pACYCDuet-1 carrying MCR-1 or M6 under the regulation of the MCR-1 native promoter (NP MCR-1 or NP M6) was generated. Overnight cultures were sub-cultured into fresh LB broth at a ratio of 1:100. For the gene under the regulation of arabinose promoter, addition of 0.2% arabinose was required for protein expression induction. Logarithmic phase cultures were collected for following assays.

**(A**) The sensitivity of indicated strains towards CT and β-lactam antibiotics (AMP, FOX and CAZ) were evaluated by agar dilution MIC tests. Each triangle represents an independent experiment. The experiments were performed three times with similar results. NP, native promoter.

**(B)** Efficiency of plating assays on LB agar plates containing 0.1% SDS and 1 mM EDTA. Ten-fold serial-dilution of indicated cultures were inoculated onto the agar plates.

**(C)** The outer membrane integrity of indicated strains were determined by measuring NPN uptake during logarithmic phase. And the fluorescent signal for each sample was immediately monitored with a microplate reader at an excitation wavelength of 350 nm and emission wavelength of 420 nm after staining.

**(D-E)** The inner membrane permeability of indicated strains were evaluated by PI staining assay. Overnight cultures were sub-cultured into fresh LB broth at a ratio of 1:100. After cultivation for 8 hr, cultures were collected, respectively, followed by staining with PI dye for 15 min. The PI-positive proportion was determined by flow cytometry and analysed by FlowJo version 10 software.

All the above-described experiments were performed thrice with similar results. Error bars indicate standard errors of the means (SEMs) for three biological replicates. A two-tailed unpaired *t* test was performed to determine the statistical significance of the data. ns, no significant difference; **, *P*< 0.01; ***, *P*< 0.001.

Briefly, we found that M6 expression controlled by MCR-1 native promoter also resulted in phenotypic co-resistance and membrane permeabilization. The results were mentioned in the manuscript as follows.

Line 159-161: “This phenotype was further confirmed by regulating M6 expression with MCR-1 native promoter (Figure S4A).”

Line 176-177: “Consistently, similar phenotypes were observed when M6 expression was controlled by MCR-1 native promoter (Figure S4B-E).”

Q12: L181-182, NPN uptake. The uptake is increased in the MCR-1 and M6 to high levels compared to the plasmid control, but the PI uptake indicate differences between MCR-1 and M6. These results may reflect a limitation of the NPN assay to identify differences at higher levels of fluorescence absorption. Was the assay linear?

Reply: We fully agree with you. It has been shown by several groups that the rate of NPN fluorescence increase was linearly related to the OM permeabilization, but the final amount of NPN uptake will be saturated[10].

Q13: L188-189, Why the authors conclude M6 confers a low level of co-resistance? They have shown that resistance to beta-lactams is increased?

Reply: We sincerely thanks the suggestion from reviewer and sorry for our mistake. We have corrected the related sentence as followed (line 181-182): “Overall, these results revealed that M6 conferred a co-resistance towards β-lactam antibiotics via an unexpected mechanism that impacted membrane integrity.”

Q14: L265-267, is the OM permeability increased or decreased?

Reply: We sincerely thanks the suggestion from reviewer. We have corrected the related sentence as followed (line 249-251): “These results demonstrated that the expression of M6 led to significant reduction in cellular lipid A level, which might be responsible for the increased permeability of both OM and IM.”

Q15: L346, the authors tested the susceptibility of the strains to the peptide, not the other way around.

Reply: We respectfully disagree with your view that our data was not enough to prove the efficiency of the target peptides. To test the efficiency of the peptide, we performed bacteria killing assay, drug susceptibility test, cytotoxicity test, and animal model experiment, since it has been convincingly shown that these experiments was used to test the efficiency of potential drugs [27-29]. Importantly, we evaluated the antimicrobial efficiency of the target peptides as shown in Figure 4 and Figure S15-S17.

**Suggested editorial changes**

Below is a list of the most obvious edits required. However, overall, the manuscript is verbose and requires careful editing to improve clarity and readability.

Reply: We fully agree with this reviewer’s comments and have rewrote the whole manuscript to shorten the length and improve the readability.

Q16: L80, L120, L217 delete "Notably," here and throughout the manuscript.

Reply: It has been corrected as suggested throughout the manuscript.

Q17: L87, replace "bound" by "embedded."

Reply: It has been corrected as suggested (line 88).

Q18: L88, delete

Reply: It has been removed from the manuscript as suggested.

Q19: L113, replace "impact", by "affects"

Reply: It has been corrected as suggested (line 112).

Q20: L118, delete "Subsequently,"

Reply: It has been removed from the manuscript as suggested.

Q21: L131, delete "Strikingly,"

Reply: It has been removed from the manuscript as suggested.

Q22: L144-145, delete "To decrease..." until the comma and start with "BW25113..."

Reply: It has been removed from the manuscript as suggested.

Q23: L173-174, replace "To do it", by "To confirm this"

Reply: It has been corrected as suggested (line 166).

Q24: L196, replace "target" by "selected"

Reply: The indicated sentence has been removed to improve readability.

Q25: L232, delete "kind of"

Reply: It has been removed from the manuscript as suggested.

Q26: L240, replace "the survive" by "survival"

Reply: It has been corrected as suggested (line 225).

**References**

1.	Sabnis A, Hagart KL, Klockner A, Becce M, Evans LE, Furniss RCD, et al. Colistin kills bacteria by targeting lipopolysaccharide in the cytoplasmic membrane. Elife. 2021;10. Epub 2021/04/07. doi: 10.7554/eLife.65836. PubMed PMID: 33821795; PubMed Central PMCID: PMCPMC8096433.

2.	El Hamidi A, Tirsoaga A, Novikov A, Hussein A, Caroff M. Microextraction of bacterial lipid A: easy and rapid method for mass spectrometric characterization. J Lipid Res. 2005;46(8):1773-8. Epub 2005/06/03. doi: 10.1194/jlr.D500014-JLR200. PubMed PMID: 15930524.

3.	More N, Martorana AM, Biboy J, Otten C, Winkle M, Serrano CKG, et al. Peptidoglycan Remodeling Enables Escherichia coli To Survive Severe Outer Membrane Assembly Defect. mBio. 2019;10(1). Epub 2019/02/07. doi: 10.1128/mBio.02729-18. PubMed PMID: 30723128; PubMed Central PMCID: PMCPMC6428754.

4.	Peters K, Pazos M, Edoo Z, Hugonnet JE, Martorana AM, Polissi A, et al. Copper inhibits peptidoglycan LD-transpeptidases suppressing beta-lactam resistance due to bypass of penicillin-binding proteins. Proc Natl Acad Sci U S A. 2018;115(42):10786-91. Epub 2018/10/03. doi: 10.1073/pnas.1809285115. PubMed PMID: 30275297; PubMed Central PMCID: PMCPMC6196517.

5.	Feng S, Liang W, Li J, Chen Y, Zhou D, Liang L, et al. MCR-1-dependent lipid remodelling compromises the viability of Gram-negative bacteria. Emerg Microbes Infect. 2022;11(1):1236-49. Epub 2022/04/20. doi: 10.1080/22221751.2022.2065934. PubMed PMID: 35437117; PubMed Central PMCID: PMCPMC9067951.

6.	Benarroch JM, Asally M. The Microbiologist's Guide to Membrane Potential Dynamics. Trends Microbiol. 2020;28(4):304-14. Epub 2020/01/19. doi: 10.1016/j.tim.2019.12.008. PubMed PMID: 31952908.

7.	Jin X, Zhang X, Ding X, Tian T, Tseng CK, Luo X, et al. Sensitive bacterial V(m) sensors revealed the excitability of bacterial V(m) and its role in antibiotic tolerance. Proc Natl Acad Sci U S A. 2023;120(3):e2208348120. Epub 2023/01/10. doi: 10.1073/pnas.2208348120. PubMed PMID: 36623202; PubMed Central PMCID: PMCPMC9934018.

8.	Jin L, Han Z, Platisa J, Wooltorton JR, Cohen LB, Pieribone VA. Single action potentials and subthreshold electrical events imaged in neurons with a fluorescent protein voltage probe. Neuron. 2012;75(5):779-85. Epub 2012/09/11. doi: 10.1016/j.neuron.2012.06.040. PubMed PMID: 22958819; PubMed Central PMCID: PMCPMC3439164.

9.	Kang BE, Lee S, Baker BJ. Optical consequences of a genetically-encoded voltage indicator with a pH sensitive fluorescent protein. Neurosci Res. 2019;146:13-21. Epub 2018/10/21. doi: 10.1016/j.neures.2018.10.006. PubMed PMID: 30342069; PubMed Central PMCID: PMCPMC6470055.

10.	Loh B, Grant C, Hancock RE. Use of the fluorescent probe 1-N-phenylnaphthylamine to study the interactions of aminoglycoside antibiotics with the outer membrane of Pseudomonas aeruginosa. Antimicrob Agents Chemother. 1984;26(4):546-51. Epub 1984/10/01. doi: 10.1128/AAC.26.4.546. PubMed PMID: 6440475; PubMed Central PMCID: PMCPMC179961.

11.	Yan J, Wang K, Dang W, Chen R, Xie J, Zhang B, et al. Two hits are better than one: membrane-active and DNA binding-related double-action mechanism of NK-18, a novel antimicrobial peptide derived from mammalian NK-lysin. Antimicrob Agents Chemother. 2013;57(1):220-8. Epub 2012/10/24. doi: 10.1128/AAC.01619-12. PubMed PMID: 23089755; PubMed Central PMCID: PMCPMC3535945.

12.	Benfield AH, Henriques ST. Mode-of-Action of Antimicrobial Peptides: Membrane Disruption vs. Intracellular Mechanisms. Front Med Technol. 2020;2:610997. Epub 2020/12/11. doi: 10.3389/fmedt.2020.610997. PubMed PMID: 35047892; PubMed Central PMCID: PMCPMC8757789.

13.	Lee H, Lim SI, Shin SH, Lim Y, Koh JW, Yang S. Conjugation of Cell-Penetrating Peptides to Antimicrobial Peptides Enhances Antibacterial Activity. ACS Omega. 2019;4(13):15694-701. Epub 2019/10/02. doi: 10.1021/acsomega.9b02278. PubMed PMID: 31572872; PubMed Central PMCID: PMCPMC6761801.

14.	Bertani B, Ruiz N. Function and Biogenesis of Lipopolysaccharides. EcoSal Plus. 2018;8(1). Epub 2018/08/02. doi: 10.1128/ecosalplus.ESP-0001-2018. PubMed PMID: 30066669; PubMed Central PMCID: PMCPMC6091223.

15.	Murzyn K, Rog T, Pasenkiewicz-Gierula M. Phosphatidylethanolamine-phosphatidylglycerol bilayer as a model of the inner bacterial membrane. Biophys J. 2005;88(2):1091-103. Epub 2004/11/24. doi: 10.1529/biophysj.104.048835. PubMed PMID: 15556990; PubMed Central PMCID: PMCPMC1305115.

16.	Bogdanov M, Pyrshev K, Yesylevskyy S, Ryabichko S, Boiko V, Ivanchenko P, et al. Phospholipid distribution in the cytoplasmic membrane of Gram-negative bacteria is highly asymmetric, dynamic, and cell shape-dependent. Sci Adv. 2020;6(23):eaaz6333. Epub 2020/06/17. doi: 10.1126/sciadv.aaz6333. PubMed PMID: 32537497; PubMed Central PMCID: PMCPMC7269648.

17.	Cho G, Lee E, Kim J. Structural insights into phosphatidylethanolamine formation in bacterial membrane biogenesis. Sci Rep. 2021;11(1):5785. Epub 2021/03/13. doi: 10.1038/s41598-021-85195-5. PubMed PMID: 33707636; PubMed Central PMCID: PMCPMC7952604.

18.	Doerrler WT, Gibbons HS, Raetz CR. MsbA-dependent translocation of lipids across the inner membrane of Escherichia coli. J Biol Chem. 2004;279(43):45102-9. Epub 2004/08/12. doi: 10.1074/jbc.M408106200. PubMed PMID: 15304478.

19.	Doerrler WT, Raetz CR. ATPase activity of the MsbA lipid flippase of Escherichia coli. J Biol Chem. 2002;277(39):36697-705. Epub 2002/07/18. doi: 10.1074/jbc.M205857200. PubMed PMID: 12119303.

20.	Guo D, Singh H, Shimoyama A, Guffick C, Tang Y, Rowe SM, et al. Energetics of lipid transport by the ABC transporter MsbA is lipid dependent. Commun Biol. 2021;4(1):1379. Epub 2021/12/11. doi: 10.1038/s42003-021-02902-8. PubMed PMID: 34887543; PubMed Central PMCID: PMCPMC8660845.

21.	Ng D, Moraes TF. Dynamic players and intricate interactions: an integrated investigation of the Mla lipid transport system. J Biol Chem. 2023:105146. Epub 2023/08/11. doi: 10.1016/j.jbc.2023.105146. PubMed PMID: 37562569.

22.	Chong ZS, Woo WF, Chng SS. Osmoporin OmpC forms a complex with MlaA to maintain outer membrane lipid asymmetry in Escherichia coli. Mol Microbiol. 2015;98(6):1133-46. Epub 2015/09/01. doi: 10.1111/mmi.13202. PubMed PMID: 26314242.

23.	Thong S, Ercan B, Torta F, Fong ZY, Wong HY, Wenk MR, et al. Defining key roles for auxiliary proteins in an ABC transporter that maintains bacterial outer membrane lipid asymmetry. Elife. 2016;5. Epub 2016/08/17. doi: 10.7554/eLife.19042. PubMed PMID: 27529189; PubMed Central PMCID: PMCPMC5016091.

24.	Yeow J, Chng SS. Of zones, bridges and chaperones - phospholipid transport in bacterial outer membrane assembly and homeostasis. Microbiology (Reading). 2022;168(4). Epub 2022/04/07. doi: 10.1099/mic.0.001177. PubMed PMID: 35384832.

25.	Ma K, Feng Y, Zong Z. Fitness cost of a mcr-1-carrying IncHI2 plasmid. PLoS One. 2018;13(12):e0209706. Epub 2018/12/27. doi: 10.1371/journal.pone.0209706. PubMed PMID: 30586457; PubMed Central PMCID: PMCPMC6306219.

26.	Guo Z, Feng S, Liang L, Li J, Zhong L-L, Zhao H, et al. The fitness landscape of the mobilized colistin resistance gene <em>mcr-1</em>. bioRxiv. 2022:2022.07.21.500813. doi: 10.1101/2022.07.21.500813.

27.	Di YP, Lin Q, Chen C, Montelaro RC, Doi Y, Deslouches B. Enhanced therapeutic index of an antimicrobial peptide in mice by increasing safety and activity against multidrug-resistant bacteria. Sci Adv. 2020;6(18):eaay6817. Epub 2020/05/20. doi: 10.1126/sciadv.aay6817. PubMed PMID: 32426473; PubMed Central PMCID: PMCPMC7195177.

28.	Mohamed MF, Abdelkhalek A, Seleem MN. Evaluation of short synthetic antimicrobial peptides for treatment of drug-resistant and intracellular Staphylococcus aureus. Sci Rep. 2016;6:29707. Epub 2016/07/14. doi: 10.1038/srep29707. PubMed PMID: 27405275; PubMed Central PMCID: PMCPMC4942614.

29.	Clairfeuille T, Buchholz KR, Li Q, Verschueren E, Liu P, Sangaraju D, et al. Structure of the essential inner membrane lipopolysaccharide-PbgA complex. Nature. 2020;584(7821):479-83. Epub 2020/08/14. doi: 10.1038/s41586-020-2597-x. PubMed PMID: 32788728.
