# Supplementary material for: A new variant of the colistin resistance gene MCR-1 with co-resistance to β-lactam antibiotics reveals a potential novel antimicrobial peptide
Source: PLoS Biol. 2023 Dec 13;21(12):e3002433. doi: 10.1371/journal.pbio.3002433 (PMC10786390; doi:10.1371/journal.pbio.3002433)
Supplement: Attachment — Submitted filename: Manuscript for revision 20230918.docx [file pbio.3002433.s036.docx]

**Title:** **Identification of a novel MCR-1 variant displaying low level of co-resistance to β-lactam antibiotics that uncovers a potential novel antimicrobial peptide**

Lujie Liang^1,2,3^*^a^, Lan-Lan Zhong^1,2,3^*, Lin Wang^4^*, Dianrong Zhou^1,2,3^, Yaxin Li^1,2,3^, Jiachen Li^1,2,3^, Yong Chen^5^, Wanfei Liang^1,2,3^, Wenjing Wei^6^, Chenchen Zhang^6^, Hui Zhao^7^, Lingxuan Lyu^1,2,3^, Nicole Stoesser^8^, Yohei Doi^9,10^, Fang Bai^4†b^, Siyuan Feng^1,2,3†c^, Guo-Bao Tian^1,2,3†d^

^1^Advanced Medical Technology Center, The First Affiliated Hospital, Zhongshan School of Medicine, Sun Yat-sen University, Guangzhou 510080, China;

^2^Department of Immunology, School of Medicine, Sun Yat-Sen University, Shenzhen, 518107, China;

^3^Key Laboratory of Tropical Diseases Control (Sun Yat-sen University), Ministry of Education, Guangzhou, 510080, China;

^4^Shanghai Institute for Advanced Immunochemical Studies and School of Life Science and Technology, Shanghai Tech University, Shanghai, China;

^5^School of Laboratory Medicine, Chengdu Medical College, Chengdu, China;

^6^Center for Tuberculosis Control of Guangdong Province, Guangzhou, Guangdong, 510630, China

^7^Laboratory Medicine, Guangdong Provincial People's Hospital, Guangdong Academy of Medical Sciences, Guangzhou, Guangdong 510000, China;

^8^Modernising Medical Microbiology, Nuffield Department of Medicine, University of Oxford, Oxford, UK;

^9^University of Pittsburgh School of Medicine, Pittsburgh, PA, USA;

^10^Department of Microbiology, Fujita Health University School of Medicine, Aichi, Japan;

^a^ORCID: 0000-0002-1659-0493

^b^ORCID: 0000-0003-1468-5568

^c^ORCID: 0000-0003-2416-0693

^d^ORCID: 0000-0001-6750-5482

*These authors contributed equally to this article.

^†^**Corresponding author:**

Guo-Bao Tian, PhD, Professor

Siyuan Feng, PhD

Fang Bai, PhD, Assistant professor

**Address for correspondence:**

**Guo-Bao Tian, PhD, Professor**

Mailing address: Department of Immunology and Microbiology, Zhongshan School of Medicine, Sun Yat-sen University, 74 Zhongshan 2^nd^ Road, Guangzhou 510080, China. Phone: +86 (0)20 87335387; Fax: +86 (0)20 87335387. Email: tiangb@mail.sysu.edu.cn.

**Siyuan Feng, PhD**

Mailing address: Department of Immunology and Microbiology, Zhongshan School of Medicine, Sun Yat-sen University, 74 Zhongshan 2^nd^ Road, Guangzhou 510080, China. Phone: +86 (0)20 87335387; Fax: +86 (0)20 87335387. Email: fsy0593@163.com.

**Fang Bai, PhD, Assistant professor**

Mailing address: Shanghai Institute for Advanced Immunochemical Studies and School of Life Science and Technology, Shanghai Tech University, Shanghai, China. Email: baifang@shanghaitech.edu.cn

**Abstract**

The emerging and global spread of a novel plasmid-mediated colistin resistance gene, *mcr-1*, threatens human health. Expression of the MCR-1 protein affects bacterial fitness and this cost correlates with lipid A perturbation. However, the exact molecular mechanism remains unclear. Here, we identified the MCR-1 M6 variant carrying two point mutations that conferred co-resistance to β-lactam antibiotics. Compared to wild-type (WT) MCR-1, this variant caused severe disturbance in lipid A, resulting in upregulation of L, D-transpeptidases (LDTs) pathway, which explains co-resistance to β-lactams. Moreover, we show that a lipid A loading pocket is localized at the linker domain of MCR-1 where these two mutations are located. This pocket governs colistin resistance and bacterial membrane permeability, and the mutated pocket in M6 enhances the binding affinity towards lipid A. Based on this new information, we also designed synthetic peptides derived from M6 that exhibit broad-spectrum antimicrobial activity, exposing a potential vulnerability that could be exploited for future antimicrobial drug design.

**Introduction**

Colistin is a last-resort antibiotic against infections caused by highly drug-resistant bacteria, particularly carbapenem-resistant *Enterobacterales*[1]. However, the emergence of a novel plasmid-mediated colistin resistance gene named *mcr-1* in 2016 threatened the clinical effectiveness of colistin. Since then, instances of *mcr-1*-positive *Enterobacterales* (MCRPE) have been detected from various sources (including livestock, humans, animal food products and the environment) and have disseminated globally, spreading to >40 countries in five continents[2-4]. Colistin has been used as an additive in livestock feed to promote growth and prevent infection in China since the 1980s, and the correlation between the spread of *mcr-1* and colistin use in animal husbandry have been observed[5]. To prevent the continued spread of plasmid-borne *mcr-1*, the Chinese government banned the use of colistin as animal feed additive in 2017. Such policy resulted in remarkable reductions in the production as well as sale of colistin sulfate premix[6], and a subsequent drastic decline in *mcr-1* prevalence. For instance, a 7-years regional surveillance survey showed that the prevalence of MCRPE in pigs and chickens gradually increased during 2015-2016, peaking at 38-45%, then dropped to a trough of <2% in 2018-2019 after the policy implement[7]. Nonetheless, a low prevalence of *mcr-1* was still detected among inpatients, likely associated with the approval of colistin for clinical use in China[5, 6, 8, 9]. Furthermore, increased co-existence of *mcr-1* and carbapenemase genes after the clinical introduction of polymyxin was recently reported[10]. Hence, there is an urgent need to develop new strategies to eliminate the spread of *mcr-1* and prolong the use of colistin/polymixins as the last-resort antibiotic against carbapenem-resistant bacteria that carry *mcr-1*.

By neutralizing the overall negative charge of the bacterial surface, MCR-1 renders the bacteria resistant to colistin. MCR-1, belonging to the YhjW/YjdB/YijP alkaline phosphatase superfamily, functions as an inner-membrane metalloenzyme with its active site exposed to the periplasmic space[11, 12]. MCR-1 comprises five predicted membrane-spanning α-helixes, forming an insoluble domain that anchors in the cytoplasmic membrane. This domain is connected to a soluble catalytic domain, which extends into the periplasm through a flexible linker region[13, 14]. The catalytic domain of MCR-1 is highly homologous with that of the *Neisseria meningitidis* phosphoethanolamine (PEA) transferase EptA. It consists of several β-α-β-α “sandwich” structures comprising an active site that relies on zinc ions for coordination[11, 15]. Both EptA and MCR-1 possess a conserved zinc-binding pocket for conferring colistin resistance. The MCR-1–mediated lipid A modification involves two-steps: (i) PE hydrolysis into diacylglycerol resulting in MCR-1-bound PEA; (ii) transfer of the PEA moiety onto lipid A[16]. Phosphatidylethanolamine (PE) is the major lipid component of IM, flipped by MsbA[17, 18]. It is established that MCR-1 possesses two putative functional domains for PE entry and a catalytic centre with active site. These features are highly conserved throughout the MCR family[19]. While the PE-interacting cavity has been assigned[20], the precise mechanism for the interplay between MCR-1 and lipid A remains unknown.

The lipid A moiety of lipopolysaccharide (LPS), the primary component of outer membrane (OM) in Gram-negative bacteria, is critical to bacterial physiology and survival[21, 22]. Lipid A is synthesized on the cytoplasmic surface of the inner membrane through a well-conserved pathway involving nine enzymes. After attaching the core oligosaccharide, lipid A is flipped to the outer leaflet of the inner membrane (IM) by the ABC transporter MsbA. Subsequently, O-antigen polymers may added in some but not all Gram-negative bacteria[23]. LPS molecules are transported from the IM to the OM and distributed asymmetrically on the outer leaflet of the OM[24], a process that requires in part maintenance of lipid asymmetry (Mla) system. The proper distribution of lipid A is crucial for maintaining the mechanical integrity of the bacterial outer membrane[25, 26]. The interaction between MCR-1 and lipid A appears to compromise bacterial physiology. Various studies have demonstrated that MCR-1 leads to reduced bacterial fitness[27-30]. For example, overexpressing MCR-1 in *Escherichia coli* caused growth defects, fitness reduction, and membrane damage[30]. Such MCR-1 related fitness cost was also observed in isolates collected from hospitals[31]. The reduced *mcr-1* prevalence after the ban on colistin-added fodders is believed to be associated with the MCR-1-mediated fitness cost in the absence of antibiotic selective pressure[6, 7, 9]. Our recent research has suggested that MCR-1 induces membrane lipid remodelling, leading to a compromise in the OM integrity as well as bacterial viability[32]. These observations raise the possibility that MCR-1 possesses a potential lipid binding pocket that disrupts the distribution of lipid A in the outer membrane. However, the precise molecular mechanism by which MCR-1 disrupts lipid homeostasis remains to be fully elucidated.

Using our previous high-throughput library of *mcr-1* mutants, we characterize here an MCR-1 variant named M6, which possesses two mutations (P188A and P195S) within the linker domain. M6 expression resulted in co-resistance towards β-lactam antibiotics. Meanwhile, the expression of M6 caused membrane lipid A perturbation, resulting in a growth defect and up-regulation of the LDTs pathway. Moreover, we identified a lipid A loading pocket located within the linker domain of MCR-1, where the two mutations in M6 reside, potentially increasing the lipid A binding affinity. Synthetic peptides derived from the M6 linker domain provided antimicrobial activity, unravelling a potentially new strategy for eliminating drug-resistant bacteria.

**Results**

**MCR-1 variant M6 induces co-resistance towards β-lactam antibiotics**

Given that MCR-1 disrupts OM integrity[32, 33] facilitating entry of various antibiotics, we hypothesized that specific mutations in *mcr-1* could abolish this phenotype. We tested this hypothesis using our previously established *mcr-1* mutant library, which encompassed 171,769 mutation genotypes[34], covering 99.96% (4858/4860) possible single-nucleotide mutations of *mcr-1*. Screening was performed at antibiotic concentrations below and above the minimal inhibitory concentration (MIC, 0.8-2× MIC) to distinguish mutants with low- and high-level resistance (Figure S1A and Table S1). By counting the colony forming unit (CFU) values evaluated from plates containing aminoglycosides (streptomycin, SM), tetracyclines (tetracycline, TET), quinolones (nalidixic acid, NAL) or glycopeptides (vancomycin, VAN), we observed that some colonies from MCR-1 library could grow on plates containing penicillin (ampicillin, AMP), cephalosporin (ceftazidime, CAZ) or carbapenem (imipenem, IMP) (Figure S1B). Moreover, we confirmed that the MCR-1 library exhibited higher viability than the control strains after exposure to β-lactams (Figure S1C and Table S2). Next, 50 isolates of the MCR-1 library growing on the plates containing 2 x MIC CAZ or AMP were selected, and the *mcr-1* genotypes of the selected isolates were identified by Sanger sequencing (Table S3). Subsequently, we conducted MIC assays to evaluate the susceptibility of the selected strains upon CAZ, AMP, and FOX. Several mutants displayed low-level resistance to AMP or CAZ (Table S3). We identified a variant named M6, carrying two mutations (P188A and P195S) localized at an α-helix of the linker domain (Figure 1A and Table S3), a region of low conservation among the protein structures of the MCR family (Figure S2). Compared to MCR-1-expressing cells, this mutant displayed increased resistance towards several β-lactams while maintaining resistance towards colistin (Figure 1B and Table 1), but not to other types of antibiotics (Figure S3A). This phenotype was further confirmed by regulating M6 expression with MCR-1 native promoter (Figure S4A). In addition, MCR-1 with either P188A or P195S abolished the co-resistance phenotype (Figure S3B), indicating that simultaneous mutation in both residues was required.

The expression of M6 had a significant impact on bacterial growth compared to MCR-1 (Figure 1C). We speculated that the phenotypic co-resistance may be related with an increase in membrane permeability conferred by M6. To confirm this, we assessed whether M6 confers resistance to membrane disrupting agents like SDS/EDTA. As observed before[32], MCR-1-expressing cells displayed increased sensitivity to detergents compared with control, whereas M6 expression partially recovered host permeability (Figure 1D). We further quantified the level of membrane permeability by n-phenyl-1-napthylamine (NPN) and propidium iodide (PI) uptake assays. Increased NPN uptake was observed in the MCR-1-expressing cells, but not in cells expressing M6 (Figure 1E). Moreover, M6 expression significantly reduced the number of PI-positive cells during stationary phase compared to MCR-1-expressing cells (Figure 1F-G). Similar phenotypes were observed when M6 expression was controlled by the MCR-1 native promoter (Figure S4B-E). These observations suggested that the co-resistance phenotype mediated by M6 could be related to membrane damage. Overall, these results revealed that M6 conferred a co-resistance towards β-lactam antibiotics via an unknown mechanism enhancing membrane integrity.

**M6 expression causes a lipid A defect in the outer membrane**

We next investigated the molecular mechanism by which M6 expression caused co-resistance towards β-lactams. OM assembly defects can activate the LDT pathway, which mediates peptidoglycan (PG) layer remodelling and resistance against β-lactams[35-37]. Using scanning electron microscopy (SEM), we found that MCR-1-expressing cells displayed wrinkles. In contrast, M6-expressing cells exhibited significant morphological changes, including folded membrane stacks and holes at the bacterial poles (Figure 2A). Transmission electron microscopy (TEM) revealed that M6 expression induced noticeable interspace within the periplasm at one pole of the cell (Figure 2B), indicating cytoplasmic shrinkage. To further quantify the level of bacterial membrane perturbation, we constructed a two-fluorescence reporter system to distinguish bacterial periplasm and cytoplasm, as described[38]. In this system, super-folded green fluorescent protein (sfGFP) was fused with the signal peptide of DsbA and localized in the periplasm, while mCherry was constitutively expressed in the cytoplasm (Figure S5A). Stationary phase cultures were collected for analysis. M6-expressing bacteria exhibited a higher proportion of cells accumulating sfGFP at bacterial pole(s) (90.7%, Figure S5B) than MCR-1 containing bacteria (61.3%). The formation of sfGFP foci at bacterial pole, consistent with IM shrinkage[38], suggested that the expression of M6 contributed to more severe membrane perturbation compared to MCR-1. We also investigated the impact of M6 upon membrane potential by using a sensitive bacterial membrane voltage V_m_ sensor, which allow single-cell recording of bacterial V_m_ dynamics in live cells. The results show that M6 confers increased membrane voltage (Figure S6A-B). Since membrane permeability is a critical determinant for membrane potential, this result also confirms a disruption of membrane homeostasis caused by M6 expression.

We employed label-free quantitative proteomics to profile proteome changes in M6 -bearing *E. coli*. In total, 2287 proteins were identified, covering 55.5% of the *E. coli* BW25113 proteome (Figure S7A and Table S4). With our significant criteria (|log2-fold change (M6/MCR-1)|≥1 and *p value* < 0.05), 66 differentially expressed proteins were identified (41 upregulated and 25 downregulated). Gene Ontology (GO) analysis revealed a significant enrichment in biological events related to bacterial cell membrane and envelope space (Figure S7B and Table S5). In KEGG analysis, an augmented process of peptidoglycan (PG) layer synthesis was observed among the top enriched processes (Figure S7C and Table S6). Proteins of LDTs pathway, such as AmpH, PBP6 and LdtD, were significantly upregulated (Figure 2C). A protein‒protein interaction (PPI) analysis further revealed, in addition to AmpH and PBP6, LpoB and PBP1B might also play crucial roles in the regulation of LDTs pathway (Figure S7D). Consistent with these findings, the transcriptional levels of genes responsible for the envelope stress response and LDTs pathway, including *yiiO* (gene encoding CpxP), *yaiH* (gene encoding AmpH), *ycbB* (gene encoding LdtD) and *yaeL* (gene encoding RseP), were significantly upregulated (Figure 2D). This result indicated that M6 primarily influenced the LDTs pathway.

LdtD and its partner proteins, PBP1B and LpoB, are essential for the activity of LDTs pathway as well as PG layer remodelling[37]. Since LdtD-mediated PG layer remodeling enables the survival of Gram-negative bacteria challenged by β-lactams[39], we explored whether M6 afects the LDTs pathway. In the presence of 3.75 mM CuSO_4_, an inhibitor of LDTs[40], the M6-expressing strain exhibited increased susceptibility towards CAZ and AMP compared to the untreated group (Figure S8A). Furthermore, the deficiency of *mrcB* (gene encoding PBP1B) reduced the CAZ, AMP, and FOX MICs of M6-expressing strain (Figure 2E). However, deficiency in *ycbB* (gene encoding LdtD) or *ycfM* (gene encoding LpoB) had no effect on susceptibility of M6 towards β-lactams (Figure S8B), indicating the dominant role of PBP1B in the M6-mediated phenotypic co-resistance. Additionally, only the absence of PBP1B (i.e., Δ*mrcb*) in M6-expressing cells decreased the resistance to SDS detergent (Figure S8C). No significant difference in NPN uptake was observed between M6-expressing WT *E. coli* and *mrcB* null mutant (Figure S8D). However, the percentage of PI-positive cells in M6-bearing Δ*mrcB* mutant was significantly higher than that in the WT strain (Figure S8E-F). These results suggested that the LDTs pathway contributes to the barrier function of IM. To further verify the IM integrity of the M6-bearing strain, spheroplasts generated by removing the OM and PG layer were confirmed by microscopy (Figure S9A). Remarkably, the M6–bearing spheroplasts exhibited increased PI-positive proportion than the whole cells (Figure S9B-C), demonstrating reduced IM integrity of M6–expressing cells. Overall, these results indicated that PBP1B was essential for phenotypic co-resistance and IM barrier function in M –bearing *E. coli*.

Because defective LPS assembly activates LDTs pathway in *E. coli*[37], we hypothesized that the up-regulation in LDTs induced by M6 could be due to lipid A disruption. Therefore, we determined the expression level of PbgA, a periplasmic protein that senses lipid A levels and regulates the content of LPS in the OM[41]. The PbgA level in M6-expressing cells was higher than that in MCR-1-expressing cells (Figure 2F-G). Moreover, the overall and IM levels of LPS in M6-expressing cells was significantly lower compared to MCR-1-expressing cells (Figure 2F-G and Figure S10A). These results demonstrated that the expression of M6 led to significant reduction in cellular lipid A level, which might be responsible for the increased permeability of both OM and IM. We next generated strains overexpressing LpxC (an essential enzyme for lipid A biosynthesis), which led to an increase in the LPS expression (Figure S10B). Consistently, permeabilization caused by MCR-1 or M6 were reversed with the compensation of LpxC (Figure 2H-I). In addition, the overexpression of LpxC abolished the co-resistance phenotype conferred by M6 (Figure 2J), highlighting the connection between membrane lipid A level and co-resistance to β-lactam antibiotics in M6-bearing strain. Taken together, these results demonstrate that a lipid A defect induced by M6 expression conferred reduced sensitivity to β-lactam antibiotics.

**Uncovering an essential lipid A binding pocket**

We hypothesized that the linker region, where the M6 point mutations occurred, might play a significant role in the enzyme function of MCR-1. To test this idea, we generated a mutant of *E. coli* BW25113 MCR-1 lacking P188-P195 segment in the linker domain. As expected, ΔP188-P195 mutation abolished the colistin resistance of *mcr-1*-bearing strain (Figure S11A), resuming the susceptibility to β-lactams (Figure S11B) as well as SDS tolerance (Figure S11C). To investigate whether this region might serve as the potential lipid A loading pocket of MCR-1, we conducted molecular docking simulations with native lipid A conformations and MCR-1. A putative lipid A binding pocket with a total of 18 residues that localized at the linker domain was proposed (Figure 3A). Notably, four regions seemed to be critical for anchoring lipid A within the loading pocket (Figure 3B and Figure S12). To further validate the significance of this predicted pocket upon MCR-1, we conducted structure-guided and site-directed mutagenesis. MICs assays revealed that these mutants played different roles in the context of colistin resistance (Figure 3B). For example, a hydrogen network was observed within K211, K204, and a phosphorate group of lipid A, and the two-points mutation K211A+K204A in MCR-1 significantly increased the susceptibility towards colistin. Additionally, a large hydrophobic region consisting of L64, I65, L68, L69, I165 and I168 likely played a role in stabilizing the flexible fatty acid tails of lipid A. The MCR-1 mutant harbouring L64A+I65A+L68A+L69A mutations inactivated the function of MCR-1. We next investigated whether mutation in the predicted lipid A binding pocket might mitigate the membrane permeabilization. The MCR-1 mutations that abolished colistin resistance also restored the integrities of both OM and IM to the same as those of control (Figure 3B). Moreover, the colistin susceptibility phenotype for these mutants was highly correlated with the integrity of the IM (ρ=0.93, *P*=0.00026) or OM (ρ=0.93, *P*=0.00026) (Figure 3C). In short, these results strongly suggested that the linker region of MCR-1, where the P188A+P195S mutations occurred, serves as the lipid A binding pocket, governing phenotypic colistin resistance and bacterial membrane integrity.

To further investigate the impact of P188A+P195S mutations upon lipid A binding pocket, we conducted molecular dynamics simulations over M6 and MCR-1 to obtain probable lipid A binding conformations and evaluate their binding stabilities (Figure S13A). The binding affinities between lipid A and WT MCR-1 or M6 were evaluated by using the MM-GBSA method, and the results showed that M6 exhibited higher affinity towards lipid than the WT MCR-1 (Figure 3D). Moreover, as illustrated in Figure S13B-C, the frequencies of the interactions between lipid A and specific residues of M6 were different from those observed with MCR-1. For example, the P188A+P195S mutations increased the interaction frequency between K204 and lipid A which were not observed in WT MCR-1 (Figure S13D). To further confirm that this pocket facilitates the lipid A binding affinity, we employed synthetic peptides encompassing the pocket of MCR-1 and M6, named peptide MCR-1 (KPLRSYVNPIMPIYSV) and peptide M6 (KALRSYVNSIMPIYSV), to interact with LPS. The dissociation constant (*K*_D_) values were approximately 12.46 nM and 4.75 nM (Figure 3E-F), respectively. This result further demonstrated the presence of a potential lipid A binding pocket at the linker domain of MCR-1 and confirmed the enhanced binding affinity towards lipid A of the mutated pocket. Finally, to investigate the impact of P188A+P195S mutations upon lipid A distribution, we undertook MALDI-TOF to analyse the abundance of modified or unmodified lipid A purified from MCR-1 or M6 -bearing cells. we found that higher abundance of unmodified lipid A was detected than that of the modified lipid A in the spheroplasts expressing MCR-1, which was in line with distribution of lipid A in the whole cells of MCR-1 -bearing strains (Figure S13E). Inversely, the abundance of modified lipid A was higher than that of the unmodified lipidA in the M6 -bearing spheroplasts. Together, these results indicated that the P188A+P195S mutations enhanced the binding affinity towards lipid A of MCR-1, and potentially resulted in accumulation of modified lipid A on the bacterial IM.

**Synthetic peptides derived from the MCR-1 M6 variant achieve broad-spectrum activity**

Inspired by previous research from Thomas et al.[41], we postulated that peptides derived from the lipid A binding cavity of MCR-1 or M6 could have antimicrobial activity. To investigate this, we designed two 24AA short peptides derived from the lipid A binding pocket of WT MCR-1 and M6, termed 24AA-WT (KPLRSYVNPIMPIYSVGKLASIEY) and 24AA-2M (KALRSYVNSIMPIYSVGKLASIEY), respectively. While peptide 24AA-WT showed no antimicrobial activity, peptide 24AA-2M exhibited activity against *E. coli* ATCC 25922 and maintained its activity during the first 6 hr (Figure 4A). However, after 24 hr, no difference in bacterial viability occurred between 24AA-2M and 24AA-WT, indicating inactivation of the synthetic peptide. To enhance the stability of the indicated peptide, we generated a derivative of 24AA-2M by adding a five-tryptophan (5W) tag at the C-terminus, named 19AA-2M-tag (KALRSYVNSIMPIYSVGKLWWWWW), since such modification aimed to increase peptide stability and protect against protease activity[42-45]. Promising results were obtained with the 19AA-2M-tag, which showed potent activity against *E. coli* ATCC 25922 and maintained its activity for at least 24 hr. SEM revealed that both 19AA-2M-tag and 24AA-2M induced severe OM shrinkage in *E. coli* (Figure 4B). Moreover, by labelling the both indicated peptides with FITC fluorescent dye at the C-terminal, we found that the FITC signal was overlapped with the signal of membrane specific dye FM4-64 (Figure S14), which was in line with the previous observations of the antimicrobial peptides functioned by interacting with bacterial membrane[46]. Importantly, 19AA-2M-tag treatment exhibited higher antimicrobial activity compared to 24AA-2M-tag (Figure 4C-D), and no significant membrane lytic activity was observed upon mouse red blood cells treated with 19AA-2M-tag, indicating that it might be a promising antimicrobial peptide (Figure S15).

We tested the susceptibility of 19AA-2M-tag against three *Escherichia coli* strains and clinically relevant pathogens, including *Pseudomonas aeruginosa*, *Salmonella* Typhimurium, *Klebsiella pneumoniae* and *Acinetobacter baumannii*. The MICs of 92.5-370 μM were obtained against these strains and the IC_50_ values were similar, ranging from 27.56 to 47.19 μM (Table 2 and Figure S16). These results suggested that 19AA-2M-tag, a peptide derived from the colistin resistance gene, effectively combated antibiotic-resistant isolates. Interestingly, 19AA-2M-tag displayed synergetic activity with colistin (CT) against *E. coli* ATCC 25922 (FICI=0.312) and a carbapenem-resistant *Enterobacteriaceae* (CRE) strain collected from the clinic (FICI=0.156, Figure S17). Moreover, the *in vivo* efficacy of the 19AA-2M-tag was demonstrated in mouse models of peritonitis against the *E. coli* ATCC 25922 and CRE clinical isolate. After 16 hours of treatment, 19AA-2M-tag significantly reduced the bacterial burden in both liver and spleen compared to the untreated group (Figure 4E). Together, these results suggested that synthetic peptides derived from the M6 represent a potential strategy to combat drug-resistant bacteria both *in vivo* and *in vitro*.

**Discussion**

In this study, we identified a MCR-1 mutant harbouring two point mutations within the linker domain, causing significant disruptions in lipid A. Unlike MCR-1, the mutant protein confers phenotypic co-resistance to β-lactam antibiotics while inducing severe lipid A perturbations. This disorder eventually results in growth arrest, membrane permeabilization, and activation of LDTs pathway. Moreover, we have identified a lipid A binding pocket that is critical for colistin resistance and bacterial membrane integrity. The mutated pocket in M6 exhibited enhanced affinity towards lipid A, potentially underpinning the β-lactam co-resistance phenotype. However, the most striking outcome of our research is that antimicrobial peptides derived from MCR-1 protein itself provide a new strategy to combat drug resistance.

Of the ten *mcr* gene variants (*mcr-1* to *mcr-10*) identified to date[47, 48], MCR-1 is considered the largest lineage with the highest prevalence, followed by MCR-3[49, 50]. Although a two-step enzymatic hydrolysis has been proposed, the exact mechanism governing the interaction between MCR-1 and lipid A remains unclear[19]. Recent research on an MCR-3 protein isolated from *Aeromonas hydrophila* suggests that a linker region of 59 residues (Linker 59) plays a crucial role in forming a phosphatidylethanolamine (PE) substrate-binding cavity and governs the interaction with lipid A, which is exposed to the bacterial periplasm[51]. In contrast, this study revealed a distinct lipid A binding pocket within the cytoplasmic membrane. Several lines of evidence support our hypothesis that the lipid A binding cavity of MCR-1 is situated within the linker domain. First, our previous work demonstrated that WT MCR-1 caused OM permeabilization, resulting in fitness costs for bacteria, particularly during the stationary growth phase. The increased OM permeability was directly related to the lipid A disturbance induced by MCR-1[32]. Here, we have identified the MCR-1 variant M6, which causes unique phenotypic characteristics to bacteria, including growth retardation, up-regulation in LDTs pathway, and co-resistance to β-lactams. The phenotypic co-resistance induced by M6 was abolished by overexpressing LpxC, an enzyme responsible for the initial step of lipid A synthesis. These findings expose a link between membrane lipid A perturbation and phenotypic co-resistance induced by M6. Second, the location of the two point mutations within the linker domain instead of the catalytic domain suggests that the effects induced by these mutations are not related with changes in the catalytic process. Third, combining MD simulation with AlphaFold structural predictions, we have confirmed the presence of a lipid A binding pocket within the linker domain of MCR-1. We also introduced mutations to interfere the interplay between such pocket and lipid A. Expression of these mutated proteins demonstrated a reduction in colistin resistance and restoration of bacterial membrane integrity. Moreover, a strong correlation between the membrane permeability and colistin resistance was observed among these mutants. Based on our observations, we propose an unusual “loading-transferring” mode for this enzyme (Figure S18). This mechanism involves several key steps: during the first-half reaction, MCR-1 in apo state interacts with the PE donor in the outer leaflet of the inner membrane, accepting a PEA group in the catalytic domain. This step transforms MCR-1 into an active state and prepares it for interactions with LPS. Subsequently, LPS binds to the MCR-1 loading pocket within the linker domain, which is embedded in the lipid bilayer. Next, LPS is transferred to the catalytic domain, which is exposed at the interface between the IM and periplasm. During the second-half reaction, the PEA group from catalytic domain is added to either the 1’- or 4’-phosphate group of LPS, forming PEA-LPS, which is then released back into the inner membrane. MCR-1 reverts to its apo state, ready for the next cycle of LPS modification.

We do not yet understand how MCR-1 impairs membrane integrity. It is well established that the cytoplasmic membrane protein PbgA plays a crucial role in sensing LPS levels and maintaining membrane LPS homeostasis[52]. PbgA directly interacts with its downstream regulator, the IM proteins PlsY and LapB. These two proteins facilitate FtsH-mediated degradation upon LpxC, the enzyme responsible for the first committed step in lipid A biosynthesis[53]. Our research shown that MCR-1 expression leads to an increase in PbgA levels, indicating an aberrant distribution of LPS in *E. coli*. Consistently, a reduced level of LPS was observed in MCR-1 positive cells. We therefore concluded that MCR-1 increased bacterial permeability by reducing cellular LPS, which was enhanced by the expression of M6 and resulted in activation of LDTs pathway as well as remodelling of PG layer (Figure 5). However, the precise molecular mechanism by which MCR-1 impacts LPS transport remains unknown.

There are still several other considerations arising from our study. First, our findings indicate that severe membrane damage occurs by M6 induced activation of LDTs pathway, resulting in reduced susceptibility of *E. coli* to β-lactam antibiotics. Several studies have shown that PG remodelling mediated by the LDTs pathway is associated with increased OM permeability[37, 40, 54, 55]. However, how M6 regulates this pathway merits further investigation. Second, the amino acid sequence of the linker domain varies among the ten known MCR variants (Figure S2). Different MCR variants exhibited varying levels of colistin resistance[56, 57]. Our data revealed that the catalytic domain of the MCR proteins is relatively well conserved among the MCR families (Figure S2), which strongly suggests that differences in the linker domain potentially govern the diverse phenotypic polymyxin resistance, an idea that will require direct experimental validation. Third, it appears that MCR-1 has pleiotropic effects and under colistin selection, the acquisition of *mcr-1* guarantees a competitive advantage within bacterial communities. However, in the absence of colistin, the lipid A binding pocket of MCR-1, which disrupts membrane lipid A homeostasis, imposes a fitness cost on bacteria. Very recently, Pramod et al. represented evidence that LpxC mutations compensated for the costs associated with MCR expression. Pre-existing LpxC mutations in pathogenic *E. coli* can potentiate the evolution of antibiotic resistance by *mcr-1*-bearing plasmid acquisition[58]. Given the co-resistance phenotype conferred by the M6 mutant, this type of gain-of-function mutation could be concerning in the clinical setting. Finally, although the NPN uptake assay suggested that MCR-1 or M6 expression may lead to reduction in OM LPS and increasing OM permeability, development of novel methodology is required to distinguish the permeability of bacterial IM and OM precisely.

Antibiotic treatment has given rise to the emergence of drug-resistant bacteria. Addressing the ongoing antibiotic crisis demands the development of newer strategies against drug-resistant infections. In our study, we identified a promising antimicrobial peptide derived from the drug resistance gene itself. Antimicrobial peptides (AMPs) are short peptides that tightly bind to the LPS on the membrane surface of bacteria. Such feature aligns with the high specificity of the lipid A binding motif found in transmembrane proteins, facilitating crucial protein-substrate interactions. A recent study shown that a synthetic peptide derived from the lipid A binding motif of PbgA displayed broad-spectrum antimicrobial activity[41]. Moreover, the introduction of an LPS binding motif into the C-terminus of temporin produces a broad spectrum of antibacterial activity[59]. Our findings extend the repertoire of AMP-based novel approaches to combat drug-resistant bacteria.

**Materials and methods**

**Ethics Statement**

With the approval from Ethics Committee of Zhongshan School of Medicine on Laboratory Animal Care (reference number: SYSU-IACUC-2022-B0031), Sun Yat-sen University, all the animal experiments were conducted based on the standard of the National Institutes of Health Guide for the Care and Use of Laboratory Animals.

**Bacterial strains and growth conditions**

The *E. coli* strains used in this research were ATCC 25922 and K-12 derivatives, including BW25113 and DH5-α. BW25113 cells were utilized to assay the influence of MCR-1 or M6 expression upon bacterial drug sensitivity, viability, and membrane permeability, while DH5-α cells acted as a cloning host for plasmid construction. The ATCC 25922 strain was routinely used to evaluate the *in vitro* bacteriostasis activity of synthetic peptides and murine *in vivo* infection. In addition, *Pseudomonas aeruginosa* ATCC 27853, *Salmonella enterica* serovar Typhimurium SL1344, *Klebsiella pneumoniae* ATCC 13883 as well as *Acinetobacter baumannii* ATCC 19606 were also used to test the susceptibility towards 19AA-2M-tag. All the above strains were cultivated in Luria Bertani (LB) broth at 37°C, antibiotics and promoter inducers were supplemented as follows when necessary: 100 μg/ml for ampicillin (AMP), 30 μg/ml for chloramphenicol (CHL) and 0.2% arabinose. All the strains used in this study were listed in Table S7. For complete materials and methods, see the supplementary materials.

**Competing Interests statement**

The authors declare no conflicts of interest.

**Financial Disclosure Statement**

This work was supported by the National Natural Science Foundation of China (grant number 81830103, 82061128001 and 82325033 to G.-B T., grant number 82002173, 82272378 to S. F.), Natural Science Foundation of Guangdong Province (grant number 2017A030306012 to G.-B T, grant number 2023A1515012392 to S. F.), Scientific and Technological Planning Project of Guangzhou City (grant number SL2022A04J01941 to S. F.), and Project of High-level Health Teams of Zhuhai in 2018 (The Innovation Team for Antimicrobial Resistance and Clinical Infection to G.-B T.). The funders had no role in study design, data collection and analysis, decision to publish, or preparation of the manuscript.

**Acknowledgments**

The plasmid encoding bacterial membrane voltage sensor Vibac2 was a generous gift from Prof. Bai Fan’s lab.

**Author Contributions**

LJL, LLZ, and LW contributed equally to this study. GBT, SYF, and FB conceived the idea, designed and supervised the study. LJL, SYF, LW, DRZ, YC and LXL conducted the experiments and produced the tables and figures. YXL, WFL, WJW, CCZ, NS, and HZ searched the literature. JCL, YD and DLP contributed to data analysis and interpretation. LJL, SYF, LW, FB and GBT wrote the manuscript. All authors reviewed, revised, and approved the final report.

**References**

1.	Rojas LJ, Salim M, Cober E, Richter SS, Perez F, Salata RA, et al. Colistin Resistance in Carbapenem-Resistant Klebsiella pneumoniae: Laboratory Detection and Impact on Mortality. Clin Infect Dis. 2017;64(6):711-8. Epub 2016/12/13. doi: 10.1093/cid/ciw805. PubMed PMID: 27940944; PubMed Central PMCID: PMCPMC5850634.

2.	Biswas S, Li Y, Elbediwi M, Yue M. Emergence and Dissemination of mcr-Carrying Clinically Relevant Salmonella Typhimurium Monophasic Clone ST34. Microorganisms. 2019;7(9). Epub 2019/08/31. doi: 10.3390/microorganisms7090298. PubMed PMID: 31466338; PubMed Central PMCID: PMCPMC6780495.

3.	Wang R, van Dorp L, Shaw LP, Bradley P, Wang Q, Wang X, et al. The global distribution and spread of the mobilized colistin resistance gene mcr-1. Nat Commun. 2018;9(1):1179. Epub 2018/03/23. doi: 10.1038/s41467-018-03205-z. PubMed PMID: 29563494; PubMed Central PMCID: PMCPMC5862964.

4.	Wang Q, Sun J, Li J, Ding Y, Li XP, Lin J, et al. Expanding landscapes of the diversified mcr-1-bearing plasmid reservoirs. Microbiome. 2017;5(1):70. Epub 2017/07/08. doi: 10.1186/s40168-017-0288-0. PubMed PMID: 28683827; PubMed Central PMCID: PMCPMC5500976.

5.	Zhao Q, Li Y, Tian Y, Shen Y, Wang S, Zhang Y. Clinical Impact of Colistin Banning in Food Animal on mcr-1-Positive Enterobacteriaceae in Patients From Beijing, China, 2009-2019: A Long-Term Longitudinal Observational Study. Front Microbiol. 2022;13:826624. Epub 2022/03/01. doi: 10.3389/fmicb.2022.826624. PubMed PMID: 35222338; PubMed Central PMCID: PMCPMC8866948.

6.	Wang Y, Xu C, Zhang R, Chen Y, Shen Y, Hu F, et al. Changes in colistin resistance and mcr-1 abundance in Escherichia coli of animal and human origins following the ban of colistin-positive additives in China: an epidemiological comparative study. Lancet Infect Dis. 2020;20(10):1161-71. Epub 2020/06/09. doi: 10.1016/S1473-3099(20)30149-3. PubMed PMID: 32505232.

7.	Liu YY, Zhou Q, He W, Lin Q, Yang J, Liu JH. mcr-1 and plasmid prevalence in Escherichia coli from livestock. Lancet Infect Dis. 2020;20(10):1126. Epub 2020/09/27. doi: 10.1016/S1473-3099(20)30697-6. PubMed PMID: 32979325.

8.	Shen C, Zhong LL, Zhong Z, Doi Y, Shen J, Wang Y, et al. Prevalence of mcr-1 in Colonized Inpatients, China, 2011-2019. Emerg Infect Dis. 2021;27(9):2502-4. Epub 2021/08/24. doi: 10.3201/eid2709.203642. PubMed PMID: 34424179; PubMed Central PMCID: PMCPMC8386786.

9.	Shen C, Zhong LL, Yang Y, Doi Y, Paterson DL, Stoesser N, et al. Dynamics of mcr-1 prevalence and mcr-1-positive Escherichia coli after the cessation of colistin use as a feed additive for animals in China: a prospective cross-sectional and whole genome sequencing-based molecular epidemiological study. Lancet Microbe. 2020;1(1):e34-e43. Epub 2020/05/01. doi: 10.1016/S2666-5247(20)30005-7. PubMed PMID: 35538907.

10.	Huang H, Dong N, Shu L, Lu J, Sun Q, Chan EW, et al. Colistin-resistance gene mcr in clinical carbapenem-resistant Enterobacteriaceae strains in China, 2014-2019. Emerg Microbes Infect. 2020;9(1):237-45. Epub 2020/01/31. doi: 10.1080/22221751.2020.1717380. PubMed PMID: 31996107; PubMed Central PMCID: PMCPMC7034111.

11.	Ma G, Zhu Y, Yu Z, Ahmad A, Zhang H. High resolution crystal structure of the catalytic domain of MCR-1. Sci Rep. 2016;6:39540. Epub 2016/12/22. doi: 10.1038/srep39540. PubMed PMID: 28000749; PubMed Central PMCID: PMCPMC5175174.

12.	Sabnis A, Hagart KL, Klockner A, Becce M, Evans LE, Furniss RCD, et al. Colistin kills bacteria by targeting lipopolysaccharide in the cytoplasmic membrane. Elife. 2021;10. Epub 20210406. doi: 10.7554/eLife.65836. PubMed PMID: 33821795; PubMed Central PMCID: PMCPMC8096433.

13.	Xu Y, Lin J, Cui T, Srinivas S, Feng Y. Mechanistic insights into transferable polymyxin resistance among gut bacteria. J Biol Chem. 2018;293(12):4350-65. Epub 2018/02/21. doi: 10.1074/jbc.RA117.000924. PubMed PMID: 29462787; PubMed Central PMCID: PMCPMC5868246.

14.	Gao R, Hu Y, Li Z, Sun J, Wang Q, Lin J, et al. Dissemination and Mechanism for the MCR-1 Colistin Resistance. PLoS Pathog. 2016;12(11):e1005957. Epub 2016/11/29. doi: 10.1371/journal.ppat.1005957. PubMed PMID: 27893854; PubMed Central PMCID: PMCPMC5125707.

15.	Zhang XF, Doi Y, Huang X, Li HY, Zhong LL, Zeng KJ, et al. Possible Transmission of mcr-1-Harboring Escherichia coli between Companion Animals and Human. Emerg Infect Dis. 2016;22(9):1679-81. doi: 10.3201/eid2209.160464. PubMed PMID: WOS:000381955900036.

16.	Sun J, Zhang H, Liu YH, Feng Y. Towards understanding Mcr-like colistin resistance. Trends Microbiol. 2018;26(9):794-808. Epub 20180307. doi: 10.1016/j.tim.2018.02.006. PubMed PMID: 29525421.

17.	Bogdanov M, Pyrshev K, Yesylevskyy S, Ryabichko S, Boiko V, Ivanchenko P, et al. Phospholipid distribution in the cytoplasmic membrane of Gram-negative bacteria is highly asymmetric, dynamic, and cell shape-dependent. Sci Adv. 2020;6(23):eaaz6333. Epub 2020/06/17. doi: 10.1126/sciadv.aaz6333. PubMed PMID: 32537497; PubMed Central PMCID: PMCPMC7269648.

18.	Doerrler WT, Gibbons HS, Raetz CR. MsbA-dependent translocation of lipids across the inner membrane of *Escherichia coli*. J Biol Chem. 2004;279(43):45102-9. doi: 10.1074/jbc.M408106200. PubMed PMID: 15304478.

19.	Zhang H, Srinivas S, Xu Y, Wei W, Feng Y. Genetic and Biochemical Mechanisms for Bacterial Lipid A Modifiers Associated with Polymyxin Resistance. Trends Biochem Sci. 2019;44(11):973-88. Epub 2019/07/08. doi: 10.1016/j.tibs.2019.06.002. PubMed PMID: 31279652.

20.	Xu Y, Wei W, Lei S, Lin J, Srinivas S, Feng Y. An Evolutionarily Conserved Mechanism for Intrinsic and Transferable Polymyxin Resistance. mBio. 2018;9(2). Epub 2018/04/11. doi: 10.1128/mBio.02317-17. PubMed PMID: 29636432; PubMed Central PMCID: PMCPMC5893884.

21.	Powers MJ, Trent MS. Phospholipid retention in the absence of asymmetry strengthens the outer membrane permeability barrier to last-resort antibiotics. Proc Natl Acad Sci U S A. 2018;115(36):E8518-E27. Epub 2018/08/09. doi: 10.1073/pnas.1806714115. PubMed PMID: 30087182; PubMed Central PMCID: PMCPMC6130378.

22.	Wang X, Quinn PJ, Yan A. Kdo2 -lipid A: structural diversity and impact on immunopharmacology. Biol Rev Camb Philos Soc. 2015;90(2):408-27. Epub 2014/05/20. doi: 10.1111/brv.12114. PubMed PMID: 24838025; PubMed Central PMCID: PMCPMC4402001.

23.	Raetz CR, Reynolds CM, Trent MS, Bishop RE. Lipid A modification systems in gram-negative bacteria. Annu Rev Biochem. 2007;76:295-329. Epub 2007/03/17. doi: 10.1146/annurev.biochem.76.010307.145803. PubMed PMID: 17362200; PubMed Central PMCID: PMCPMC2569861.

24.	Yeow J, Chng SS. Of zones, bridges and chaperones - phospholipid transport in bacterial outer membrane assembly and homeostasis. Microbiology (Reading). 2022;168(4). Epub 2022/04/07. doi: 10.1099/mic.0.001177. PubMed PMID: 35384832.

25.	Sutterlin HA, Shi H, May KL, Miguel A, Khare S, Huang KC, Silhavy TJ. Disruption of lipid homeostasis in the Gram-negative cell envelope activates a novel cell death pathway. Proc Natl Acad Sci U S A. 2016;113(11):E1565-74. Epub 2016/03/02. doi: 10.1073/pnas.1601375113. PubMed PMID: 26929379; PubMed Central PMCID: PMCPMC4801249.

26.	Mandela E, Stubenrauch CJ, Ryoo D, Hwang H, Cohen EJ, Torres VL, et al. Adaptation of the periplasm to maintain spatial constraints essential for cell envelope processes and cell viability. Elife. 2022;11. Epub 2022/01/28. doi: 10.7554/eLife.73516. PubMed PMID: 35084330; PubMed Central PMCID: PMCPMC8824477.

27.	Yi L, Durand R, Grenier F, Yang J, Yu K, Burrus V, Liu JH. PixR, a Novel Activator of Conjugative Transfer of IncX4 Resistance Plasmids, Mitigates the Fitness Cost of mcr-1 Carriage in Escherichia coli. mBio. 2022:e0320921. Epub 2022/01/29. doi: 10.1128/mbio.03209-21. PubMed PMID: 35089067; PubMed Central PMCID: PMCPMC8725589.

28.	Yang J, Wang HH, Lu Y, Yi LX, Deng Y, Lv L, et al. A ProQ/FinO family protein involved in plasmid copy number control favours fitness of bacteria carrying mcr-1-bearing IncI2 plasmids. Nucleic Acids Res. 2021;49(7):3981-96. Epub 2021/03/16. doi: 10.1093/nar/gkab149. PubMed PMID: 33721023; PubMed Central PMCID: PMCPMC8053102.

29.	Wu R, Yi LX, Yu LF, Wang J, Liu Y, Chen X, et al. Fitness Advantage of mcr-1-Bearing IncI2 and IncX4 Plasmids in Vitro. Front Microbiol. 2018;9:331. Epub 2018/03/15. doi: 10.3389/fmicb.2018.00331. PubMed PMID: 29535696; PubMed Central PMCID: PMCPMC5835064.

30.	Yang Q, Li M, Spiller OB, Andrey DO, Hinchliffe P, Li H, et al. Balancing mcr-1 expression and bacterial survival is a delicate equilibrium between essential cellular defence mechanisms. Nat Commun. 2017;8(1):2054. Epub 2017/12/14. doi: 10.1038/s41467-017-02149-0. PubMed PMID: 29233990; PubMed Central PMCID: PMCPMC5727292.

31.	Ma K, Feng Y, Zong Z. Fitness cost of a mcr-1-carrying IncHI2 plasmid. PLoS One. 2018;13(12):e0209706. Epub 2018/12/27. doi: 10.1371/journal.pone.0209706. PubMed PMID: 30586457; PubMed Central PMCID: PMCPMC6306219.

32.	Feng S, Liang W, Li J, Chen Y, Zhou D, Liang L, et al. MCR-1-dependent lipid remodelling compromises the viability of Gram-negative bacteria. Emerg Microbes Infect. 2022;11(1):1236-49. Epub 2022/04/20. doi: 10.1080/22221751.2022.2065934. PubMed PMID: 35437117; PubMed Central PMCID: PMCPMC9067951.

33.	Li B, Yin F, Zhao X, Guo Y, Wang W, Wang P, et al. Colistin Resistance Gene mcr-1 Mediates Cell Permeability and Resistance to Hydrophobic Antibiotics. Front Microbiol. 2019;10:3015. Epub 2020/01/31. doi: 10.3389/fmicb.2019.03015. PubMed PMID: 31998280; PubMed Central PMCID: PMCPMC6966882.

34.	Guo Z, Feng S, Liang L, Li J, Zhong L-L, Zhao H, et al. The fitness landscape of the mobilized colistin resistance gene <em>mcr-1</em>. bioRxiv. 2022:2022.07.21.500813. doi: 10.1101/2022.07.21.500813.

35.	Apostolos AJ, Pidgeon SE, Pires MM. Remodeling of Cross-bridges Controls Peptidoglycan Cross-linking Levels in Bacterial Cell Walls. ACS Chem Biol. 2020;15(5):1261-7. Epub 2020/03/14. doi: 10.1021/acschembio.0c00002. PubMed PMID: 32167281; PubMed Central PMCID: PMCPMC7887760.

36.	Cavallari JF, Lamers RP, Scheurwater EM, Matos AL, Burrows LL. Changes to its peptidoglycan-remodeling enzyme repertoire modulate beta-lactam resistance in Pseudomonas aeruginosa. Antimicrob Agents Chemother. 2013;57(7):3078-84. Epub 2013/04/25. doi: 10.1128/AAC.00268-13. PubMed PMID: 23612194; PubMed Central PMCID: PMCPMC3697359.

37.	More N, Martorana AM, Biboy J, Otten C, Winkle M, Serrano CKG, et al. Peptidoglycan Remodeling Enables Escherichia coli To Survive Severe Outer Membrane Assembly Defect. mBio. 2019;10(1). Epub 2019/02/07. doi: 10.1128/mBio.02729-18. PubMed PMID: 30723128; PubMed Central PMCID: PMCPMC6428754.

38.	Shi H, Westfall CS, Kao J, Odermatt PD, Anderson SE, Cesar S, et al. Starvation induces shrinkage of the bacterial cytoplasm. Proc Natl Acad Sci U S A. 2021;118(24). Epub 2021/06/13. doi: 10.1073/pnas.2104686118. PubMed PMID: 34117124; PubMed Central PMCID: PMCPMC8214708.

39.	Hugonnet JE, Mengin-Lecreulx D, Monton A, den Blaauwen T, Carbonnelle E, Veckerle C, et al. Factors essential for L,D-transpeptidase-mediated peptidoglycan cross-linking and beta-lactam resistance in Escherichia coli. Elife. 2016;5. Epub 2016/11/02. doi: 10.7554/eLife.19469. PubMed PMID: 27767957; PubMed Central PMCID: PMCPMC5089857.

40.	Peters K, Pazos M, Edoo Z, Hugonnet JE, Martorana AM, Polissi A, et al. Copper inhibits peptidoglycan LD-transpeptidases suppressing beta-lactam resistance due to bypass of penicillin-binding proteins. Proc Natl Acad Sci U S A. 2018;115(42):10786-91. Epub 2018/10/03. doi: 10.1073/pnas.1809285115. PubMed PMID: 30275297; PubMed Central PMCID: PMCPMC6196517.

41.	Clairfeuille T, Buchholz KR, Li Q, Verschueren E, Liu P, Sangaraju D, et al. Structure of the essential inner membrane lipopolysaccharide-PbgA complex. Nature. 2020;584(7821):479-83. Epub 2020/08/14. doi: 10.1038/s41586-020-2597-x. PubMed PMID: 32788728.

42.	Malmsten M, Kasetty G, Pasupuleti M, Alenfall J, Schmidtchen A. Highly selective end-tagged antimicrobial peptides derived from PRELP. PLoS One. 2011;6(1):e16400. Epub 2011/02/08. doi: 10.1371/journal.pone.0016400. PubMed PMID: 21298015; PubMed Central PMCID: PMCPMC3029338.

43.	Schmidtchen A, Pasupuleti M, Morgelin M, Davoudi M, Alenfall J, Chalupka A, Malmsten M. Boosting antimicrobial peptides by hydrophobic oligopeptide end tags. J Biol Chem. 2009;284(26):17584-94. Epub 2009/04/29. doi: 10.1074/jbc.M109.011650. PubMed PMID: 19398550; PubMed Central PMCID: PMCPMC2719397.

44.	Pasupuleti M, Chalupka A, Morgelin M, Schmidtchen A, Malmsten M. Tryptophan end-tagging of antimicrobial peptides for increased potency against Pseudomonas aeruginosa. Biochim Biophys Acta. 2009;1790(8):800-8. Epub 2009/04/07. doi: 10.1016/j.bbagen.2009.03.029. PubMed PMID: 19345721.

45.	Stromstedt AA, Pasupuleti M, Schmidtchen A, Malmsten M. Oligotryptophan-tagged antimicrobial peptides and the role of the cationic sequence. Biochim Biophys Acta. 2009;1788(9):1916-23. Epub 2009/06/10. doi: 10.1016/j.bbamem.2009.06.001. PubMed PMID: 19505433.

46.	Lee H, Lim SI, Shin SH, Lim Y, Koh JW, Yang S. Conjugation of Cell-Penetrating Peptides to Antimicrobial Peptides Enhances Antibacterial Activity. ACS Omega. 2019;4(13):15694-701. Epub 2019/10/02. doi: 10.1021/acsomega.9b02278. PubMed PMID: 31572872; PubMed Central PMCID: PMCPMC6761801.

47.	Wang C, Feng Y, Liu L, Wei L, Kang M, Zong Z. Identification of novel mobile colistin resistance gene mcr-10. Emerg Microbes Infect. 2020;9(1):508-16. Epub 2020/03/03. doi: 10.1080/22221751.2020.1732231. PubMed PMID: 32116151; PubMed Central PMCID: PMCPMC7067168.

48.	Hussein NH, Al-Kadmy IMS, Taha BM, Hussein JD. Mobilized colistin resistance (mcr) genes from 1 to 10: a comprehensive review. Mol Biol Rep. 2021;48(3):2897-907. Epub 2021/04/12. doi: 10.1007/s11033-021-06307-y. PubMed PMID: 33839987.

49.	Smelikova E, Tkadlec J, Krutova M. How to: screening for mcr-mediated resistance to colistin. Clin Microbiol Infect. 2022;28(1):43-50. Epub 2021/09/20. doi: 10.1016/j.cmi.2021.09.009. PubMed PMID: 34537365.

50.	Talebi G, Hashemi A. Metallo-β-lactamase, extended spectrum β-lactamase and mcr-1 gene as major therapeutic challenges. Reviews and Research in Medical Microbiology. 2022;33(1).

51.	Xu Y, Chen H, Zhang H, Ullah S, Hou T, Feng Y. The MCR-3 inside linker appears as a facilitator of colistin resistance. Cell Rep. 2021;35(7):109135. Epub 2021/05/20. doi: 10.1016/j.celrep.2021.109135. PubMed PMID: 34010644.

52.	Simpson BW, Douglass MV, Trent MS. Restoring Balance to the Outer Membrane: YejM's Role in LPS Regulation. mBio. 2020;11(6). Epub 2020/12/17. doi: 10.1128/mBio.02624-20. PubMed PMID: 33323515; PubMed Central PMCID: PMCPMC7773988.

53.	Guest RL, Same Guerra D, Wissler M, Grimm J, Silhavy TJ. YejM modulates activity of the YciM/FtsH protease complex to prevent lethal accumulation of lipopolysaccharide. mBio. 2020;11(2). Epub 20200414. doi: 10.1128/mBio.00598-20. PubMed PMID: 32291302; PubMed Central PMCID: PMCPMC7157816.

54.	Sandoz KM, Moore RA, Beare PA, Patel AV, Smith RE, Bern M, et al. beta-Barrel proteins tether the outer membrane in many Gram-negative bacteria. Nat Microbiol. 2021;6(1):19-26. Epub 2020/11/04. doi: 10.1038/s41564-020-00798-4. PubMed PMID: 33139883; PubMed Central PMCID: PMCPMC7755725.

55.	Simpson BW, Nieckarz M, Pinedo V, McLean AB, Cava F, Trent MS. Acinetobacter baumannii Can Survive with an Outer Membrane Lacking Lipooligosaccharide Due to Structural Support from Elongasome Peptidoglycan Synthesis. mBio. 2021;12(6):e0309921. Epub 2021/12/01. doi: 10.1128/mBio.03099-21. PubMed PMID: 34844428; PubMed Central PMCID: PMCPMC8630537.

56.	Liao W, Cui Y, Quan J, Zhao D, Han X, Shi Q, et al. High prevalence of colistin resistance and mcr-9/10 genes in Enterobacter spp. in a tertiary hospital over a decade. Int J Antimicrob Agents. 2022;59(5):106573. Epub 2022/03/22. doi: 10.1016/j.ijantimicag.2022.106573. PubMed PMID: 35307563.

57.	Chatzidimitriou M, Kavvada A, Kavvadas D, Kyriazidi MA, Meletis G, Chatzopoulou F, Chatzidimitriou D. mcr Genes Conferring Colistin Resistance in Enterobacterales; a Five Year Overview. Acta Med Acad. 2021;50(3):365-71. Epub 2022/02/16. doi: 10.5644/ama2006-124.355. PubMed PMID: 35164512.

58.	Thanert R, Choi J, Reske KA, Hink T, Thanert A, Wallace MA, et al. Persisting uropathogenic Escherichia coli lineages show signatures of niche-specific within-host adaptation mediated by mobile genetic elements. Cell Host Microbe. 2022. Epub 2022/05/12. doi: 10.1016/j.chom.2022.04.008. PubMed PMID: 35545083.

59.	Mohanram H, Bhattacharjya S. 'Lollipop'-shaped helical structure of a hybrid antimicrobial peptide of temporin B-lipopolysaccharide binding motif and mapping cationic residues in antibacterial activity. Biochim Biophys Acta. 2016;1860(6):1362-72. Epub 2016/03/27. doi: 10.1016/j.bbagen.2016.03.025. PubMed PMID: 27015761.

**Figures**

**Figure 1. An MCR-1 mutant M6 (harbouring P188A+P195S) exhibited reduced sensitivity towards β-lactam antibiotics.**

**(A)** MCR-1 protein structure in cartoon. The linker domain (blue) and approximate membrane boundaries are indicated. TMD, transmembrane domain. Close-up view of two mutated residues. The mutated residues P188 and P195 are highlighted in orange.

**(B)** The logarithmic phase cultures of *E. coli* BW25113 carrying empty vector, M6, or MCR-1 were collected to evaluate the sensitivity towards CT and β-lactam antibiotics (AMP, FOX, IMP, CAZ, CTX and MEM) by agar dilution MICs tests. Each triangle represents an independent experiment. And the experiments were performed three times with same results.

**(C)** Growth curves of *E. coli* BW25113 carrying empty vector, M6, or MCR-1. The y-axes show optical densities at 600 nm (OD_600_) of broth cultures, x-axes show period of growth (hr).

**(D)** Efficiency of plating assays on LB agar plates containing 1% SDS and 1 mM EDTA or 0.001% SDS and 1 mM EDTA. Ten-fold serial-dilution of indicated cultures were inoculated onto the agar plates.

**(E)** The OM integrity of strains carrying M6 or MCR-1 was determined by measuring NPN uptake during logarithmic phase. And the fluorescent signal for each sample was immediately monitored with a microplate reader at an excitation wavelength of 350 nm and emission wavelength of 420 nm after staining.

**(F-G)** The IM permeability of cells carrying MCR-1 or M6 was evaluated by PI staining assay. Overnight cultures were sub-cultured into fresh LB broth at a ratio of 1:100 and induced with 0.2% arabinose to express MCR-1 or M6. After induction for 4 hr and 8 hr, stationary and late-stationary phase cultures were collected, respectively, followed by staining with PI dye for 15 min. The PI-positive proportion was determined by flow cytometry and analysed by FlowJo version 10 software.

All the above-described experiments were performed thrice with similar results. Error bars indicate standard errors of the means (SEMs) for three biological replicates. A two-tailed unpaired *t* test was performed to determine the statistical significance of the data. ns, no significant difference; *, *P*< 0.1; **, *P*< 0.01; ***, *P*< 0.001.

**Figure 2. M6 activated LDTs pathway via the induction of severe lipid disorders.**

**(A)** SEM micrographs of *E. coli* BW25113 carrying MCR-1 or M6. Overnight cultures of the above strains were sub-cultured into fresh LB broth at a ratio of 1:100 and induced with 0.2% arabinose for 2 hr before sample preparation.

**(B)** TEM micrographs of *E. coli* BW25113 carrying MCR-1 or M6. Overnight cultures of the above strains were sub-cultured into fresh LB broth at a ratio of 1:100 and induced with 0.2% arabinose for 2 hr before sample preparation.

**(C)** The volcano diagram of the differentially expressed protein between MCR-1 -bearing cells and M6 -bearing cells determined by label-free quantitative proteomics. The differential expression threshold was set as log_2_ fold change >1 and *p* value <0.05. Red dots represent significantly upregulated genes, while blue dots represent significantly downregulated genes. The LDTs-pathway related proteins are highlighted in green.

**(D)** The transcriptional level of differentially expressed genes was measured by qRT‒PCR, which was normalized to the transcript level of the housekeeping gene *rpoB* and quantified with ΔΔCT analysis. The heatmap represents the fold change in target gene transcriptional levels for MCR-1-expressing cells or M6-expressing cells compared to those of the empty plasmid control.

**(E)** Evaluation of the impact of *mrcB* on the M6-mediated co-resistance phenotype. The antibiotic susceptibility of WT or Δ*mrcB* carrying empty plasmid (control), MCR-1 or MCR-6 was determined by agar dilution MIC tests. Each triangle represents an independent experiment. And the experiments were performed three times with same results.

**(F-G)** The logarithmic phase cultures of *E. coli* BW25113 carrying empty vector, M6, or MCR-1 were collected. And the cellular levels of PbgA and LPS of indicated strains were determined by western blot after cell lysis with ultrasonication.

**(H)** Evaluation of the impact of LpxC overexpression on M6-mediated OM permeability. The OM integrity was determined by measuring NPN uptake during logarithmic phase.

**(I)** Evaluation of the impact of LpxC overexpression on M6-mediated IM permeability. The IM integrity was evaluated by PI staining assay.

**(J)** Assessment of the impact of LpxC on the M6-mediated co-resistance phenotype. The FOX MICs of WT or LpxC overexpression strains carrying empty plasmid (control), MCR-1 or M6 were determined by agar dilution MICs tests.

All the above-described experiments were performed three times with similar results. Error bars indicate standard errors of the means (SEMs) for three biological replicates. A two-tailed unpaired *t* test was performed to determine the statistical significance of the data. ns, no significant difference; **, *P*< 0.01; ***, *P*< 0.001.

**Figure 3. Discovery and functional definition of the lipid A loading pocket in MCR-1.**

**(A)** Protein structure of MCR-1 and close-up view of the LPS binding pockrt at the linker domain. The catalytic domain, linker domain and transmembrane domain are in cyan, magenta and orange, respectively, with LPS is shown as green sticks and salt bridges for interaction as red dashes.

**(B)** Verification of the key residues in the lipid A binding cavity that interacts with lipid A. To evaluate the influence of mutations upon colistin resistance activity, the colistin MICs of the indicated mutants were determined by agar dilution MICs tests. In addition, logarithmic phase cultures of target mutants were applied to evaluate the permeability of the IM and OM by NPN uptake assay and PI staining assay, respectively.

**(C)** Evaluating the correlation between membrane integrity and colistin resistance. ρ is Spearman’s rank correlation coefficient. The related *P* value and regression line (blue) are shown.

**(D)** Estimate the free energy of binding of lipid A to WT MCR-1 and M6. The results show that the M6 mutant has a better binding free energy compared to WT. Where the binding free energy was calculated using the MM-GBSA for the last 200 ns trajectory after the protein-ligand complex reached equilibrium.

**(E-F)** The affinity between lipid A substrate and linker domain of MCR-1/M6 was determined by biolayer interferometry. The synthetic peptides derived from the linker domain of WT MCR-1 or M6 (named peptide MCR-1 and peptide M6, respectively) were coated on the surface of streptavidin (SA) biosensor biosensors. Target peptides were bound to Kdo_2_-lipid A in series of concentrations (150, 100, 50, 25 and 10 μM) with 300 s association and dissociation steps. Assays were performed in triplicate on an Octet Red384 (Sartorius).

All the above-described experiments were performed three times with similar results. Error bars indicate standard errors of the means (SEMs) for three biological replicates. A two-tailed unpaired *t* test was performed to determine the statistical significance of the data. ns, no significant difference; *, *P*< 0.1; **, *P*< 0.01; ***, *P*< 0.001; ****, *P*< 0.0001.

**Figure 4. Synthetic peptides derived from M6 displayed antimicrobial activity *in vitro* and *in vivo*.**

**(A)** Time-dependent killing of *E. coli* ATCC 25922 by 24AA-WT, 24AA-2M, or 19AA-2M-tag. An exponential culture of *E. coli* ATCC25922 was challenged with the indicated peptides. Cultures treated with DMSO or 5W were set as control.

**(B)** Scanning electron microscopy (SEM) analysis of *E. coli* ATCC 25922 treated with 24AA-2M or 19AA-2M-tag in the concentration of 0.25× MICs.

**(C-D)** Evaluation of the bacterial membrane permeability with the treatment of 24AA-2M or 19AA-2M-tag *in vitro* by PI staining assay.

**(E)** Evaluation of the bactericidal ability of 19AA-2M-tag *in vivo*. C57BL/6 mice were first infected with 1×10^7^ CFUs of *E. coli* ATCC 25922 or CRE isolated in the clinic through intraperitoneal injection for 1 hr, followed by treatment with 200 μg of the indicated peptides. The infected individuals treated with DMSO were used as controls. Liver or spleen bacterial burdens were determined 16 hours after treatment by spotting serial dilutions of tissue homogenates on LB plates. The average CFUs values of each organ with (blue) or without (grey) 19AA-2M-tag treatment were counted 16 hr after incubation at 37 °C.

All the above-described experiments were performed thrice with similar results. Error bars indicate standard errors of the means (SEMs) for three biological replicates. A two-tailed unpaired *t* test was performed to determine the statistical significance of the data. *, *P*< 0.1; **, *P*< 0.01; ***, *P*< 0.001.

**Figure 5. Mode of membrane perturbation induced by MCR-1 or M6.**

The lipid A PEA modification mediated by MCR-1 involves in following main steps: (i) lipid A interacts with the binding pocket within linker region, followed by (ii) transferring lipid A to the catalytic domain to accept PEA group. Finally, (iii) lipid A-PEA is released from MCR-1. (**A**) The expression of wild-type MCR-1 is able to modify LPS localized at the outer leaflet of inner membrane. Subsequently, lipid A-PEA is transported to outer membrane through Lpt bridge, a trans-envelope protein family for lipid A transportation. However, the expression of M6 induced perturbation upon membrane lipid A. **(B)** The enhanced lipid A substrate loading process of M6 (highlighted in green) appears to be responsible for the reduced lipid A on both IM and OM and, to some extent, reduces the bacterial sensitivity towards β-lactam antibiotics.

**Table 1. β-Lactam antibiotics MICs of M6**

| **Strains** | **MICs (µg/ml)** | | | | | |
| --- | --- | --- | --- | --- | --- | --- |
|  | **CAZ** | **AMP** | **PIP** | **CTX** | **FOX** | **CT** |
| M6 expressing BW25113 | ≥0.6 | 16 | 4 | 0.2 | ≥10 | ≥16 |
| MCR-1 expressing BW25113 | 0.2 | 8 | 4 | 0.05 | 4 | ≥16 |
| BW25113 carrying empty vector | 0.2 | 8 | 4 | 0.05 | 4 | 0.5 |

**Table 2. 19AA-2M-tag exhibits broad-spectrum antibacterial activity**

| **Strains** | **MICs (μM)** |
| --- | --- |
| *Escherichia coli* BW25113 | 185 |
| *Escherichia coli* MG1655 | 370 |
| *Escherichia coli* ATCC 25922 | 370 |
| *Pseudomonas aeruginosa* ATCC 27853 | 185 |
| *Salmonella enterica serovar Typhimurium* SL1344 | 185 |
| *Klebsiella pneumoniae* ATCC 13883 | 185 |
| *Acinetobacter baumannii* ATCC 19606 | 185 |
| *A. baumannii* clinical isolate 1 | 185 |
| *A. baumannii* clinical isolate 2 | 185 |
| *E. coli* CRE clinical isolate 1 | 92.5 |
| *K. pneumoniae* CRKP clinical isolate 1 | 185 |
| *E. coli* *mcr-1*^+^ clinical isolate 1 | 370 |
| *E. coli* *mcr-1*^+^ clinical isolate 2 | 370 |
