# Supplementary material for: A new variant of the colistin resistance gene MCR-1 with co-resistance to β-lactam antibiotics reveals a potential novel antimicrobial peptide
Source: PLoS Biol. 2023 Dec 13;21(12):e3002433. doi: 10.1371/journal.pbio.3002433 (PMC10786390; doi:10.1371/journal.pbio.3002433)
Supplement: Attachment — Submitted filename: Response letter 20231110.docx [file pbio.3002433.s037.docx]

**Responding letter**

**Editor’s Comments**
Based on the review and our Academic Editor's assessment of your revision, we are likely to accept this manuscript for publication, provided you satisfactorily address the remaining points raised by the reviewer and the following data and other policy-related requests.

Reply: Thank you for your kind effort handling our manuscript. And it’s our great honor to publish this manuscript in PLOS BIOLOGY. Please see our point-to-point responses to the comments from editor and reviewer below.

Particularly, we have changed the title of the manuscript into “A new variant of the colistin resistance gene MCR-1 with co-resistance to β-lactam antibiotics reveals a potential novel antimicrobial peptide”. And we also provide a blurb about our research. Additionally, we have shared the custom code available as open source. Finally, based on the suggestion of reviewer #2, we have improved the readability and precision of the manuscript. The sentences and paragraphs added to make corrections in the revised manuscript are highlighted in yellow.

IMPORTANT - please attend to the following:

a) For our broad readership, please change your Title to "A new variant of the colistin resistance gene MCR-1 with low level of co-resistance to β-lactam antibiotics reveals a potential novel antimicrobial peptide"

Reply: We sincerely thank the suggestion from editor. We have renamed the manuscript as “A new variant of the colistin resistance gene MCR-1 with co-resistance to β-lactam antibiotics reveals a potential novel antimicrobial peptide”.

b) Please provide a blurb, according to the instructions in the submission form.

Reply: We sincerely thank the suggestion from editor, and the blurb of our research is as followed: “A *mcr-1* variant exhibits co-resistance towards β-lactam antibiotics that uncovers a potential lipid A binding cavity. Moreover, a novel antimicrobial peptide derived from this mutant itself displays broad-spectrum antimicrobial activity.”

c) Please address the remaining comments from reviewer #2. Note that this reviewer has attached an annotated version of your manuscript, which you may find helpful.

Reply: We sincerely thank the comments from editor and reviewer. And we have corrected the related problem and mistake based on the suggestions of reviewer #2.

d) Please rename your data file "Raw data 20230918.xlsx" to "S1_data.xlsx" and cite the location of the data clearly in all relevant main and supplementary Figure legends, e.g. “The data underlying this Figure can be found in S1 Data.”

Reply: We sincerely thank the suggestion from editor. We have already renamed the related file and cited the location of the raw data for each figure in the corresponding legends.

e) Please make any custom code available, either as a supplementary file or as part of a deposition.

Reply: We sincerely thank the suggestion from editor. We have shared our custom code as online open source and the related demonstration has been added into the manuscript as the section of “Code availability”: Line 438-440, “The custom code used in this research for proteomics analysis, MCR proteins homology analysis and homology protein modelling were deposited in https://github.com/LiangLujie/MCR-1_new_variant and https://github.com/Wang-Lin-boop/AutoMD.”

**Reviewer #2’s Comments:**
IMPORTANT: ALSO SEE THE ATTACHED EDITED VERSION OF THE MANUSCRIPT

The revised manuscript of Liang et al. is considerably improved. The authors have responded extensively to the referees' comments and provided new experimental information. However, the manuscript continues to be poorly written. It is not only about English usage but mainly about style. On this respect, the style is verbose, and many sentences begin with "Notably", "remarkably", "unexpectedly" which make reading fastidious and are not necessary.

Reply: We sincerely thank the suggestion from reviewer, and we have removed the related words from the manuscript. Also, to increase the readability of the manuscript, we have polished the writing of the manuscript based on the suggestions of reviewer.

Further there are several sentences that need major editing for clarity. For example, "two-points mutation" means nothing. The authors are referring to "two point mutations".

Reply: We sincerely thank the suggestion from reviewer. We have corrected the related words in the manuscript, which are as followed:

Line 52-54: “Here, we identified the MCR-1 M6 variant carrying two point mutations that conferred co-resistance to β-lactam antibiotics.”

Line 119: “…where the two point mutations in M6, potentially increasing the lipid A binding affinity.”

Line 319: “…we identified an MCR-1 mutant harbouring two point mutations within the linker domain.”

Line 345: “Second, the location of the two point mutations within the linker domain…”

In other cases, additional explanations are given when the references are provided, which could be safely deleted.

Reply: We sincerely thank the suggestion from reviewer. Several sentences have been removed to increase the readability of the manuscript.

There also confusion between genes *mcr-1* (in italics) and the protein, MCR-1 (which technically should be referred to as Mcr-1 in keeping to the conventional naming of bacterial proteins). Also, in several occasions, the authors attribute to M6 an activity that is produced by the bacteria expressing this protein.

Reply: We sincerely thank the suggestion from reviewer. However, the protein name of *mcr-1* gene should be “MCR-1”, please refer to previous articles as reference[1-4]. Meanwhile, we have polished the writing of the manuscript to improve the accuracy of the expression.

Finally, some adjectives, like "severe" are subjective.

Reply: We sincerely thank the suggestion from reviewer and we have corrected the related mistakes in the manuscript. The demonstrations including the word of “severe” in the manuscript are as followed:

Line 54: “Compared to wild-type (WT) MCR-1, this variant caused severe disturbance in lipid A…”

Line 179-180: “…suggested that the expression of M6 contributed to more severe membrane perturbation compared to MCR-1.”

Line 295-296: “SEM revealed that both 19AA-2M-tag and 24AA-2M induced severe OM shrinkage in *E. coli*.”

Line 320-321: “…the mutant protein confers phenotypic co-resistance to β-lactam antibiotics while inducing severe lipid A perturbations.”

Line 372-373: “First, our findings indicate that severe membrane damage occurs by M6 induced activation of LDTs pathway…”

There is a fair bit to rewrite but considering that English is not the mother tongue of the authors, I have taken the liberty to provide an edited copy for the authors' consideration (uploaded with this review), hoping it can help them to rework the manuscript and bring it to an adequate standard. I have not edited the figure legends, but they also need some work.

Reply: We sincerely thank the help from reviewer and we have polished the writing of the manuscript based on the suggestions.

**References:**

1.	Daly SM, Sturge CR, Felder-Scott CF, Geller BL, Greenberg DE. MCR-1 Inhibition with Peptide-Conjugated Phosphorodiamidate Morpholino Oligomers Restores Sensitivity to Polymyxin in Escherichia coli. mBio. 2017;8(6). Epub 2017/11/09. doi: 10.1128/mBio.01315-17. PubMed PMID: 29114023; PubMed Central PMCID: PMCPMC5676038.

2.	Gao R, Hu Y, Li Z, Sun J, Wang Q, Lin J, et al. Dissemination and Mechanism for the MCR-1 Colistin Resistance. PLoS Pathog. 2016;12(11):e1005957. Epub 2016/11/29. doi: 10.1371/journal.ppat.1005957. PubMed PMID: 27893854; PubMed Central PMCID: PMCPMC5125707.

3.	Kieffer N, Aires-de-Sousa M, Nordmann P, Poirel L. High Rate of MCR-1-Producing Escherichia coli and Klebsiella pneumoniae among Pigs, Portugal. Emerg Infect Dis. 2017;23(12):2023-9. Epub 2017/11/18. doi: 10.3201/eid2312.170883. PubMed PMID: 29148380; PubMed Central PMCID: PMCPMC5708242.

4.	Sun Z, Palzkill T. Deep Mutational Scanning Reveals the Active-Site Sequence Requirements for the Colistin Antibiotic Resistance Enzyme MCR-1. mBio. 2021;12(6):e0277621. Epub 2021/11/17. doi: 10.1128/mBio.02776-21. PubMed PMID: 34781730; PubMed Central PMCID: PMCPMC8593676.
